# A cuproptosis-inducing covalent organic framework synergizes with oncolytic virus for tumor radiotherapy

**DOI:** 10.1038/s41467-026-76947-w

**Published:** 2026-08-21

**Authors:** Yuan-Tong Liu, Ke-Shan Liu, Han Li, Shuo Wang, Shu-Ying Bie, Ying-Qi Wei, Shan-Shan Gou, Xiang-Bo Wan, Xing-Zhen Li, Liang Zhang, Wei He

**Affiliations:** 1 Department of Stomatology, the First Affiliated Hospital of Zhengzhou University, Zhengzhou, China; 2 State Key Laboratory of Metabolic Dysregulation & Prevention and Treatment of Esophageal Cancer, Tianjian Laboratory of Advanced Biomedical Sciences, Academy of Medical Sciences, Zhengzhou University, Zhengzhou, China; 3 State Key Laboratory of Precision and Intelligent Chemistry, School of Chemistry and Materials Science, University of Science and Technology of China, Hefei, China

**Keywords:** Cancer immunotherapy, Biomedical materials, Nanotechnology in cancer, Radiotherapy

## Abstract

Acquired radioresistance limits effective radiotherapy. Cuproptosis is a copper-dependent form of cell death driven by mitochondrial copper accumulation and lipoylated protein aggregation. Building on evidence that radioresistant cancer cells exhibit increased cuproptosis susceptibility, here we combine a radiation-responsive, copper-incorporated covalent organic framework (COF‑Tpy‑Se‑Cu) with an oncolytic adenovirus to target this vulnerability. X-ray irradiation triggers copper release from COF‑Tpy‑Se‑Cu and induces FDX1-dependent cuproptosis, while the oncolytic adenovirus depletes intracellular glutathione and stabilizes FDX1 by limiting its mitochondrial protease-mediated degradation. The combination enhances tumor cell death and remodels the immunosuppressive tumor microenvironment into a T-cell-inflamed state. In models of radioresistant tumors in female mice, this regimen improves tumor control and elicits CD8^+^ T cell-dependent systemic antitumor immunity and durable immunological memory. These findings support the combined use of radiation-responsive copper delivery and oncolytic virotherapy as a strategy for exploiting cuproptosis susceptibility and improving radiotherapy responses.

## Abstract (web summary)

It has been reported that cuproptosis inducers can sensitize radioresistant tumors to radiotherapy (RT). Here, the authors report the synergistic anti-tumor efficacy of an oncolytic adenovirus combined with a radiation-responsive, copper-incorporated covalent organic framework, overcoming radio-resistance via induction of cuproptosis.

## Introduction

Radiotherapy (RT) is a cornerstone treatment for localized and oligometastatic cancers, with more than half of patients requiring RT during their treatment course^1–3^. However, its clinical efficacy is frequently compromised by the development of acquired radioresistance in surviving tumor cells following fractionated irradiation^4,5^. This resistance impairs local tumor control and elevates the risk of locoregional recurrence, often necessitating re‑irradiation (reRT) of previously treated sites. Curative reRT is constrained by the cumulative tolerance of adjacent normal tissues, thereby increasing the likelihood of severe toxicities—including radiation pneumonitis, necrosis, and cognitive impairment—particularly in radiosensitive organs such as the breast, lung, and brain^6–8^. Accordingly, overcoming acquired radioresistance without exacerbating normal-tissue injury remains an urgent unmet need in radiation oncology.

Emerging evidence suggests that radiotherapy can induce cuproptosis, a copper-dependent, mitochondria-mediated cell death program, and that disruption of this pathway contributes to radioresistance^9,10^. Cuproptosis is initiated by intracellular copper overload, specifically within the mitochondria, where excess copper binds to lipoylated enzymes in the tricarboxylic acid cycle, resulting in proteotoxic stress and eventual cell death^11–13^. This process is regulated by ferredoxin 1 (FDX1), which catalyzes the reduction of Cu^2+^ to Cu^+^ and subsequently promotes the lipoylation of downstream proteins^14–16^. Leveraging nanomaterials to induce intracellular copper overload and FDX1-mediated cuproptosis can promote dendritic cell (DC) maturation and CD8^+^ T cell recruitment, thereby enhancing the efficacy of anti-programmed cell death-1 (PD-1) therapy^17–19^. However, the application of nanomaterial-induced cuproptosis relies on endogenous FDX1 expression within tumors. Notably, FDX1 expression decreases during cuproptosis, suggesting a negative-feedback mechanism that may limit sustained pathway activation^11,19,20^. Furthermore, the efficacy of this approach can be limited by potential off-target effects of the materials, insufficient tumor penetration, and the dual role of copper in modulating the immunosuppressive tumor microenvironment^17,20,21^. The combination of nanodrugs with oncolytic virotherapy has emerged as a promising strategy to address these limitations.

Oncolytic viruses, exemplified by oncolytic adenoviruses (OVADs), represent a promising anticancer modality due to their capacity to remodel the immunosuppressive tumor microenvironment and elicit potent antitumor immunity^22^. Through release of tumor-associated antigens and damage-associated molecular patterns (DAMPs), oncolytic viruses engage both innate and adaptive immune compartments, thereby shifting immunologically non-inflamed tumors toward a T-cell-inflamed phenotype^23,24^. Moreover, local intratumoral administration of oncolytic viruses can reduce the off-target effects on normal tissues associated with systemic drug delivery, while also overcoming the dense physiological barriers that often impede the penetration of conventional therapeutics^22,24,25^. Although oncolytic viruses can initiate antitumor immunity, their efficacy as monotherapy is constrained by inherent limitations, including incomplete tumor cell killing and an inability to eliminate therapy-resistant cell populations^22,25–27^. These shortcomings highlight the imperative for developing more potent combination strategies.

**Fig. 1:**
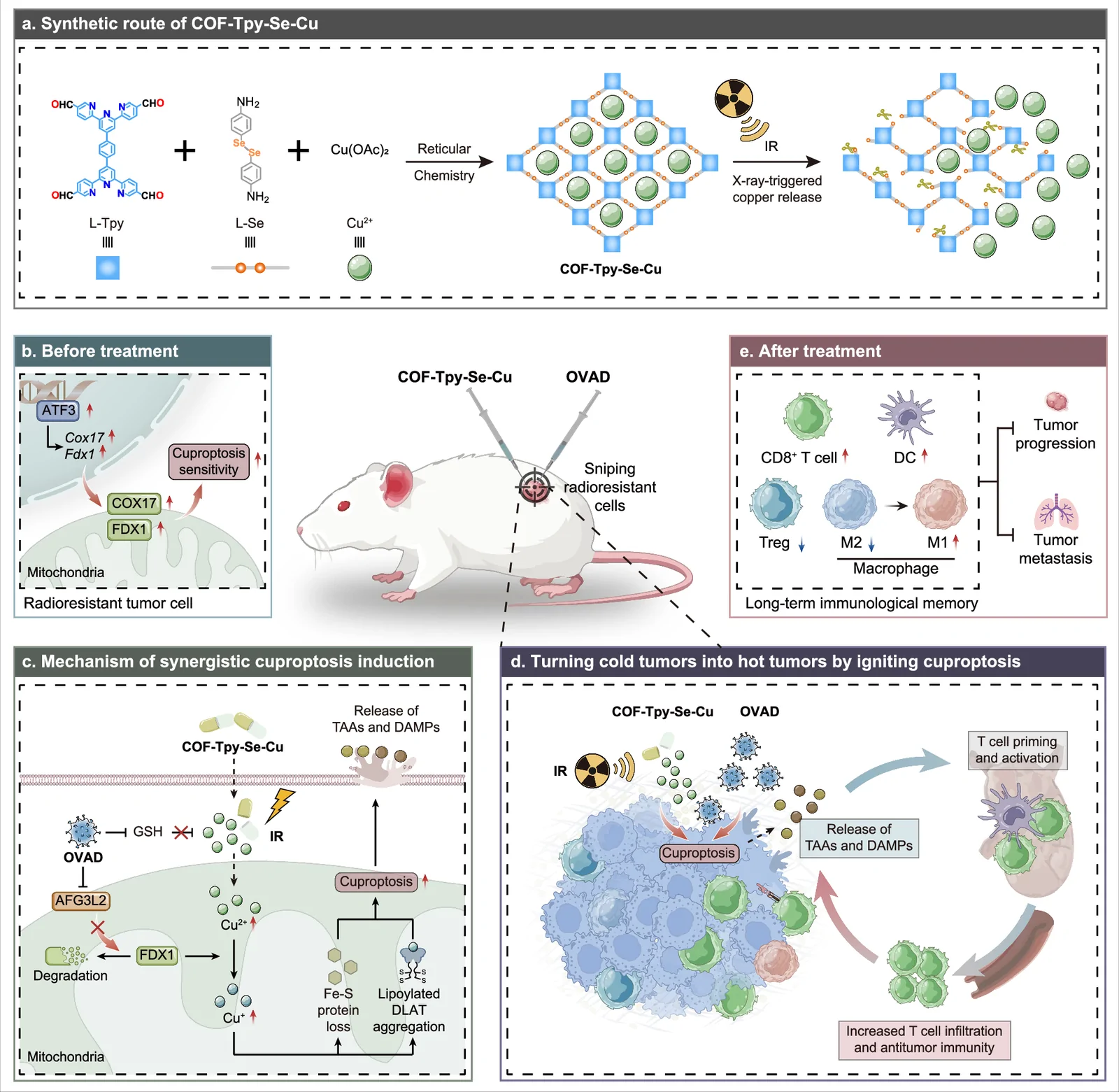
Schematic illustration of COF‑Tpy‑Se‑Cu combined with oncolytic adenovirus to overcome radioresistance through cuproptosis induction and remodeling of the tumor immune microenvironment. **a** Synthetic route of COF‑Tpy‑Se‑Cu and schematic illustration of X‑ray-triggered copper release. **b** Proposed basis for the increased cuproptosis susceptibility of radioresistant tumor cells. **c** Proposed mechanism by which X‑ray-irradiated COF‑Tpy‑Se‑Cu and oncolytic adenovirus (OVAD) cooperatively induce cuproptosis. COF‑Tpy‑Se‑Cu releases copper following irradiation, whereas OVAD depletes intracellular glutathione (GSH) and stabilizes FDX1. **d** Schematic illustration of cuproptosis-associated release of tumor-associated antigens (TAAs) and damage-associated molecular patterns (DAMPs), followed by T cell priming, activation, and increased tumor infiltration. **e** This combination regimen elicits a T cell-dependent antitumor response that suppresses metastasis and establishes durable immunological memory.

In this work, we investigate a therapeutic strategy that targets the increased cuproptosis susceptibility of radioresistant cancer cells by combining a radiation-responsive, copper-incorporated covalent organic framework (COF‑Tpy‑Se‑Cu) with OVAD. Upon X-ray irradiation, COF‑Tpy‑Se‑Cu releases copper ions to trigger FDX1-mediated cuproptosis, while OVAD depletes intracellular glutathione and stabilizes FDX1 by limiting mitochondrial protease-mediated degradation. The combination enhances cuproptosis, remodels the immunosuppressive tumor microenvironment toward a T-cell-inflamed state, and elicits systemic antitumor immunity. Together, these findings support further evaluation of radiation-responsive copper delivery combined with oncolytic virotherapy for acquired radioresistance (Fig. 1).

## Results

### Radioresistant cells exhibit enhanced susceptibility to cuproptosis

**Fig. 2:**
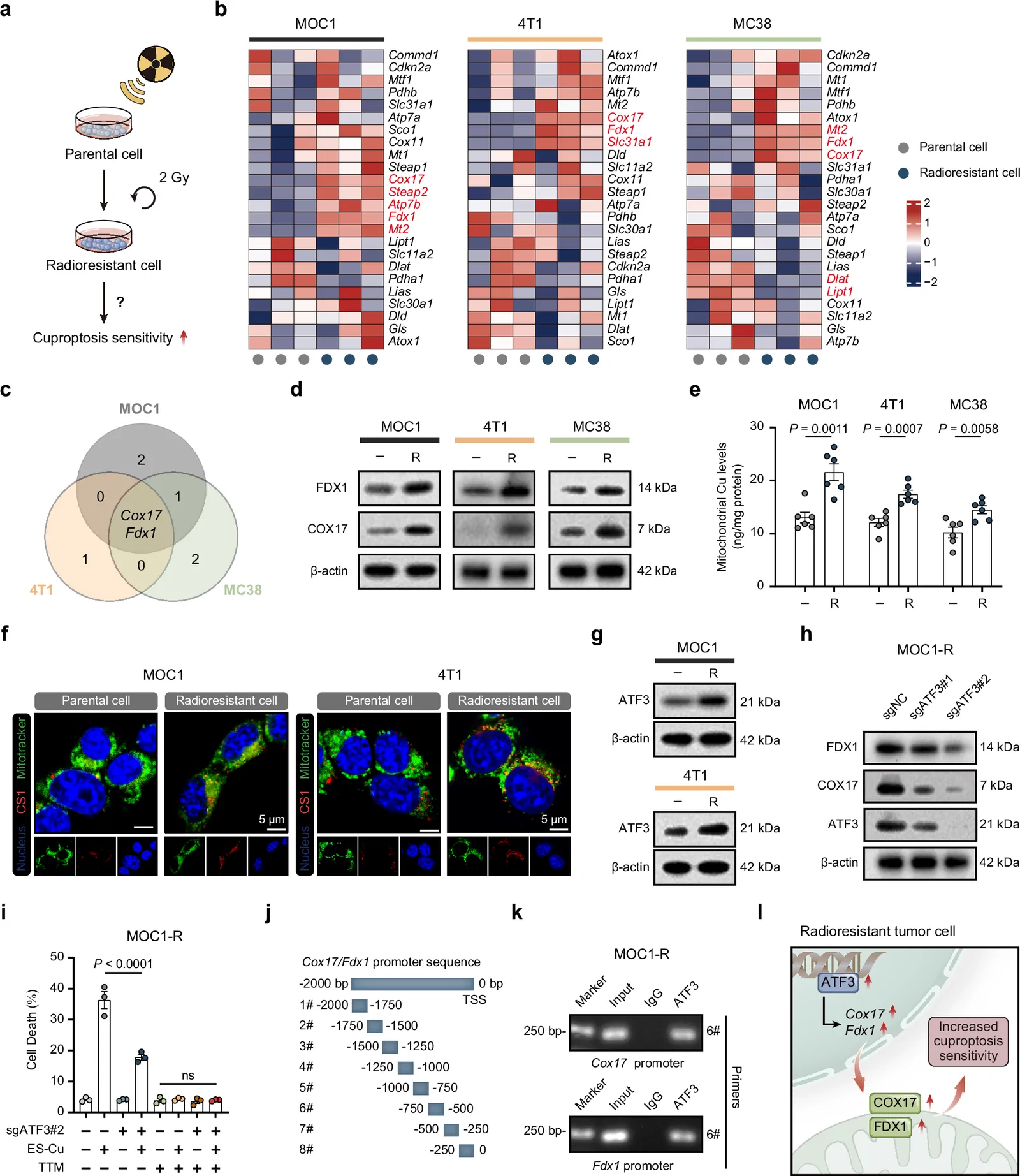
Radioresistant cancer cells display increased susceptibility to cuproptosis. **a** Schematic of the fractionated irradiation regimen (2 Gy per fraction) used to generate radioresistant cell lines. **b** Heatmap depicting the relative mRNA expression of cuproptosis-related and copper-homeostasis genes in three matched pairs of parental and radioresistant tumor cell lines, as determined by quantitative PCR. **c** Venn diagram showing the overlap of differentially expressed genes between radioresistant and parental cells. *Fdx1* and *Cox17* were consistently upregulated across all three radioresistant lines. **d** Immunoblot analysis of FDX1 and COX17 in parental versus radioresistant cells (representative of three independent experiments). **e** Mitochondrial copper levels quantified by ICP–MS in parental and radioresistant MOC1, 4T1, and MC38 cells and normalized to mitochondrial protein (*n* = 6 independent experiments). **f** Confocal images of MOC1 and 4T1 cells stained with the copper-selective probe CopperSensor-1 and the mitochondrial dye MitoTracker (representative of three independent experiments) (scale bar, 5 μm). **g** Immunoblot analysis of ATF3 in parental and radioresistant cells (representative of three independent experiments). **h** Immunoblot of the indicated proteins in MOC1‑R cells transduced with sgNC or sgATF3 (representative of three independent experiments). **i** Flow cytometric quantification of cell death in MOC1‑R cells following the indicated treatments (ES-Cu, 200 nM, 24 h; TTM, 40 μM, 24 h) (*n* = 3 independent experiments). **j** Schematic of primer sets spanning the *Cox17* and *Fdx1* promoter regions used for chromatin immunoprecipitation (ChIP). **k** Agarose gel electrophoresis confirming amplification of *Cox17* and *Fdx1* promoter fragments from ATF3 ChIP samples (IgG as negative control) (representative of three independent experiments). **l** Working model: ATF3 occupies the proximal promoter regions of *Fdx1* and *Cox17* and promotes their transcription, thereby increasing cuproptosis sensitivity. Statistical significance was assessed using a two-tailed Student’s *t* test (**e**) or one-way ANOVA with Tukey’s multiple-comparisons test (**i**). Data are presented as mean ± s.e.m. Source data are provided as a Source Data file.

Acquired radioresistance is a major barrier to effective radiotherapy^28^. To investigate this phenotype and evaluate candidate therapeutic strategies, we selected a panel of three representative tumor cell lines encompassing distinct histological origins and radiobiological contexts: MOC1 (murine head and neck squamous cell carcinoma), 4T1 (murine triple‑negative breast cancer), and MC38 (murine colon adenocarcinoma). This panel was chosen to encompass diverse tissue origins, established clinical radiotherapy indications, and distinct baseline immune microenvironments, enabling evaluation of both antitumor efficacy and the broader generalizability of our approach^29–33^. An additional murine esophageal squamous cell carcinoma line, AKR, was included for parallel mechanistic validation. To uniformly recapitulate the clinical scenario of acquired radioresistance, we derived isogenic radioresistant sublines from each parental line through long‑term fractionated irradiation (repeated irradiation at 2 Gy per fraction) (Fig. 2a). These irradiated lines (MOC1-R, 4T1-R, MC38-R, and AKR-R) exhibited radioresistance, as demonstrated by significantly higher clonogenic survival compared to their parental controls (Supplementary Fig. 1a). We also developed an in vivo radioresistant tumor model by implanting 4T1-R cells. Unlike parental 4T1 tumors, 4T1-R tumors responded poorly to irradiation (4 Gy × 2), confirming acquired radioresistance in vivo (Supplementary Fig. 1b, c).

Emerging evidence indicates that radiotherapy can induce cuproptosis, a regulated, copper-dependent form of cell death driven by mitochondrial dysfunction^9^. We therefore asked whether the development of radioresistance alters the cellular sensitivity to this pathway (Fig. 2a). Notably, radioresistant cells were more sensitive to the canonical cuproptosis inducer elesclomol–Cu (ES-Cu) than their parental counterparts (Supplementary Fig. 2a–e). To elucidate the mechanistic basis for this differential sensitivity, we profiled expression of cuproptosis and copper homeostasis genes (Fig. 2b). A comparison across radioresistant lines (MOC1-R, 4T1-R, and MC38-R) identified a core set of upregulated genes, with *Fdx1* and *Cox17* consistently elevated in all models (Fig. 2c). Immunoblotting confirmed higher FDX1 and COX17 protein levels (Fig. 2d). FDX1 catalyzes the reduction of Cu^2+^ to the more toxic Cu^+^, while COX17 facilitates mitochondrial copper uptake^14^. Both processes are required for the execution of cuproptosis^14^. Accordingly, radioresistant cells had significantly higher mitochondrial copper levels, measured by inductively coupled plasma mass spectrometry (ICP‑MS) and immunofluorescence (Fig. 2e, f). Together, these results connect acquired radioresistance to a heightened vulnerability to cuproptosis, driven by the coordinated upregulation of FDX1 and COX17 and the resulting mitochondrial copper overload.

### ATF3 contributes to cuproptosis sensitivity in radioresistant cells by transcriptionally regulating FDX1 and COX17

To explore the upstream regulation of FDX1 and COX17, we performed RNA sequencing using MOC1 cells selected through fractionated irradiation and their corresponding parental control. By analyzing differentially expressed transcription factors and integrating these data with transcription factor-binding predictions from the JASPAR database, we identified ATF3 as a candidate upstream regulator (Supplementary Fig. 3a). Consistent with this prediction, ATF3 expression was increased in MOC1-R and 4T1-R cells relative to their parental counterparts (Fig. 2g and Supplementary Fig. 3b). We next evaluated the functional role of ATF3 by generating ATF3-knockout clones in MOC1-R and 4T1-R cells using CRISPR/Cas9. Deletion of ATF3 reduced both mRNA and protein levels of FDX1 and COX17 (Fig. 2h and Supplementary Fig. 3c, d). Accordingly, ATF3-deficient cells exhibited significantly attenuated sensitivity to ES-Cu, indicating that ATF3 is required for the heightened cuproptosis susceptibility of radioresistant cells (Fig. 2i and Supplementary Fig. 3e).

ATF3 is a stress-inducible transcription factor implicated in immune regulation, metabolism, and adaptive stress responses in cancer^34–36^. We therefore asked whether ATF3 regulates *Fdx1* and *Cox17* transcription through their promoter regions. Chromatin immunoprecipitation (ChIP) assays revealed significant ATF3 occupancy at discrete promoter regions of both genes, with the highest enrichment located approximately 750–500 bp upstream of the transcription start site (Fig. 2j, k and Supplementary Fig. 4a, b). JASPAR analysis identified candidate ATF3-binding motifs within these regions (Supplementary Fig. 4c). To test their functional relevance, we performed dual-luciferase reporter assays (Supplementary Fig. 4d). In MOC1-R cells overexpressing ATF3, the wild-type *Cox17* promoter showed increased promoter activity. This effect was abolished by mutation of a single motif (MUTB), whereas mutations at adjacent motifs (MUTA and MUTC) were without effect (Supplementary Fig. 4d). Similarly, ATF3-driven activation of the *Fdx1* promoter was significantly reduced by mutation of two motifs (MUTB and MUTC) (Supplementary Fig. 4d). These results indicate that ATF3 transactivates FDX1 and COX17 through specific cis-regulatory elements.

Together, these findings support a regulatory role for ATF3 in *Fdx1* and *Cox17* transcription and provide mechanistic context for the increased cuproptosis susceptibility of radioresistant cells (Fig. 2l). This susceptibility was subsequently exploited through the combined therapeutic strategy evaluated in this study.

### Synthesis and characterization of COF‑Tpy‑Se‑Cu

**Fig. 3:**
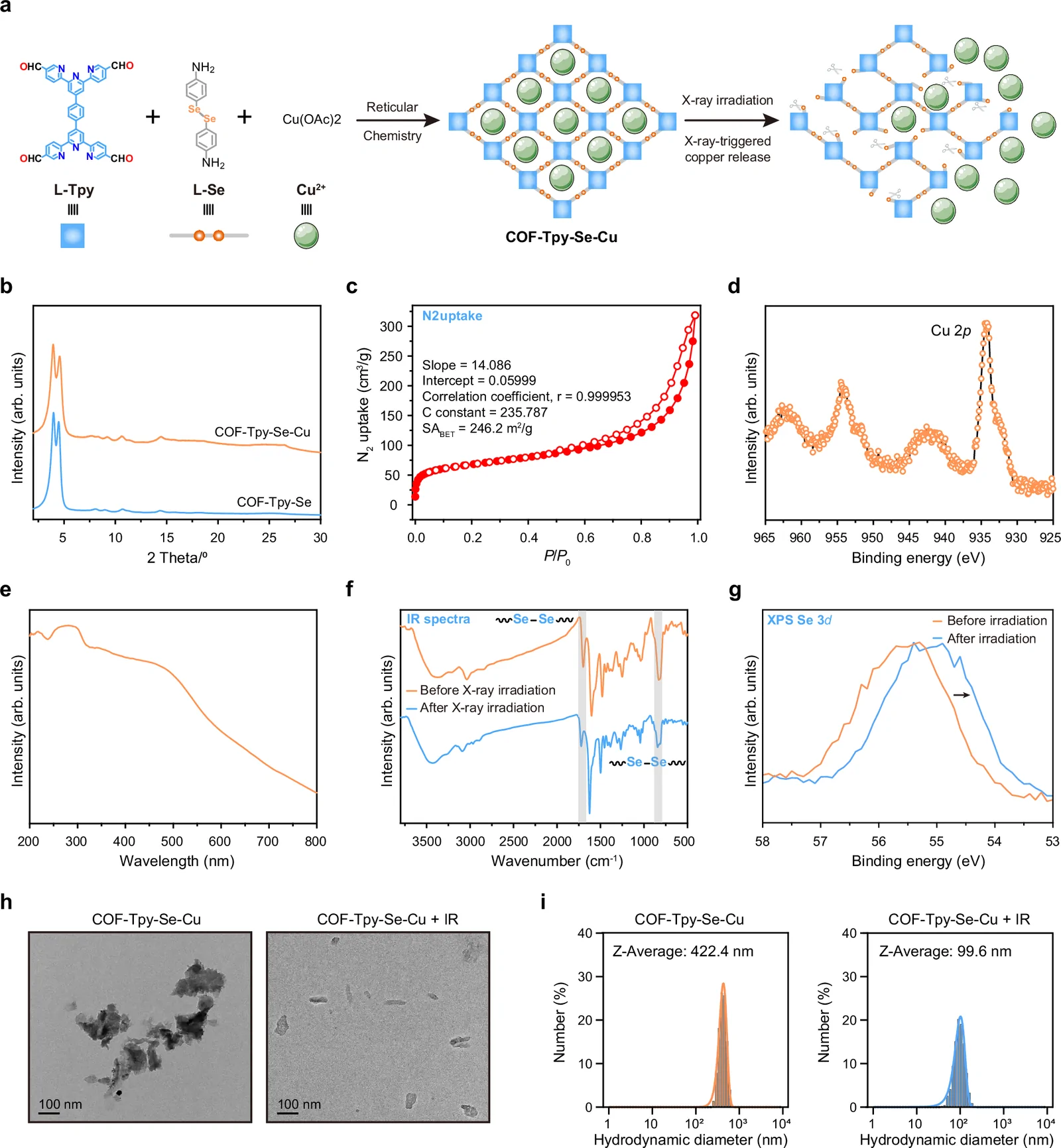
Synthesis and characterization of the radiation-responsive diselenide-bonded COF, COF‑Tpy‑Se‑Cu. **a** Schematic of the synthetic route to COF‑Tpy‑Se‑Cu and its X‑ray–triggered copper-release behavior. **b** Powder X‑ray diffraction (PXRD) patterns of COF‑Tpy‑Se and COF‑Tpy‑Se‑Cu (representative of three independent measurements). **c** N_2_ adsorption–desorption isotherm of COF‑Tpy‑Se‑Cu (representative of three independent measurements). **d** X‑ray photoelectron spectroscopy (XPS) spectrum of COF‑Tpy‑Se‑Cu in the Cu 2*p* region (representative of three independent measurements). **e** UV‑Vis absorption spectrum of COF‑Tpy‑Se‑Cu (representative of three independent measurements). **f** FT‑IR spectra of COF‑Tpy‑Se‑Cu before and after X‑ray irradiation (4 Gy) (representative of three independent measurements). **g** Se 3 *d* XPS spectra of COF‑Tpy‑Se‑Cu before and after X‑ray irradiation (4 Gy) (representative of three independent measurements). **h** Transmission electron microscopy (TEM) images of COF‑Tpy‑Se‑Cu before and after X‑ray irradiation (4 Gy) (representative of three independent measurements). **i** Dynamic light scattering (DLS) profiles of COF‑Tpy‑Se‑Cu before and after X‑ray irradiation (4 Gy) (representative of three independent measurements). Source data are provided as a Source Data file.

Covalent organic frameworks (COFs) possess tunable structures and readily functionalizable properties, making them effective carriers for targeted therapeutics in tumor immunotherapy and radiotherapy^31,37,38^. COF-Tpy-Se-Cu was synthesized by solvothermal condensation of the diamine ligand bis(4-aminophenyl)perselenide (L-Se) with the tetra-aldehyde ligand 4’,4”“-(1,4-phenylene)bis(([2,2’:6’,2”-terpyridine]-5,5”-dicarbaldehyde)) (L-Tpy) in the presence of copper(II) acetate (Fig. 3a and Supplementary Fig. 5). The design of COF-Tpy-Se-Cu incorporates the diselenide bond (-Se-Se-) as an X-ray-sensitive responsive unit, while Cu^2+^ ions are integrated as active centers within the framework’s periodic structure (Fig. 3a). The resulting material was comprehensively characterized using complementary spectroscopic and structural analyses.

Powder X-ray diffraction (PXRD) provided critical structural insights into both COF-Tpy-Se and COF-Tpy-Se-Cu (Fig. 3b). The PXRD pattern revealed two prominent diffraction peaks, with the most intense reflections observed at 3.9° and 4.6°, corresponding to the (100) and (110) planes, respectively, confirming the periodicity and high crystallinity of COF-Tpy-Se-Cu. The Brunauer-Emmett-Teller (BET) surface area of COF-Tpy-Se-Cu was determined to be 246.2 m² g⁻¹, consistent with its permanent porosity (Fig. 3c). X-ray photoelectron spectroscopy (XPS) displayed characteristic signals at 934.24 eV for Cu 2p and 55.51 eV for Se 3 d (Fig. 3d, g and Supplementary Fig. 6). In addition, UV–Vis absorption spectroscopy revealed optical features characteristic of the COF framework, while Fourier-transform infrared (FT-IR) spectroscopy exhibited a notable absorption peak at 1610 cm⁻¹, attributed to the imine bond (-C = N-) (Fig. 3e, f).

To assess the radiation responsiveness, FT-IR spectra showed a decrease in the intensity of the –Se–Se– stretching vibration following X-ray irradiation, indicating the cleavage of the diselenide bond (Fig. 3f). Corresponding changes in the chemical state of selenium were confirmed by XPS analysis, which showed a redshift in the characteristic diselenide peak after irradiation (Fig. 3g). Furthermore, transmission electron microscopy (TEM) imaging and dynamic light scattering (DLS) analyses revealed a change in particle size after irradiation, suggesting radiation-triggered disassembly or structural disruption of the COF (Fig. 3h, i).

To further verify the structural stability and radiation‑specific copper release profile, we monitored copper release kinetics over 72 h under a panel of pathophysiologically relevant conditions, with or without 4 Gy X‑ray irradiation (Supplementary Fig. 7). In the absence of irradiation, negligible spontaneous copper release was observed across all tested conditions, including neutral physiological buffer, 10% serum, 10 mM glutathione (GSH), 100 μM hydrogen peroxide (H_2_O_2_), and acidic buffers (pH 6.5 and pH 5.0) mimicking the tumor microenvironment. These results confirm that COF‑Tpy‑Se‑Cu maintains structural stability and exhibits minimal non‑specific copper leakage when exposed to the redox, pH, and protein gradients characteristic of tumor tissue. By contrast, time‑dependent copper release was consistently detected across all conditions upon X‑ray irradiation, verifying that copper liberation is primarily triggered by radiation (Supplementary Fig. 7).

Collectively, these findings support the synthesis of a crystalline, porous, and radiation-responsive copper-incorporated covalent organic framework, in which the diselenide linker enables controlled copper release upon X-ray irradiation.

### COF‑Tpy‑Se‑Cu exerts in vitro radiosensitization effects via controlled induction of cuproptosis

**Fig. 4:**
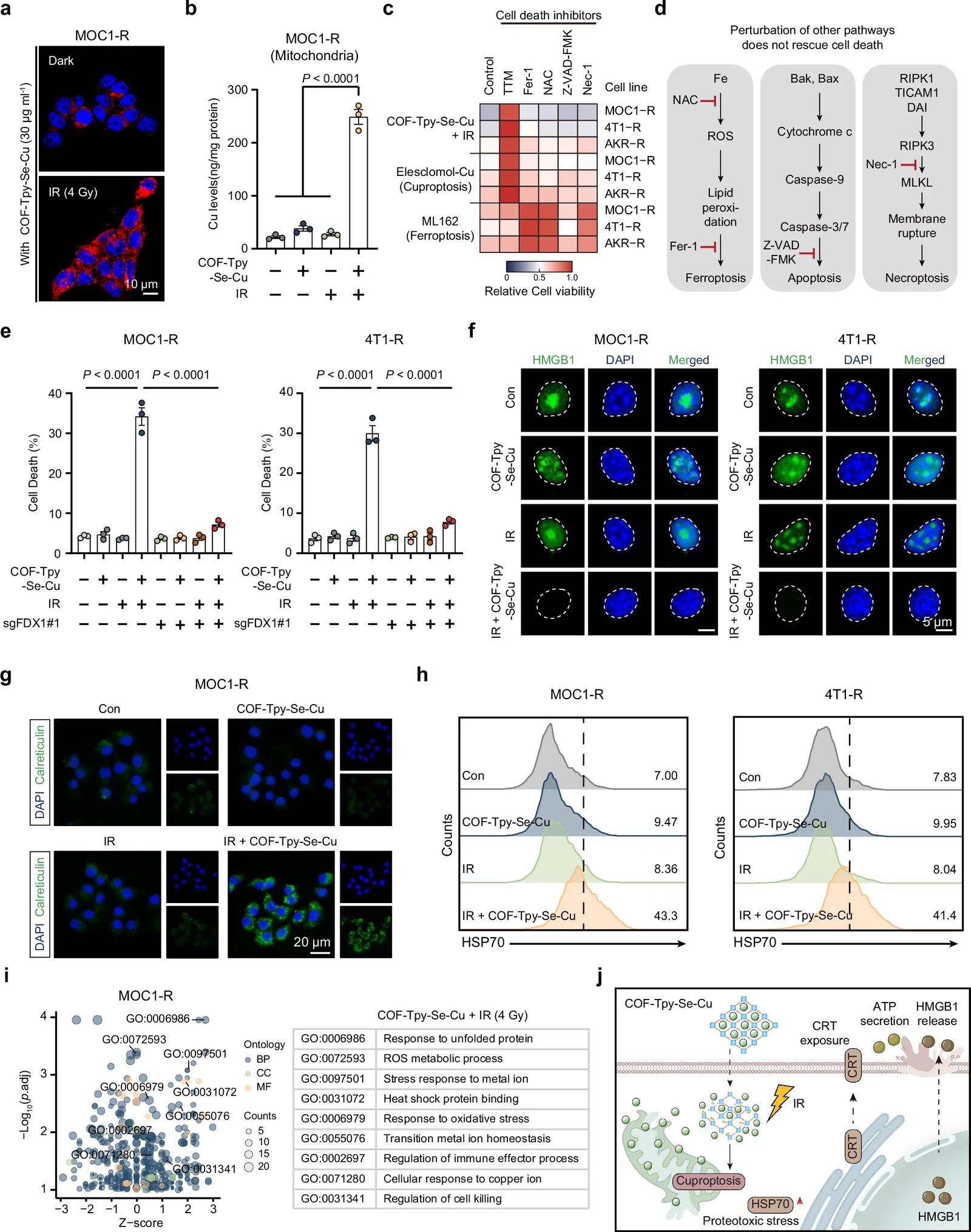
Mechanisms underlying COF‑Tpy‑Se‑Cu–mediated radiosensitization in vitro. **a** Representative confocal images of MOC1‑R cells stained with the copper-selective probe CopperSensor‑1 (red) after treatment with COF‑Tpy‑Se‑Cu (30 μg mL^-1^, 12 h) with or without irradiation (IR, 4 Gy) (representative of three independent experiments). Scale bar, 10 μm. **b** Mitochondrial copper levels in MOC1‑R cells quantified by ICP–MS and normalized to mitochondrial protein (*n* = 3 independent experiments). **c** Heatmap of cell viability after 24 h of co-treatment with COF‑Tpy‑Se‑Cu (30 μg mL^-1^) plus IR (4 Gy), elesclomol–Cu (200 nM), or ML162 (1 μM), in the presence or absence of inhibitors of ferroptosis (Fer‑1, 10 μM), apoptosis (Z‑VAD‑FMK, 20 μM), necroptosis (Nec‑1, 20 μM), oxidative stress (NAC, 1 mM), or copper chelation (TTM, 40 μM). Blue denotes reduced viability and red denotes higher viability (means of three independent experiments). **d** Schematic of the core signaling pathways regulating ferroptosis, apoptosis, and necroptosis. Inhibitory arrows indicate blockade by the corresponding inhibitors. **e** Flow cytometric quantification of cell death in MOC1‑R and 4T1‑R cells 24 h after treatment with COF‑Tpy‑Se‑Cu (30 μg mL^-1^) plus IR (4 Gy) (*n* = 3 independent experiments). **f** Confocal immunofluorescence images showing HMGB1 (green) release in MOC1‑R and 4T1‑R cells under the indicated conditions (*n* = 10 independent fields per condition across three independent experiments). Scale bar, 5 μm. **g** Immunofluorescence detection of surface-exposed calreticulin (CRT) (green) in MOC1‑R cells (*n* = 3 independent experiments). Scale bar, 20 μm. **h** Flow cytometric quantification of the relative HSP70‑positive cell subset ratio in MOC1‑R and 4T1‑R cells following the indicated treatments (*n* = 3 independent experiments). **i** Gene Ontology (GO) enrichment analysis of genes associated with the response of MOC1‑R cells to COF‑Tpy‑Se‑Cu (30 μg mL^−1^) plus IR (4 Gy) for 24 h (*n* = 3 independent experiments). **j** Working model: COF‑Tpy‑Se‑Cu functions as a radiosensitizer by inducing cuproptosis, which promotes immunogenic cell death via release of damage-associated molecular patterns (DAMPs) and thereby enhances antitumor immunity. Statistical significance was assessed using one-way ANOVA with Tukey’s multiple-comparisons test (**b**, **e**). Data are presented as mean ± s.e.m. Source data are provided as a Source Data file.

We first validated that X‑ray irradiation triggers copper release from COF‑Tpy‑Se‑Cu in radioresistant cells. Using the copper‑selective probe CopperSensor‑1, we observed a pronounced increase in fluorescence in cells treated with COF‑Tpy‑Se‑Cu followed by irradiation compared with COF‑Tpy‑Se‑Cu alone (Fig. 4a). Consistently, ICP‑MS quantification revealed that COF‑Tpy‑Se‑Cu combined with irradiation significantly elevated both total intracellular and mitochondrial copper levels in MOC1‑R and 4T1‑R cells (Fig. 4b and Supplementary Fig. 8a, b), whereas either treatment alone produced minimal changes (Fig. 4b and Supplementary Fig. 8a, b).

We next assessed the radiosensitizing activity of COF‑Tpy‑Se‑Cu. The copper-free analogue COF‑Tpy‑Se exhibited negligible cytotoxicity with or without irradiation (Supplementary Fig. 8c), whereas COF‑Tpy‑Se‑Cu produced a concentration-dependent loss of viability upon irradiation (Supplementary Fig. 8d). We further validated the radiosensitizing effect using the clonogenic survival assay, the gold standard for assessing long‑term proliferative capacity and radiotherapeutic efficacy. In both MOC1‑R and 4T1‑R radioresistant cell lines, the combination of COF‑Tpy‑Se‑Cu and irradiation effectively suppressed colony formation compared with either monotherapy (Supplementary Fig. 9a, b). Given that aldehyde dehydrogenase (ALDH)‑positive cancer stem cells (CSCs) represent a subpopulation tightly associated with radioresistance, we specifically investigated whether the combination therapy could target this therapy‑resistant compartment^39–41^. The combination of COF‑Tpy‑Se‑Cu and irradiation markedly reduced the proportion of ALDH-positive CSCs, demonstrating efficacy against this typically resistant cell population (Supplementary Fig. 9c).

To define the death modality, we applied inhibitors of major regulated cell death pathways, including ferrostatin‑1 (ferroptosis), N‑acetylcysteine (oxidative stress), Z‑VAD‑FMK (apoptosis), and necrostatin‑1 (necroptosis). None of these agents rescued the cytotoxicity induced by COF‑Tpy‑Se‑Cu plus irradiation (Fig. 4c, d). By contrast, the copper chelator tetrathiomolybdate (TTM) completely abrogated cell death, indicating a copper-dependent mechanism (Fig. 4c, d). Moreover, CRISPR/Cas9-mediated deletion of FDX1 in radioresistant MOC1‑R, 4T1‑R, and AKR‑R cells eliminated the cytotoxic effect of the combination treatment (Fig. 4e and Supplementary Fig. 10a–c). Together, these data indicate that COF‑Tpy‑Se‑Cu functions as a radiosensitizer by releasing cytotoxic copper in a radiation-controlled manner and selectively triggering FDX1-dependent cuproptosis.

Because cuproptosis may exhibit features of immunogenic cell death, we next evaluated whether COF‑Tpy‑Se‑Cu combined with irradiation elicited canonical DAMPs^10,19^. In radioresistant tumor cells, the combination treatment significantly increased extracellular high mobility group box 1 (HMGB1) release (Fig. 4f and Supplementary Fig. 11a) and enhanced calreticulin (CRT) exposure on the cell surface in MOC1‑R cells relative to controls (Fig. 4g). Extracellular ATP and lactate dehydrogenase (LDH) levels were likewise elevated (Supplementary Fig. 11b, c), and flow cytometric analysis confirmed upregulation of intracellular heat shock protein 70 (HSP70) (Fig. 4h and Supplementary Fig. 11d). Consistent with these observations, RNA sequencing of treated tumor cells showed enrichment of pathways associated with metal-ion responses, unfolded protein responses, heat-shock protein binding, and regulation of immune effector processes (Fig. 4i). Taken together, these results indicate that cuproptosis induced by COF‑Tpy‑Se‑Cu plus irradiation is accompanied by robust DAMP emission, supporting the immunostimulatory potential of this therapeutic strategy (Fig. 4j).

### COF‑Tpy‑Se‑Cu mediates radiosensitization in vivo

We next evaluated the in vivo biodistribution and excretion profiles of COF‑Tpy‑Se‑Cu following intratumoral administration. For semi-quantitative ex vivo fluorescence assessment, Cy5.5 was loaded into the COF pores to serve as a fluorescent tracer. Major organs and tumor tissues were harvested at 7 days post‑injection for ex vivo fluorescence imaging (Supplementary Fig. 12a). In both MOC1‑R orthotopic and 4T1‑R subcutaneous tumor models, fluorescent signals were predominantly retained in tumor tissues, with apparent accumulation also detected in the liver and spleen, and negligible signals observed in the heart, lungs, and kidneys (Supplementary Fig. 12b). Quantitative analysis of average radiant efficiency confirmed that the signal intensity in tumors was significantly higher than that in all examined normal organs (Supplementary Fig. 12b), demonstrating the favorable local retention effect of intratumoral delivery. For accurate, time‑resolved quantification of biodistribution, we used an independent cohort of tumor‑bearing mice that received unlabeled native COF‑Tpy‑Se‑Cu (without Cy5.5 loading). Tissues were harvested at serial time points, weighed, and subjected to acid digestion. Selenium, an intrinsic elemental constituent of the COF backbone, was used as an elemental tracer, and its content in each tissue was measured via ICP‑MS. Among the normal organs examined, the material preferentially accumulated in the major mononuclear phagocyte system (MPS) organs (i.e., the liver and spleen), with tissue concentrations peaking between days 7 and 14 followed by gradual clearance (Supplementary Fig. 12c). Only minimal amounts of the material were detected in the heart, lungs, and kidneys throughout the 30‑day monitoring period (Supplementary Fig. 12c).

To define the excretion profile, we monitored daily fecal and urinary excretion rates over 30 days using metabolic cages, with selenium content in fecal and urinary samples quantified by ICP‑MS using the same elemental tracer strategy described above. The daily fecal excretion rate exhibited an initial rise followed by a gradual decline, peaking at approximately days 9–12. By contrast, urinary excretion remained consistently low at all time points, indicating that fecal elimination via the hepatobiliary pathway is the dominant clearance route for COF‑Tpy‑Se‑Cu (Supplementary Fig. 12d).

**Fig. 5:**
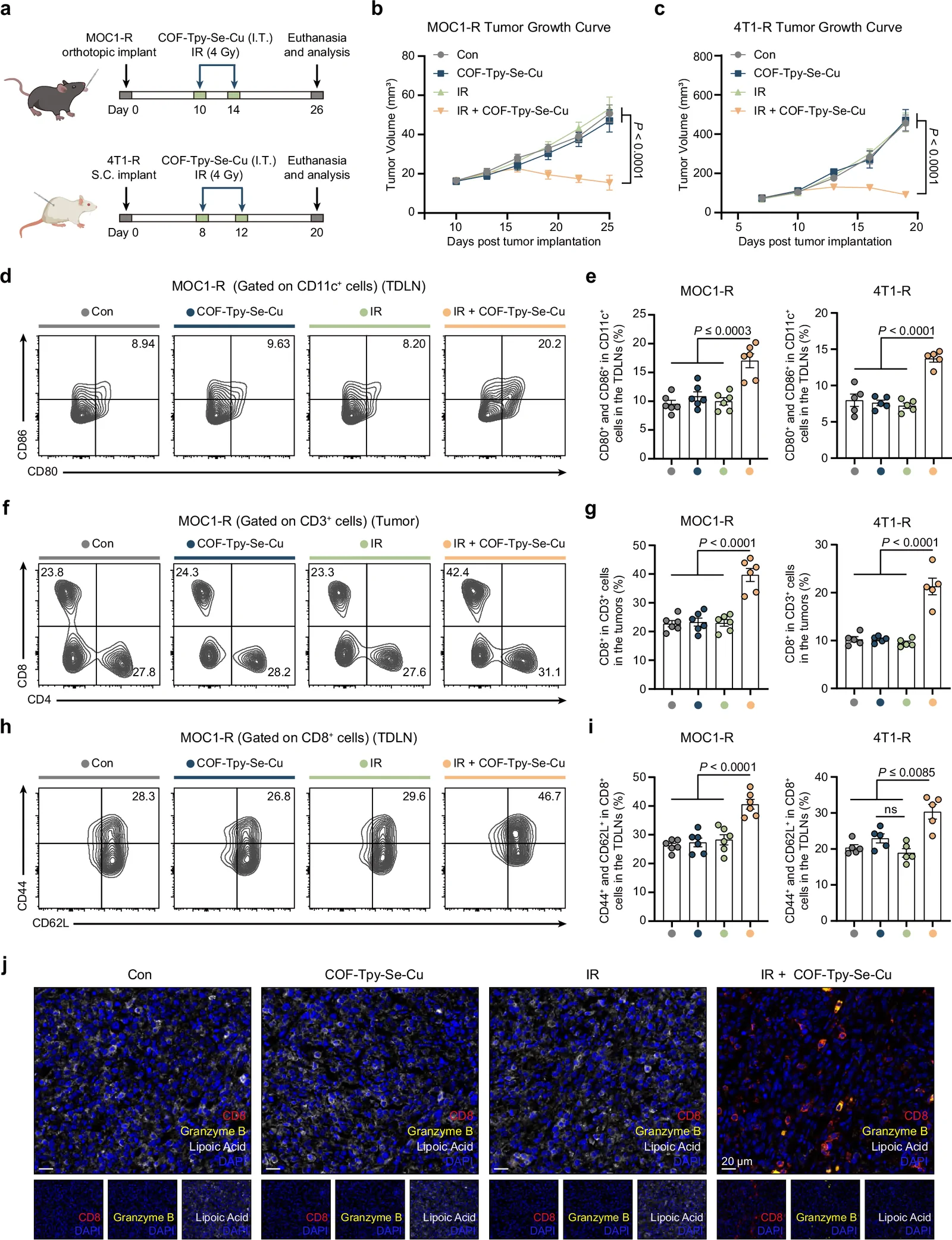
COF‑Tpy‑Se‑Cu-mediated radiosensitization in vivo. **a** Experimental scheme for tumor implantation and treatment in the MOC1‑R (top) and 4T1‑R (bottom) models. Mice received intratumoral (i.t.) COF‑Tpy‑Se‑Cu (2 mg kg^-1^ for MOC1‑R; 5 mg kg^-1^ for 4T1‑R), followed by localized irradiation (IR, 4 Gy). s.c., subcutaneous. Tumor growth curves for MOC1‑R (**b**) and 4T1‑R (**c**) tumors across the indicated treatment groups (MOC1‑R, *n* = 6 mice per group; 4T1‑R, *n* = 5 mice per group). **d**, **e** Flow cytometric analysis of CD80^+^CD86^+^ cells among CD11c^+^ dendritic cells (DCs) in tumor-draining lymph nodes (TDLNs) (MOC1‑R, *n* = 6 mice per group; 4T1‑R, *n* = 5 mice per group). **d** Representative plots showing the frequency of CD80⁺CD86⁺ cells among CD11c^+^ DCs. **e** Quantification of mature DCs in TDLNs from the MOC1‑R (left) and 4T1‑R (right) models. **f**, **g** Flow cytometric analysis of tumor-infiltrating CD8^+^ T and CD4^+^ T cells among CD3^+^ T cells (MOC1‑R, *n* = 6; 4T1‑R, *n* = 5). **f** Representative plots from MOC1‑R tumors. **g** Quantification of CD8^+^ T cell frequency among CD3^+^ T cells in MOC1‑R (left) and 4T1‑R (right) tumors. **h**, **i** Flow cytometric analysis of memory T cell populations among CD8^+^ T cells in TDLNs (MOC1‑R, *n* = 6; 4T1‑R, *n* = 5). **h** Representative plots showing CD44^+^CD62L^+^ T cells. **i** Quantification of memory T cells among CD8^+^ T cells in TDLNs from the MOC1‑R (left) and 4T1‑R (right) models. **j** Multiplex immunohistochemistry (mIHC) staining showing the expression of CD8 (red), granzyme B (yellow), and lipoic acid (white) in tumor sections from the indicated treatment groups (representative of five biologically independent tumor samples per group). Scale bars, 20 μm. Gating strategies are provided in Supplementary Figs. 16–19. Statistical significance was assessed using one-way ANOVA with Tukey’s multiple-comparisons test (**e**, **g**, **i**) or two-way ANOVA (**b**, **c**). Data are presented as mean ± s.e.m. Source data are provided as a Source Data file.

Compared with parental 4T1 tumors, radioresistant 4T1‑R tumors expressed higher levels of FDX1 and COX17, consistent with increased cuproptosis susceptibility (Supplementary Fig. 13a, b). We therefore evaluated the antitumor activity of COF‑Tpy‑Se‑Cu in vivo (Fig. 5a). COF‑Tpy‑Se‑Cu combined with IR suppressed tumor growth significantly more than IR alone or COF‑Tpy‑Se‑Cu monotherapy (Fig. 5b, c and Supplementary Fig. 14a, b). Treatment was well tolerated, with no significant body-weight loss (Supplementary Fig. 14c) and no overt histopathological abnormalities in major organs on hematoxylin and eosin (H&E) staining (Supplementary Fig. 14d, e).

To validate that intratumoral copper elevation is driven by irradiation rather than spontaneous framework leakage, we performed time‑course ICP‑MS analysis of copper levels in tumor (Supplementary Fig. 15a). In the absence of irradiation, intratumoral copper concentrations in the COF‑Tpy‑Se‑Cu monotherapy group were comparable to those in the control and IR‑only groups, confirming negligible spontaneous copper release from the framework in vivo. By contrast, the COF‑Tpy‑Se‑Cu + IR group exhibited a pronounced, time‑dependent increase in intratumoral copper content, which peaked at 24 h post‑irradiation (Supplementary Fig. 15a). We next assessed whether radiation‑triggered copper release achieves functionally effective concentrations to drive cuproptosis in vivo by examining subcellular copper distribution and canonical cuproptosis biochemical hallmarks in tumor lysates. ICP‑MS revealed no significant alterations in total cellular or mitochondrial copper levels following COF‑Tpy‑Se‑Cu or irradiation alone (Supplementary Fig. 15b, c). In contrast, the combination treatment markedly elevated both total tumor copper and mitochondrial copper levels (Supplementary Fig. 15b, c), indicating increased copper accumulation within the mitochondrial compartment—the primary subcellular site of cuproptosis execution. At the protein level, the combination upregulated the proteotoxic stress marker HSP70 and induced DLAT oligomerization, a defining biochemical feature of cuproptosis (Supplementary Fig. 15d). Together, these data demonstrate that radiation‑controlled copper delivery drives sufficient mitochondrial copper overload and elicits cuproptotic proteotoxic stress in vivo.

To define the immunological consequences of COF‑Tpy‑Se‑Cu plus IR treatment, we profiled immune cell populations in tumors and tumor-draining lymph nodes (TDLNs) (gating strategy shown in Supplementary Fig. 16–19). Under irradiation, COF‑Tpy‑Se‑Cu significantly increased the frequency of mature CD80^+^CD86^+^ DCs in TDLNs (Fig. 5d, e and Supplementary Fig. 20a). Within tumors, the combination induced a marked increase in CD8^+^ T‑cell infiltration compared with all other groups (Fig. 5f, g and Supplementary Fig. 20b–d) and increased the proportion of CD44^+^CD62L^+^CD8^+^ central memory T cells (Tcm) in the TDLNs (Fig. 5h, i and Supplementary Fig. 20e). Multiplex immunofluorescence analysis confirmed in situ that combined IR and COF‑Tpy‑Se‑Cu treatment increased CD8^+^ T cell infiltration and granzyme B expression, and reduced lipoic acid levels, consistent with enhanced cytotoxic immunity and cuproptosis induction (Fig. 5j and Supplementary Fig. 21a). Immunohistochemical staining for Ki67, quantified via histoscoring, verified significantly suppressed tumor proliferation in both models (Supplementary Fig. 21b).

To confirm that the in vivo therapeutic effects are mediated by copper‑dependent cuproptosis, we included material‑based and pharmacological control groups. The copper‑free scaffold COF‑Tpy‑Se, either alone or in combination with irradiation, exerted no antitumor activity in either model, ruling out a contribution from the framework itself (Supplementary Fig. 22a–f). Systemic administration of the copper chelator TTM largely abrogated the radiosensitizing efficacy of COF‑Tpy‑Se‑Cu, verifying that bioavailable copper is required for tumor suppression (Supplementary Fig. 23a–f). Genetic ablation of FDX1 further attenuated the tumor‑suppressive effect of the combination regimen in vivo (Supplementary Fig. 24a–d). Gene expression profiling further showed that upregulation of inflammatory chemokines (e.g., *Cxcl9* and *Cxcl10*) and T cell effector genes (e.g., *Gzmb* and *Ifng*) was restricted to the IR + COF‑Tpy‑Se‑Cu group, and was abolished by copper chelation or FDX1 knockout (Supplementary Fig. 25a, b). Collectively, these data demonstrate that COF‑Tpy‑Se‑Cu mediates in vivo radiosensitization via FDX1‑dependent cuproptosis, which drives immunogenic cell death and remodels the tumor microenvironment toward a T‑cell‑inflamed phenotype.

### Oncolytic adenovirus sensitizes tumor cells to cuproptosis via GSH depletion and FDX1 stabilization

Oncolytic virotherapy, exemplified by OVADs, represents a promising anticancer approach, yet their efficacy as monotherapy remains limited (Supplementary Fig. 26)^25,42^. Accumulating evidence indicates that combining oncolytic virotherapy with treatments that induce distinct forms of immunogenic cell death can produce synergistic antitumor effects^24,43^. Notably, the interplay between oncolytic virotherapy and metal-ion-mediated cell death pathways, particularly cuproptosis, has received limited investigation. We therefore examined the interaction between oncolytic virotherapy and cuproptosis, including whether OVAD modulates intracellular copper homeostasis or the cuproptosis machinery.

Before investigating the interaction, we first evaluated whether acquired radioresistance alters OVAD infectivity, replication, and cytolytic activity to exclude potential confounding effects of differential viral sensitivity. Expression of the coxsackievirus and adenovirus receptor (CAR), which mediates viral entry, and telomerase reverse transcriptase (TERT), which regulates tumor‑selective viral replication, did not differ significantly between parental and radioresistant cells at both the cellular protein and tissue levels (Supplementary Fig. 27a–c). TCID_50_ assays performed at a multiplicity of infection (MOI) of 1 confirmed comparable OVAD replication kinetics in MOC1 and MOC1‑R cells (Supplementary Fig. 27d). Consistent with these findings, OVAD elicited equivalent levels of cell death in both cell lines across a range of MOIs (Supplementary Fig. 28a, b). Collectively, these data indicate that acquired radioresistance does not impair OVAD infectivity, replicative capacity, or cytolytic activity.

**Fig. 6:**
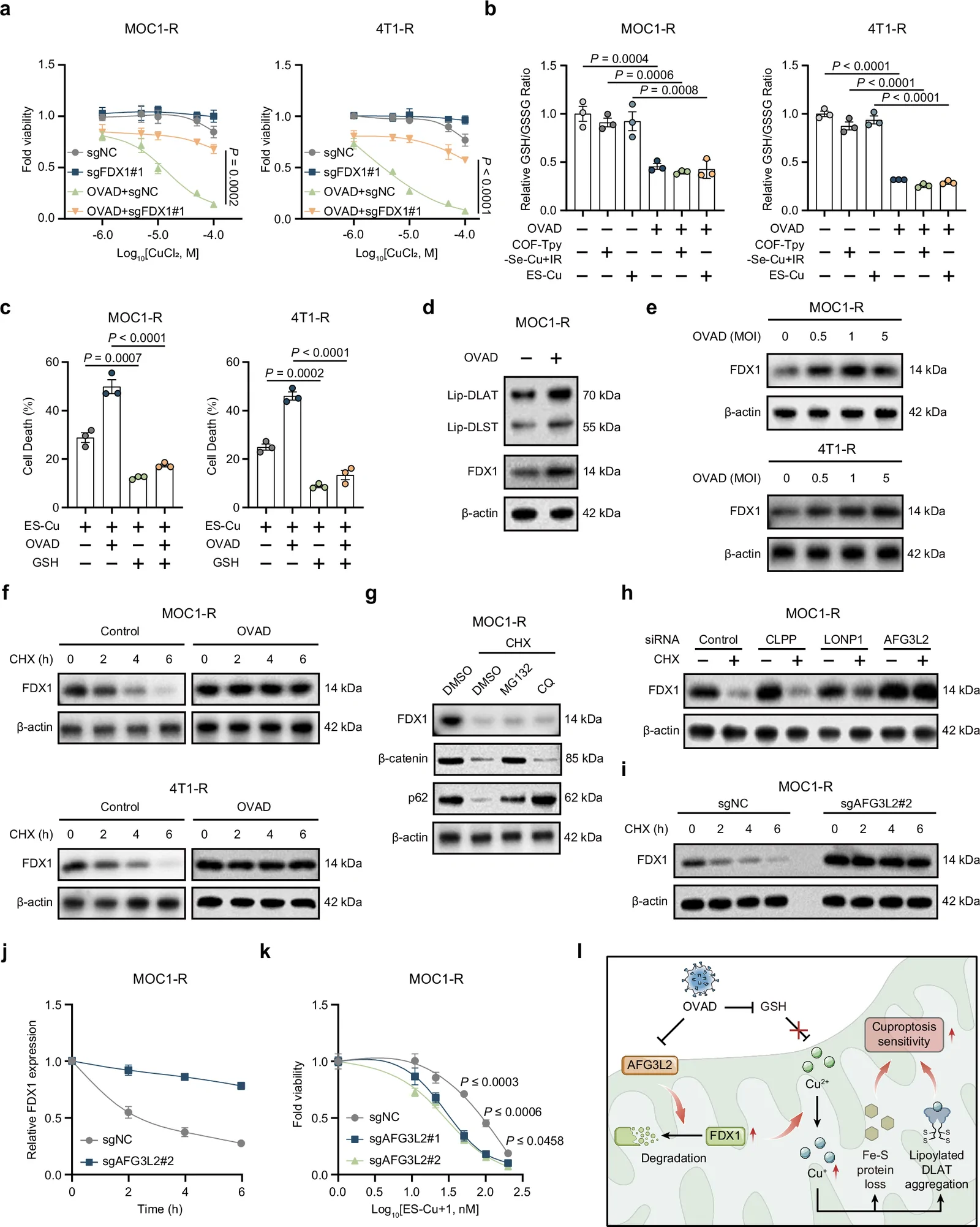
Oncolytic adenovirus sensitizes tumor cells to cuproptosis. **a** Cell viability of MOC1‑R and 4T1‑R cells treated with oncolytic adenovirus (OVAD, MOI = 1) and increasing concentrations of CuCl_2_ for 24 h, measured by CCK‑8 assay (*n* = 3 independent experiments). **b** Intracellular GSH/GSSG ratio in MOC1‑R and 4T1‑R cells after 12 h treatment with elesclomol–Cu (ES-Cu, 200 nM), COF‑Tpy‑Se‑Cu (30 μg mL^-1^) plus irradiation (IR, 4 Gy), or OVAD (MOI = 1) (*n* = 3 independent experiments). **c** Flow cytometric quantification of cell death in MOC1‑R and 4T1‑R cells treated for 24 h with ES-Cu (200 nM), OVAD (MOI = 1), and/or glutathione (GSH, 10 mM) (*n* = 3 independent experiments). **d** Immunoblot analysis of lipoylated DLAT, lipoylated DLST, and FDX1 in MOC1‑R cells after 12 h OVAD treatment (MOI = 1). β‑actin served as a loading control (representative of three independent experiments). **e** FDX1 protein levels following treatment with increasing doses of OVAD (MOI = 0, 0.5, 1, 5) for 12 h (representative of three independent experiments). **f** Cycloheximide (CHX) chase assay assessing FDX1 stability in MOC1‑R and 4T1‑R cells pretreated with OVAD (MOI = 1) and then exposed to CHX (100 μg mL^-1^) for the indicated times (representative of three independent experiments). **g** Immunoblot analysis of FDX1 in MOC1‑R cells treated for 12 h with DMSO, MG132 (10 μM), or chloroquine (CQ, 20 μM). β‑catenin and p62 were included as positive controls (representative of three independent experiments). **h** FDX1 protein levels in MOC1‑R cells 48 h after transfection with siRNAs targeting candidate mitochondrial proteases (representative of three independent experiments). CHX chase analysis of FDX1 degradation in MOC1‑R cells transduced with sgNC or sgAFG3L2: representative immunoblot (**i**) and quantification (**j**) (*n* = 3 independent experiments). **k** Viability of MOC1‑R cells transduced with sgNC or sgAFG3L2 and treated with ES-Cu for 36 h (*n* = 3 independent experiments). **l** Working model: OVAD sensitizes cells to cuproptosis by depleting intracellular GSH and stabilizing FDX1 via inhibition of AFG3L2‑mediated mitochondrial proteolysis. Statistical significance was assessed using one-way ANOVA with Tukey’s multiple-comparisons test (**a**–**c**, **k**). Data are presented as mean ± s.e.m. Source data are provided as a Source Data file.

We next investigated the potential synergistic interaction between OVAD and copper‑induced cytotoxicity. Our results showed that OVAD strongly enhanced copper-induced cell death, and genetic ablation of FDX1 largely blocked this effect (Fig. 6a). These findings prompted further investigation of the underlying mechanism. We next considered whether OVAD alters copper bioavailability or the cuproptosis execution machinery. Because intracellular GSH acts as a physiological copper chelator and has been reported to be depleted by oncolytic viruses, we tested whether GSH depletion mediates OVAD–cuproptosis crosstalk^11,44^. OVAD treatment significantly reduced GSH levels in radioresistant cells (Fig. 6b), whereas exogenous GSH supplementation substantially rescued cell death induced by OVAD plus copper (Fig. 6c and Supplementary Fig. 29a). These findings suggest that OVAD enhances cuproptosis susceptibility, at least in part, by depleting intracellular GSH and thereby increasing the pool of bioavailable cytotoxic copper.

We then examined the effects of OVAD on core regulators of the cuproptosis pathway. OVAD infection increased FDX1 protein abundance and enhanced DLAT lipoylation in radioresistant cells (Fig. 6d). To determine whether the elevated lipoylation signal arose from increased total protein levels or enhanced FDX1‑mediated post‑translational modification, we immunoprecipitated DLAT and immunoblotted with an anti‑lipoic acid antibody. OVAD treatment markedly increased the abundance of lipoylated DLAT in the immunoprecipitates, whereas total DLAT protein levels remained unchanged (Supplementary Fig. 29b). This result indicates that OVAD increases FDX1 protein abundance, which in turn enhances DLAT lipoylation and may thereby modulate cellular sensitivity to cuproptosis. Notably, OVAD treatment did not alter the mRNA levels of cuproptosis‑related genes (Fig. 6d, e and Supplementary Fig. 29c), indicating that FDX1 is regulated at the post‑translational level. Consistently, cycloheximide (CHX) chase assays confirmed that OVAD stabilizes FDX1 protein rather than upregulating its transcription (Fig. 6f). Pharmacological inhibition of the proteasome or the autophagy–lysosome pathway failed to increase FDX1 protein levels (Fig. 6g and Supplementary Fig. 29d), implicating an alternative degradative mechanism.

Given the central role of mitochondrial proteases in matrix protein quality control^45^, we investigated whether they regulate FDX1 turnover. A targeted siRNA screen identified AFG3-like matrix AAA peptidase subunit 2 (AFG3L2) as a principal determinant of FDX1 stability (Fig. 6h and Supplementary Fig. 29e, f). AFG3L2 knockout increased steady-state FDX1 abundance and nearly abolished FDX1 degradation following CHX treatment (Fig. 6i and Supplementary Fig. 29g), indicating that FDX1 is predominantly degraded via an AFG3L2-dependent pathway within mitochondria (Fig. 6j). Functionally, AFG3L2 depletion sensitized tumor cells to cuproptosis (Fig. 6k), underscoring mitochondrial proteostasis as a key regulator of cuproptosis susceptibility in radioresistant cells.

In summary, these data demonstrate that OVAD modulates cuproptosis susceptibility in radioresistant cells through a dual mechanism: depletion of intracellular GSH to relieve copper chelation and stabilization of FDX1 by inhibiting AFG3L2-mediated mitochondrial degradation (Fig. 6l).

### Synergistic antitumor efficacy of COF‑Tpy‑Se‑Cu in combination with oncolytic adenovirus

**Fig. 7:**
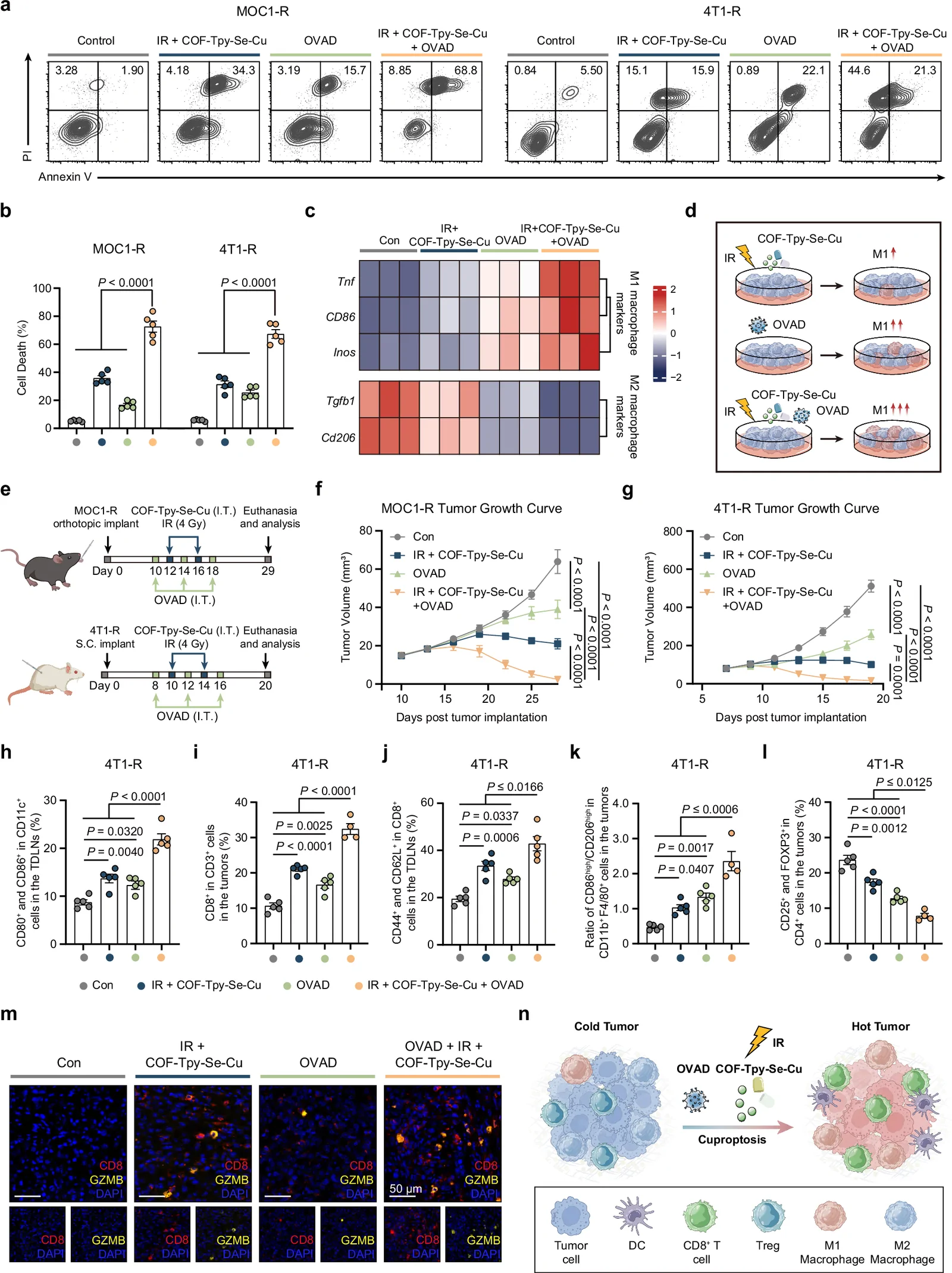
Synergistic antitumor activity of COF‑Tpy‑Se‑Cu combined with oncolytic adenovirus. **a**, **b** Flow-cytometric assessment of cell death 24 h after treatment. **a** Representative Annexin V/PI plots of MOC1‑R (left) and 4T1‑R (right) cells treated with COF‑Tpy‑Se‑Cu (30 μg mL^-1^), irradiation (IR, 4 Gy), and/or oncolytic adenovirus (OVAD, MOI = 1), alone or in combination (*n* = 5 independent experiments). **b** Quantification of cell death, expressed as the percentage of PI^+^ cells, in MOC1‑R (left) and 4T1‑R (right) cells (*n* = 5 independent experiments). **c** Heatmap showing mRNA expression of selected M1-associated (top) and M2-associated (bottom) macrophage signature genes (*n* = 3 independent experiments). **d** Schematic summarizing the effects of COF‑Tpy‑Se‑Cu and OVAD on macrophage polarization. **e** In vivo treatment schedule. MOC1‑R (top) and 4T1‑R (bottom) tumor-bearing mice received intratumoral (i.t.) injections of COF‑Tpy‑Se‑Cu (2 mg kg^-1^ for MOC1‑R; 5 mg kg^-1^ for 4T1‑R) and/or OVAD (0.4 × 10^6^ PFU for MOC1‑R; 2 × 10^6^ PFU for 4T1‑R), followed by local IR (4 Gy) as indicated. s.c., subcutaneous. Tumor growth curves for MOC1‑R (**f**) and 4T1‑R (**g**) models under the indicated treatments (*n* = 5 mice per group). **h**–**l** Flow cytometric profiling of immune populations in the 4T1‑R tumor model (*n* = 5 mice per group; for **i**, **k**, **l**, *n* = 4 tumors were analyzed in the triple-combination group because sufficient tumor tissue was unavailable from one mouse): **h** frequency of CD80^+^CD86^+^ mature dendritic cells (DCs) among CD11c^+^ cells in tumor-draining lymph nodes (TDLNs); **i** quantification of tumor-infiltrating CD8^+^ T among CD3^+^ T cells; **j** proportion of CD44^+^CD62L^+^ memory T cells among CD8^+^ T cells in TDLNs; **k** ratio of CD86^+^ to CD206^+^ macrophages among F4/80^+^ cells within tumors; and **l** frequency of CD25^+^FOXP3^+^ regulatory T cells (Tregs) among CD4^+^ cells in tumor tissues. **m** Multiplex immunohistochemistry (mIHC) staining showing expression of CD8 (red) and granzyme B (yellow) in tumor sections from the indicated treatment groups (representative of three biologically independent tumor samples per group). Scale bars, 50 μm. **n** Working model illustrating how COF-Tpy-Se-Cu and OVAD shift immunologically non-inflamed tumors toward a T-cell-inflamed phenotype. Gating strategies are provided in Supplementary Figs. 16–19. Statistical significance was assessed using one-way ANOVA with Tukey’s multiple-comparisons test (**b**, **h**–**l**) or two-way ANOVA (**f**, **g**). Data are presented as mean ± s.e.m. Source data are provided as a Source Data file.

Given the capacity of OVAD to modulate cellular susceptibility to cuproptosis, we next evaluated the therapeutic efficacy of combining OVAD with COF‑Tpy‑Se‑Cu under irradiation conditions. Relative to irradiation alone, COF‑Tpy‑Se‑Cu significantly increased the proportion of propidium iodide (PI)‑positive cells (Fig. 7a, b). Notably, sequential irradiation followed by OVAD administration resulted in a non‑significant increase in cell death relative to OVAD monotherapy (Supplementary Fig. 30a, b). This observation is consistent with the intrinsic radioresistant phenotype of these cells, whereby ionizing radiation alone exerts limited cytotoxicity and cannot substantially amplify oncolytic virus‑mediated cell killing. In contrast, the triple combination of irradiation, COF‑Tpy‑Se‑Cu, and OVAD induced markedly enhanced cell death, consistent with the synergistic amplification of cuproptosis (Fig. 7a, b and Supplementary Fig. 30a, b).

Clonogenic survival assays further corroborated the radiosensitizing effect of this regimen. The triple combination of OVAD, irradiation, and COF‑Tpy‑Se‑Cu yielded the lowest surviving fraction, indicating sustained suppression of the clonogenic capacity of radioresistant cells (Supplementary Fig. 30c, d). Mechanistically, the triple combination was accompanied by detectable DLAT oligomerization, a canonical biochemical hallmark of cuproptosis (Supplementary Fig. 30e). In a separate experiment performed in the presence of the copper chelator TTM, no apparent DLAT oligomerization was detected under the triple-combination condition (Supplementary Fig. 30f).

To obtain initial insight into immunomodulatory effects, we assessed macrophage polarization. In RAW 264.7 cells polarized toward an M2-like phenotype, OVAD alone repolarized macrophages toward an M1 state, with a stronger effect than COF‑Tpy‑Se‑Cu alone (Fig. 7c, d). Importantly, co-treatment with COF‑Tpy‑Se‑Cu and OVAD further amplified the M2-to-M1 shift, suggesting cooperative remodeling of the tumor immune microenvironment toward a pro-inflammatory phenotype (Fig. 7c, d).

We next examined the intratumoral infectivity and replicative capacity of OVAD in vivo. By flow cytometric analysis gating on the CD45^⁻^ tumor cell population, we observed a time-dependent increase in the frequency of EGFP-positive cells in both MOC1-R and 4T1-R models, indicating that the virus progressively infects more tumor cells and spreads within the tumor parenchyma after intratumoral injection (Supplementary Fig. 31a). In line with this, immunoblot analysis revealed a gradual increase in the viral E1A protein, a marker of active adenoviral replication, supporting ongoing viral replication within tumors (Supplementary Fig. 31b). Meanwhile, we detected elevated cleaved caspase-3 and decreased intracellular HMGB1, indicative of augmented tumor cell death and HMGB1 release, consistent with oncolytic virus-mediated cytolysis (Supplementary Fig. 31b).

We next evaluated the in vivo antitumor performance of the combined therapeutic regimen. Relative to the control group, OVAD monotherapy and COF‑Tpy‑Se‑Cu plus irradiation both achieved significant tumor growth suppression (Fig. 7e–g and Supplementary Fig. 32). In line with the radioresistant phenotype of these models, the addition of single‑modality irradiation to OVAD failed to further improve tumor growth control, resulting in comparable efficacy to OVAD alone (Supplementary Fig. 32). In contrast, the triple combination of COF‑Tpy‑Se‑Cu, OVAD, and irradiation elicited the strongest antitumor effect, with final tumor volumes significantly lower than those of all monotherapy and dual‑combination groups (Fig. 7e–g and Supplementary Fig. 32, 33a, b). The treatment regimen was well tolerated, with no significant body weight loss (Supplementary Fig. 33c), no histopathological abnormalities in major organs (Supplementary Fig. 33d, e), and no notable alterations in complete blood counts or serum biochemical parameters (Supplementary Fig. 34).

To examine whether the improved therapeutic efficacy was associated with enhanced immune activation, we immunophenotyped immune cell populations in tumor tissues and TDLNs. No significant alteration in OVAD infection efficiency was detected following the addition of irradiation plus COF‑Tpy‑Se‑Cu treatment (Supplementary Fig. 35a). In the context of irradiation, COF‑Tpy‑Se‑Cu combined with OVAD significantly increased the proportion of mature DCs in TDLNs (Fig. 7h and Supplementary Fig. 35b). Within tumor tissues, this triple combination elicited the most robust CD8^+^ T cell infiltration (Fig. 7i and Supplementary Fig. 35c–e) and significantly elevated the frequency of CD44^+^CD62L^+^CD8^+^ central memory T cells in TDLNs (Fig. 7j and Supplementary Fig. 35f). The combination also promoted repolarization of tumor-associated macrophages (TAMs) from an M2 to an M1 phenotype (Fig. 7k and Supplementary Fig. 36a) and reduced frequency of regulatory T cells (Tregs) among CD4^+^ T cells within the tumor microenvironment (Fig. 7l and Supplementary Fig. 36b). Multiplex immunofluorescence staining further corroborated enhanced infiltration and activation of cytotoxic T cells in situ (Fig. 7m and Supplementary Fig. 36c). Collectively, increased CD8^+^ T-cell infiltration, strengthened pro-inflammatory signaling, and attenuation of immunosuppressive populations indicate that tumors treated with COF‑Tpy‑Se‑Cu plus irradiation and OVAD acquire features of a T-cell-inflamed phenotype (Fig. 7n), supporting the potential of this combined strategy for treating radioresistant cancers.

### COF‑Tpy‑Se‑Cu combined with oncolytic adenovirus promotes immune memory and inhibits metastasis

**Fig. 8:**
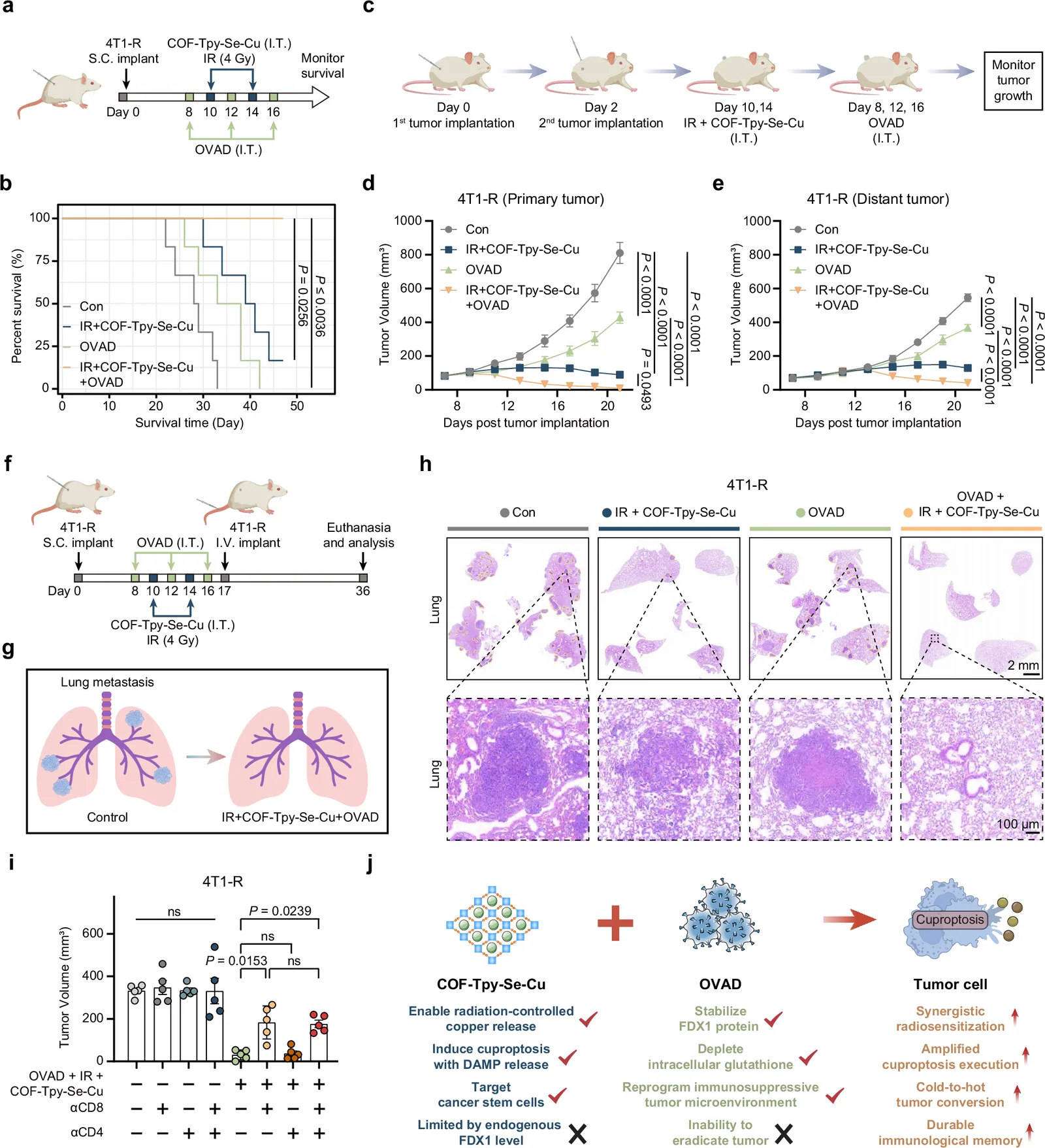
COF‑Tpy‑Se‑Cu combined with oncolytic adenovirus promotes systemic antitumor immunity and inhibits metastasis. **a** Experimental schedule for 4T1‑R tumor implantation and treatment with intratumoral COF‑Tpy‑Se‑Cu (5 mg kg^-1^), irradiation (IR, 4 Gy), and intratumoral OVAD (2 × 10^6^ PFU). s.c., subcutaneous. i.t., intratumoral. **b** Kaplan–Meier survival curves of 4T1‑R tumor–bearing mice across treatment groups (*n* = 6 mice per group). **c**–**e** Evaluation of abscopal activity using a bilateral tumor model in which a secondary contralateral tumor was established (*n* = 5 mice per group). **c** Treatment schedule for the combination regimen. Growth curves of the treated primary tumor (**d**) and the untreated distant tumor (**e**). **f**–**h** Experimental lung colonization model. **f**, **g** Schematic of the experimental design for assessing pulmonary metastatic burden. i.v., intravenous. **h** Representative H&E-stained lung sections showing metastatic nodules across treatment groups (*n* = 5 per group). **i** Tumor volume for the indicated treatment groups (*n* = 5 per group). **j** Schematic overview of the proposed treatment strategy, which synergistically induces cuproptosis, overcomes acquired treatment resistance, reprograms the tumor immune microenvironment, and suppresses metastasis. Statistical significance was assessed using one-way ANOVA with Tukey’s multiple-comparisons test (**i**) or two-way ANOVA (**d**, **e**). Survival (**b**) was analyzed using the log-rank (Mantel–Cox) test. Data are presented as mean ± s.e.m. Source data are provided as a Source Data file.

Given the immunostimulatory effects observed above, we next asked whether the combination therapy establishes durable systemic antitumor immunity. Consistent with this possibility, mice treated with the triple regimen (COF‑Tpy‑Se‑Cu + OVAD + IR) showed significantly prolonged survival compared with animals receiving OVAD alone or COF‑Tpy‑Se‑Cu combined with IR (Fig. 8a, b). We subsequently evaluated the abscopal antitumor activity of the regimen using a bilateral 4T1‑R tumor model, with treatment restricted exclusively to the primary lesion (Fig. 8c). The triple combination of IR, COF‑Tpy‑Se‑Cu, and OVAD significantly suppressed the growth of both the treated primary tumors and the untreated contralateral distant tumors, outperforming both OVAD monotherapy and the IR + COF‑Tpy‑Se‑Cu dual regimen (Fig. 8d, e and Supplementary Fig. 37a, b). Immunophenotyping of contralateral tumor‑draining lymph nodes showed enhanced DC maturation and expanded memory CD8^+^ T cell populations, paired with increased CD8^+^ T cell infiltration in distal tumors (Supplementary Fig. 37c–j). Collectively, these data demonstrate that the combination therapy elicits robust systemic antitumor immunity capable of controlling distant tumor foci. Furthermore, in an experimental lung colonization model, the triple combination regimen significantly attenuated pulmonary metastatic burden compared with the untreated control, and exhibited the most potent antimetastatic activity across all tested treatment groups (Fig. 8f–h and Supplementary Fig. 37k).

To delineate the respective contributions of CD8^+^ and CD4^+^ T cells to the observed therapeutic efficacy, we performed in vivo antibody‑mediated cell depletion during treatment (Fig. 8i and Supplementary Fig. 38a–f). CD8^+^ T cell depletion significantly attenuated the antitumor effect of the triple regimen. By contrast, CD4^+^ T cell depletion alone did not significantly alter treatment efficacy (Fig. 8i and Supplementary Fig. 38a–f). Together, these results indicate that COF‑Tpy‑Se‑Cu, OVAD, and irradiation act synergistically to improve local tumor control and stimulate CD8^+^ T cell–dependent systemic immunity. This response promotes durable immune memory and suppresses metastasis. The observed synergy (Fig. 8j) likely arises from the complementary mechanisms that overcome limitations of individual treatments and improve therapeutic outcomes.

## Discussion

Radiotherapy is a standard treatment for cancer, but its effectiveness is often limited by acquired resistance^1,5^. Building on prior observations linking cuproptosis to cellular radiation response, in this study, we confirm heightened cuproptosis sensitivity in radioresistant cancer cells and identify the stress‑responsive transcription factor ATF3 as a key upstream regulator that directly transactivates the core cuproptosis effector genes *Fdx1* and *Cox17* to confer this phenotype. To exploit this vulnerability, we developed a radiosensitization strategy that combines a radiation-responsive, copper-incorporated covalent organic framework (COF-Tpy-Se-Cu) with OVAD, whose components converge on the core cuproptosis effector FDX1 to synergistically induce cuproptosis. Our approach combines controlled copper delivery, viral immunotherapy, and radiotherapy to overcome acquired radioresistance, enhance tumor immunogenicity, and elicit robust systemic antitumor immunity.

Recent evidence links radiotherapy with cuproptosis, a copper-dependent, mitochondria-driven form of cell death^9,10^. Consistent with prior phenotypic reports, we confirm that radioresistant cells, despite surviving irradiation, show increased sensitivity to cuproptosis. This susceptibility is mechanistically driven by the transcription factor ATF3, which is upregulated in radioresistant cells and directly activates the promoters of two key cuproptosis mediators: FDX1, which reduces Cu^2+^ to the more cytotoxic Cu^+^, and COX17, which facilitates mitochondrial copper import. Their coordinated upregulation leads to mitochondrial copper overload, thereby priming the cells for cuproptosis. This creates a therapeutic window where cells surviving irradiation become vulnerable to a distinct form of metabolic cell death.

To therapeutically target this defined vulnerability, we rationally designed COF‑Tpy‑Se‑Cu with material principles tailored to the shared biological features of radioresistant tumors and the clinical practice of local fractionated radiotherapy across multiple cancer types^3–6,46^. Its covalently crosslinked scaffold incorporates radiation‑cleavable diselenide (–Se–Se–) bonds^37,38,47^, which undergo selective cleavage under X‑ray irradiation to trigger localized copper release within tumor tissue. This radiation‑responsive release behavior is engineered to align with clinical fractionated radiotherapy regimens, with the goal of achieving on‑demand copper delivery coupled to each radiation session. Additionally, the robust covalent framework confers high structural stability under pathophysiologically relevant tumor microenvironmental conditions, which is expected to support prolonged intratumoral retention and responsiveness to repeated irradiation. Collectively, this design minimizes systemic copper exposure and selectively exploits the intrinsic cuproptosis vulnerability of radioresistant cancer cells with upregulated FDX1 and COX17 expression, enabling preferential elimination of treatment‑resistant tumor subpopulations. COF‑Tpy‑Se‑Cu acts as a radiosensitizer by triggering FDX1‑dependent cuproptosis and enhancing tumor immunogenicity, as evidenced by elevated release of DAMPs such as HMGB1, ATP, and CRT.

The cuproptosis induced by COF‑Tpy‑Se‑Cu depends on endogenous FDX1 expression within tumors. Notably, FDX1 expression decreases during cuproptosis, which may restrict the sustained efficacy of cuproptosis‑based therapies^11,19,20^. Furthermore, the effectiveness of cuproptosis‑inducing nanomedicine can be constrained by insufficient tumor penetration and a limited capacity to remodel the immunosuppressive tumor microenvironment^14,17,21^. To address these constraints, we established a dual‑modality combinatorial strategy centered on the core cuproptosis effector FDX1, pairing COF‑Tpy‑Se‑Cu with OVAD. OVAD stabilizes FDX1 by inhibiting its degradation by mitochondrial proteases, thereby addressing a central constraint of cuproptosis-based approaches that rely on intrinsic FDX1 levels. The two modalities converge at the FDX1 node to exert synergistic cuproptotic effects that exceed simple additive activity. This transcription‑independent stabilization reveals a link between viral infection and the regulation of mitochondrial protein homeostasis. Moreover, we found that OVAD, more effectively than COF‑Tpy‑Se‑Cu alone, redirects macrophages toward an antitumor phenotype and reduces Treg abundance, thereby actively reshaping the immunosuppressive microenvironment. Conversely, COF‑Tpy‑Se‑Cu exhibits superior potency in directly inducing cuproptosis, killing tumor cells, and targeting resistant cell populations, thus overcoming the inherent limitation of oncolytic viruses in eradicating tumors.

Notably, a recent study provided important mechanistic insights into the reciprocal interplay between cuproptosis and antitumor immunity, demonstrating the therapeutic potential of exploiting this crosstalk to overcome immunotherapy resistance^48^. Consistent with these findings, our results show that cuproptosis induction is associated with features of immunogenic cell death and enhanced antitumor immune responses, further supporting the close connection between cuproptosis and tumor immunity. Specifically, OVAD and COF‑Tpy‑Se‑Cu significantly increased tumor cell cuproptosis and reshaped the tumor immune environment (Fig. 8j). This combination turned immunologically cold tumors into hot tumors, characterized by increased infiltration and activation of CD8⁺ T cells, enhanced DC maturation in draining lymph nodes, expansion of memory T cell populations, repolarization of TAMs from an M2 to an M1 phenotype, and a pronounced reduction in Tregs. Depletion of CD8^+^ T cells significantly attenuated the therapeutic efficacy, confirming CD8^+^ effector T cells as the central mediators of the immune-driven antitumor response. Notable residual tumor growth inhibition was retained after CD8^+^ T cell depletion, which we attribute to the direct, T cell–independent cytotoxicity of radiation-triggered copper release from COF‑Tpy‑Se‑Cu, which induces FDX1-mediated cuproptosis in tumor cells irrespective of adaptive immunity. In contrast, pan‑CD4^+^ T cell depletion alone did not significantly alter the overall antitumor effect, and combined depletion of CD4^+^ and CD8^+^ T cells yielded outcomes comparable to CD8^+^ T cell depletion alone. This lack of net effect upon bulk CD4 depletion reflects the functional heterogeneity of CD4^+^ T cell subsets in the tumor microenvironment. As also evidenced by our observation of reduced intratumoral Treg frequency, pan-CD4 depletion simultaneously eliminates two functionally opposing populations: conventional type 1 helper T (Th1) cells that support CD8^+^ T cell priming and clonal expansion, and immunosuppressive CD4^+^CD25^+^FOXP3^+^ regulatory T cells that constrain antitumor immunity. These two countervailing effects offset each other, resulting in no significant net change in short-term tumor growth control. Additionally, the robust release of DAMPs and strong DC maturation induced by the combination regimen provide sufficient co-stimulatory signals to drive CD8^+^ T cell activation. It is worth noting that although CD4^+^ T cell depletion did not significantly impair acute tumor growth control in our model, CD4^+^ T cells are well recognized to support the long-term maintenance of CD8^+^ T cell memory. This established function is consistent with our observation of expanded memory T cell populations and prolonged antitumor protection in the combination regimen. Systemic antitumor immunity was further demonstrated by an abscopal effect—manifested by suppression of distant, non-irradiated tumors—and a significant decrease in lung metastatic burden. Together, these results indicate that the combination regimen elicits a potent, T‑cell‑dependent antitumor response capable of suppressing metastasis and establishing durable immunological memory, thereby addressing the challenge of acquired radioresistance.

In summary, we present an integrated strategy merging radiochemistry, nanomedicine, and oncolytic virotherapy. By targeting the increased cuproptosis sensitivity of radioresistant cells with a radiation-responsive material and amplifying cell death with oncolytic virus, we show a powerful approach to overcome acquired radioresistance. Beyond revealing mechanisms of copper metabolism in cancer therapy, this work provides a flexible combined platform with potential to improve outcomes in radiotherapy.

## Methods

### Ethical statements

Ethical approval for this study was obtained from the Institutional Animal Care and Use Committee of Zhengzhou University (approval number: ZZU-LAC20251212[28]). All animal procedures complied with the national guidelines for laboratory animal care and use issued by the State Council of the People’s Republic of China. Animals were housed in a specific pathogen-free facility under a 12-h light/12-h dark cycle at approximately 22 °C and 50% relative humidity.

### Construction of COF‑Tpy‑Se‑Cu

COF-Tpy-Se-Cu was synthesized by combining an aldehyde-functionalized pyridine-based ligand (L-Tpy), a diselenide linker (L-Se), and copper(II) acetate [Cu(OAc)_2_] in a Pyrex tube. To this precursor mixture, 1.5 mL of mesitylene, 1.5 mL of dimethylacetamide, and 0.6 mL of 6 M aqueous acetic acid were added. The Pyrex tube was subsequently sealed and subjected to heating at 120 °C for 72 h. Following the reaction, the resulting COF sample was centrifuged and washed with a chloroform/acetone solvent mixture. The final product was then dried at 120 °C for 24 h. COF‑Tpy‑Se‑Cu and other materials generated in this study are available from the corresponding authors upon reasonable request.

### Cells and reagents

The mouse breast cancer cell line 4T1 (ATCC, CRL‑2539) was cultured in RPMI‑1640 (Gibco, Thermo Fisher Scientific, NY, USA) supplemented with 10% fetal bovine serum (FBS, Gibco). MOC1 murine oral carcinoma cells (Kerafast, EWL001‑FP, MA, USA) were maintained in a 2:1 mixture of Iscove’s modified Dulbecco’s medium (IMDM) and Ham’s F‑12 nutrient mixture (Gibco), supplemented with 5% FBS, epidermal growth factor (EGF, 5 ng mL^-1^), hydrocortisone (40 ng mL^-1^), and insulin (5 μg mL^-1^). The mouse esophageal squamous cell carcinoma cell line AKR (Otwo Biotech, HTX2545, Shenzhen, China) was cultured in Dulbecco’s modified Eagle medium (DMEM, Corning, NY, USA) containing 10% FBS. The mouse colon adenocarcinoma cell line MC38 (Kerafast, ENH204‑FP) was cultured in DMEM supplemented with 10% FBS. RAW 264.7 macrophages (ATCC, TIB‑71) and human embryonic kidney 293 T cells (ATCC, CRL‑3216) were cultured in DMEM supplemented with 10% FBS. Radioresistant cell lines were generated from their respective parental cells by repeated irradiation at 2 Gy per fraction, with recovery and passaging between treatments. The radioresistant phenotype was confirmed using clonogenic survival assays. All cell lines were maintained at 37 °C in a humidified incubator with 5% CO_2_.

Small-molecule reagents were purchased from MedChemExpress (MCE, NJ, USA), including elesclomol (HY‑12040), tetrathiomolybdate (TTM, HY‑128530), ferrostatin‑1 (Fer‑1, HY‑100579), Z‑VAD‑FMK (HY‑16658B), N‑acetylcysteine (NAC, HY‑B0215), necrostatin‑1 (Nec‑1, HY‑15760), cycloheximide (CHX, HY‑12320), MG132 (HY‑13259), and chloroquine (CQ, HY‑17589 A).

### Oncolytic adenovirus

The oncolytic adenovirus OVAD‑hTERT‑E1A‑IRES‑E1B‑SV40‑EGFP‑RGD (catalog no. OVAD184‑2282; FuBio Biotechnology, Suzhou, China) was used in this study^49,50^, with its vector map provided in Supplementary Fig. 26. This serotype 5 adenovirus features E1A/E1B expression driven by the human telomerase reverse transcriptase (hTERT) promoter for tumor‑selective replication, a complete E3 region deletion, and an RGD‑modified fiber knob mediating viral entry. An EGFP reporter was incorporated for infection tracking.

Virus was amplified in HEK293 cells. Cells were incubated with viral inoculum and cultured until widespread cytopathic effect appeared. Harvested cells were subjected to three freeze‑thaw cycles, and the resulting lysate was concentrated by PEG 8000 precipitation and purified via double CsCl density gradient ultracentrifugation. Viral stocks were titrated by plaque-forming assay and stored at −80 °C until use. For replication kinetics, cells were seeded 24 h before infection at an MOI of 1. After 2 h of incubation, the inoculum was replaced with fresh medium, and viral titers were measured at serial time points using the TCID_50_ assay.

### Animal experiments

Female BALB/c and C57BL/6 mice (6–8 weeks old) were obtained from GemPharmatech (Nanjing, China). The study was designed to evaluate therapeutic efficacy rather than sex-dependent responses. No sex-based comparisons were performed. Mice were housed under specific pathogen-free conditions. For tumor establishment, 1 × 10^6^ 4T1‑R cells were injected subcutaneously into the right inguinal region of BALB/c mice, and 1 × 10^5^ MOC1‑R cells were implanted orthotopically into the dorsal tongue of C57BL/6 mice. When tumor volumes reached ~100 mm^3^ (4T1‑R) or ~20 mm^3^ (MOC1‑R), mice received intratumoral COF‑Tpy‑Se‑Cu (4T1‑R: 5 mg kg^−1^; MOC1‑R: 2 mg kg^−1^) or OVAD (4T1‑R: 2 × 10^6^ PFU; MOC1‑R: 0.4 × 10^6^ PFU). To verify the copper‑dependent mechanism of therapeutic efficacy, mice received intraperitoneal administration of TTM (10 mg kg^−1^). Tumor size and body weight were monitored throughout the study, and mice were euthanized at the endpoint for tissue collection. The maximal tumor volumes permitted under the approved animal study protocol were 1500 mm^3^ for subcutaneous 4T1‑R tumors and 150 mm^3^ for orthotopic MOC1‑R tongue tumors. Mice were euthanized when tumor volumes reached these predefined humane endpoints or earlier if tumor burden otherwise compromised animal welfare. These maximal permitted tumor volumes were not exceeded in any experiment.

To assess the role of adaptive immunity in antitumor responses induced by the COF‑Tpy‑Se‑Cu + IR + OVAD combination regimen, a separate cohort of 4T1‑R tumor–bearing mice received intraperitoneal injections of anti‑CD8α antibody (Bio X Cell, BE0061, NH, USA, 10 mg kg^−1^), anti‑CD4 antibody (Bio X Cell, BP0003-1, 10 mg kg^−1^), or IgG2b isotype control antibody (Bio X Cell, BP0090, 10 mg kg^−1^).

For immune memory and abscopal effect studies, mice were inoculated with 1 × 10^6^ 4T1‑R cells in the right flank on day 0, followed by secondary contralateral inoculation with an equal number of tumor cells in the contralateral flank 2 days later. In this bilateral tumor model, only the primary lesion received the designated treatment, enabling concurrent assessment of treated primary and untreated distant tumors within the same animal.

To evaluate the lung colonization capacity of tumor cells, an experimental lung colonization model was established. BALB/c mice bearing established subcutaneous 4T1‑R tumors were treated with COF‑Tpy‑Se‑Cu and OVAD, followed by intravenous injection of 5 × 10^5^ 4T1‑R cells via the tail vein to generate pulmonary metastatic foci. Lungs were harvested on day 36 for histopathological assessment and quantitative analysis of metastatic nodules. The general humane endpoints described above also applied to the experimental lung colonization model. Mice were euthanized before the scheduled endpoint if the tumor burden compromised animal welfare, as indicated by persistent respiratory distress, markedly reduced activity, or a loss of more than 20% of their initial body weight. The maximal permitted tumor size was not exceeded.

### Cell viability and colony formation assays

Cell viability was measured using the CCK-8 reagent (Beyotime Biotechnology, Shanghai, China)^31^. Cells were plated in 96‑well plates, treated as indicated, and then incubated with the CCK‑8 working solution. Absorbance at 450 nm was recorded using a microplate reader, and viability was normalized to untreated controls. For colony formation assays, cells were plated into 6-well plates and cultured to allow colony growth. Colonies were fixed with methanol, stained with 0.1% crystal violet, and thoroughly washed. Colonies were quantified using ImageJ software.

### Measurement of glutathione (GSH) and glutathione disulfide (GSSG) levels

Intracellular reduced GSH and oxidized GSSG levels in tumor cells were quantified using a GSH/GSSG assay kit (Beyotime Biotechnology)^13^. Briefly, harvested cells were lysed in the protein-removal reagent M and centrifuged to remove precipitated proteins. The clarified supernatant was used for subsequent measurements. To selectively quantify GSSG, an aliquot of each supernatant was pretreated with the GSH-scavenging reagent to deplete reduced glutathione. Samples were then combined with the total glutathione detection working solution in 96-well plates, followed by the addition of NADPH, and absorbance at 412 nm was recorded kinetically. A standard curve was generated using GSSG standards processed in parallel. Total glutathione and GSSG concentrations were calculated from the standard curve.

### Immunogenic cell death detection

Immunogenic cell death was evaluated by assessing CRT exposure, HMGB1 release, extracellular ATP secretion, LDH release, and intracellular HSP70 upregulation. For CRT and HMGB1 staining, MOC1‑R or 4T1‑R cells seeded in confocal dishes were treated with PBS or COF‑Tpy‑Se‑Cu (30 μg mL^−1^) and irradiation (0 or 4 Gy). Cells were fixed with 4% paraformaldehyde, permeabilized with 0.2% Triton X‑100 (Sigma‑Aldrich, MO, USA), blocked with 5% BSA (Sigma‑Aldrich), and incubated overnight with anti‑CRT (CST, #12238, 1:400) and anti‑HMGB1 (Abcam, ab18256, 1:400). After washing, DyLight 488–conjugated anti‑rabbit secondary antibody (Abbkine, #23220, 1:500) was applied, and nuclei were counterstained with DAPI (Beyotime Biotechnology) before imaging.

Intracellular HSP70 was quantified by flow cytometry after staining with anti‑HSP70 (Abcam, ab2787, 1:200) followed by DyLight 488–conjugated anti‑mouse secondary antibody (Abcam, ab96879, 1:500). Mouse IgG1 isotype control (Abcam, ab91353, 1:200) was used to define background, and at least 10,000 events were acquired.

Extracellular ATP was quantified from supernatants of cells plated in 24‑well plates (1 × 10^5^ cells per well) using an ATP assay kit (Beyotime Biotechnology) 24 h after treatment. LDH release was measured using an LDH assay kit (Beyotime Biotechnology) in cells seeded in 96‑well plates (5 × 10^3^ cells per well) and serum-starved for 12 h before treatment. After 24 h, supernatants were centrifuged, mixed with the LDH working solution, incubated at 37 °C for 30 min, and read at 490 nm.

### Mitochondrial isolation

Mitochondria were isolated from cultured tumor cells with a Mitochondria Isolation Kit (Thermo Fisher Scientific) following the manufacturer’s protocol. In brief, cell pellets or homogenized tumor tissues were resuspended in Reagent A supplemented with protease inhibitors. Reagent B was added to permeabilize the plasma membrane, followed by the addition of Reagent C. Nuclei and cellular debris were removed by differential centrifugation to obtain a crude mitochondrial fraction. An additional centrifugation step was performed to reduce lysosomal and peroxisomal contamination and to enrich the heavy mitochondrial fraction. The final mitochondrial pellet was washed, resuspended, and maintained on ice for analyses.

### Inductively coupled plasma mass spectrometry (ICP‑MS)

Intracellular copper content from cultured cells and tissue samples was quantified by ICP‑MS^13^. Cell pellets or homogenized tumor tissues were fully lysed, and soluble fractions were collected via ultrafiltration prior to digestion. Samples were digested in high‑purity nitric acid, and copper concentrations were measured on an Agilent 7800 ICP‑MS system (Agilent Technologies, CA, USA), with technical support from Shiyanjia Lab (Hangzhou, China, www.shiyanjia.com). Copper levels were normalized to corresponding protein content or tissue weight.

### Immunofluorescence imaging for copper detection

Cells were seeded in confocal dishes (5 × 10^3^ cells per dish) and allowed to adhere. Mitochondria were labeled with MitoTracker Green FM (200 nM, Thermo Fisher Scientific) at 37 °C for 30 min in the dark. Cells were then incubated with the copper-sensitive probe CopperSensor‑1 (5 μM, MCE) and Hoechst 33342 (Thermo Fisher Scientific) for 15 min under light-protected conditions. After washing with PBS, cells were imaged immediately in serum-free medium. Colocalization between the MitoTracker (mitochondria) and CopperSensor‑1 (copper) channels was quantified to assess mitochondrial copper distribution.

### Biodistribution and excretion study of COF-Tpy-Se-Cu

Female C57BL/6 mice bearing MOC1-R orthotopic tongue tumors were employed for in vivo biodistribution and excretion assays. For ex vivo fluorescence imaging, Cy5.5-loaded COF-Tpy-Se-Cu (2 mg kg^−1^ per mouse) was administered via intratumoral injection. At 7 days post-injection, mice were euthanized, and major organs (heart, liver, spleen, lung, kidney) along with tumor tissues were excised and imaged using an in vivo optical imaging system. The average radiant efficiency of each tissue was quantified for semi-quantitative evaluation of material distribution. For quantitative time-course biodistribution analysis, unlabeled native COF-Tpy-Se-Cu (2 mg kg^−1^ per mouse) was intratumorally injected. Mice were sacrificed at predetermined time points (1, 3, 7, 14, and 30 days post-administration). Tumors and major organs were collected, accurately weighed, and digested via microwave-assisted acid digestion. Selenium, covalently integrated into the COF framework as an intrinsic elemental tracer, was quantified by ICP-MS. All quantitative results were corrected by subtracting the average endogenous selenium background from untreated controls. Material accumulation in each tissue was normalized to tissue weight and expressed as percentage of injected dose per gram of tissue (% ID/g).

For excretion profiling, tumor-bearing mice were individually housed in metabolic cages immediately after injection. Urine and fecal samples were collected at serial intervals over a 30-day monitoring period. Feces were lyophilized, ground into fine powder, and digested following the same acid digestion protocol. Urine samples were diluted with dilute nitric acid prior to ICP-MS measurement. The baseline selenium levels in feces and urine were also determined using samples from the same untreated control cohort, and all excretion data were corrected by subtracting the corresponding endogenous selenium background. Daily fecal and urinary excretion rates were calculated and expressed as the percentage of injected dose per day.

### Flow cytometry

Single-cell suspensions were prepared from excised tumors and tumor-draining lymph nodes using a gentleMACS Dissociator (Miltenyi Biotec, Bergisch Gladbach, Germany)^37^. For immunophenotyping, cells were stained with fluorophore-conjugated antibodies according to the manufacturer’s instructions. The antibody panel included: Fixable Viability Dye eFluor^TM^ 506 (eBioscience, 1:1000); anti‑CD45‑APC‑Cy7 (1:500), anti‑CD4‑eFluor 450 (1:500), anti‑CD8‑PerCP (1:500), anti‑CD44‑PE (1:500), anti‑CD62L‑APC (1:200), anti‑CD25‑APC (1:200), anti‑FOXP3‑PE (1:100), anti‑CD80‑PE (1:300), anti‑CD86‑APC (1:300), and anti‑F4/80‑PE‑Cy7 (1:200) from eBioscience; and anti‑CD3‑PE-Cy7 (1:500), anti‑CD11b‑PerCP (1:500), anti‑CD11c‑PerCP (1:500), anti‑CD86‑PE (1:200), and anti‑CD206‑APC (1:200) from BioLegend. The boundaries of all positive gates were determined by fluorescence-minus-one (FMO) controls for each fluorochrome, to objectively distinguish specific signals from background autofluorescence and non-specific staining. Gating strategies are provided in Supplementary Figs. 16–19.

Intratumoral OVAD infection efficiency was quantified by flow cytometry as the percentage of EGFP^+^ cells within the CD45^-^ tumor cell population, leveraging the EGFP reporter cassette encoded in the viral genome. Cell death was assessed using an Annexin V‑APC/PI apoptosis detection kit (Elabscience, Wuhan, China) according to the manufacturer’s protocol, and PI-positive cells were enumerated as the total dead cell population. ALDH activity was measured using the ALDEFLUOR assay kit (STEMCELL Technologies, BC, Canada), with the specific ALDH inhibitor diethylaminobenzaldehyde (DEAB) used as a negative control. Coxsackievirus and adenovirus receptor (CAR) expression was quantified by flow cytometry after staining with anti-CAR antibody (Abcam, ab214354, 1:100) followed by DyLight 488–conjugated anti‑rabbit secondary antibody (Abcam, ab96883, 1:200). IgG isotype control (Abcam, ab37415, 1:100) was used to define background, and at least 10,000 events were acquired. Flow-cytometry data were analyzed and visualized using FlowJo (v10.0.6).

### Quantitative real-time polymerase chain reaction (qRT‒PCR)

Expression of inflammatory and cuproptosis-related genes in tumor tissues or cells was quantified by qRT–PCR^37^. Tissues were homogenized under cryogenic conditions, and total RNA was extracted from tissue homogenates or cultured cells using an RNA isolation kit (Axygen, CA, USA) following the manufacturer’s instructions. RNA concentration was determined using a UV–Vis spectrophotometer (UV‑2401PC, Kyoto, Japan). First-strand cDNA was synthesized from 1 μg total RNA using HiScript II Reverse Transcriptase (Vazyme Biotech, Nanjing, China). qPCR was performed using SYBR Premix (Vazyme Biotech) with ~1% of the cDNA reaction as template. Relative expression was calculated using the 2^⁻ΔΔCT^ method with β‑actin as the reference gene. Primer sequences are listed in Supplementary Table 1. Heatmaps were generated in RStudio.

### Western blot and immunoprecipitation assay

Protein concentrations were quantified using a BCA Protein Assay Kit (Beyotime Biotechnology) according to the manufacturer’s instructions. Proteins were resolved by SDS–PAGE and transferred to PVDF membranes (MilliporeSigma, Burlington, MA, USA). Membranes were blocked with antibody blocking buffer (Beyotime Biotechnology) and incubated with primary antibodies overnight. After washing, membranes were incubated with horseradish peroxidase (HRP)–conjugated secondary antibodies. Immunoreactive bands were developed using an enhanced chemiluminescence (ECL) substrate kit (Advansta, San Jose, CA, USA) and imaged on an iBright FL1500 system (Thermo Fisher Scientific, Waltham, MA, USA). Band intensities were quantified using ImageJ. For DLAT oligomerization assay, protein samples were prepared in LDS sample buffer without DTT or TCEP and resolved via non‑reducing SDS–PAGE, with all subsequent immunoblotting steps performed following the general protocol described above.

For immunoprecipitation, cells were lysed in IP lysis buffer (Invitrogen) supplemented with a protease inhibitor cocktail. Clarified cell lysates were incubated with anti‑DLAT antibody (Abmart, T58125, 1:50) or rabbit IgG (Beyotime, A7016) overnight at 4 °C, followed by capture with Protein A/G Magnetic Beads (MCE, HY‑K0202). Precipitated protein complexes were washed, eluted by boiling in SDS–PAGE loading buffer, and analyzed by immunoblotting with an anti‑lipoic acid antibody (Abcam, ab58724).

Primary antibodies included anti-adenovirus type 5 E1A (Abcam, ab204123, 1:200), anti‑AFG3L2 (Abcam, ab68023, 1:2000), anti‑ATF3 (Abcam, ab207434, 1:1000), anti‑HMGB1 (Abcam, ab18256, 1:1000), anti‑HSP70 (Abcam, ab2787, 1:2000), anti‑lipoic acid (Abcam, ab58724, 1:1000), anti-CAR (Abmart, PK30578, 1:500), anti-cleaved caspase 3 (Abmart, TA7022, 1:2000), anti‑COX17 (Abmart, MG935574, 1:2000), anti‑DLAT (Abmart, T58125, 1:1000), anti‑FDX1 (Abmart, T510671, 1:1000), anti-TERT (Abmart, TD7129, 1:1000), anti‑p62 (CST, #5114, 1:1000), anti‑β‑catenin (CST, #8480, 1:1000), and anti‑β‑actin (CST, #4970, 1:1000). Source data are provided in the Source Data file.

### Immunohistochemistry (IHC) and histological analysis

Paraffin-embedded tumor tissues and major organs were sectioned (4 μm), deparaffinized, rehydrated, and stained with H&E using a commercial kit (Sparkjade, Jinan, China) according to the manufacturer’s instructions^51^. For immunohistochemistry, tissue sections were subjected to heat‑induced antigen retrieval under pressure in citrate buffer. Endogenous peroxidase activity was blocked with 3% hydrogen peroxide, followed by serum blocking. The sections were then incubated overnight at 4 °C with the following primary antibodies: anti‑Ki67 (CST, #12202, 1:500), anti-CAR (Abmart, PK30578, 1:200), anti‑COX17 (Abmart, MG935574, 1:200), anti‑FDX1 (Abmart, T510671, 1:200), and anti-TERT (Abmart, TD7129, 1:500). After washing, the sections were treated with ready‑to‑use HRP‑conjugated anti‑rabbit or anti‑mouse secondary antibodies (CST, #8114/#8125) and visualized using a DAB substrate kit (CST). Slides were digitized using an Aperio ScanScope CS scanner (Leica Biosystems, Wetzlar, Germany). Images were captured with CaseViewer (v2.4, 3DHISTECH), and histoscores were quantified using Aperio ImageScope (v12.3.2, Leica Biosystems).

### Multiplexed immunohistochemistry

Multiplex immunofluorescence staining was performed using a four‑color multiplex IHC fluorescence kit (RC0086, Huilan Biological Technology, Shanghai, China) based on tyramide signal amplification (TSA) technology, following the manufacturer’s instructions. Briefly, deparaffinized and rehydrated paraffin sections underwent heat-induced antigen retrieval, 3% H_2_O_2_ peroxidase quenching, and nonspecific blocking. Sequential incubations with primary antibody, HRP secondary antibody and TSA fluorophore were followed by citrate heat stripping between each staining cycle. The antibody panel included anti‑CD8α (CST, #98941, 1:200), anti‑granzyme B (CST, #44153, 1:200), and anti‑lipoic acid (Abcam, ab58724, 1:200), each conjugated to spectrally distinct TSA fluorophores. Nuclei were counterstained with DAPI, and slides were mounted with anti‑fade mounting medium. For quantitative analysis, all slides were scanned under identical laser power and exposure settings. The mean fluorescence intensity (MFI) was quantified separately using ImageJ software. Relative fluorescence intensity was calculated by normalizing values to the control group for statistical comparison.

### Chromatin immunoprecipitation (ChIP)

Chromatin immunoprecipitation was performed using the Pierce Magnetic ChIP Kit (Thermo Fisher Scientific). Following the indicated culture and treatment conditions, protein–DNA complexes were crosslinked with formaldehyde and quenched with glycine. Cells were lysed in the Membrane Extraction Buffer, and chromatin was fragmented by optimized micrococcal nuclease (MNase) digestion. Fragmented chromatin was immunoprecipitated overnight with an anti‑ATF3 antibody (Abcam, ab207434) or control IgG, and immune complexes were captured with Protein A/G Magnetic Beads (Thermo Fisher Scientific). After washing, complexes were eluted, reverse-crosslinked, and digested with proteinase K, and DNA was purified using the spin columns. Enrichment of target genomic regions was quantified by qPCR using primers listed in Supplementary Table 1.

### Luciferase reporter assay

Firefly luciferase reporter plasmids containing wild-type or mutant promoter sequences of *Fdx1* or *Cox17* were constructed and co-transfected with the Renilla luciferase control plasmid pRL‑TK (GenePharma, Shanghai, China) into MOC1‑R cells using Lipofectamine 3000 (Invitrogen). Luciferase activities were measured using a dual-luciferase reporter assay system (Promega, WI, USA), and transcriptional activity was calculated as the firefly/Renilla luminescence ratio.

### Plasmid construction, transfection and RNA interference

For stable gene knockout, sgRNAs targeting *Fdx1*, *Atf3*, and *Afg3l2* were designed and cloned into the lentiCRISPRv2 vector (Addgene, MA, USA). Lentiviruses were packaged in 293 T cells and used to transduce MOC1‑R or 4T1‑R cells. At 36 h post-transduction, cells were selected with puromycin (5 μg mL^−1^; MilliporeSigma, MA, USA) for 2 days and maintained in puromycin-containing medium for continuous selection. For transient knockdown, cells were transfected with gene-specific or control siRNAs using Lipofectamine 3000 following the manufacturer’s protocol. Knockdown efficiency was evaluated 48 h later by immunoblotting or qRT–PCR. sgRNA and siRNA sequences are provided in Supplementary Tables 2 and 3.

### RNA sequencing and bioinformatics analysis

Total RNA was extracted from tumor cells using TRIzol reagent (Invitrogen). Sequencing libraries were prepared by Chi Biotech Co., Ltd. (Shenzhen, China), and paired-end sequencing was performed on a DNBSEQ‑T7 platform (MGI Tech). Raw reads were trimmed to remove adapters and low-quality bases, and high-quality reads were aligned to the mouse reference genome/transcriptome using FANSe3^52,53^. Gene expression was quantified using uniquely mapped reads. Differential expression analysis was performed with edgeR, which models count data with a negative binomial distribution. RNA-seq data have been deposited in the NCBI Gene Expression Omnibus (GEO) database under accession code GSE343347 and GSE343355.

### Statistical analysis

The data were presented as mean ± standard error of the mean (s.e.m.). Multiple-group comparisons were performed using one-way ANOVA with Tukey’s multiple comparisons test, and two-group comparisons were assessed using a two-tailed Student’s *t* test. Two-way ANOVA was used to compare tumor growth curves. Survival was analyzed using the log-rank (Mantel–Cox) test. Statistical analyses were conducted in GraphPad Prism (v8.0.2) and Microsoft Excel 2024. *P* < 0.05 was considered statistically significant.

### Reporting summary

Further information on research design is available in the Nature Portfolio Reporting Summary linked to this article.

## Supplementary information

Supplementary Information

Reporting Summary

Transparent Peer Review file

## Source data

Source Data

## Data Availability

RNA-seq data have been deposited in the NCBI Gene Expression Omnibus (GEO) database under accession code GSE343347 and GSE343355. The remaining data generated in this study are available within the Article, Supplementary Information, or Source Data file. Source data are provided with this paper.
