# Supplementary material for: A cuproptosis-inducing covalent organic framework synergizes with oncolytic virus for tumor radiotherapy
Source: Nat Commun. 2026 Aug 21;17:10011. doi: 10.1038/s41467-026-76947-w (PMC13590516; doi:10.1038/s41467-026-76947-w)
Supplement: Supplementary file 1 [file 41467_2026_76947_MOESM1_ESM.pdf]

Supplementary information

A cuproptosis-inducing covalent organic framework synergizes with oncolytic virus for tumor radiotherapy

Yuan-Tong Liu [1,2,#,*], Ke-Shan Liu [1,2,#], Han Li [2,#], Shuo Wang [1,2], Shu-Ying Bie [2], Ying-Qi Wei [2], Shan-Shan Gou [2], Xiang-Bo Wan [2], Xing-Zhen Li [3,*], Liang Zhang [3,*], Wei He [1,2,*]

[1] Department of Stomatology, the First Affiliated Hospital of Zhengzhou University, Zhengzhou, Henan 450052, China.

[2] State Key Laboratory of Metabolic Dysregulation & Prevention and Treatment of Esophageal Cancer, Tianjian Laboratory of Advanced Biomedical Sciences, Academy of Medical Sciences, Zhengzhou University, Zhengzhou, Henan 450052, China.

[3] State Key Laboratory of Precision and Intelligent Chemistry, School of Chemistry and Materials Science, University of Science and Technology of China, Hefei, Anhui 230026, China.

[#] These authors contributed equally: Yuan-Tong Liu, Ke-Shan Liu, and Han Li.

[*]Corresponding authors:

Yuan-Tong Liu, E-mail: yuantongdejia@zzu.edu.cn.

Xing-Zhen Li, E-mail: lixingzhen@ustc.edu.cn.

Liang Zhang, E-mail: zhangliang2025@ustc.edu.cn.

Wei He, E-mail: heweizdyfy@163.com.

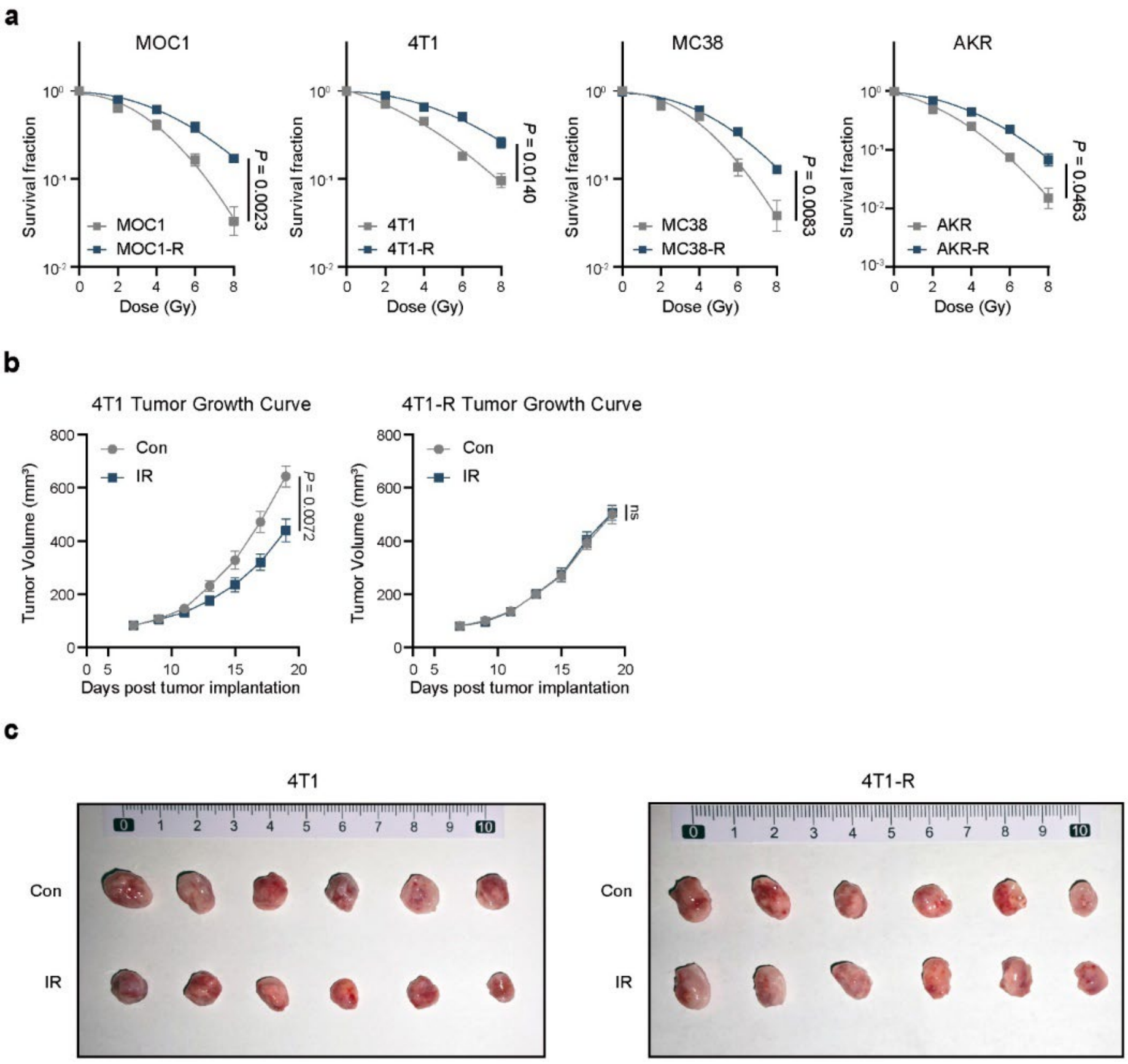

Supplementary Fig. 1. Establishment and validation of radioresistant tumor models. **a** Clonogenic survival curves of parental (MOC1, 4T1, MC38, AKR) and matched radioresistant (MOC1-R, 4T1-R, MC38-R, AKR-R) cell lines following exposure to escalating doses of ionizing radiation (IR) ($n = 3$ independent experiments). **b** Growth of 4T1 (parental) and 4T1-R (radioresistant) models in BALB/c mice treated with or without IR (4 Gy × 2) ($n = 6$ mice per group). **c** Representative images of excised 4T1 and 4T1-R tumors from control and IR-treated groups at the study endpoint. Statistical significance was assessed using two-tailed Student's *t* test (**a, b**). Data are presented as mean ± s.e.m. Source data are provided as a Source Data file.

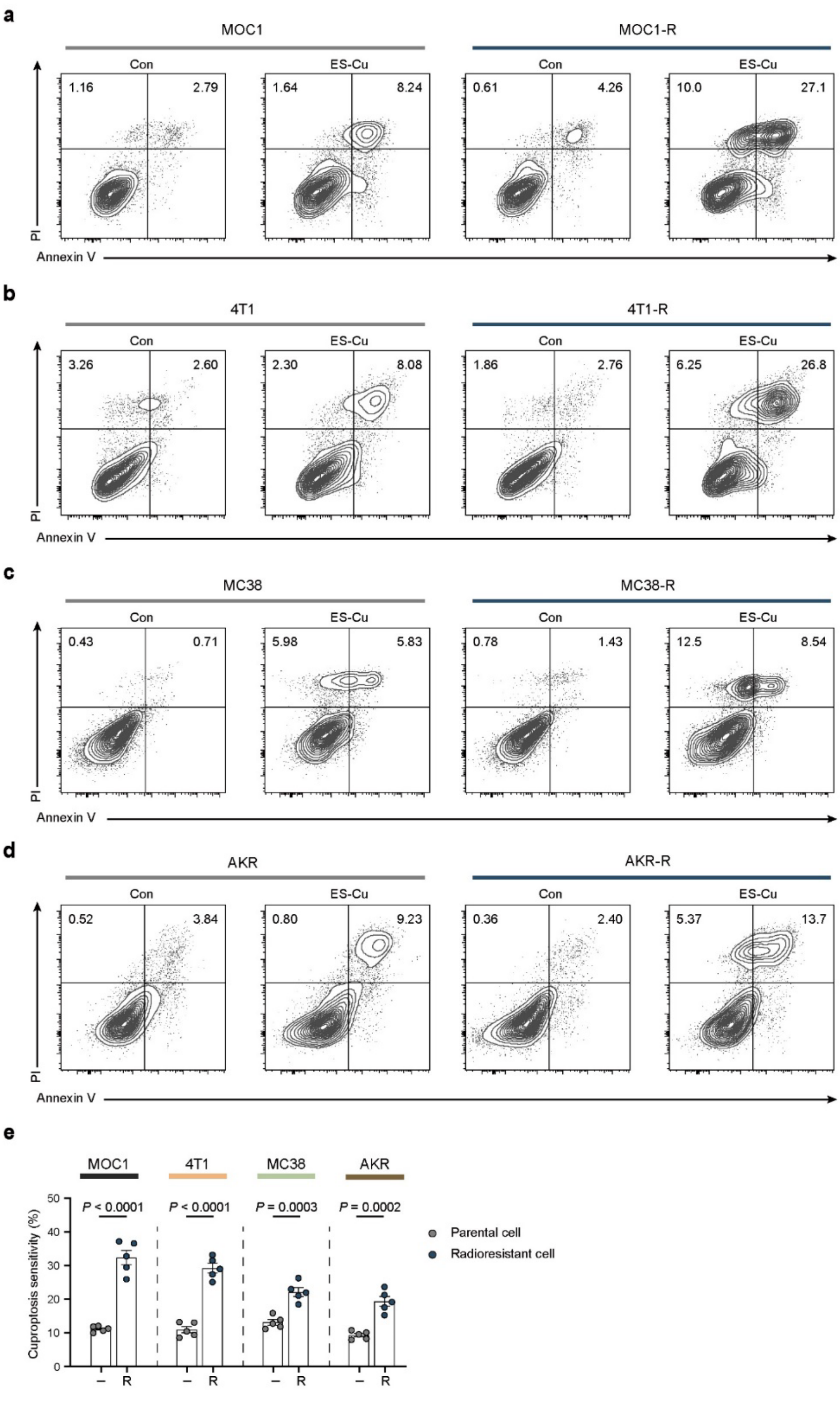

Supplementary Fig. 2. Radioresistant cells exhibit increased sensitivity to cuproptosis. a–d Representative Annexin V/PI flow-cytometry plots of parental (MOC1, 4T1, MC38, AKR) and matched radioresistant (MOC1-R, 4T1-R, MC38-R, AKR-R) cell lines after 24 h treatment with or without the cuproptosis inducer elesclomol–Cu (ES-Cu, 200 nM) (*n* = 5 independent experiments). **e** Quantification of cell death (percentage of PI-positive cells) in parental versus radioresistant cells following ES-Cu treatment (*n* = 5 independent experiments). Statistical significance was assessed using two-tailed Student's *t* test (**e**). Data are presented as mean ± s.e.m.

Source data are provided as a Source Data file.

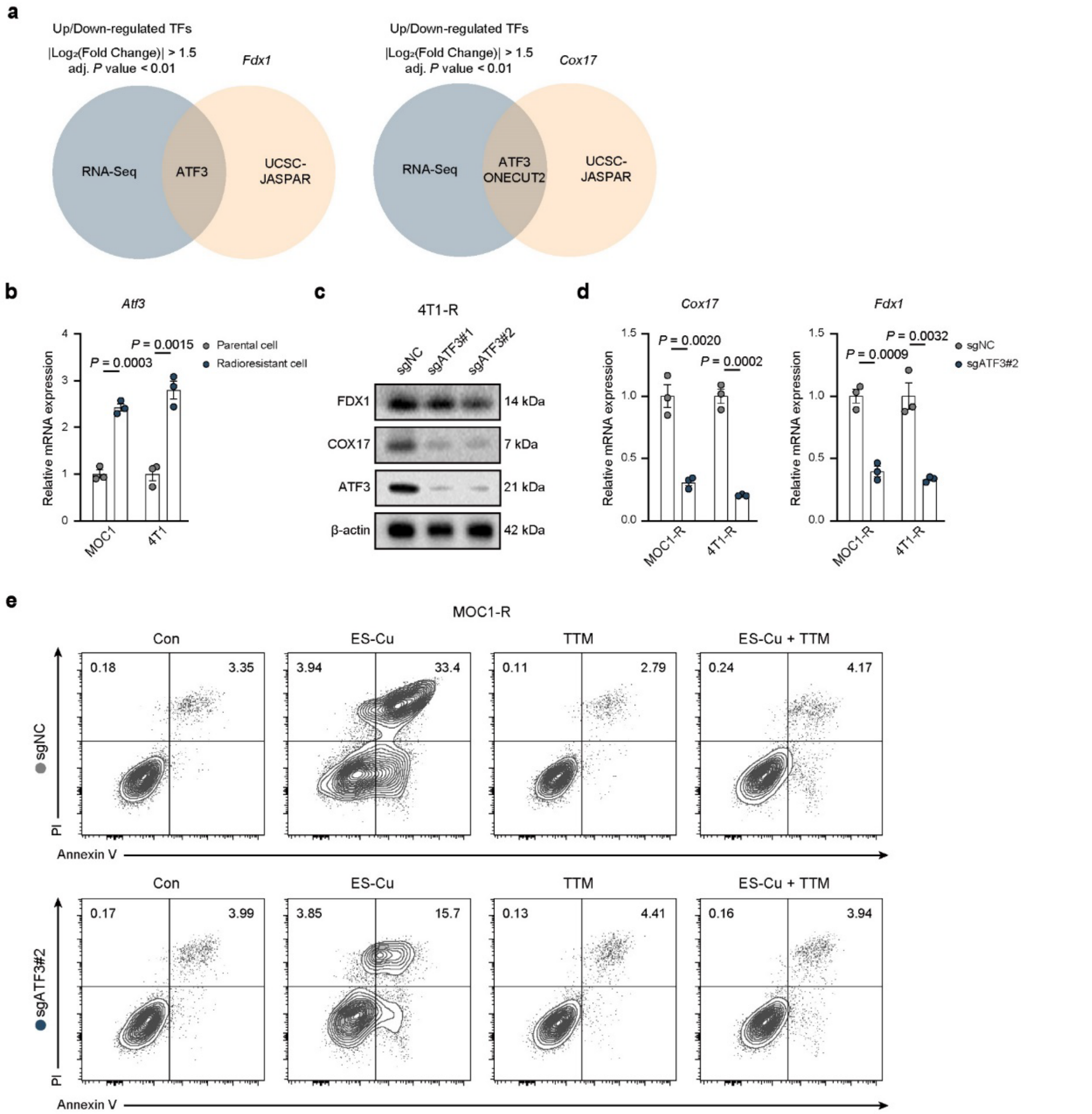

Supplementary Fig. 3. ATF3 confers cuproptosis sensitivity in radioresistant cells by regulating FDX1 and COX17 expression. a Venn diagram showing the overlap between transcription factors differentially expressed in MOC1 cells selected through fractionated irradiation relative to their corresponding parental control, as determined by RNA sequencing (*n* = 3 independent experiments), and transcription factors predicted by the JASPAR database to bind the *Fdx1* and *Cox17* promoter regions. ATF3 was identified as a candidate upstream regulator. **b** *Atf3* mRNA levels in parental and radioresistant (MOC1-R, 4T1-R) cells quantified by qPCR (*n* = 3 independent experiments). **c** Immunoblot analysis of FDX1, COX17, and ATF3 in 4T1-R cells transduced with sgATF3 or control sgNC. β-actin served as a loading control (representative of three independent experiments). **d** *Cox17* and *Fdx1* mRNA expression measured by qPCR in MOC1-R and 4T1-R cells transduced with sgATF3 or sgNC (*n* = 3 independent experiments). **e** Representative Annexin V/PI flow-cytometry plots of MOC1-R cells treated with DMSO, ES-Cu (200 nM), TTM (40 μM), or ES-Cu + TTM (*n* = 3 independent experiments). Statistical significance was assessed

using two-tailed Student's *t* test (**b, d**). Data are presented as mean ± s.e.m. Source data are provided as a Source Data file.

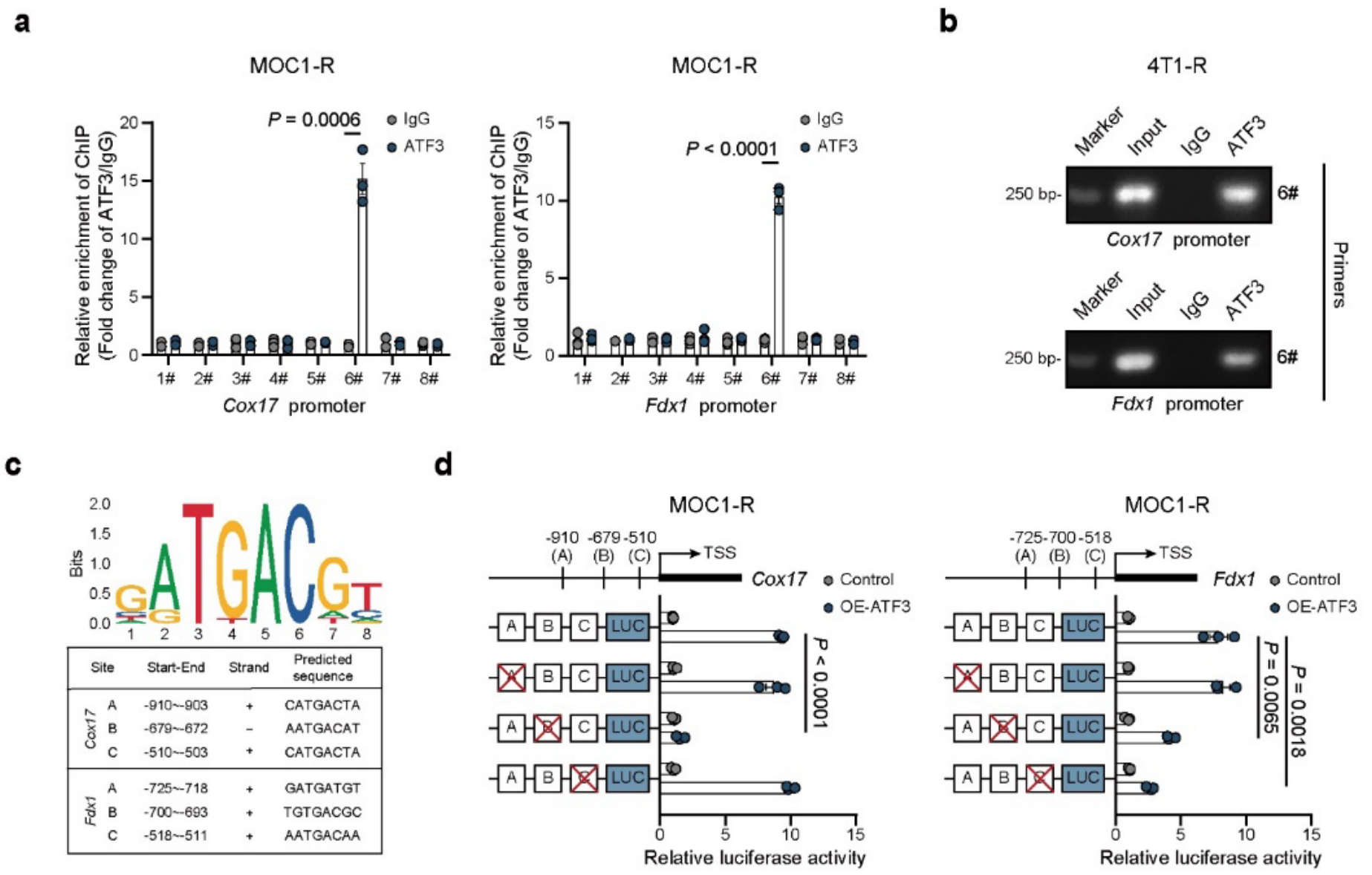

Supplementary Fig. 4. ATF3 directly binds to and activates the *Cox17* and *Fdx1* promoters. a ChIP–qPCR using an anti-ATF3 antibody to quantify enrichment at *Cox17* and *Fdx1* promoter fragments (*n* = 3 independent experiments). **b** ChIP–PCR followed by agarose gel electrophoresis confirming ATF3 binding to the *Cox17* and *Fdx1* promoters in 4T1-R cells (IgG as negative control) (representative of three independent experiments). **c** Predicted ATF3-binding motifs and their positions within the *Cox17* and *Fdx1* promoters. **d** Dual-luciferase reporter assays validating functional ATF3-binding sequences within the *Cox17* and *Fdx1* promoters (*n* = 3 independent experiments). Statistical significance was assessed using two-tailed Student's *t* test (**a, d**). Data are presented as mean ± s.e.m. Source data are provided as a Source Data file.

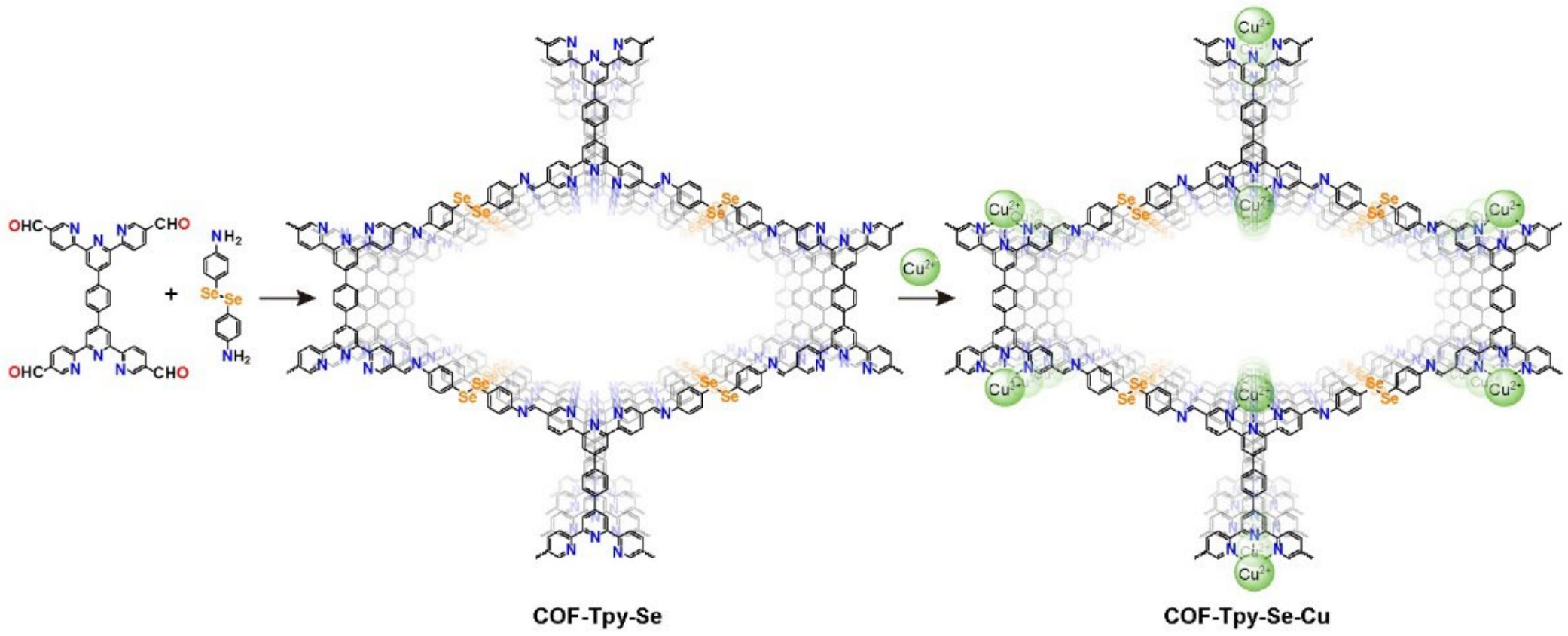

Supplementary Fig. 5. Synthetic route for COF-Tpy-Se-Cu.

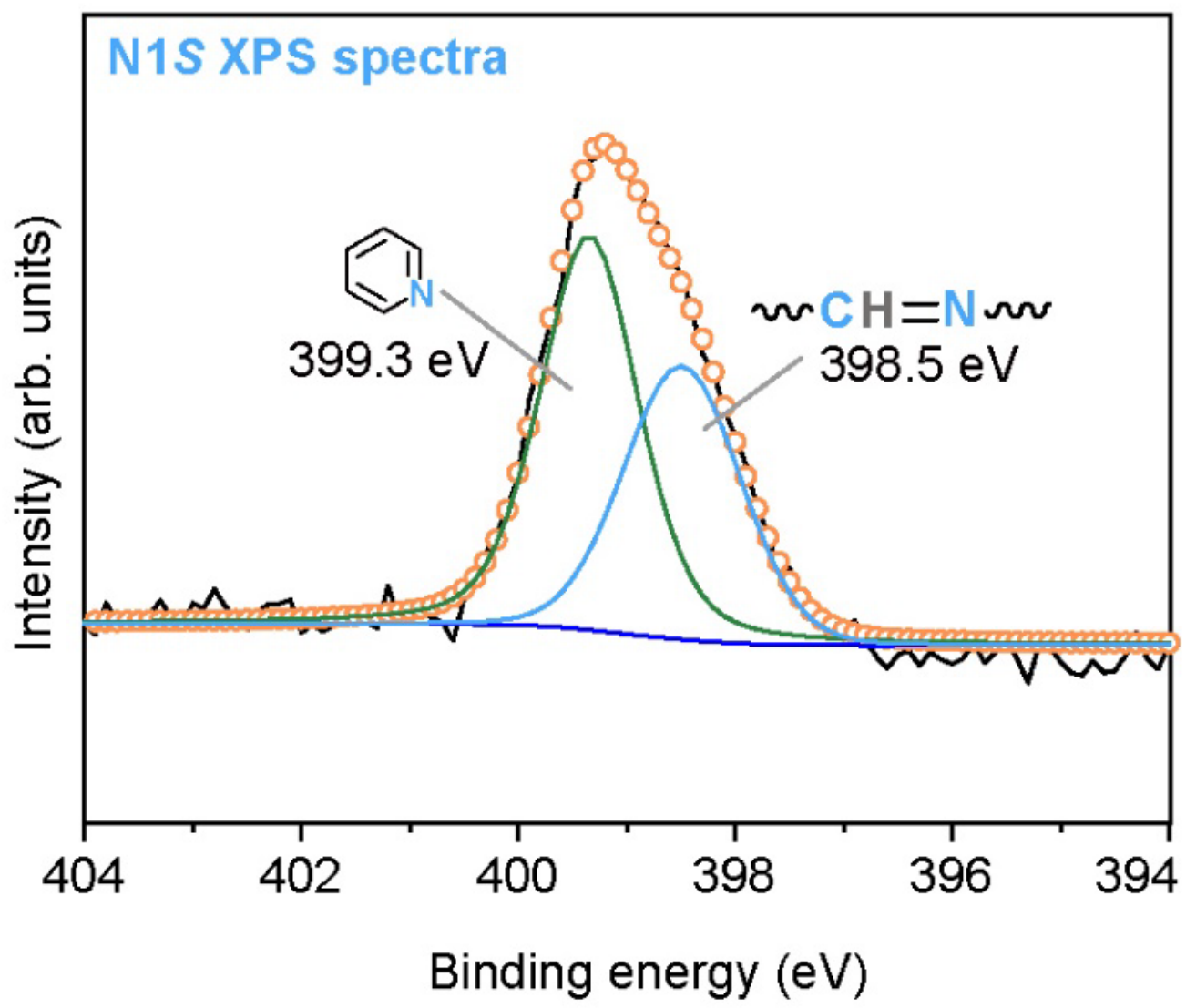

Supplementary Fig. 6. Characterization of COF-Tpy-Se-Cu. N1*s* X-ray photoelectron spectroscopy (XPS) spectrum of COF-Tpy-Se-Cu (representative of three independent measurements). Source data are provided as a Source Data file.

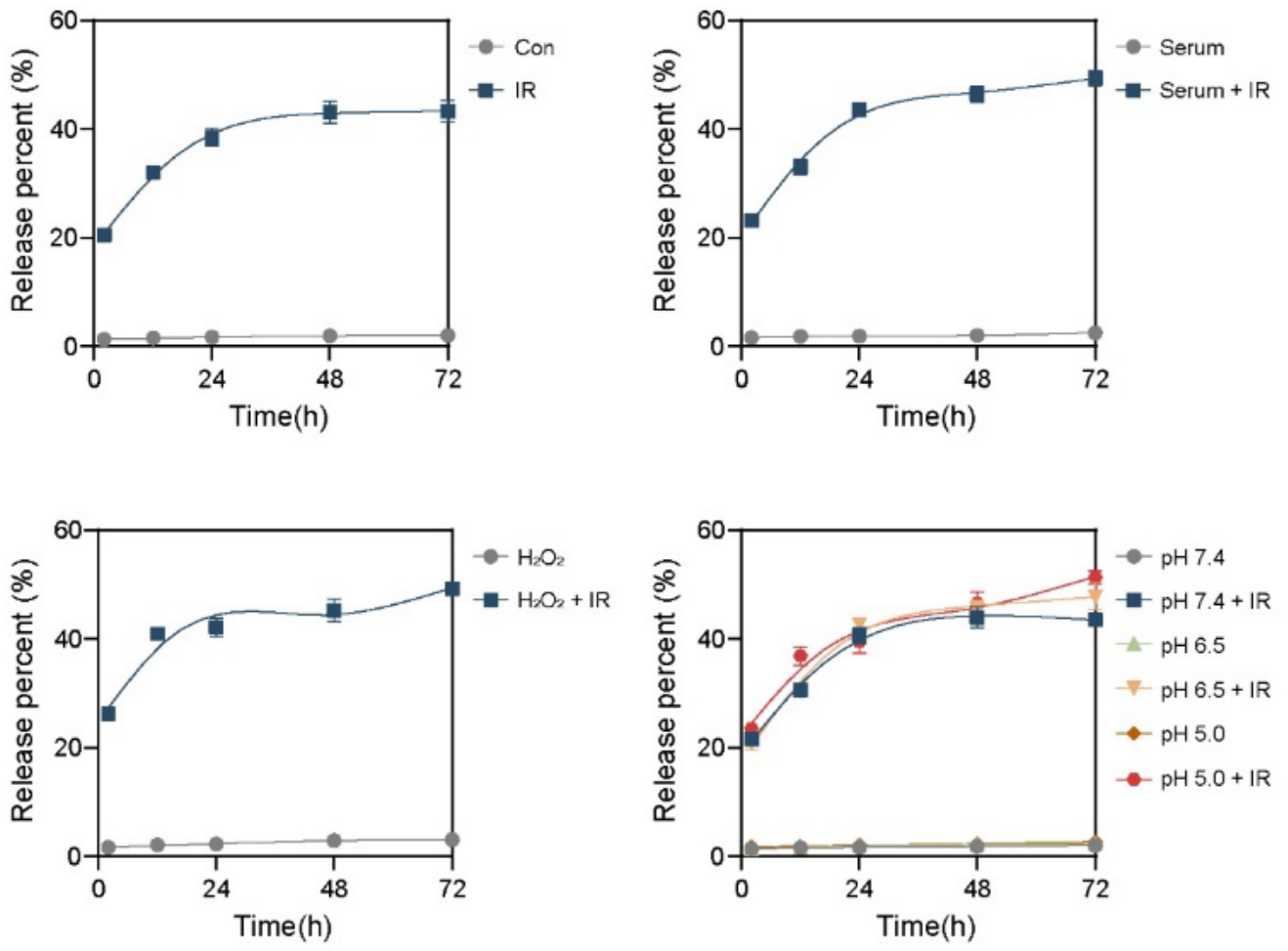

Supplementary Fig. 7. Copper release kinetics of COF-Tpy-Se-Cu under pathophysiologically relevant conditions. Time-dependent copper release profiles were determined over 72 h with or without 4 Gy X-ray irradiation, across a panel of conditions including neutral physiological buffer, 10% serum, 10 mM glutathione (GSH), 100 μM hydrogen peroxide (H_2O_2), and acidic buffers (pH 6.5 and pH 5.0) mimicking the tumor microenvironment (*n* = 3 independent experiments). Data are presented as mean ± s.e.m. Source data are provided as a Source Data file.

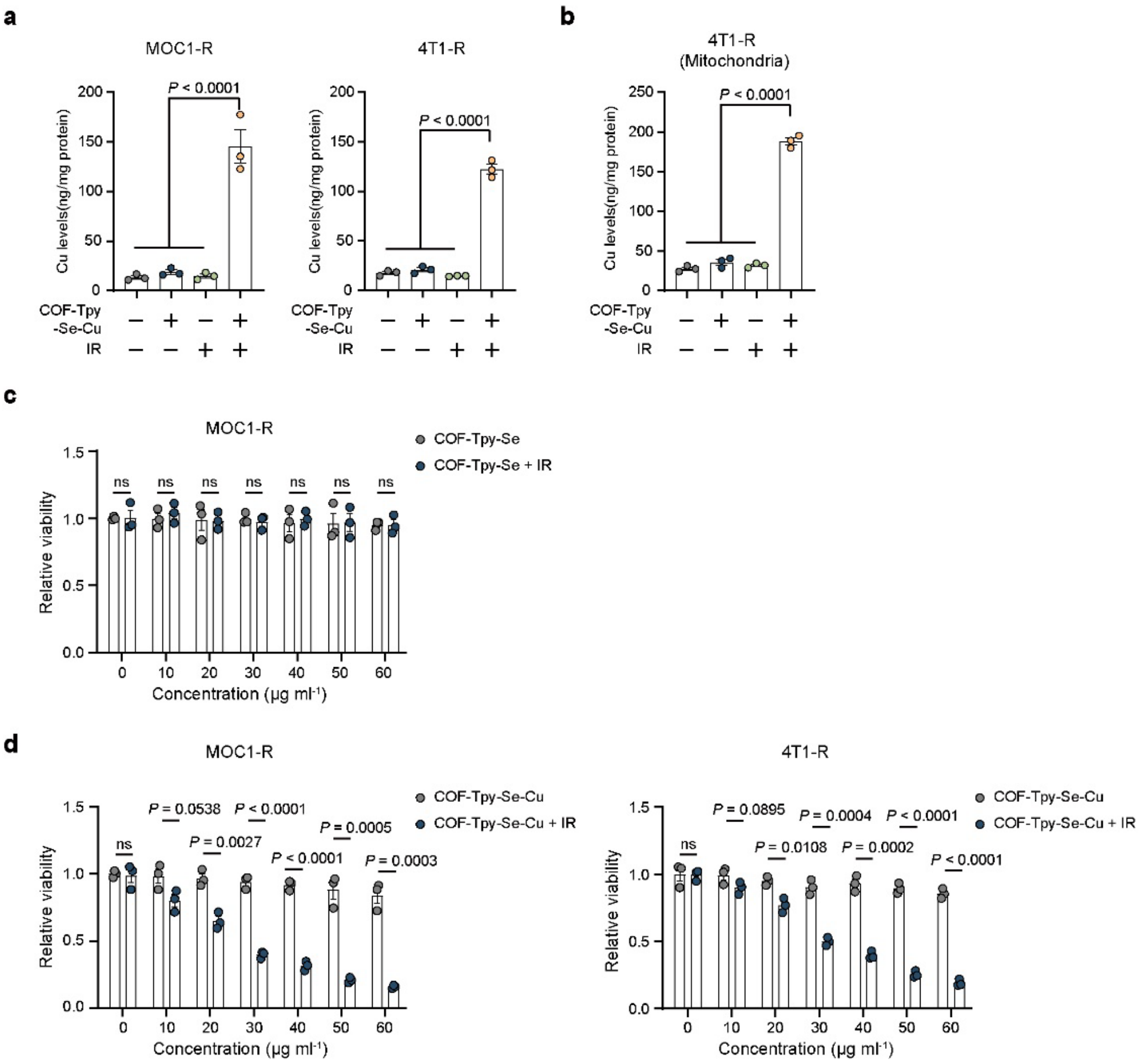

Supplementary Fig. 8. COF-Tpy-Se-Cu elevates cellular and mitochondrial copper and reduces viability upon irradiation. a Total intracellular copper quantified by ICP–MS in MOC1-R (left) and 4T1-R (right) cells after treatment with COF-Tpy-Se-Cu (30 µg mL^{-1}, 12 h) with or without irradiation (IR, 4 Gy), normalized to total protein (n = 3 independent experiments). **b** Mitochondrial copper levels measured by ICP–MS in 4T1-R cells under the same conditions and normalized to mitochondrial protein (n = 3 independent experiments). **c** Cell viability of MOC1-R and 4T1-R cells treated with increasing concentrations of the copper-free analog COF-Tpy-Se, with or without IR (4 Gy), assessed by CCK-8 assay at 24 h (n = 3 independent experiments). **d** Cell viability of MOC1-R (left) and 4T1-R (right) cells treated with increasing concentrations of COF-Tpy-Se-Cu, with or without IR (4 Gy), measured by CCK-8 assay at 24 h (n = 3 independent experiments). Statistical significance was assessed using one-way ANOVA with Tukey's multiple-comparisons test (**a, b**) and two-tailed Student's *t* test (**c, d**). Data are presented as mean ± s.e.m. Source data are provided as a Source Data file.

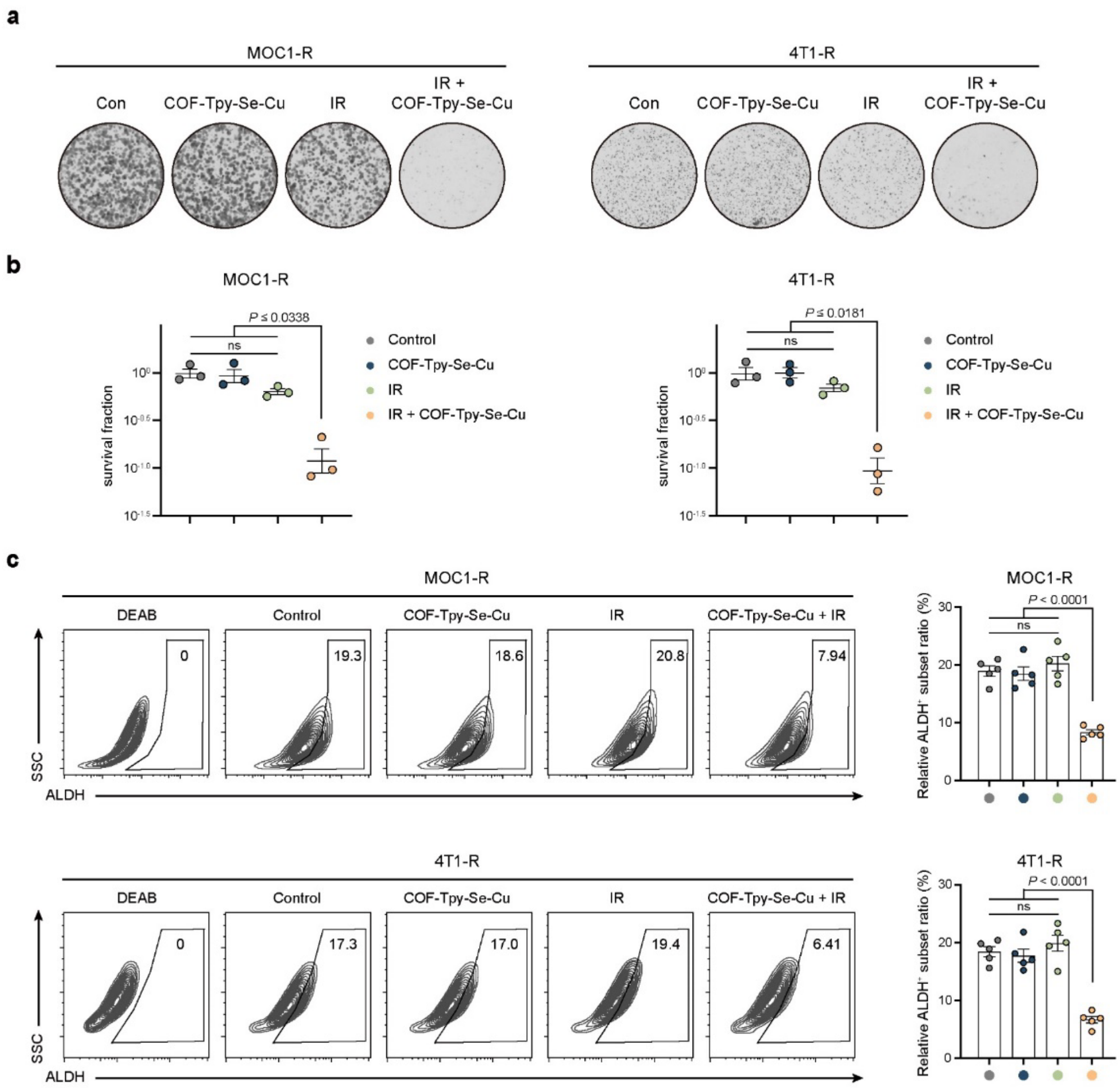

Supplementary Fig. 9. COF-Tpy-Se-Cu–mediated radiosensitization in vitro. a Representative images of crystal violet-stained colonies of MOC1-R (left) and 4T1-R cells (right) following the indicated treatments (COF-Tpy-Se-Cu, 30 μg mL^{-1}; IR, 4 Gy) after 7 days (n = 3 independent experiments). **b** Quantification of survival fraction from clonogenic survival assays (n = 3 independent experiments). **c** ALDH activity assessed by flow cytometry in MOC1-R (top) and 4T1-R (bottom) cells across the indicated treatment groups (COF-Tpy-Se-Cu, 30 μg mL^{-1}; IR, 4 Gy) (n = 5 independent experiments). Statistical significance was assessed using one-way ANOVA with Tukey's multiple-comparisons test (**b, c**). Data are presented as mean ± s.e.m. Source data are provided as a Source Data file.

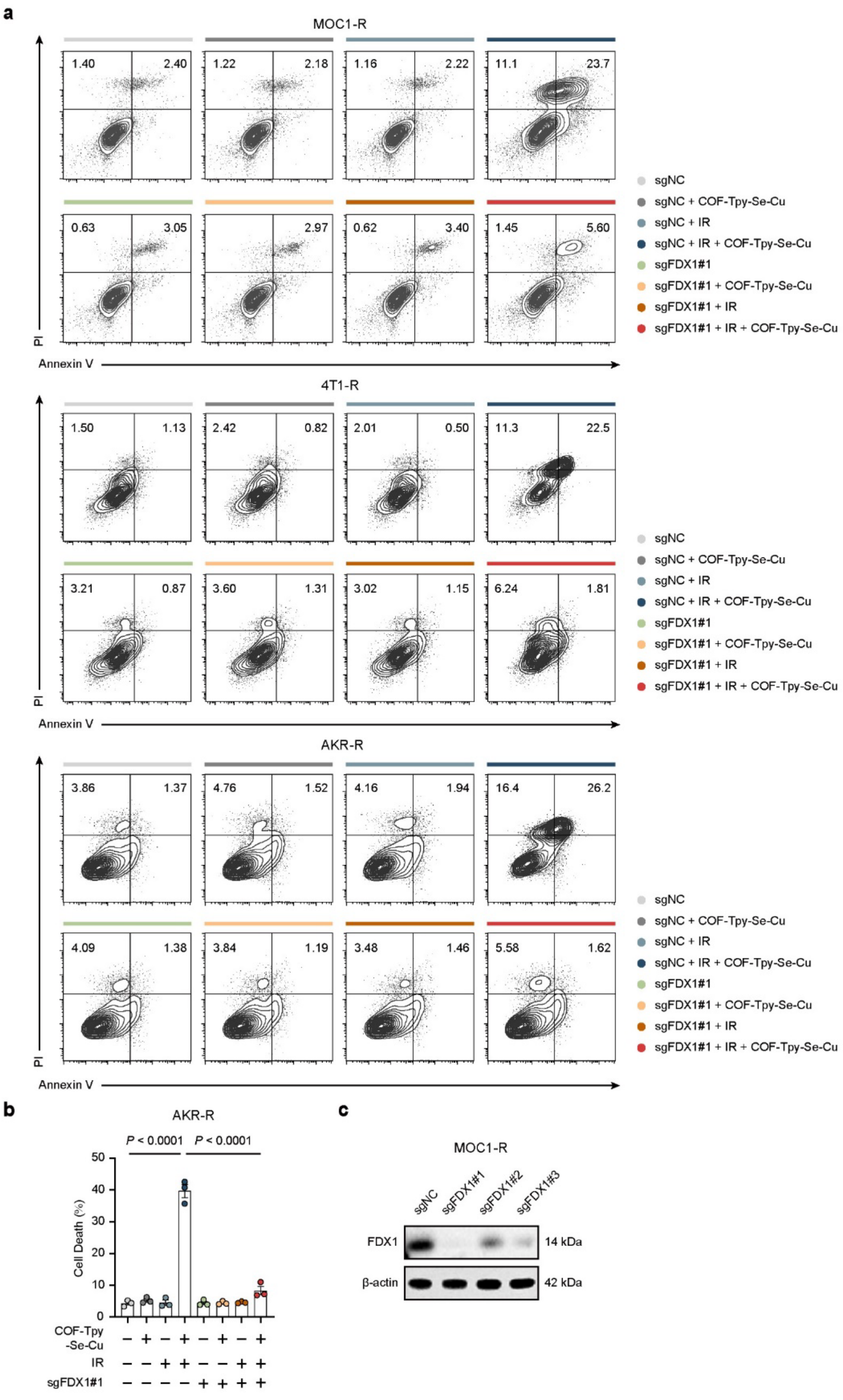

Supplementary Fig. 10. FDX1 is required for COF-Tpy-Se-Cu-induced cell death. **a** Representative Annexin V/PI flow-cytometry plots of radioresistant MOC1-R, 4T1-R, and AKR-R cells treated with COF-Tpy-Se-Cu (30 μg mL^{-1}) plus irradiation (IR, 4 Gy) for 24 h (*n* = 3 independent experiments). **b** Quantification of cell death (percentage of PI-positive cells) in AKR-R cells under the indicated treatments (*n* = 3 independent experiments). **c** Immunoblot confirming FDX1 knockout efficiency in MOC1-R cells

(representative of three independent experiments). Statistical significance was assessed using one-way ANOVA with Tukey's multiple-comparisons test (**b**). Data are presented as mean ± s.e.m. Source data are provided as a Source Data file.

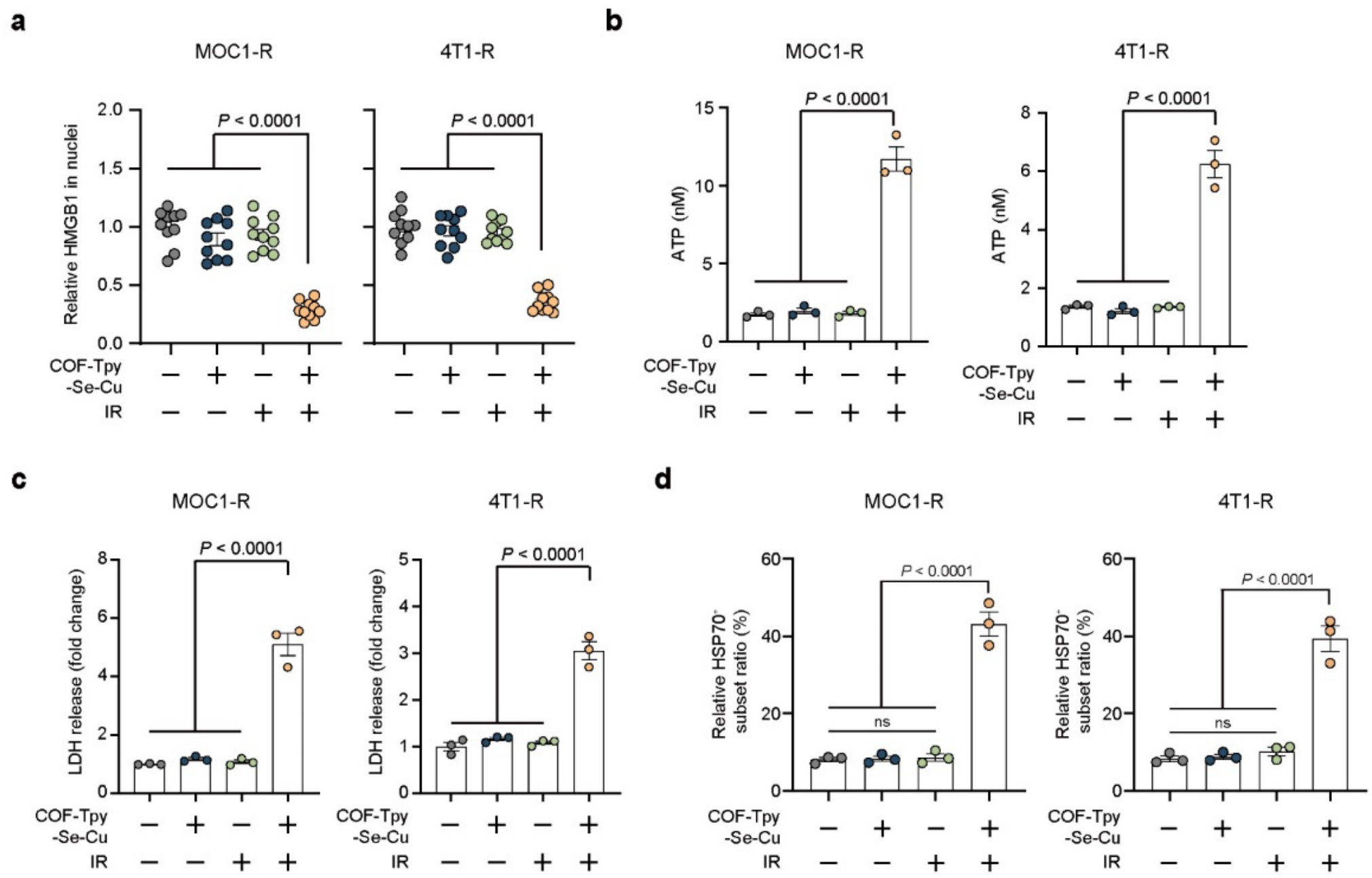

Supplementary Fig. 11. COF-Tpy-Se-Cu plus irradiation induces the release of immunogenic cell death markers. a Quantification of nuclear HMGB1 fluorescence intensity in MOC1-R and 4T1-R cells after 24 h treatment with COF-Tpy-Se-Cu (30 μg mL^{-1}) and/or irradiation (IR, 4 Gy) (n = 10 independent fields). **b, c** Extracellular release of ATP (b) and lactate dehydrogenase (LDH) (c) from MOC1-R and 4T1-R cells following the indicated treatments for 24 h (n = 3 independent experiments). **d** Flow cytometric quantification of the relative HSP70-positive cell subset ratio in MOC1-R and 4T1-R cells following the indicated treatments (n = 3 independent experiments). Statistical significance was assessed using one-way ANOVA with Tukey's multiple-comparisons test (**a–d**). Data are presented as mean ± s.e.m. Source data are provided as a Source Data file.

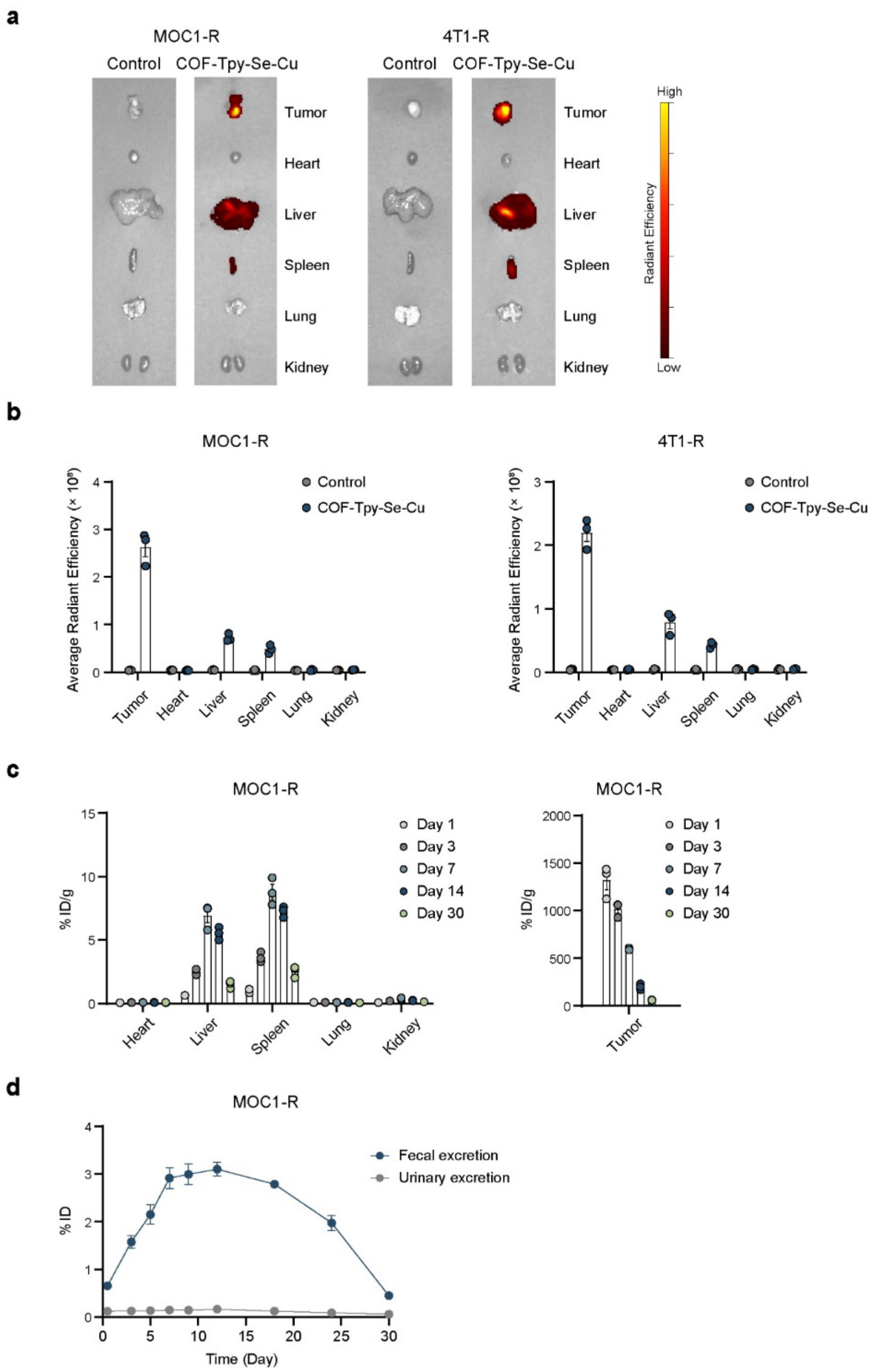

Supplementary Fig. 12. In vivo biodistribution and excretion profile of COF-Tpy-Se-Cu in tumor-bearing mice. a Ex vivo fluorescence imaging of tumor tissues and major organs (heart, liver, spleen, lung, kidney) from mice bearing MOC1-R orthotopic or 4T1-R subcutaneous tumors at 7 days after intratumoral injection of Cy5.5-loaded COF-Tpy-Se-Cu. The color scale indicates relative radiant efficiency ($n = 3$ biologically independent mice per tumor model). **b** Quantitative analysis of average radiant efficiency in tumor and each organ ($n = 3$ biologically independent mice per tumor model). **c** Time-course biodistribution of COF-Tpy-Se-Cu in major organs (left) and tumor tissues (right) of MOC1-R tumor-bearing mice following intratumoral injection, quantified by inductively coupled plasma mass spectrometry (ICP-MS) using selenium as the elemental tracer. COF-Tpy-Se-Cu content is expressed as the percentage of

injected dose per gram of tissue (% ID/g) (n = 3 biologically independent mice). **d** Daily fecal and urinary excretion rates of COF-Tpy-Se-Cu in MOC1-R tumor-bearing mice over 30 days post-injection. Data are presented as the percentage of injected dose per day (% ID/day) (n = 3 biologically independent mice). Data are presented as mean ± s.e.m. Source data are provided as a Source Data file.

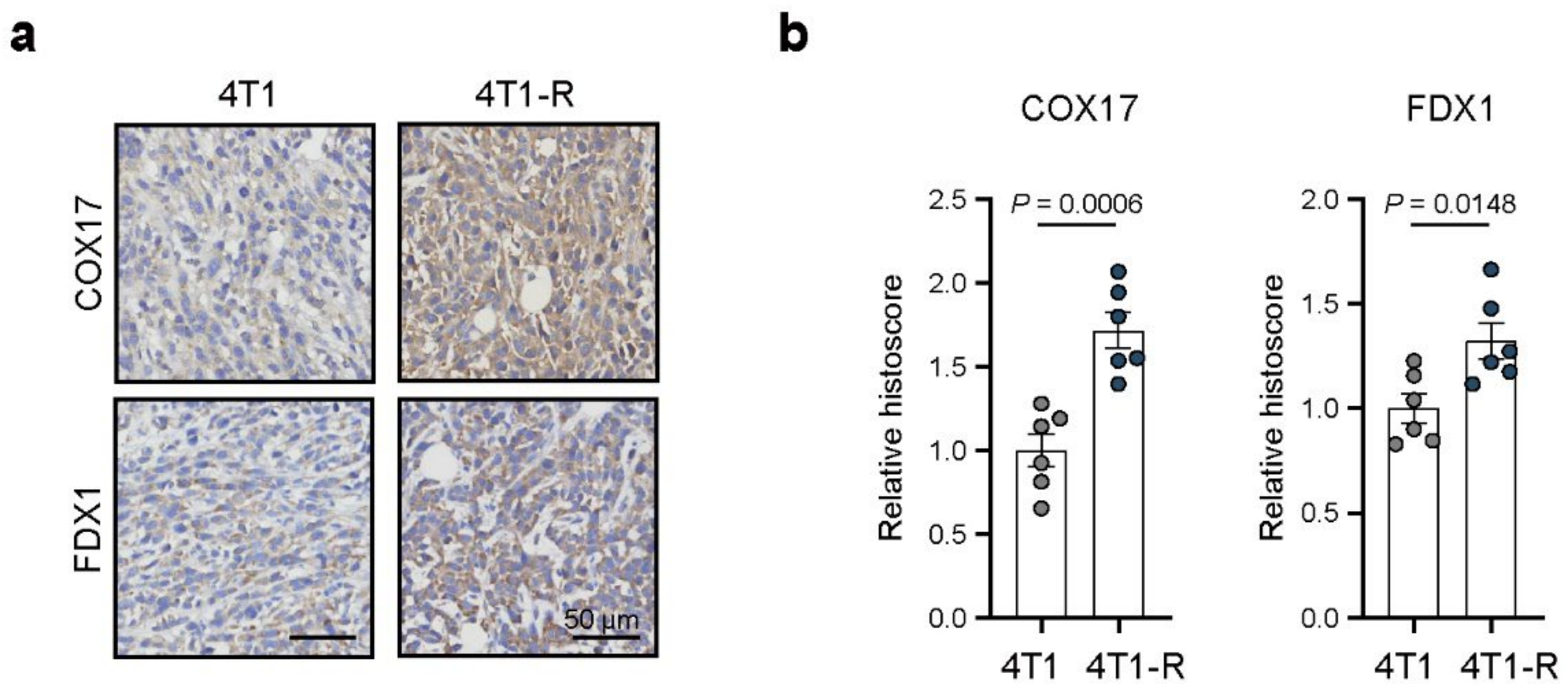

Supplementary Fig. 13. COX17 and FDX1 are upregulated in radioresistant tumors. a Representative immunohistochemistry images of COX17 and FDX1 in paraffin-embedded sections from parental 4T1 and radioresistant 4T1-R models (scale bar, 50 μm) (n = 6 biologically independent mice). **b** Quantitative histoscores for COX17 and FDX1 in 4T1 versus 4T1-R tumor sections (n = 6 biologically independent mice). Statistical significance was assessed using two-tailed Student's t test (**b**). Data are presented as mean ± s.e.m. Source data are provided as a Source Data file.

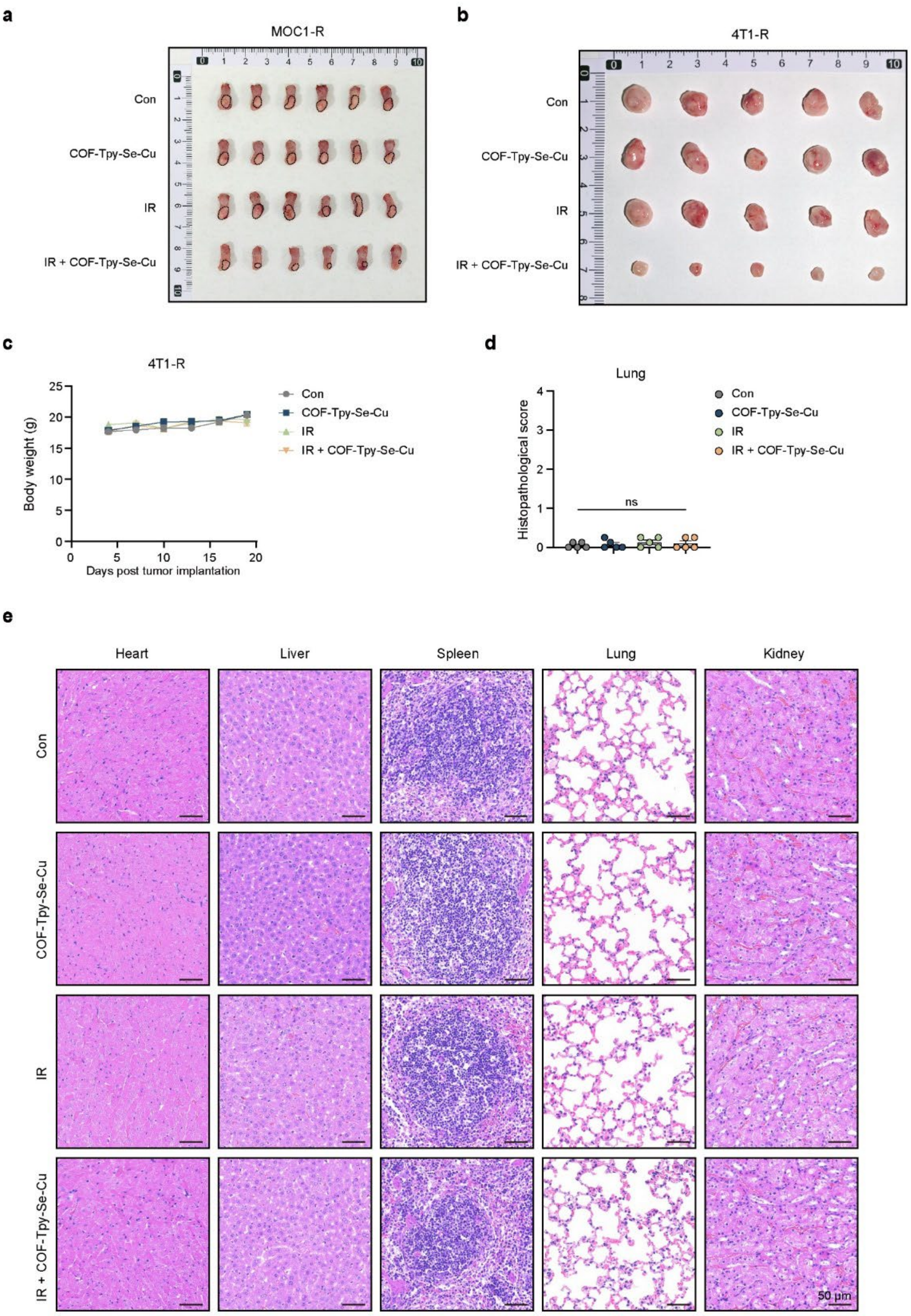

Supplementary Fig. 14. In vivo therapeutic efficacy and safety assessment. a Representative images of excised tongues from mice bearing orthotopic MOC1-R tumors at the study endpoint (n = 6 mice per group). Tumor regions are delineated by black dashed lines. **b** Representative images of resected subcutaneous 4T1-R tumors from each treatment group at the endpoint (n = 5 mice per group). **c** Body-weight changes during the treatment period (n = 5 mice per group). **d** Blinded semi-quantitative histopathological scoring of H&E-stained lung tissue sections across the indicated treatment groups (n = 5 mice per group). **e** Representative hematoxylin and eosin

(H&E)–stained sections of the heart, liver, spleen, lung, and kidney from treated and control BALB/c mice are shown (scale bar, 50 µm). No overt histopathological abnormalities were observed in any treatment group relative to controls. Images are representative of five mice per group, with consistent findings across all groups. Statistical significance was assessed using one-way ANOVA with Tukey's multiple-comparisons test (**c, d**). Data are presented as mean ± s.e.m. Source data are provided as a Source Data file.

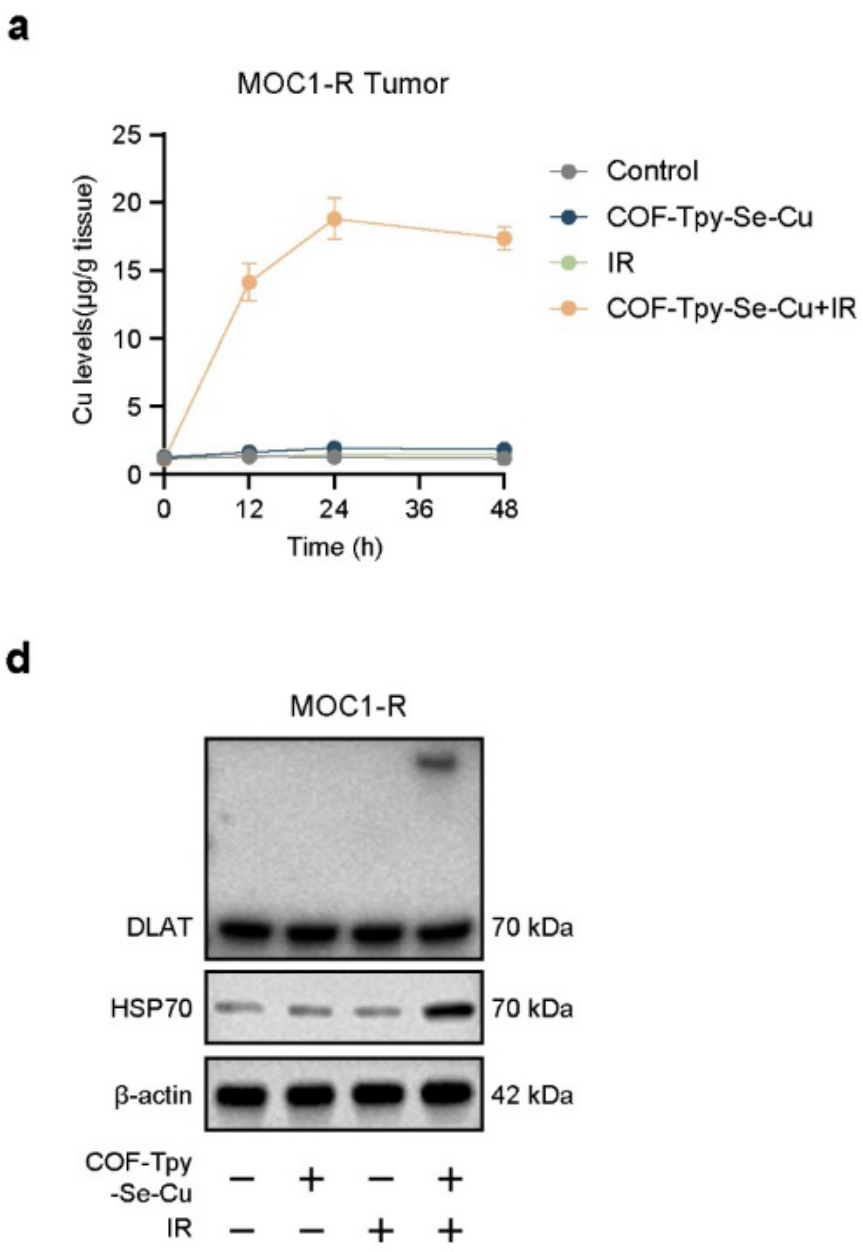

Supplementary Fig. 15. Radiation-driven intratumoral copper accumulation and induction of cuproptosis hallmarks by COF-Tpy-Se-Cu in vivo. a Time-course ICP-MS quantification of copper concentrations in tumor tissues from the MOC1-R model across different treatment groups (COF-Tpy-Se-Cu, 2 mg kg^{-1}; IR, 4 Gy). Copper levels are normalized to tumor tissue weight (*n* = 3 independent tumor samples). **b** ICP-MS quantification of total cellular copper levels in MOC1-R tumor tissues 24 h after the indicated treatments. Copper contents are normalized to total cellular protein (*n* = 3 independent tumor samples). **c** ICP-MS quantification of mitochondrial copper levels in MOC1-R tumor tissues 24 h after the indicated treatments, normalized to mitochondrial protein (*n* = 3 independent tumor samples). **d** Immunoblot analysis of DLAT oligomerization and the proteotoxic stress marker HSP70 in MOC1-R tumor tissue lysates. β-actin served as the loading control (representative of three independent experiments). Statistical significance was assessed using one-way ANOVA with Tukey's multiple-comparisons test (**b, c**). Data are presented as mean ± s.e.m. Source data are provided as a Source Data file.

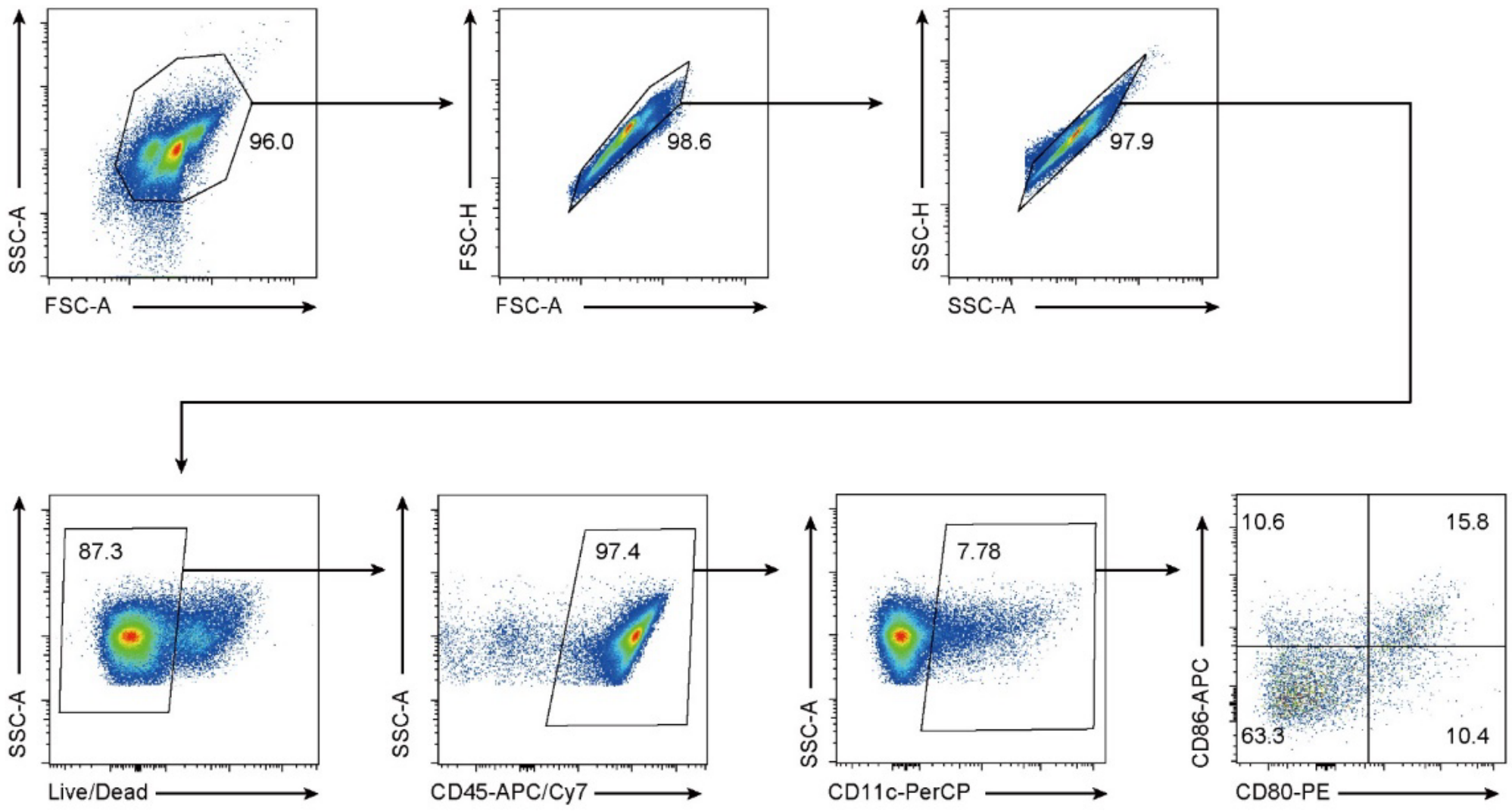

Supplementary Fig. 16. Flow-cytometry gating strategy for dendritic cells in tumor-draining lymph nodes. The sequential gating strategy shown here corresponds to the dendritic-cell analyses presented in Fig. 5d, Supplementary Fig. 20a, Supplementary Fig. 35b, and Supplementary Fig. 37c.

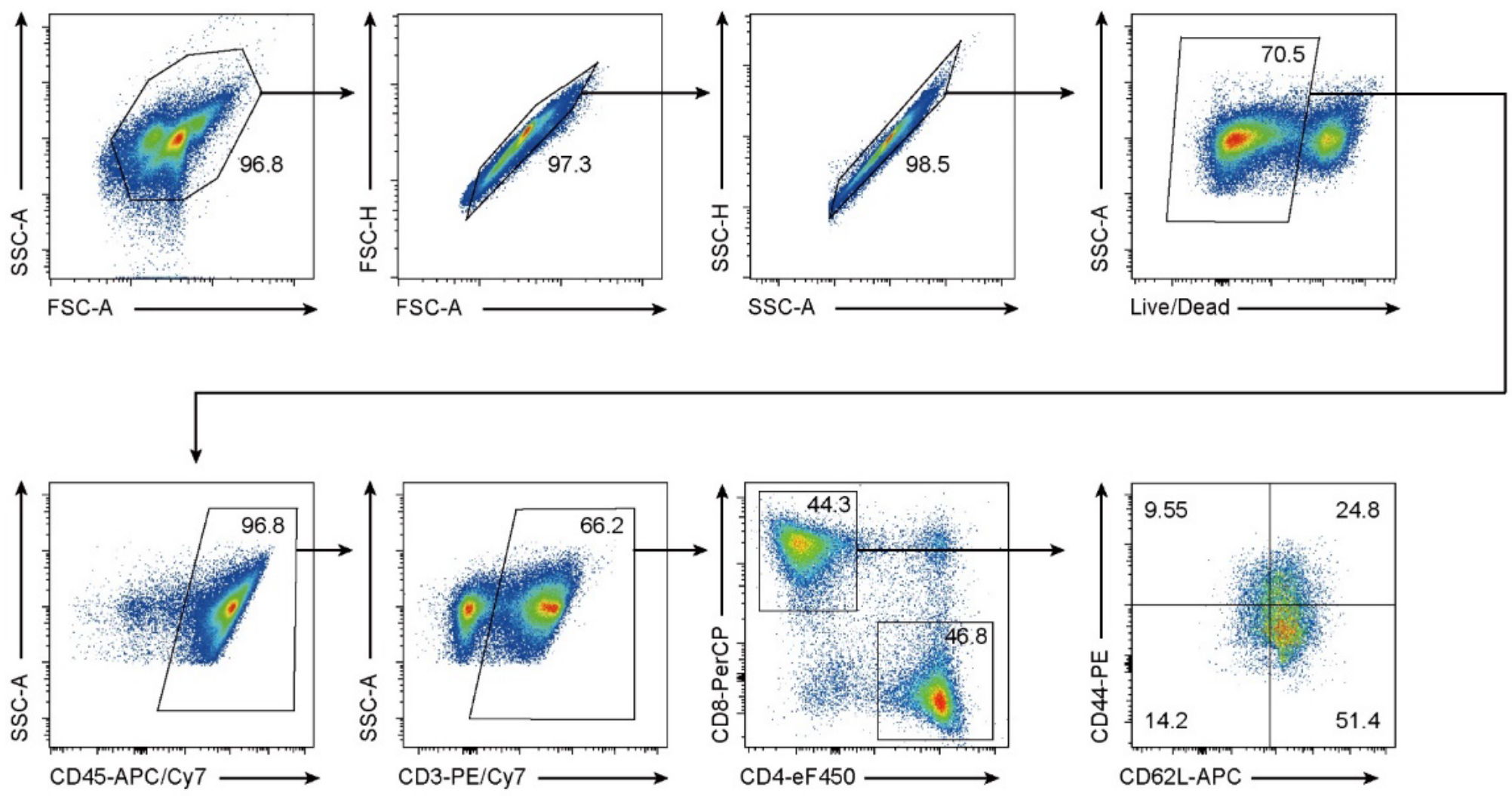

Supplementary Fig. 17. Flow-cytometry gating strategy for T-cell populations. The sequential gating strategy shown here corresponds to the $CD4^+$ and $CD8^+$ T-cell analyses presented in Fig. 5f, Supplementary Fig. 20b, Supplementary Fig. 35c, Supplementary Fig. 37g, and Supplementary Fig. 38c, and to the memory $CD8^+$ T-cell analyses presented in Fig. 5h, Supplementary Fig. 20e, Supplementary Fig. 35f, and Supplementary Fig. 37e.

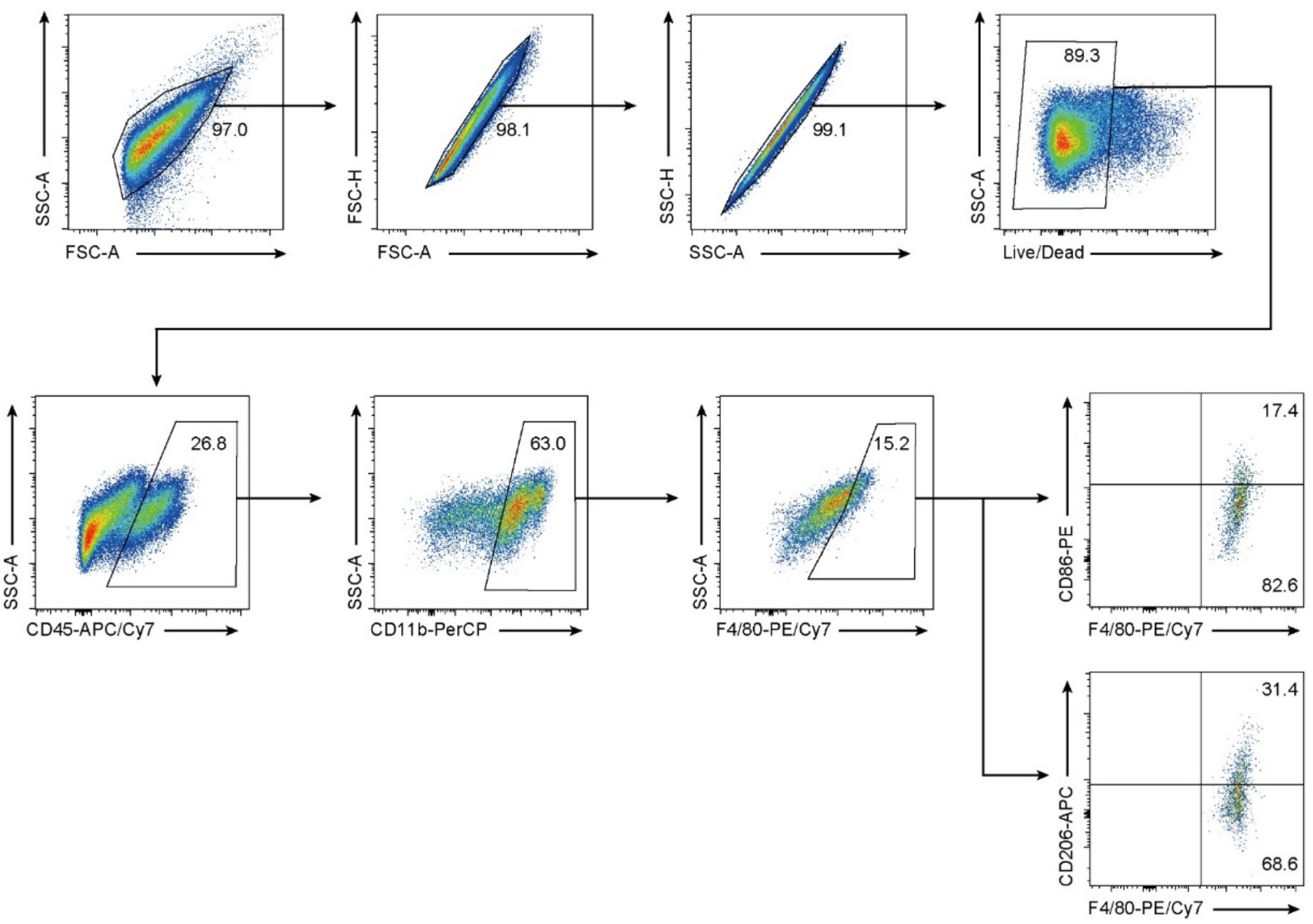

Supplementary Fig. 18. Flow-cytometry gating strategy for tumor-associated macrophages in tumor tissues. The sequential gating strategy shown here corresponds to the tumor-associated macrophage analyses presented in Supplementary Fig. 36a.

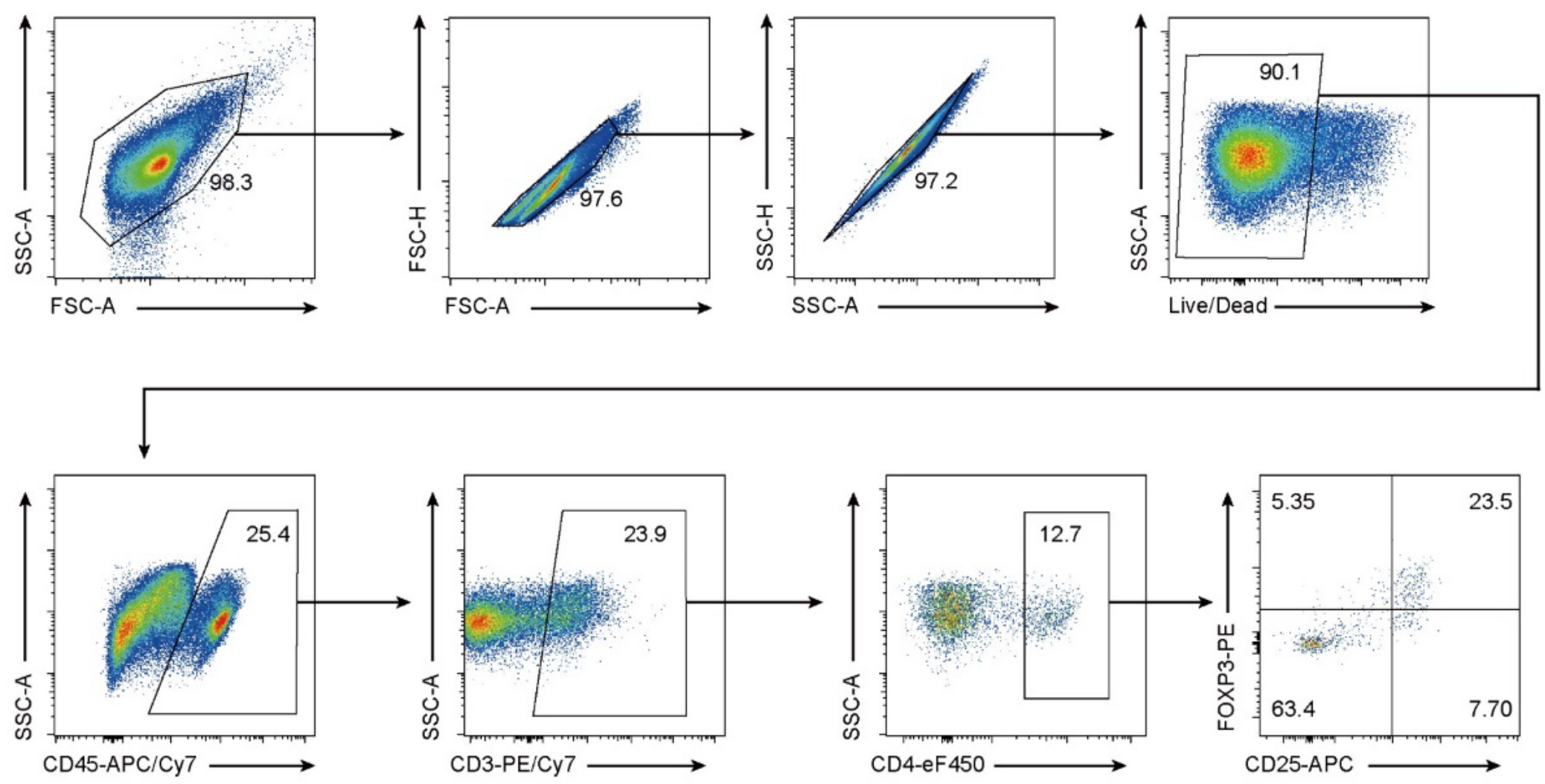

Supplementary Fig. 19. Flow-cytometry gating strategy for regulatory T cells in tumor tissues. The sequential gating strategy shown here corresponds to the regulatory T-cell analyses presented in Supplementary Fig. 36b.

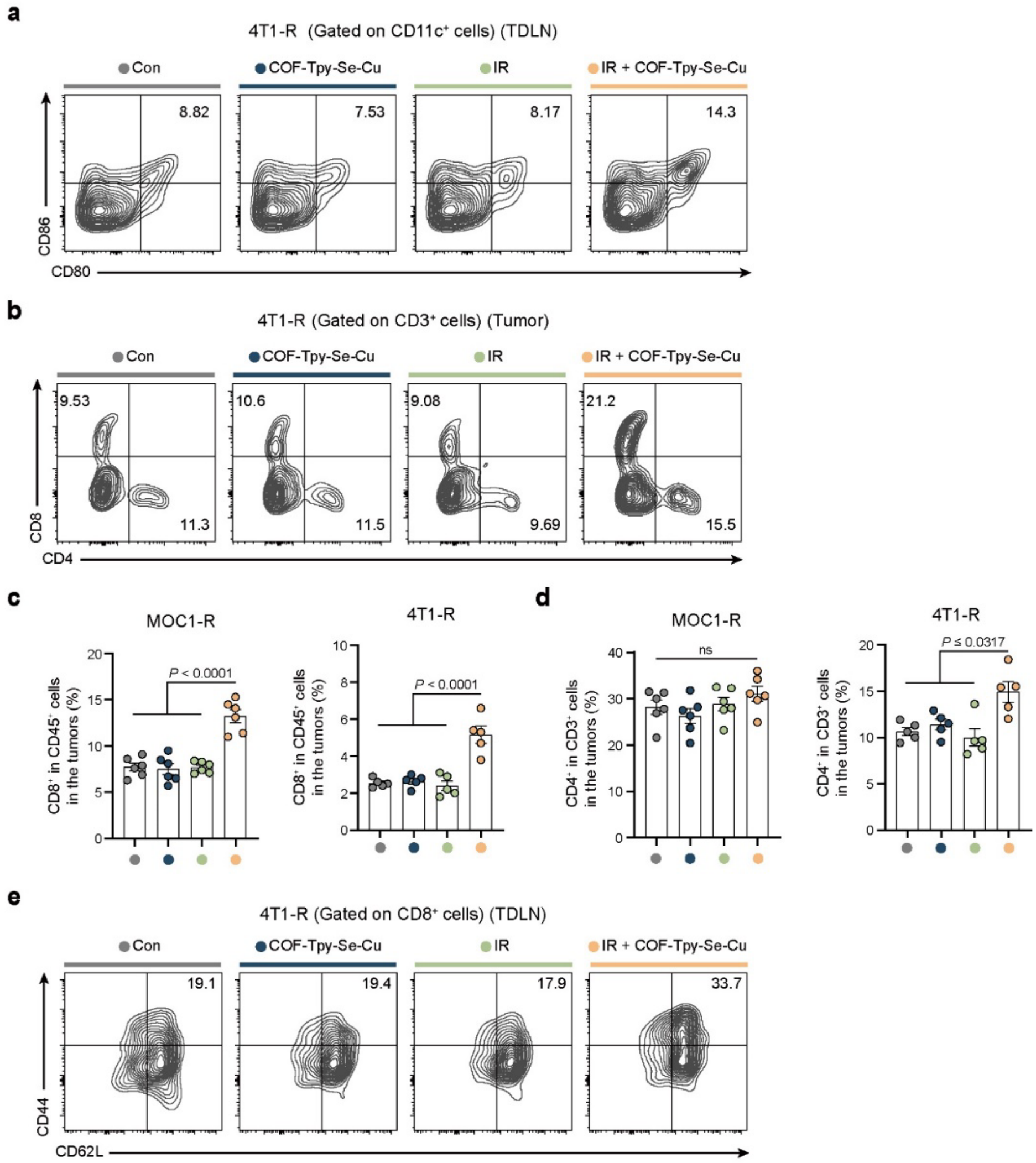

Supplementary Fig. 20. COF-Tpy-Se-Cu plus irradiation reshapes the tumor immune microenvironment. a Representative plots showing the frequency of $CD80^+CD86^+$ cells among $CD11c^+$ DCs in tumor-draining lymph nodes (TDLNs) from 4T1-R models (*n* = 5 mice per group). **b** Flow cytometric analysis of tumor-infiltrating $CD8^+$ T and $CD4^+$ T cells among $CD3^+$ T cells from 4T1-R models (*n* = 5 mice per group). **c** Quantification of $CD8^+$ T cell frequency among $CD45^+$ leukocytes in MOC1-R (left, *n* = 6 mice per group) and 4T1-R (right, *n* = 5 mice per group) tumors. **d** Quantification of $CD4^+$ T cell frequency among $CD3^+$ T cells in MOC1-R (left, *n* = 6) and 4T1-R (right, *n* = 5 mice per group) tumors. **e** Frequency of $CD44^+CD62L^+$ memory T cells among $CD8^+$ T cells in TDLNs from 4T1-R models (*n* = 5 mice per group). Statistical significance was assessed using one-way ANOVA with Tukey's multiple-comparisons test (**c, d**). Data are presented as mean ± s.e.m. Source data are provided as a Source Data file.

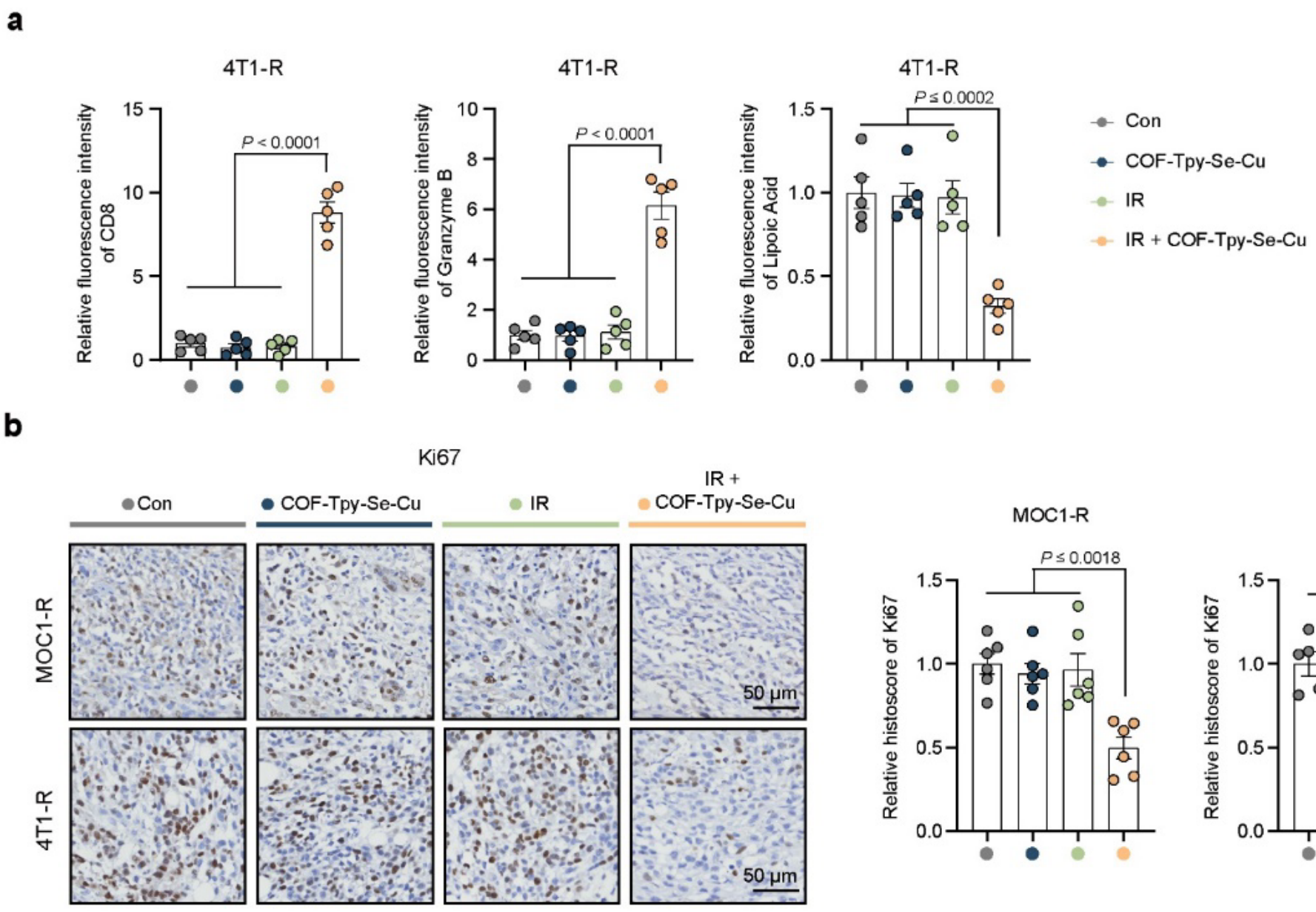

Supplementary Fig. 21. Multiplex immunofluorescence and immunohistochemical analysis of immune activation, cuproptosis hallmarks, and tumor proliferation in vivo. a Quantification of relative fluorescence intensity of CD8, granzyme B, and lipoic acid in 4T1-R tumor sections across the indicated treatment groups, analyzed by multiplex immunohistochemistry (mIHC) (n = 5 tumor samples per group). **b** Representative immunohistochemistry staining images and quantitative histoscore analysis of the proliferation marker Ki67 in MOC1-R (n = 6 tumor samples per group) and 4T1-R (n = 5 tumor samples per group) tumor sections from each treatment group (scale bar, 50 μm). Each point represents an individual tumor. Statistical significance was assessed using one-way ANOVA with Tukey's multiple-comparisons test (**a, b**). Data are presented as mean ± s.e.m. Source data are provided as a Source Data file.

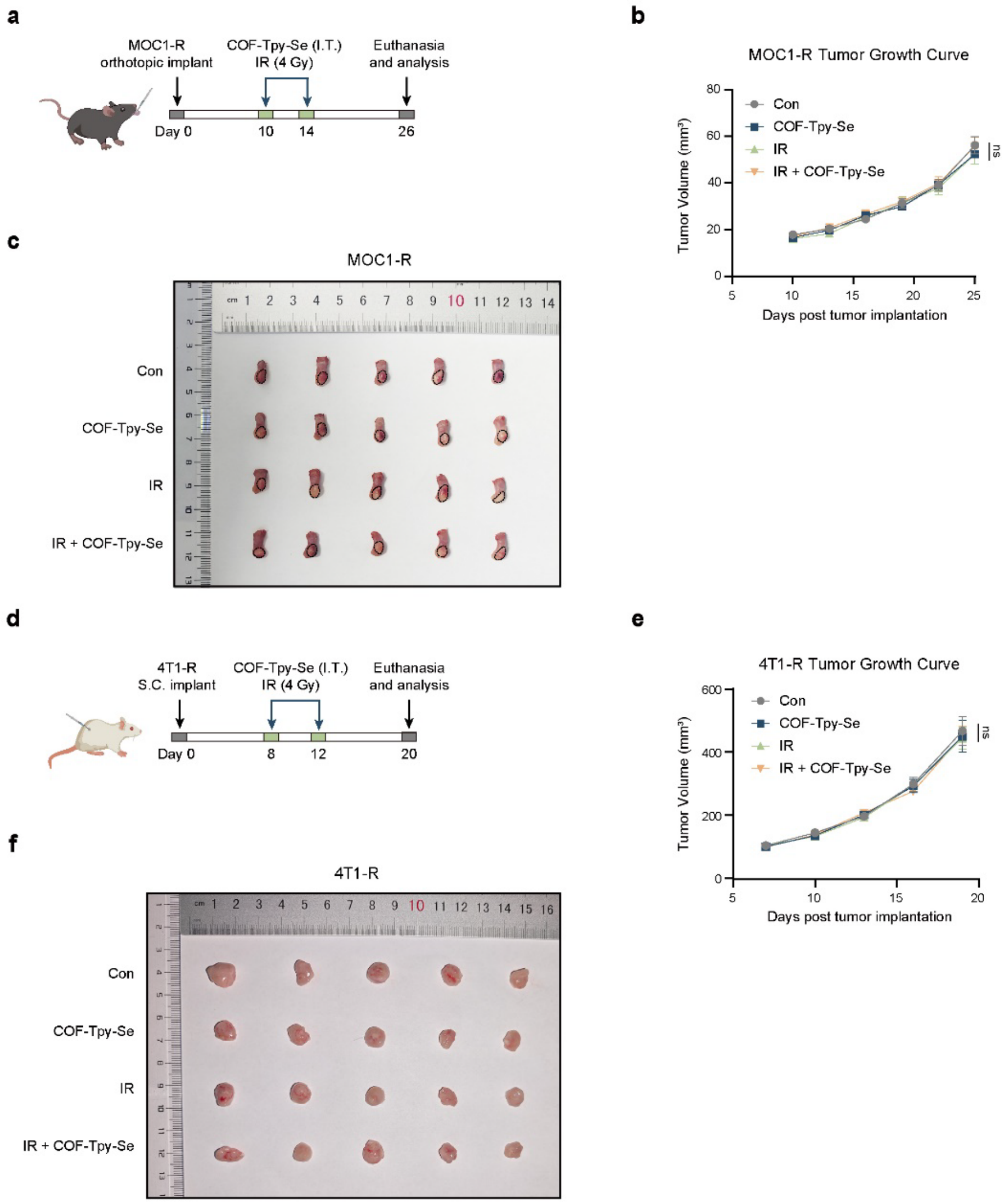

Supplementary Fig. 22. The copper-free COF-Tpy-Se scaffold does not significantly affect tumor growth under the tested conditions. a Experimental scheme for tumor implantation and treatment in the MOC1-R models. Mice received intratumoral (i.t.) COF-Tpy-Se (2 mg kg^{-1}), followed by localized irradiation (IR, 4 Gy). **b** Tumor growth curves for MOC1-R tumors across the indicated treatment groups (*n* = 5 mice per group). **c** Representative images of excised tongues from mice bearing orthotopic MOC1-R tumors at the study endpoint. Tumor regions are delineated by black dashed lines. **d** Experimental scheme for tumor implantation and treatment in the 4T1-R models. Mice received intratumoral (i.t.) COF-Tpy-Se (5 mg kg^{-1}), followed by localized irradiation (IR, 4 Gy). **e** Tumor growth curves for 4T1-R tumors across the indicated treatment groups (*n* = 5 mice per group). **f** Representative images of resected subcutaneous 4T1-R tumors from each treatment group at the endpoint. Statistical significance was assessed using two-way ANOVA (**b, e**). Data are presented as mean ± s.e.m. Source data are provided as a Source Data file.

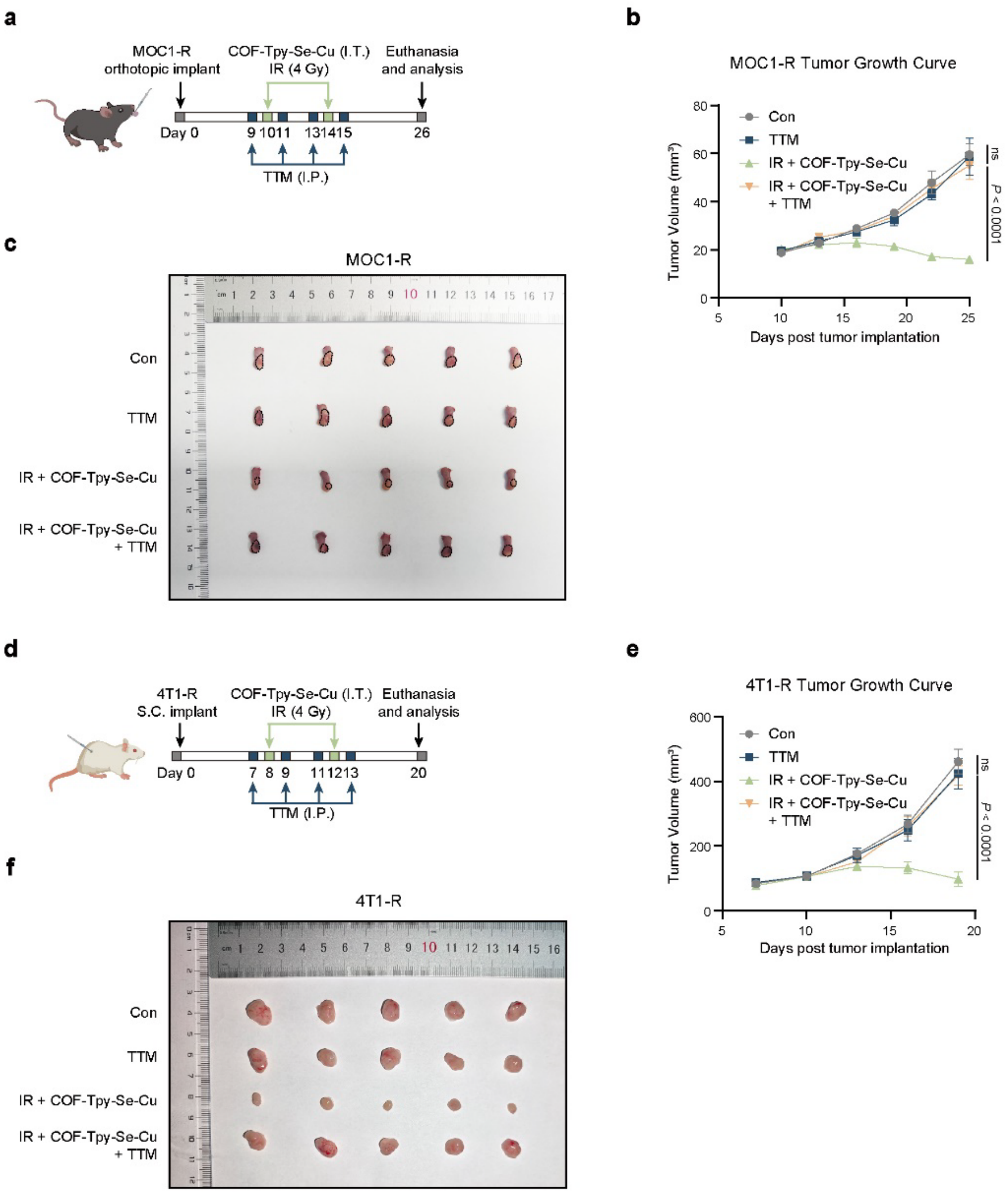

Supplementary Fig. 23. Copper chelation by TTM abrogates the in vivo radiosensitizing efficacy of COF-Tpy-Se-Cu. a Experimental schedule for the TTM rescue assay in the MOC1-R orthotopic tumor model. Mice received intratumoral (i.t.) injection of COF-Tpy-Se-Cu (2 mg kg^{-1}) and intraperitoneal (i.p.) administration of the copper chelator TTM (10 mg kg^{-1}), combined with localized irradiation (IR, 4 Gy) as indicated. **b** Tumor growth curves of MOC1-R orthotopic models across the indicated treatment groups (*n* = 5 mice per group). **c** Representative images of excised MOC1-R orthotopic tumors from each treatment group at the study endpoint. Tumor regions are delineated by black dashed lines. **d** Experimental schedule for the TTM rescue assay in the 4T1-R subcutaneous tumor model. Mice received intratumoral (i.t.) injection of COF-Tpy-Se-Cu (5 mg kg^{-1}) and intraperitoneal (i.p.) TTM (10 mg kg^{-1}), combined with localized irradiation (IR, 4 Gy) as indicated. **e** Tumor growth curves of 4T1-R subcutaneous models across the indicated treatment groups (*n* = 5 mice per group). **f** Representative images of excised 4T1-R subcutaneous tumors from each treatment group at the study endpoint. Statistical significance was assessed using two-way ANOVA (**b, e**). Data are presented as mean ± s.e.m. Source data are provided as a Source Data file.

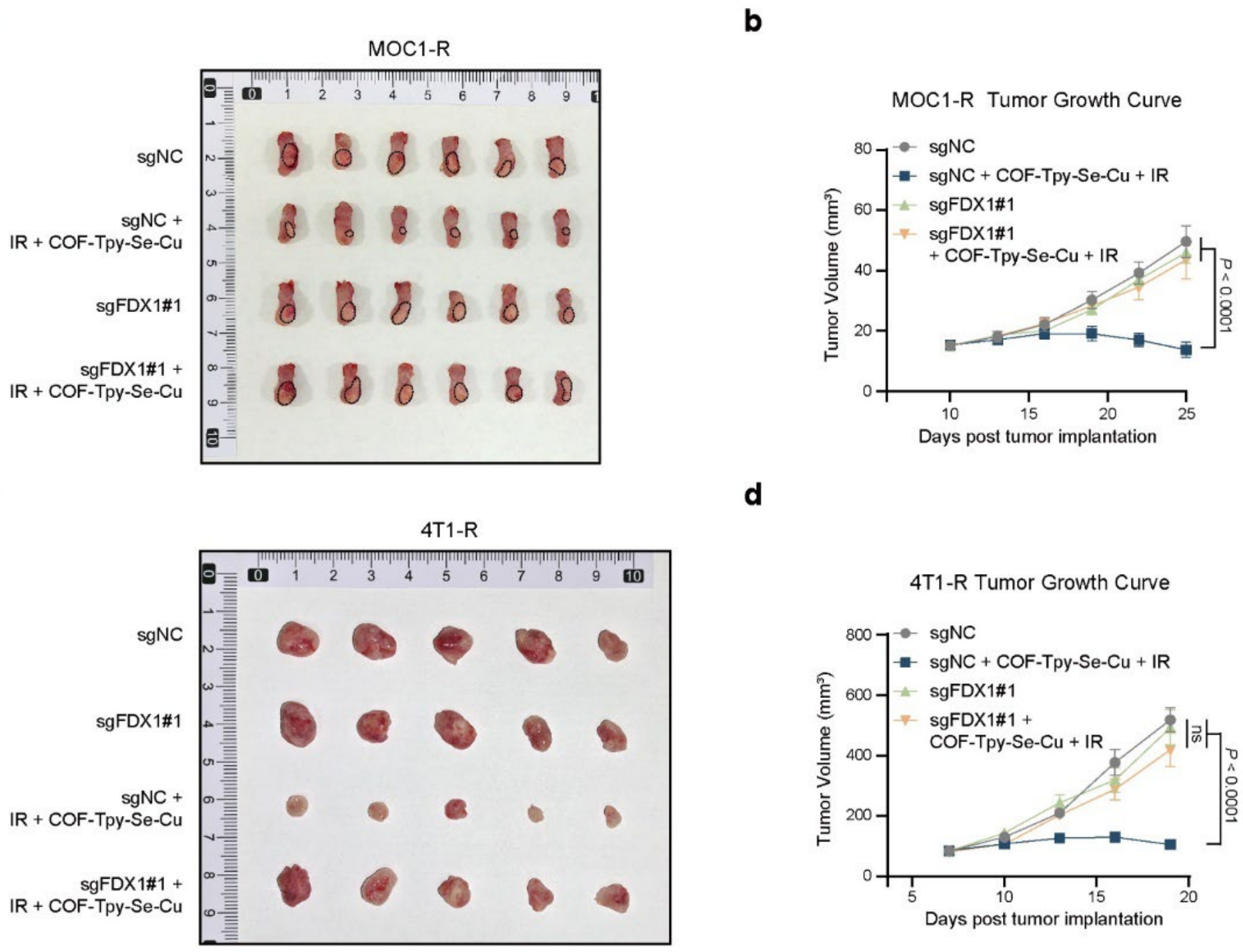

Supplementary Fig. 24. Genetic ablation of FDX1 attenuates the in vivo antitumor activity of COF-Tpy-Se-Cu plus irradiation. a Representative images of excised tongues from mice bearing orthotopic MOC1-R tumors at the study endpoint (n = 6 mice per group). Tumor regions are outlined with black dashed lines. **b** Tumor growth curves for MOC1-R tumor-bearing mice under the indicated treatment regimens. **c** Representative images of resected subcutaneous 4T1-R tumors from each treatment group at the endpoint (n = 5 mice per group). **d** Tumor growth curves for 4T1-R tumor-bearing mice under the indicated treatment regimens. Statistical significance was assessed using two-way ANOVA (**b, d**). Data are presented as mean ± s.e.m. Source data are provided as a Source Data file.

a

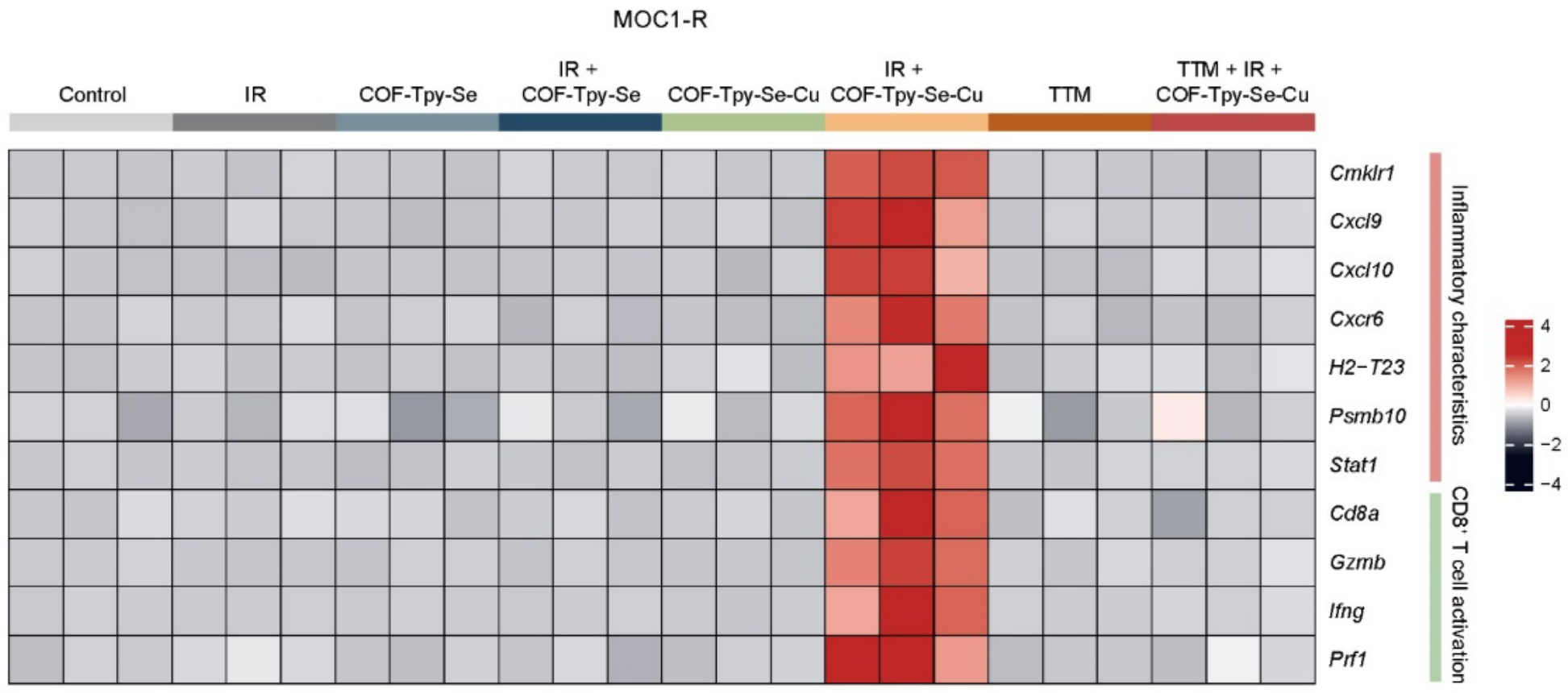

b

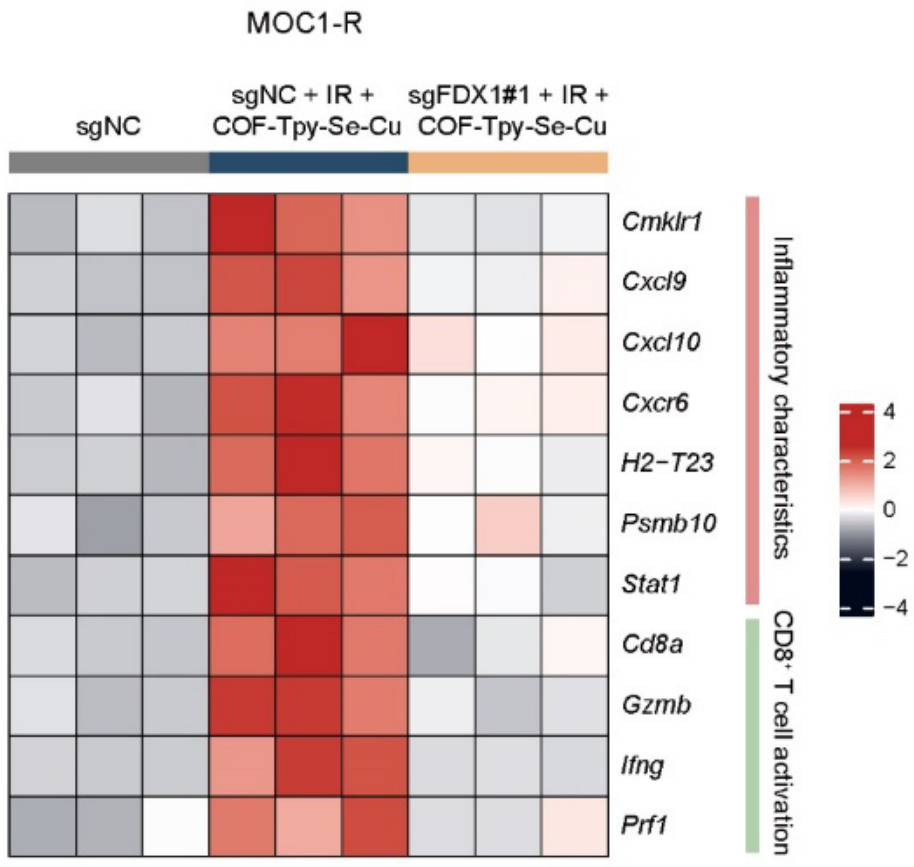

Supplementary Fig. 25. Heatmap summarizing expression of selected genes associated with inflammatory signatures and CD8^{+} T cell activation quantified by quantitative real-time PCR (qPCR) across treatment groups. For a and b, *n* = 3 independent experiments per group. Source data are provided as a Source Data file.

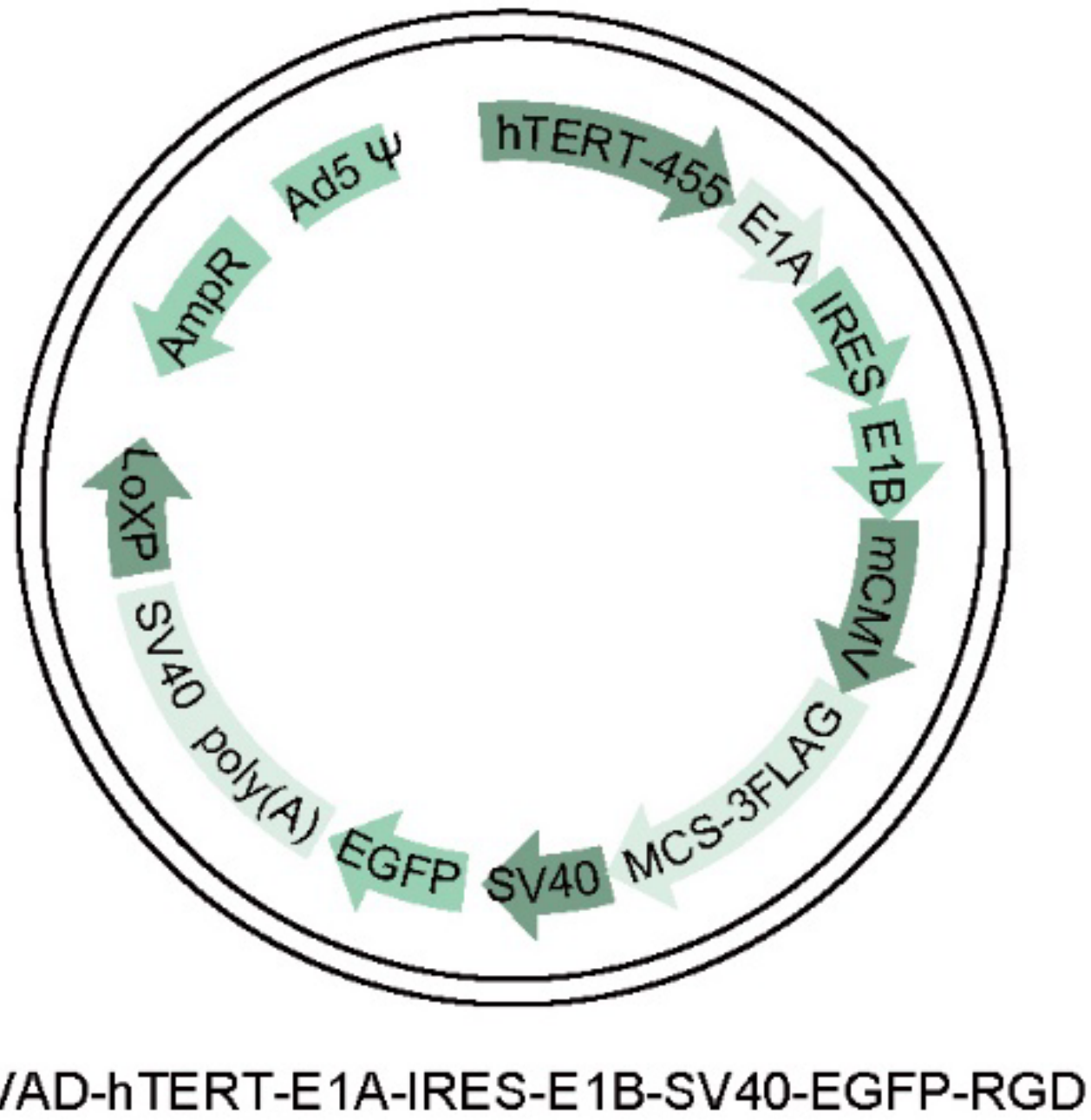

Supplementary Fig. 26. Schematic of the engineered oncolytic adenovirus vector OVAD-hTERT-E1A-IRES-E1B-SV40-EGFP-RGD (OVAD184-2282).

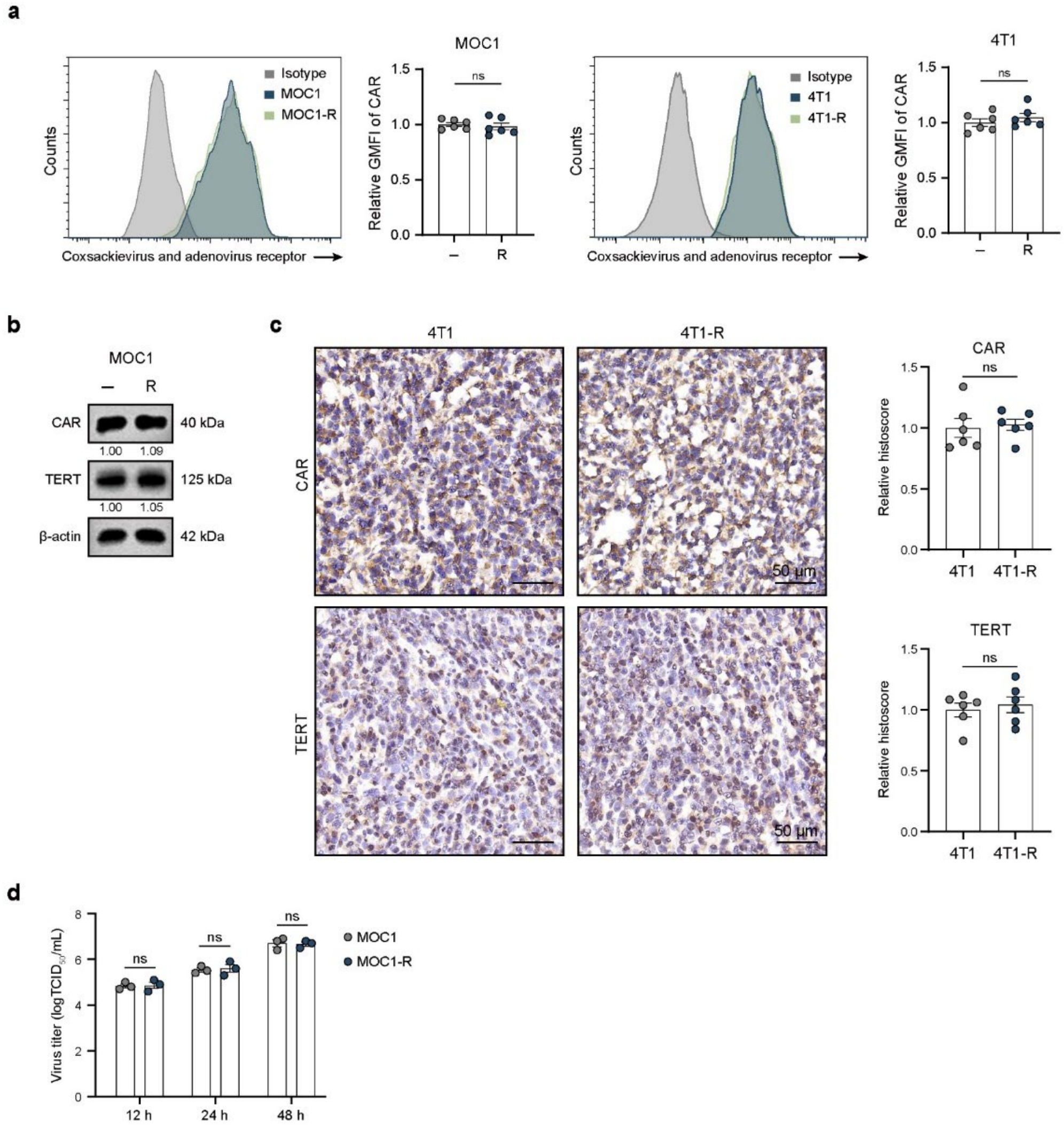

Supplementary Fig. 27. Radioresistance does not alter oncolytic adenovirus receptor expression or viral replication kinetics. a Flow cytometric analysis of coxsackievirus and adenovirus receptor (CAR) expression in parental and radioresistant cells. Isotype antibody was used as the negative control (*n* = 6 independent experiments). **b** Immunoblot analysis of CAR and telomerase reverse transcriptase (TERT) protein levels in MOC1 and MOC1-R cells. β-actin served as the loading control, with relative gray values labeled below each band (representative of three independent experiments). **c** Immunohistochemical staining of CAR and TERT in 4T1 and 4T1-R tumor tissues, with quantitative analysis of relative histoscore on the right (*n* = 6 independent tumor samples) (scale bar, 50 μm). **d** Replication kinetics of OVAD in MOC1 and MOC1-R cells infected at an MOI of 1. Viral titers were determined by $TCID_{50}$ assay at the indicated time points post-infection (*n* = 3 independent experiments). Statistical significance was assessed using two-tailed Student's *t* test (**a, c, d**). Data are presented as mean ± s.e.m. Source data are provided as a Source Data file.

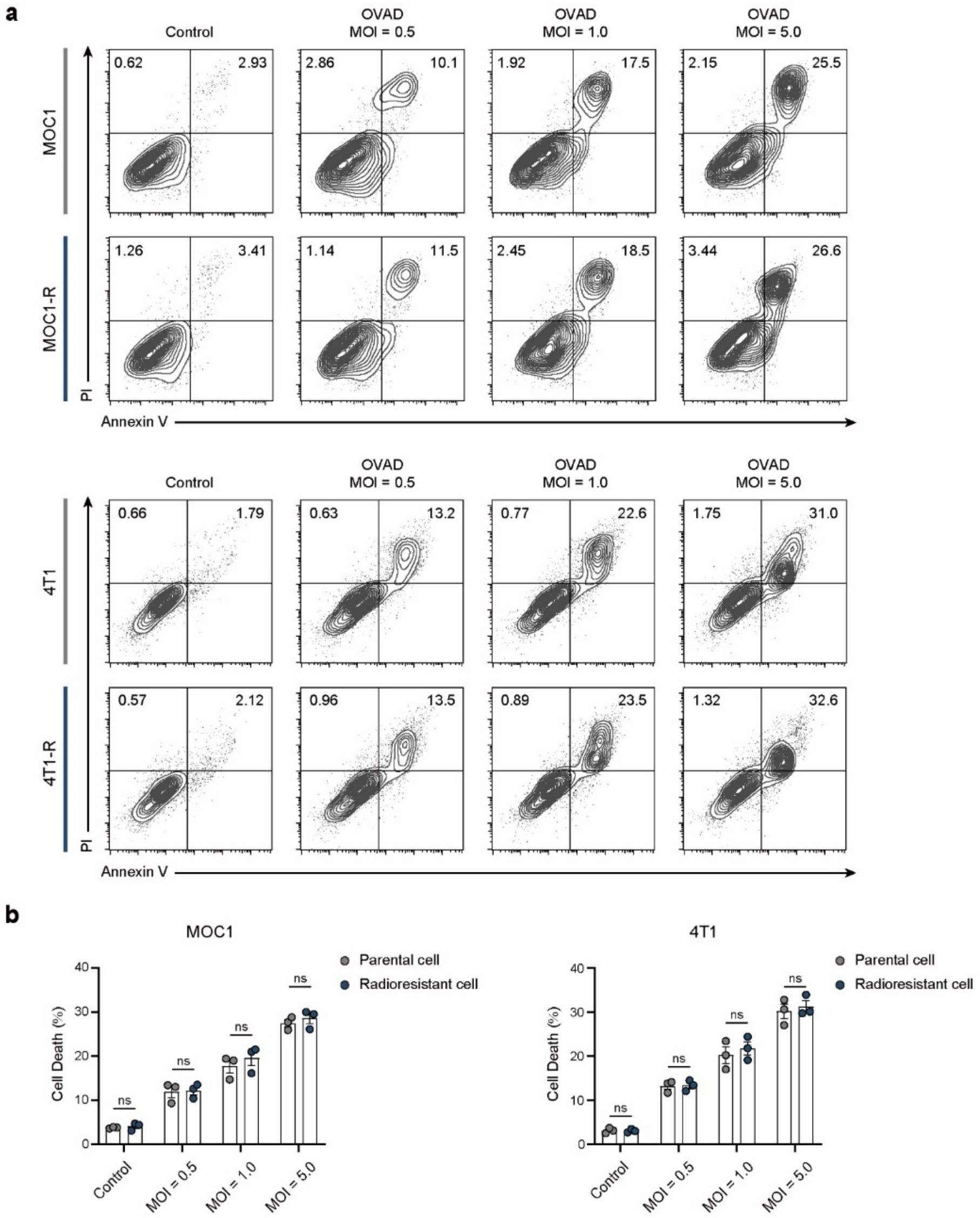

Supplementary Fig. 28. Oncolytic adenovirus exerts comparable cytotoxicity in parental and radioresistant tumor cells. a Flow cytometric analysis of Annexin V/PI staining in MOC1 and 4T1 parental and radioresistant cells at 24 h after OVAD infection at the indicated multiplicities of infection (MOIs) (*n* = 3 independent experiments). **b** Quantification of cell death, expressed as the percentage of PI-positive cells (*n* = 3 independent experiments). Statistical significance was assessed using two-tailed Student's *t* test (**b**). Data are presented as mean ± s.e.m. Source data are provided as a Source Data file.

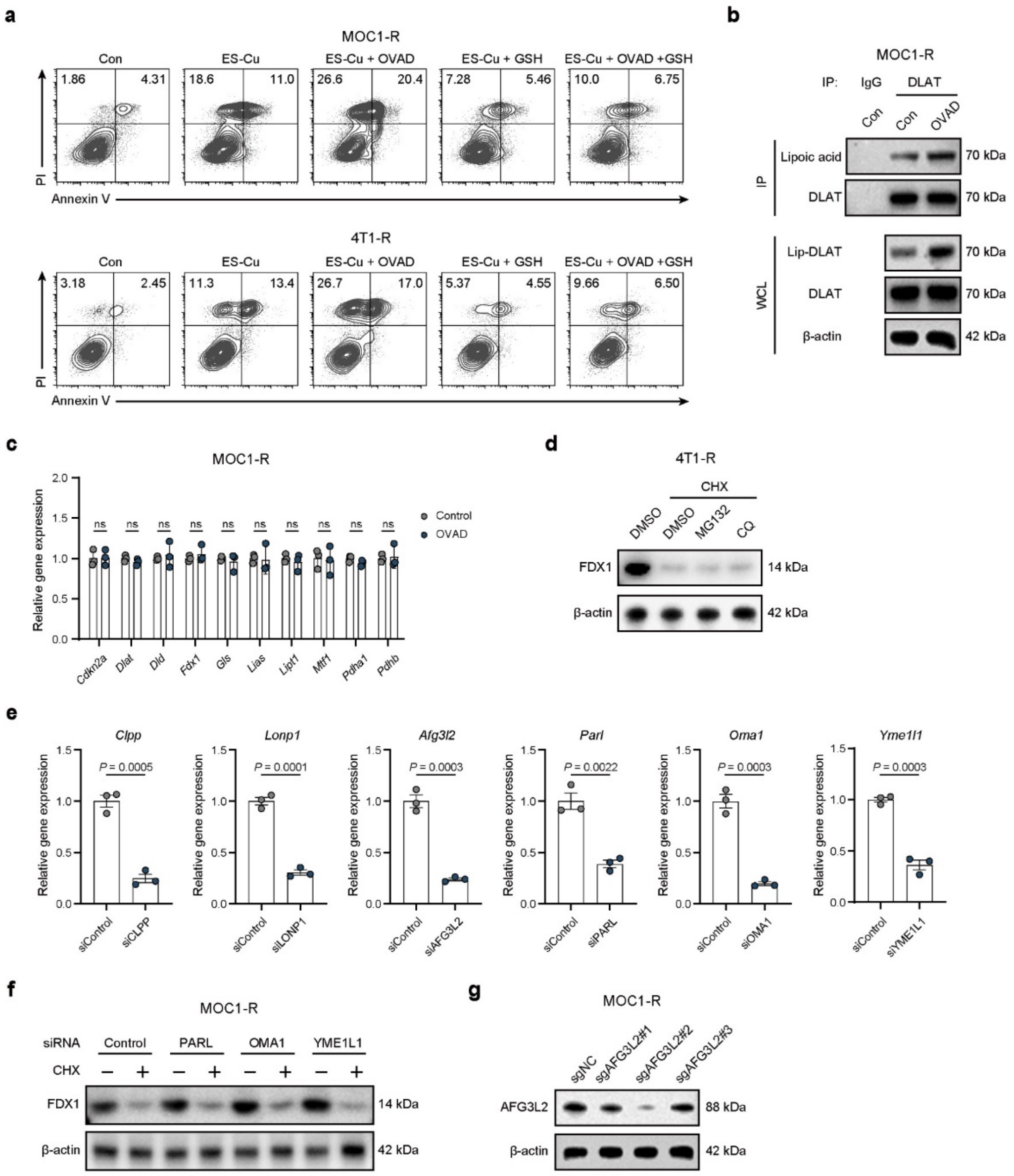

Supplementary Fig. 29. Oncolytic adenovirus modulates cuproptosis sensitivity in radioresistant tumor cells. a Flow cytometric analysis of Annexin V/PI staining in radioresistant MOC1-R (upper) and 4T1-R (lower) cells at 24 h after the indicated treatments (ES-Cu, 200 nM; OVAD, MOI = 1; GSH, 10 mM) (n = 3 independent experiments). **b** Co-immunoprecipitation (IP) analysis of DLAT lipoylation in MOC1-R cells with or without OVAD infection. DLAT was immunoprecipitated and immunoblotted with anti-lipoic acid antibody to detect lipoylated DLAT (Lip-DLAT), and with anti-DLAT antibody to confirm equal IP input. Whole-cell lysates (WCL) were analyzed in parallel. β-actin served as the loading control (representative of three independent experiments). **c** qPCR analysis of cuproptosis-associated gene expression in MOC1-R cells treated with vehicle or OVAD (MOI = 1) for 12 h (n = 3 independent experiments). **d** Immunoblot analysis of FDX1 in 4T1-R cells after 12 h treatment with DMSO, the proteasome inhibitor MG132 (10 μM), or the autophagy inhibitor chloroquine (CQ, 20 μM) (representative of three independent experiments). **e** Relative

gene expression of mitochondrial proteases and associated factors in siRNA-transfected MOC1-R cells (*n* = 3 independent experiments). **f** Immunoblot assessment of FDX1 in MOC1-R cells 48 h after transfection with siRNAs targeting candidate regulators of protein turnover (representative of three independent experiments). **g** Immunoblot validation of AFG3L2 knockout in MOC1-R cells transduced with a control sgRNA (sgNC) or three independent AFG3L2-targeting sgRNAs (sgAFG3L2#1/2/3). β-actin served as a loading control (representative of three independent experiments). Statistical significance was assessed using a two-tailed Student's *t* test (**c, e**). Data are presented as mean ± s.e.m. Source data are provided as a Source Data file.

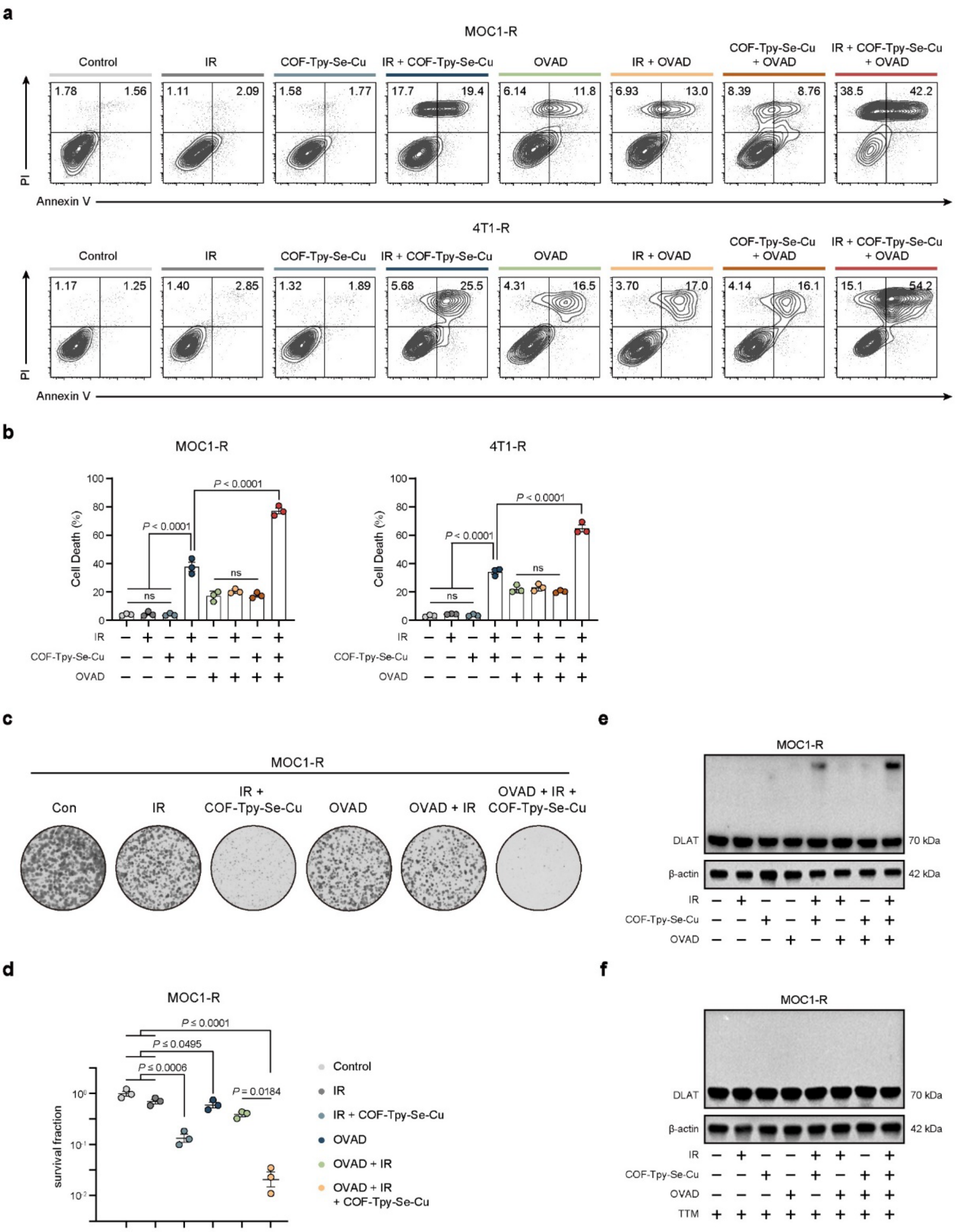

Supplementary Fig. 30. Synergistic radiosensitization efficacy of COF-Tpy-Se-Cu combined with an oncolytic adenovirus in vitro. a Flow cytometric analysis of Annexin V/PI staining in radioresistant MOC1-R (upper) and 4T1-R (lower) cells at 24 h after the indicated treatments (COF-Tpy-Se-Cu, 30 μg mL^{-1}; IR, 4 Gy; OVAD, MOI = 1) (n = 3 independent experiments). **b** Quantification of cell death, expressed as the percentage of PI-positive cells, in MOC1-R (left) and 4T1-R (right) cells (n = 3 independent experiments). **c** Representative images of crystal violet-stained colonies of MOC1-R cells following the indicated treatments (COF-Tpy-Se-Cu, 30 μg mL^{-1}; IR, 4 Gy; OVAD, MOI = 1) after 7 days (n = 3 independent experiments). **d** Quantification of survival fraction from clonogenic survival assays (n = 3 independent experiments).

e, f Immunoblot analysis of DLAT oligomerization in MOC1-R cells under the indicated treatments (COF-Tpy-Se-Cu, 30 μg mL^{-1}; IR, 4 Gy; OVAD, MOI = 1; TTM, 40 μM). β-actin served as the loading control (representative of three independent experiments). Statistical significance was assessed using one-way ANOVA with Tukey's multiple-comparisons test (**b, d**). Data are presented as mean ± s.e.m. Source data are provided as a Source Data file.

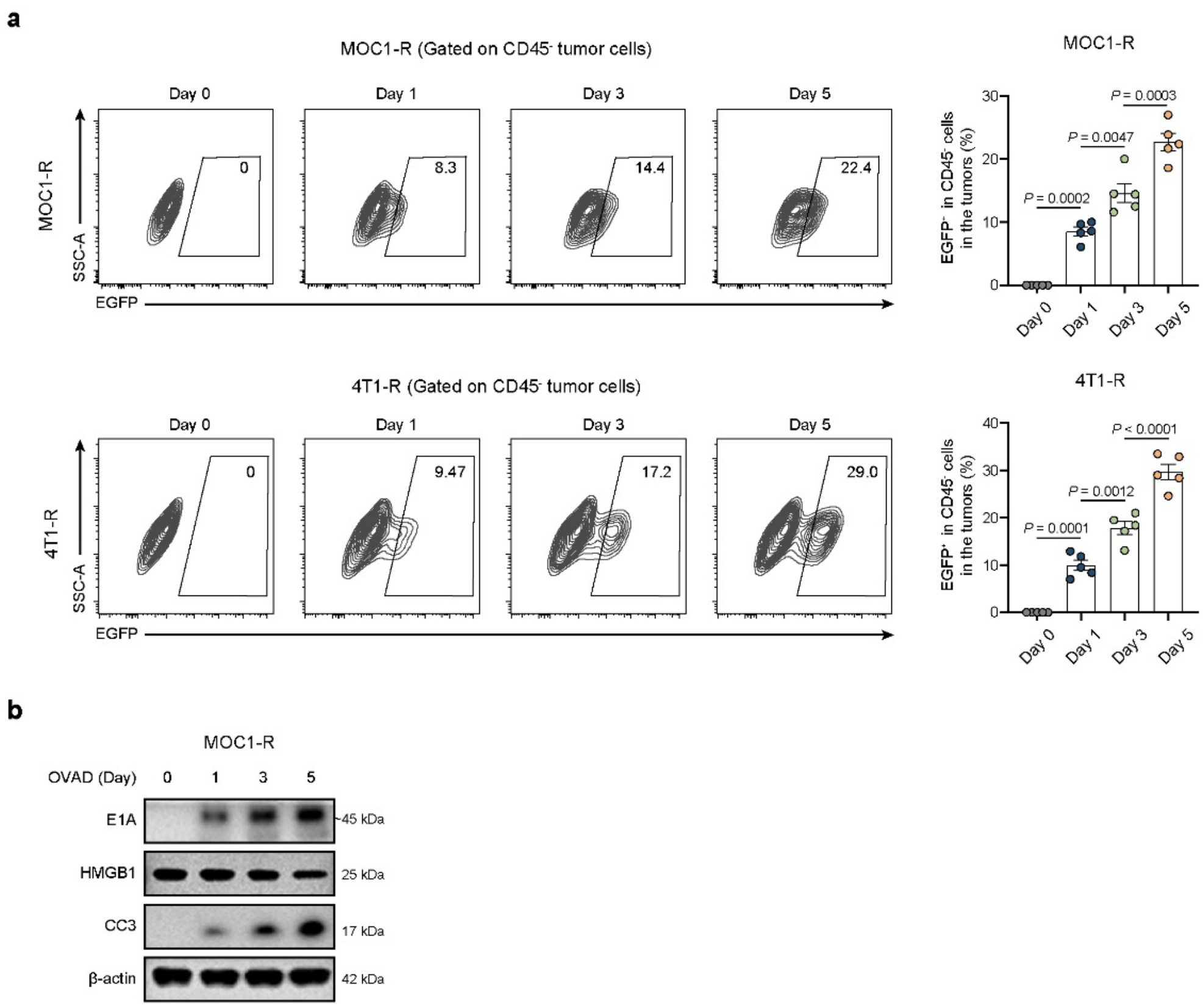

Supplementary Fig. 31. Intratumoral replication and cytotoxicity of oncolytic adenovirus in radioresistant tumor models. a Flow cytometric analysis of EGFP-positive cell frequency in MOC1-R (upper) and 4T1-R (lower) tumor tissues at the indicated days after intratumoral OVAD injection (0.4×10^6 PFU for MOC1-R; 2×10^6 PFU for 4T1-R) (n = 5 independent tumor samples). **b** Immunoblot analysis of viral E1A protein, HMGB1, and cleaved caspase-3 (CC3) in MOC1-R tumor lysates across sequential time points post OVAD administration. β-actin served as the loading control (representative of three independent experiments). Statistical significance was assessed using one-way ANOVA with Tukey's multiple-comparisons test (**a**). Data are presented as mean ± s.e.m. Source data are provided as a Source Data file.

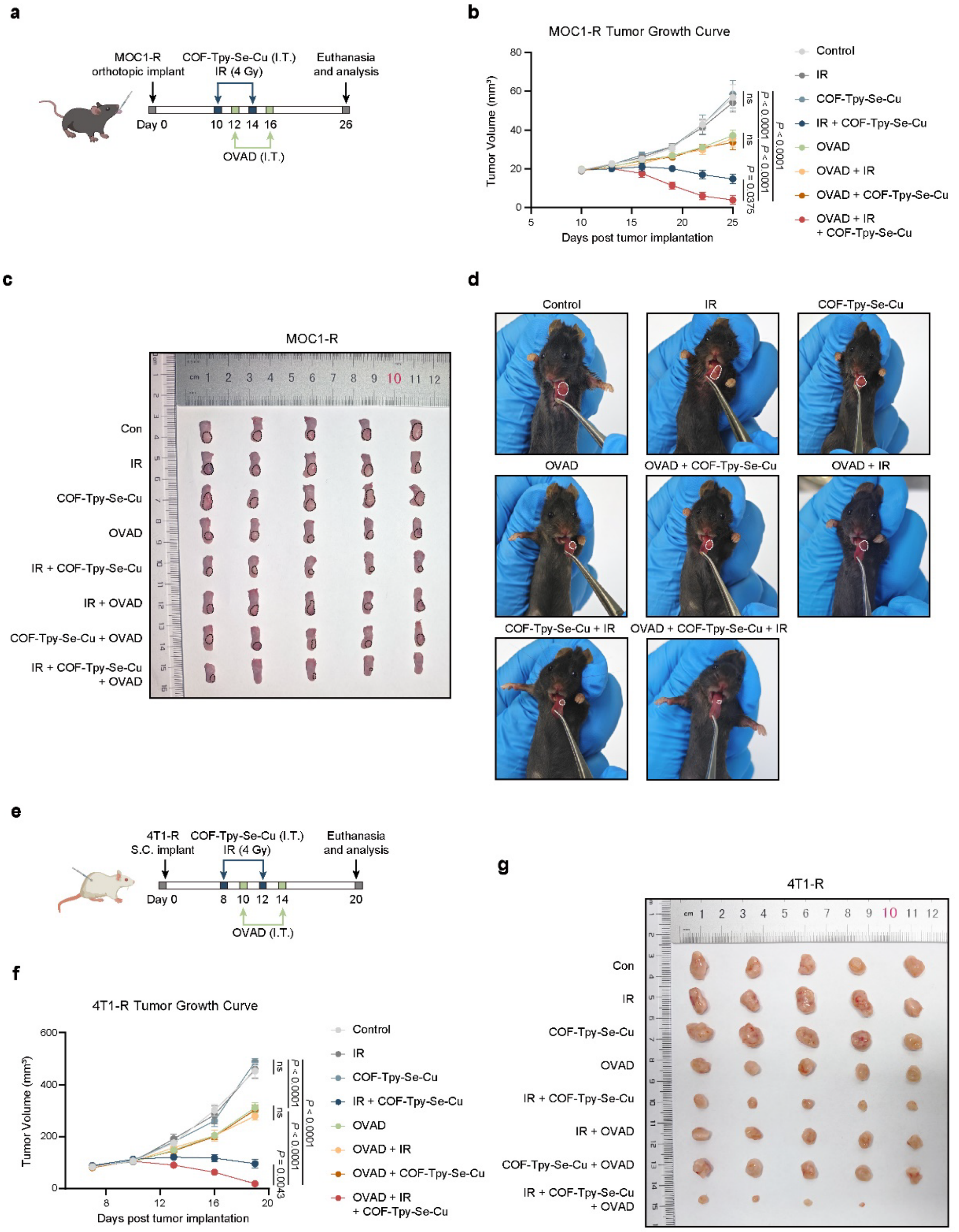

Supplementary Fig. 32. Synergistic radiosensitization efficacy of COF-Tpy-Se-Cu combined with oncolytic adenovirus in vivo. a In vivo treatment schedule. MOC1-R tumor-bearing mice received intratumoral injections of COF-Tpy-Se-Cu (2 mg kg^{-1}) and/or OVAD (0.4×10^6 PFU), combined with localized irradiation (IR, 4 Gy) as indicated. **b** Tumor growth curves for MOC1-R orthotopic tumors under the indicated treatments (n = 5 mice per group). **c** Representative images of excised tongues from mice bearing orthotopic MOC1-R tumors at the endpoint (n = 5 mice per group). Tumor boundaries are delineated by black dashed lines. **d** Representative images of mice bearing orthotopic MOC1-R tongue tumor across eight treatment groups. Tumor

boundaries are delineated with white dashed lines. **e** In vivo treatment schedule. 4T1-R tumor-bearing mice received intratumoral injections of COF-Tpy-Se-Cu (5 mg kg^{-1}) and/or OVAD (2×10^6 PFU), combined with localized irradiation (IR, 4 Gy) as indicated. **f** Tumor growth curves for 4T1-R models under the indicated treatments (*n* = 5 mice per group). **g** Representative images of excised subcutaneous 4T1-R tumors from each treatment group at the endpoint (*n* = 5 mice per group). Statistical significance was assessed using two-way ANOVA (**b, f**). Data are presented as mean ± s.e.m. Source data are provided as a Source Data file.

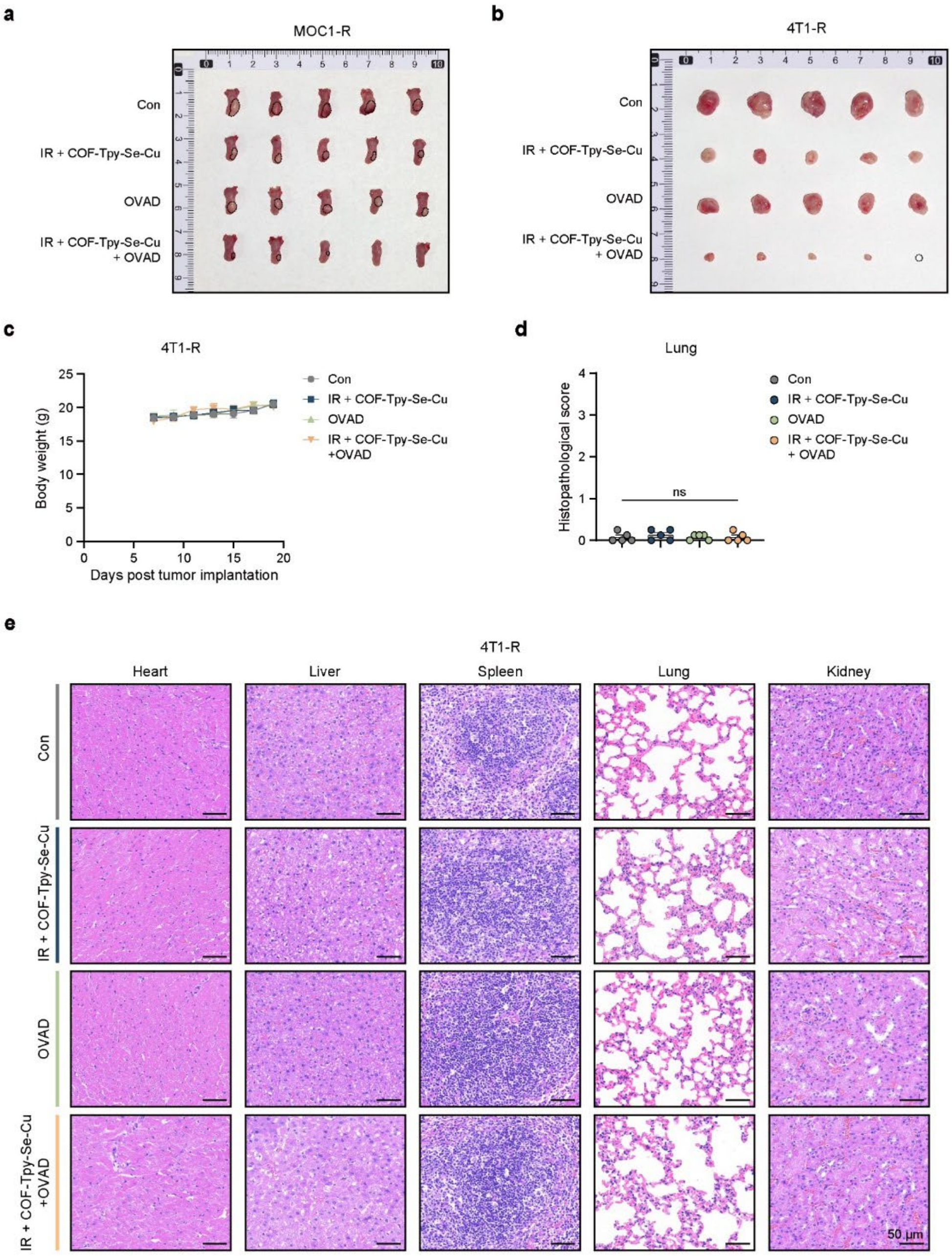

Supplementary Fig. 33. In vivo efficacy and safety evaluation. a Representative images of excised tongues from mice bearing orthotopic MOC1-R tumors at the endpoint (*n* = 5 mice per group). Tumor boundaries are delineated by black dashed lines. **b** Representative images of excised subcutaneous 4T1-R tumors from each treatment group at the endpoint (*n* = 5 mice per group). **c** Body-weight changes during the treatment period (*n* = 5 mice per group). **d** Blinded semi-quantitative histopathological scoring of H&E-stained lung tissue sections across the indicated treatment groups (*n* = 5 mice per group). **e** Representative H&E-stained sections of major organs (heart, liver, spleen, lung, and kidney) from treated and control BALB/c mice (scale bar, 50 μm). No overt histopathological abnormalities were observed in any treatment group relative to controls. Images are representative of five mice per group, with consistent findings

across all groups. Statistical significance was assessed using one-way ANOVA with Tukey's multiple-comparisons test (**c, d**). Data are presented as mean ± s.e.m. Source data are provided as a Source Data file.

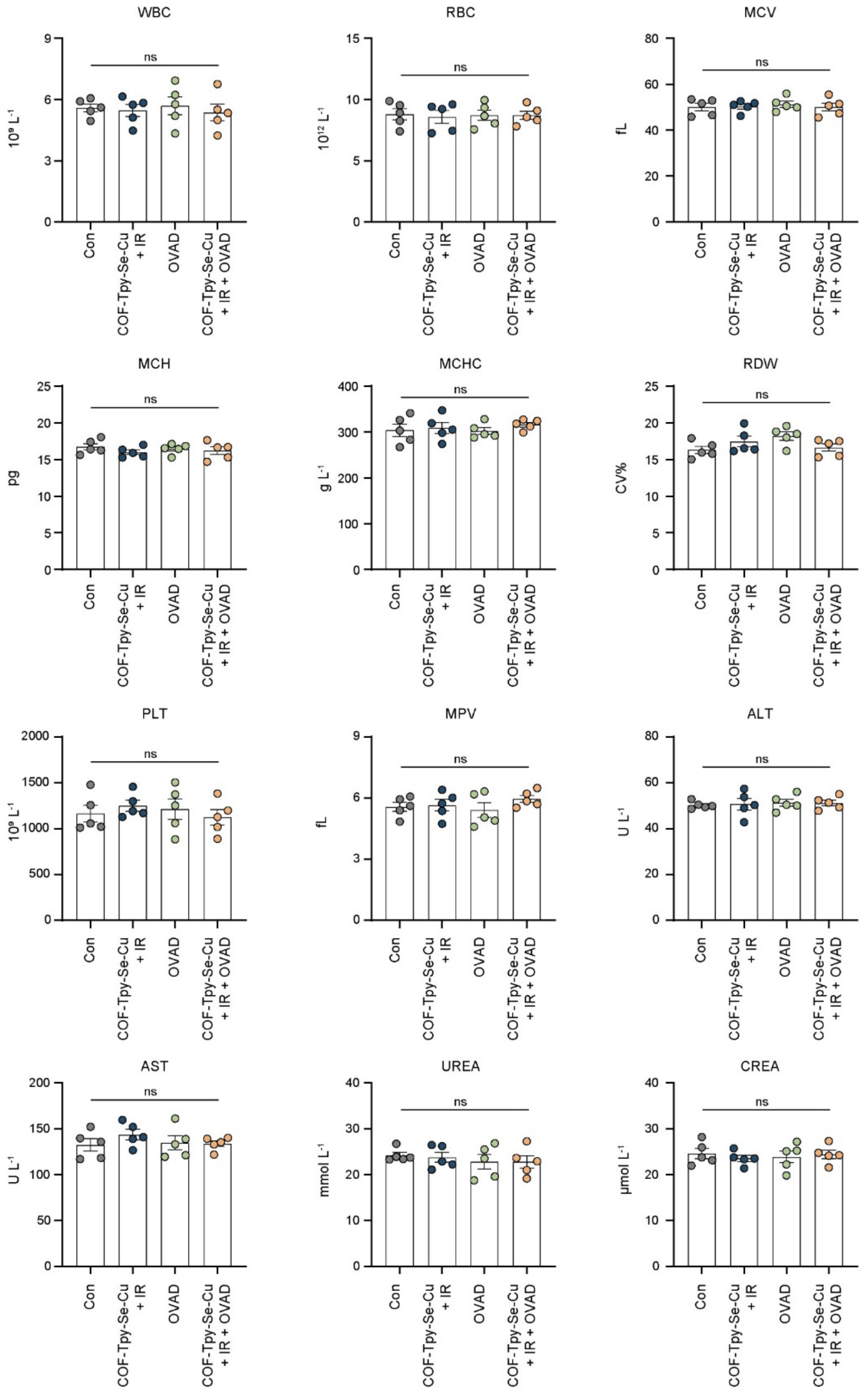

Supplementary Fig. 34. Routine blood test and blood biochemical test of mice in the control and treated groups. For all hematological and serum biochemical measurements, n = 5 mice per group. WBC: white blood cell; RBC: red blood cell; MCV: mean corpuscular volume; MCH: mean corpuscular hemoglobin; MCHC: mean corpuscular hemoglobin concentration; RDW: red cell distribution width; PLT: platelets; MPV: mean platelet volume; ALT: alanine aminotransferase; AST: aspartate aminotransferase; CREA: creatinine. Statistical significance was assessed using one-

way ANOVA with Tukey's multiple-comparisons test. Data are presented as mean ± s.e.m. Source data are provided as a Source Data file.

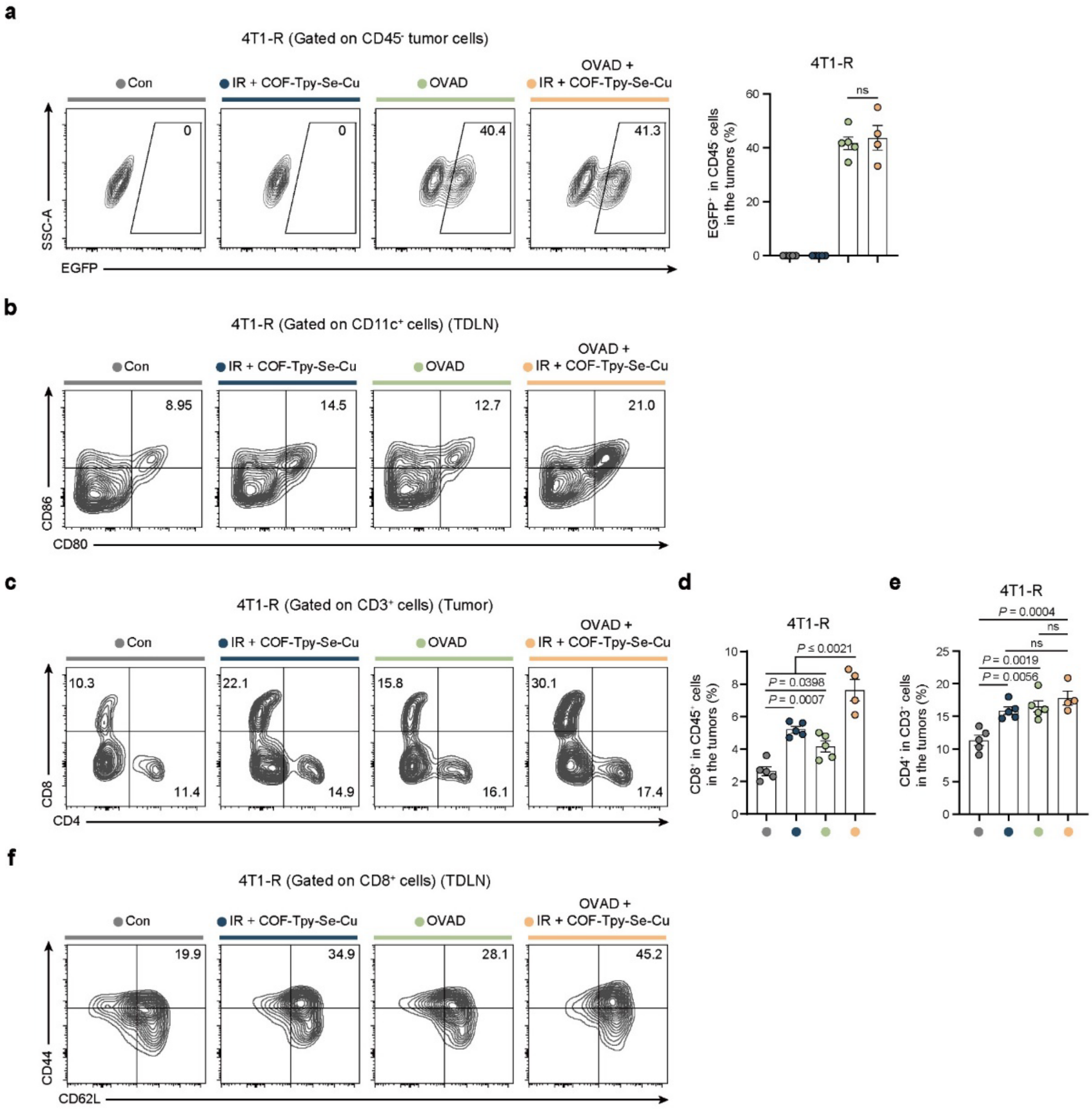

Supplementary Fig. 35. Combination therapy remodels the tumor immune microenvironment. **a** Flow cytometric assessment of intratumoral OVAD infection efficiency, quantified as the percentage of EGFP-positive cells among $CD45^-$ tumor cells in 4T1-R models across the indicated treatment groups (*n* = 5 mice per group, except that n = 4 tumors were analyzed in the triple-combination group because sufficient tumor tissue was unavailable from one mouse). **b** Representative plots showing the frequency of $CD80^+CD86^+$ cells among $CD11c^+$ DCs in tumor-draining lymph nodes (TDLNs) from 4T1-R models (*n* = 5 mice per group). **c** Flow cytometric analysis of tumor-infiltrating $CD8^+$ T and $CD4^+$ T cells among $CD3^+$ T cells from 4T1-R models (*n* = 5 mice per group). **d** Quantification of $CD8^+$ T cell frequency among $CD45^+$ leukocytes (*n* = 5 mice per group). **e** Quantification of $CD4^+$ T cell frequency among $CD3^+$ T cells (*n* = 5 mice per group). **f** Frequency of $CD44^+CD62L^+$ memory T cells among $CD8^+$ T cells in TDLNs from 4T1-R models (*n* = 5 mice per group). Statistical significance was assessed using one-way ANOVA with Tukey's multiple-comparisons test (**a, d, e**). Data are presented as mean ± s.e.m. Source data are provided as a Source Data file.

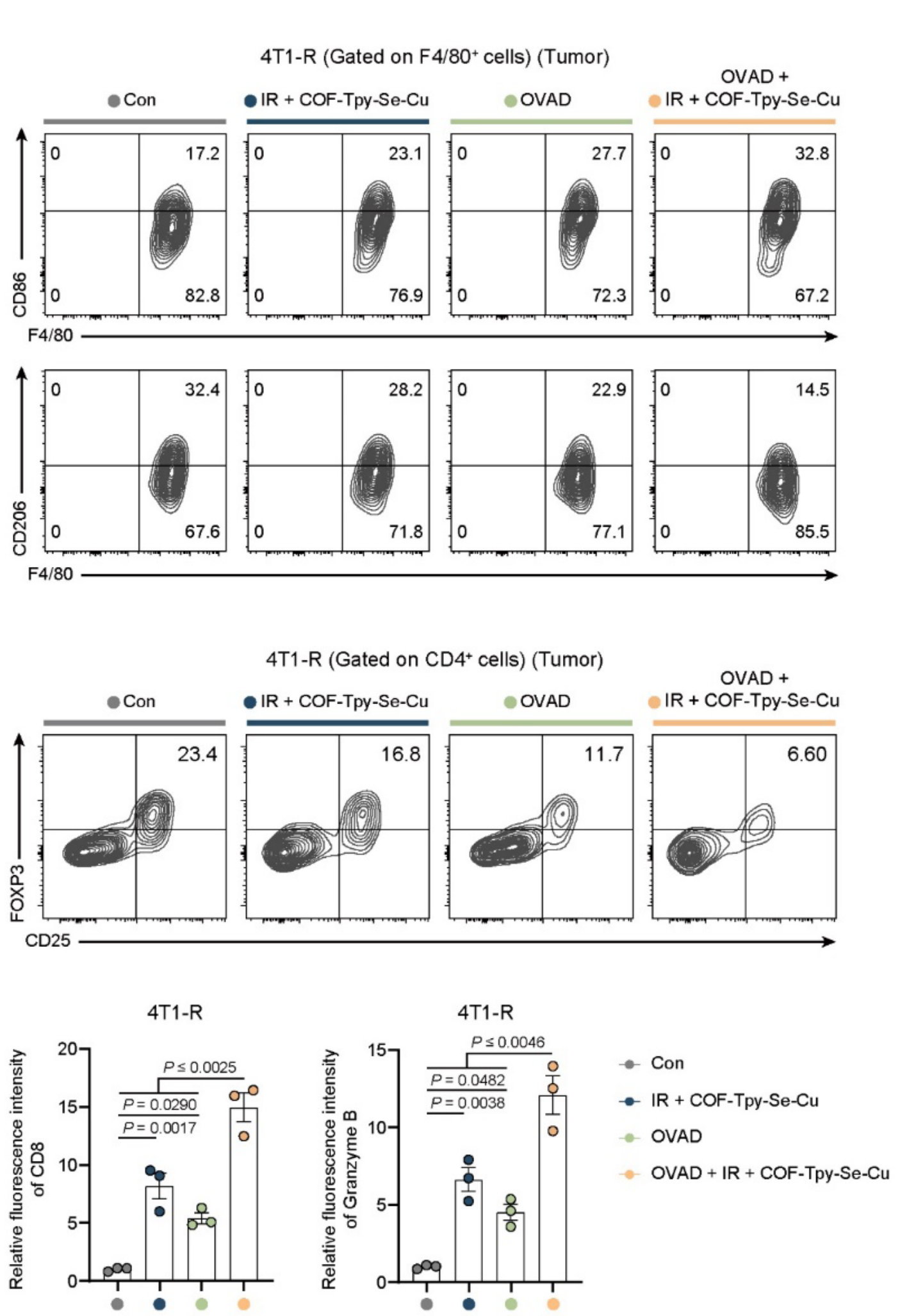

Supplementary Fig. 36. Combination therapy remodels the tumor immune microenvironment. a Representative flow cytometric plots of M1-like ($CD86^+$) and M2-like ($CD206^+$) tumor-associated macrophages (TAMs), gated on $F4/80^+$ cells, in 4T1-R tumor tissues across the indicated treatment groups (*n* = 5 mice per group, except that n = 4 tumors were analyzed in the triple-combination group because sufficient tumor tissue was unavailable from one mouse). **b** Representative flow cytometric plots of $CD25^+FOXP3^+$ regulatory T cells (Tregs) among $CD4^+$ cells in 4T1-R tumor tissues (*n* = 5 mice per group). **c** Quantification of relative fluorescence intensity of CD8 and granzyme B in 4T1-R tumor sections across the indicated treatment groups, analyzed by multiplex immunohistochemistry (mIHC) (*n* = 3 independent tumor samples). Statistical significance was assessed using one-way ANOVA with Tukey's multiple-comparisons test (**c**). Data are presented as mean ± s.e.m. Source data are provided as a Source Data file.

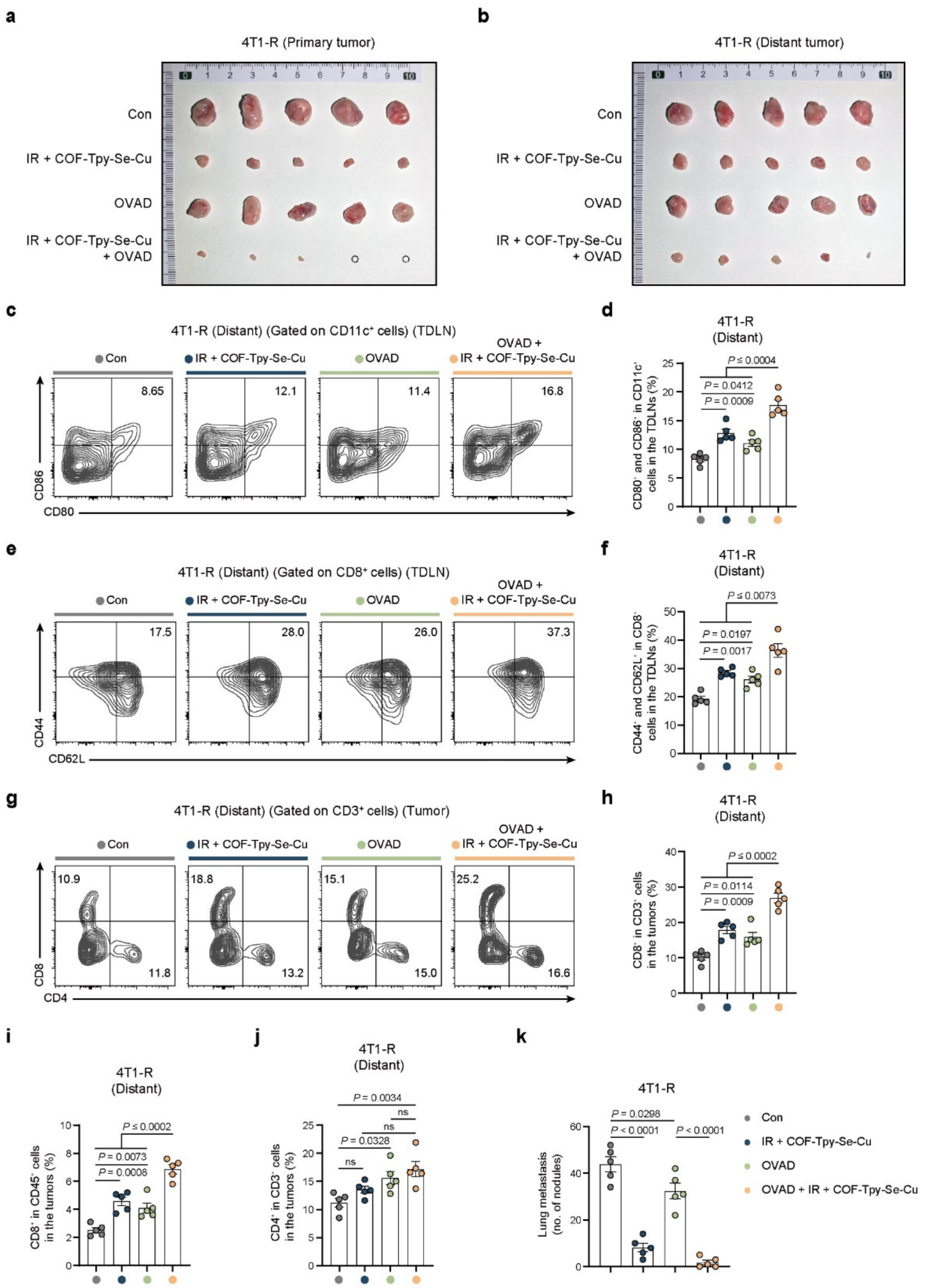

Supplementary Fig. 37. Combination therapy elicits an abscopal effect and systemic antitumor immunity. a Representative images of excised primary (ipsilateral, treated) tumors from 4T1-R–bearing mice under the indicated treatments (*n* = 5 mice per group). **b** Representative images of the corresponding distant (contralateral, untreated) tumors, demonstrating tumor rejection following combination therapy (*n* = 5 mice per group). **c, d** Flow cytometric analysis of $CD80^+CD86^+$ cells among $CD11c^+$ DCs in distant-tumor-draining lymph nodes (TDLNs) (*n* = 5 mice per group). **e, f** Flow cytometric analysis of $CD44^+CD62L^+$ memory T cells among $CD8^+$ T cells in

distant-tumor-draining lymph nodes (*n* = 5 mice per group). **g–j** Flow cytometric profiling of tumor-infiltrating T cells in distant tumors (*n* = 5 mice per group). **k** Quantification of pulmonary metastatic nodules across treatment groups (*n* = 5 mice per group). Statistical significance was assessed using one-way ANOVA with Tukey's multiple-comparisons test (**d, f, h, i, j, k**). Data are presented as mean ± s.e.m. Source data are provided as a Source Data file.

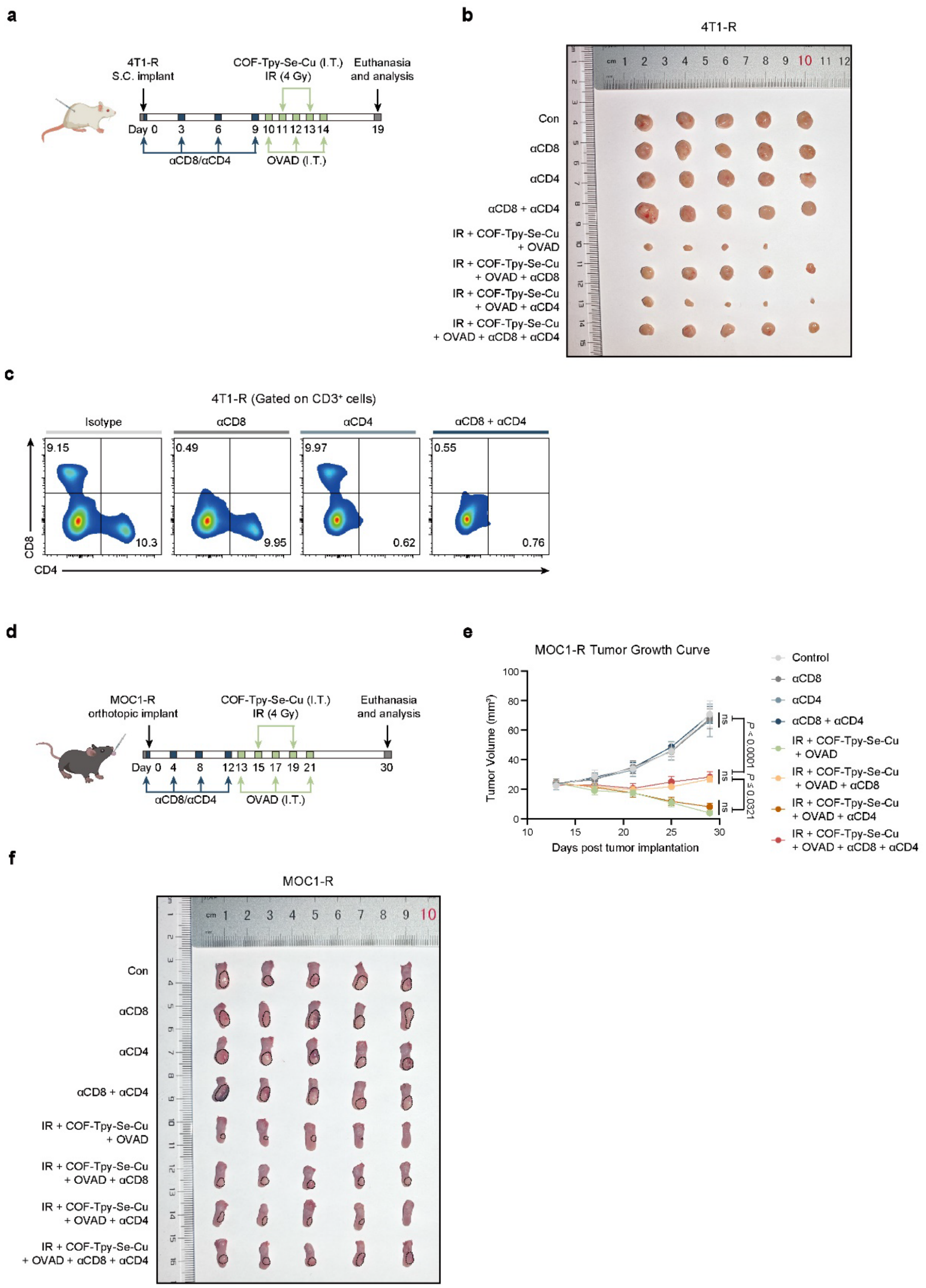

Supplementary Fig. 38. Antitumor efficacy of the combination regimen is dependent on CD8⁺ T cells in radioresistant tumor models. a Schematic of the T cell depletion experiment in the 4T1-R subcutaneous tumor model. Mice received intratumoral combination therapy with or without intraperitoneal injection of anti-CD8α antibody (10 mg kg^{-1}), anti-CD4 depleting antibody (10 mg kg^{-1}), the combination of both, or IgG isotype control antibody (10 mg kg^{-1}) at the indicated time

points. **b** Representative images of excised subcutaneous 4T1-R tumors from each treatment group at the endpoint (*n* = 5 mice per group). **c** Flow cytometric validation of $CD8^+$ and $CD4^+$ T cell depletion efficiency in 4T1-R tumor tissues (gated on $CD3^+$ T cells), with isotype antibody serving as the negative control. **d** Schematic of the T cell depletion experiment in the MOC1-R orthotopic tumor model. Mice received the combination treatment along with anti-CD8α (10 mg kg^{-1}, i.p.), anti-CD4 depleting antibody (10 mg kg^{-1}, i.p.), the combination of both, or IgG isotype control antibody (10 mg kg^{-1}, i.p.) at the indicated time points. **e** Tumor growth curves of MOC1-R orthotopic models across the indicated treatment and T cell depletion groups (*n* = 5 mice per group). **f** Representative images of excised tongues from mice bearing orthotopic MOC1-R tumors at the endpoint. Tumor boundaries are delineated by black dashed lines. Statistical significance of tumor growth curves was assessed by two-way ANOVA (**e**). Data are presented as mean ± s.e.m. Source data are provided as a Source Data file.

Supplementary Table 1. Sequence of primers used in this study.

Primers for qRT-PCR assay.

Gene	Primer	Sequence
Afg3l2	Forward	GAAGAACCGATAAGCTGCGGA
	Reverse	GCATATTGATAGAGCGTACGGA
Atf3	Forward	AGCGAAGACTGGAGCAAAATG
	Reverse	CATCCGATGGCAGAGGTGTT
Atox1	Forward	ATAAACTGAAGAGGCAGGCTGT
	Reverse	CTGAGCAGTTGGTGTTCCAGA
Atp7a	Forward	ACCACCATCGCATTTGCCTA
	Reverse	GGAGGTTTTGCCCTTCGCTA
Atp7b	Forward	TGCTGTCTGTGCAGAAGAGG
	Reverse	GCAAGTTTAGATAAGATCTGGGACC
Cd8a	Forward	CCGTTGACCCGCTTTCTGT
	Reverse	CGGCGTCCATTTTCTTTGGAA
Cd86	Forward	CAGCACGGACTTGAACAACC
	Reverse	CTCCACGGAAACAGCATCTGA
Cd206	Forward	TTCAGCTATTGGACGCGAGG
	Reverse	GAATCTGACACCCAGCGGAA
Cdkn2a	Forward	TGAATCTCCGCGAGGAAAGC
	Reverse	TGCCCATCATCATCACCTGAA
Clpp	Forward	GAATCATGATCCACCAGCCCT
	Reverse	CTCTCCATTGCTGACTCGAT
Cmklr1	Forward	ATGGAGTACGACGCTTACAACG
	Reverse	GGTGGCGATGACAATCACCA
Commd1	Forward	TCCAGTTATCCCACGCAGTTAC
	Reverse	ATGTCTGCAGATGCAATAGACTCC
Cox11	Forward	TCTGAGACGCCTCGGGACA
	Reverse	CTCCAAGTCCAGTAGTCTGGCA
Cox17	Forward	CGCTCATAGTTGCTTTCGCC
	Reverse	CAAAGTAGGCCACCACGTCA
Cxcl9	Forward	TCCTTTTGGGCATCATCTTCC
	Reverse	TTTGTAGTGGATCGTGCCTCG
Cxcl10	Forward	CCAAGTGCTGCCGTCATTTTC
	Reverse	GGCTCGCAGGGATGATTTCAA
Cxcr6	Forward	GAGTCAGCTCTGTACGATGGG
	Reverse	TCCTTGAACTTTAGGAAGCGTTT
Dlat	Forward	ACAAGGCCACCATAGGCTTT
	Reverse	TGGTCACTTCTGTTGGCCTG
Dld	Forward	AGTCTTACCAGGCGTTCAGC
	Reverse	TCACGTCAGCCTCAATTGGT
Fdx1	Forward	TCAAGAACCGAGATGGCGAG
	Reverse	GTTCCCTCACACGCACCAAA

Gls	Forward	CCGCGGGCGACAATAAAATA
	Reverse	AGCATGACACCATCTGACGTT
Gzmb	Forward	CCACTCTCGACCCTACATGG
	Reverse	GGCCCCCAAAGTGACATTTATT
H2-T23	Forward	ACAGTCCCGACCCAGAGTAG
	Reverse	CCACGTAGCCGACAATGATGA
Ifng	Forward	ATGAACGCTACACACTGCATC
	Reverse	CCATCCTTTTGCCAGTTCCTC
Lias	Forward	TCCCGGACAAGAGTACATGG
	Reverse	TGTGTCCCCCATCAACATGA
Lipt1	Forward	AACTGCGTCTAGAGGGCTGA
	Reverse	AAAGTGGAATTAGCATGCTGCC
Lonp1	Forward	GCCCTGACTGCAGAGATTGT
	Reverse	AGCCGCTTAAGGATGTTGGT
Mt1	Forward	AGATCTCGGAATGGACCCCA
	Reverse	AGGAGCAGCAGCTCTTCTTG
Mt2	Forward	TGTACTTCCTGCAAGAAAAGCTG
	Reverse	GTTGTGGAGAACGAGTCAGGG
Mtf1	Forward	AGTGACGCTAGGGACAGGT
	Reverse	AAAGGTAACGGCCTTTCTCCG
Nos2	Forward	TCTAGTGAAGCAAAGCCCAACA
	Reverse	TGATGGACCCCAAGCAAGAC
Oma1	Forward	TCGGGCTGTGGCTTCAATAA
	Reverse	TGCATTTACTGCTCGGAGCTT
Parl	Forward	ACAGGCTGTGCATTTGGGT
	Reverse	GCAGCTATGATGCCTGTCAC
Pdha1	Forward	TCACTGCGTTTTGGGCGTG
	Reverse	AGCCGATGAAGGTCACATTTCTTA
Pdhb	Forward	CTGTGTTGTCATTCGGCAGC
	Reverse	CACGAACTGTCAACTGCACC
Prf1	Forward	AGCACAAGTTCGTGCCAGG
	Reverse	GCGTCTCTCATTAGGGAGTTTTT
Psmb10	Forward	GGCTTGTGTTCCGAGATGGA
	Reverse	CTTCTCGCAGCTTTTGTCCG
Sco1	Forward	TCATGGCAGCACTTGTACGC
	Reverse	GACTTCCAAGAAACAGGCCCA
Slc11a2	Forward	AAAGATGCCAGACGATGGCG
	Reverse	TGCTGTAGGCAGGGTTGATG
Slc30a1	Forward	ACCAGGCAGAGCCAGAA
	Reverse	CAAGGACGTGCAGAAACACTC
Slc31a1	Forward	GGGCTTACCCTGTGAAGACTTT
	Reverse	GTGGTGAGGTGGCATGGTAA
Stat1	Forward	TCACAGTGGTTCGAGCTTCAG

	Reverse	GCAAACGAGACATCATAGGCA
Steap1	Forward	AAGCCAAAGGGAAACCTGGAA
	Reverse	TCTCTCCCGAGTCCTTAGTCGAG
Steap2	Forward	ATTGAGAACGCGTGGAACGA
	Reverse	GCCAAGCGTAGACTGGATGA
Tgfb1	Forward	ACGTCACTGGAGTTGTACGG
	Reverse	TGGGGCTGATCCCGTTGATT
Tnf	Forward	ACCCTCACACTCACAAACCA
	Reverse	ATAGCAAATCGGCTGACGGT
Yme1l1	Forward	ACGACGTTTGCAGTCTACCT
	Reverse	CCGCAAAAGAAACCCCTTCAC
β-actin	Forward	CATTGCTGACAGGATGCAGAAGG
	Reverse	TGCTGGAAGGTGGACAGTGAGG

Primers for PCR after ChIP assay.

Cox17 promoter		
-2000~-1750bp	Forward	GGGTCTGCTCATAGATTCCAAAGGG
	Reverse	ACATCACTCTGAACTTCCTGAAAGGC
-1750~-1500bp	Forward	CAAGCCAGGAACTTTCCTTCACAAAG
	Reverse	CTCACCTGTATTTACTGGCGCTACTG
-1500~-1250bp	Forward	GCCCTTCTGAACCTCCGTTT
	Reverse	TCAGCATCACAGCTTCCCTG
-1250~-1000bp	Forward	CCAGTTTAGGAGCTGAGGGT
	Reverse	TGTGTGCCATCCTTTCCTGG
-1000~-750bp	Forward	TGTGAAGAAATGGCTCACTGG
	Reverse	CAGCATTGAGTGGCTTCCTT
-750~-500bp	Forward	TGGTTGATTTGAAGCAATGGAG
	Reverse	TCAACAACTGTGATGGGCTGT
-500~-250bp	Forward	CATATATGCCGGAGAGAAATAGGA
	Reverse	TTCCTATATGACCCATTCAGTCCA
-250~0bp	Forward	GGGAGGAAAAGGCCATGAATTTGAAG
	Reverse	GCGCAAAAGAAGTGGTACAAGGC

Fdx1 promoter		
-2000~-1750bp	Forward	AGCAAGTCTGCAATGATTCCATGTTG
	Reverse	GGCATTTAGAATAAGCTGGTGTTCCC
-1750~-1500bp	Forward	ATACACACCCTGCCAAGCAA
	Reverse	ACCTTAGTCTTTGGGCTAACATCA
-1500~-1250bp	Forward	CCCTGCAGGTTCAATATCCA
	Reverse	TAAGGACATTTGCCGCTCGT
-1250~-1000bp	Forward	GTAGGTACGTGTATGCCACATGTCAG
	Reverse	ACACATACATTTGGGAGGAGAAAGGG
-1000~-750bp	Forward	CGAGGGGCCAGGAGAAAGTTTTG

	Reverse	CAGAAGCACATGTTGCAAGCGAAC
-750~-500bp	Forward	GCACCTTATCAGGAATGCTCAA
	Reverse	GGTCAGATACGCAACCTCCA
-500~-250bp	Forward	CGGCTTTCTAGTTTTGAAGTCTGT
	Reverse	GCAGGTAATAACGGTCAGCCT
-250~0bp	Forward	GGTGGCTTTTGGTCAGTCC
	Reverse	CGAGCACTGTTCCAGGGATC

Supplementary Table 2. Sequence of sgRNAs used in this study.

Fdx1 sgRNA#1	Forward	CACCgTGCGCCTCCGTCCCTTTCCG
	Reverse	AAACCGGAAAGGGACGGAGGCGCAC
Fdx1 sgRNA#2	Forward	CACCgTGGCGAGACGCTAACGACCA
	Reverse	AAACTGGTCGTTAGCGTCTCGCCAC
Fdx1 sgRNA#3	Forward	CACCgCGGGAACTGCCATCTCTCCG
	Reverse	AAACCGGAGAGATGGCAGTTCCCGC
Atf3 sgRNA#1	Forward	CACCgTCAAATACCAGTGACCCAGG
	Reverse	AAACCCTGGGTCACTGGTATTTGAC
Atf3 sgRNA#2	Forward	CACCgCCAGCGCAGAGGACATCCGA
	Reverse	AAACTCGGATGTCCTCTGCGCTGGC
Afg3l2 sgRNA#1	Forward	CACCgAAAAGCGATAATGAGTACGG
	Reverse	AAACCCGTACTCATTATCGCTTTTC
Afg3l2 sgRNA#2	Forward	CACCgTTCGTGTGACCTTTACACC
	Reverse	AAACGGTGTAAAGGTCACACGAAC
Afg3l2 sgRNA#3	Forward	CACCgTGAAGTTTAAAGATGTGGC
	Reverse	AAACGCCACATCTTTAAACTTCAC

Supplementary Table 3. Sequence of siRNAs used in this study.

Afg3l2 siRNA	ACCGGGTCCCTGTGGTTTATA
Clpp siRNA	GCCCAATTCCAGAATCATGAT
Lonp1 siRNA	CGGAGACAAGTTGCGAATGAT
Parl siRNA	GCTGTCAACGTTCAGTCATTT
Oma1 siRNA	CCCTAACAAGAAGGAGCTATT
Yme1l1 siRNA	GCAGTCTACCTCTGAAAGATT
