# Supplementary material for: A cuproptosis-inducing covalent organic framework synergizes with oncolytic virus for tumor radiotherapy
Source: Nat Commun. 2026 Aug 21;17:10011. doi: 10.1038/s41467-026-76947-w (PMC13590516; doi:10.1038/s41467-026-76947-w)
Supplement: Supplementary file 2 [file 41467_2026_76947_MOESM2_ESM.pdf]

nature portfolio

Corresponding author(s): Wei He

Last updated by author(s): Aug 11, 2026

Reporting Summary

Nature Portfolio wishes to improve the reproducibility of the work that we publish. This form provides structure for consistency and transparency in reporting. For further information on Nature Portfolio policies, see our Editorial Policies and the Editorial Policy Checklist.

Statistics

For all statistical analyses, confirm that the following items are present in the figure legend, table legend, main text, or Methods section.

n/a	Confirmed	
☐	☒	The exact sample size (n) for each experimental group/condition, given as a discrete number and unit of measurement
☐	☒	A statement on whether measurements were taken from distinct samples or whether the same sample was measured repeatedly
☐	☒	The statistical test(s) used AND whether they are one- or two-sided<br>*Only common tests should be described solely by name; describe more complex techniques in the Methods section.*
☐	☒	A description of all covariates tested
☐	☒	A description of any assumptions or corrections, such as tests of normality and adjustment for multiple comparisons
☐	☒	A full description of the statistical parameters including central tendency (e.g. means) or other basic estimates (e.g. regression coefficient) AND variation (e.g. standard deviation) or associated estimates of uncertainty (e.g. confidence intervals)
☐	☒	For null hypothesis testing, the test statistic (e.g. F, t, r) with confidence intervals, effect sizes, degrees of freedom and P value noted<br>*Give P values as exact values whenever suitable.*
☒	☐	For Bayesian analysis, information on the choice of priors and Markov chain Monte Carlo settings
☒	☐	For hierarchical and complex designs, identification of the appropriate level for tests and full reporting of outcomes
☒	☐	Estimates of effect sizes (e.g. Cohen's d, Pearson's r), indicating how they were calculated

Our web collection on statistics for biologists contains articles on many of the points above.

Software and code

Policy information about availability of computer code

Data collection	CytExpert software v2.3 (Beckman), iBright FL1500 system (Thermo Fisher Scientific), Aperio ImageScope v12.3.2 (Leica), ABI QuantStudio Software v1.3 (Thermo Fisher Scientific), FV10-ASW v4.0 software (Olympus).
Data analysis	Softwares used for analysis include: GraphPad Prism v8.0.2 (GraphPad Software), Excel 2024 (Microsoft), FlowJo v10.0.6 (TreeStar), Aperio ImageScope v12.3.2 (Leica), CaseViewer v2.4 (3DHISTECH), ImageJ v1.8 (National Institutes of Health).

For manuscripts utilizing custom algorithms or software that are central to the research but not yet described in published literature, software must be made available to editors and reviewers. We strongly encourage code deposition in a community repository (e.g. GitHub). See the Nature Portfolio guidelines for submitting code & software for further information.

Data

Policy information about availability of data

All manuscripts must include a data availability statement. This statement should provide the following information, where applicable:

- Accession codes, unique identifiers, or web links for publicly available datasets
- A description of any restrictions on data availability
- For clinical datasets or third party data, please ensure that the statement adheres to our policy

The data generated in this study are available within the Article, Supplementary Information, or Source Data file. RNA-seq data have been deposited in the NCBI Gene Expression Omnibus (GEO) database under accession code [GSE343347](https://www.ncbi.nlm.nih.gov/geo/query/acc.cgi?acc=GSE343347) and [GSE343355](https://www.ncbi.nlm.nih.gov/geo/query/acc.cgi?acc=GSE343355). Source data are provided with this paper.

Research involving human participants, their data, or biological material

Policy information about studies with human participants or human data. See also policy information about sex, gender (identity/presentation), and sexual orientation and race, ethnicity and racism.

Reporting on sex and gender	This study does not involve.
Reporting on race, ethnicity, or other socially relevant groupings	This study does not involve.
Population characteristics	This study does not involve.
Recruitment	This study does not involve.
Ethics oversight	This study does not involve.

Note that full information on the approval of the study protocol must also be provided in the manuscript.

Field-specific reporting

Please select the one below that is the best fit for your research. If you are not sure, read the appropriate sections before making your selection.

[x] Life sciences [ ] Behavioural & social sciences [ ] Ecological, evolutionary & environmental sciences

For a reference copy of the document with all sections, see nature.com/documents/nr-reporting-summary-flat.pdf

Life sciences study design

All studies must disclose on these points even when the disclosure is negative.

Sample size	Although no sample size calculation was performed, sample sizes for the in vivo experiments are similar to those generally employed and accepted in the field (Nat Nanotechnol 14(1):89 (2019); Nat Biotechnol 37(11):1322 (2019)) and were sufficient to support our conclusions with statistical significance. Sample sizes for the in vitro experiments are also based on previous work (Nat Commun, 16(1):44 (2025)).
Data exclusions	No data were excluded.
Replication	The experiments were reproduced successfully, the number of experiments was listed in corresponding figure legends.
Randomization	The samples were allocated into experimental groups randomly.
Blinding	The investigators were blinded to group allocation during data collection and analysis.

Reporting for specific materials, systems and methods

We require information from authors about some types of materials, experimental systems and methods used in many studies. Here, indicate whether each material, system or method listed is relevant to your study. If you are not sure if a list item applies to your research, read the appropriate section before selecting a response.

Materials & experimental systems

n/a	Involved in the study	
[ ]	[x]	Antibodies
[ ]	[x]	Eukaryotic cell lines
[x]	[ ]	Palaeontology and archaeology
[ ]	[x]	Animals and other organisms
[x]	[ ]	Clinical data
[x]	[ ]	Dual use research of concern
[x]	[ ]	Plants

Methods

n/a	Involved in the study	
[x]	[ ]	ChIP-seq
[ ]	[x]	Flow cytometry
[x]	[ ]	MRI-based neuroimaging

Antibodies

Antibodies used	The following antibodies were used for western blotting. They are listed as antigen first, followed by supplier, catalog number as applicable.<br>1) anti-CAR, 1:500, Abmart, PK30578;<br>2) anti-cleaved caspase 3, 1:2000, Abmart, TA7022;<br>3) anti-COX17, 1:2000, Abmart, MG935574;

4) anti-DLAT, 1:1000, Abmart, T58125;
5) anti-FDX1, 1:1000, Abmart, T510671;
6) anti-TERT, 1:1000, Abmart, TD7129;
7) anti-adenovirus type 5 E1A, 1:200, Abcam, ab204123;
8) anti-AFG3L2, 1:2000, Abcam, ab68023;
9) anti-ATF3, 1:1000, Abcam, ab207434;
10) anti-HMGB1, 1:1000, Abcam, ab18256;
11) anti-HSP70, 1:2000, Abcam, ab2787;
12) anti-lipoic acid, 1:1000, Abcam, ab58724;
13) anti-p62, 1:1000, CST, #5114;
14) anti-β-catenin, 1:1000, CST, #8480;
15) anti-β-actin, 1:1000, CST, #4970;
16) HRP-conjugated Affinipure Goat Anti-Mouse IgG(H+L), 1:5000, Proteintech, Cat SA00001-1;
17) HRP-conjugated Affinipure Goat Anti-Rabbit IgG(H+L), 1:5000, Proteintech, Cat SA00001-2.

The following antibodies were used for immunohistochemistry (IHC) and immunofluorescence. They are listed as antigen first, followed by supplier, catalog number as applicable.
1) anti-CD8α, 1:200, CST, #98941;
2) anti-granzyme B, 1:200, CST, #44153;
3) anti-lipoic acid, 1:200, Abcam, ab58724;
4) anti-Ki67, 1:500, CST, #12202;
5) anti-CAR, 1:200, Abmart, PK30578;
6) anti-COX17, 1:200, Abmart, MG935574;
7) anti-FDX1, 1:200, Abmart, T510671;
8) anti-TERT, 1:500, Abmart, TD7129;
9) anti-HMGB1, 1:400, Abcam, ab18256;
10) anti-CRT, 1:400, CST, #12238;
11) HRP-conjugated secondary antibody (anti-rabbit), undiluted, Cell Signaling Technology, Cat 8114;
12) HRP-conjugated secondary antibody (anti-mouse), undiluted, Cell Signaling Technology, Cat 8125;
13) DyLight 488–conjugated anti-rabbit secondary antibody, 1:500, Abbkine, #23220.

The following primary antibodies were used for flow cytometry. They are listed as antigen first, followed by supplier and clone/catalog number as applicable.
1) Fixable Viability Dye, 1:1000, eBioscience, eFluor 506, Cat 65-0866-14;
2) anti-mouse CD45, 1:500, eBioscience, APC-Cy7, Clone I3/2.3, Cat A15395;
3) anti-mouse CD4, 1:500, eBioscience, eFluor 450, Clone RM4-5, Cat 48-0042-82;
4) anti-mouse CD8α, 1:500, eBioscience, PerCP, Clone 5H10, Cat MCD0831;
5) anti-mouse CD44, 1:500, eBioscience, PE, Clone IM7, Cat 12-0441-82;
6) anti-mouse CD62L, 1:200, eBioscience, APC, Clone MEL-14, Cat 17-0621-82;
7) anti-mouse Foxp3, 1:100, eBioscience, PE, Clone FJK-16s, Cat 12-5773-82;
8) anti-mouse CD80, 1:300, eBioscience, PE, Clone 16-10A1, Cat 12-0801-82;
9) anti-mouse CD86, 1:300, eBioscience, APC, Clone GL1, Cat 17-0862-82;
10) anti-mouse F4/80, 1:200, eBioscience, PE-Cy7, Clone BM8, Cat 25-4801-82;
11) anti-mouse CD25, 1:200, eBioscience, APC, Clone PC61.5, Cat 17-0251-82;
12) anti-mouse CD3ε, 1:500, BioLegend, PE-Cy7, Clone 145-2C11, Cat 100320;
13) anti-mouse CD11b, 1:500, BioLegend, PerCP, Clone M1/70, Cat 101230;
14) anti-mouse CD11c, 1:500, BioLegend, PerCP, Clone N418, Cat 117326;
15) anti-mouse CD86, 1:200, BioLegend, PE, Clone A17199A, Cat 159204;
16) anti-mouse CD206, 1:200, BioLegend, APC, Clone C068C2, Cat 141707;
17) anti-mouse HSP70, 1:200, Abcam, ab2787;
18) anti-mouse CAR, 1:100, Abcam, ab214354.
The following primary antibodies were used for blocking. They are listed as antigen first, followed by supplier and clone/catalog number as applicable.
1) anti-CD8α, 10 mg/kg, Bio X Cell, BE0061;
2) anti-CD4, 10 mg/kg, Bio X Cell, BP0003-1;
3) IgG2b isotype control antibody, 10 mg/kg, Bio X Cell, BP0090.

Validation

No customized antibodies were used. Validation data of the antibodies purchased from commercial vendors are available on the manufacturers' website and datasheets.
1) anti-CAR, Abmart, PK30578;
https://www.ab-mart.com.cn/page.aspx?node=%2077%20&id=%2075111
2) anti-cleaved caspase 3, Abmart, TA7022;
https://www.ab-mart.com.cn/page.aspx?node=%2077%20&id=%2021621
3) anti-COX17, Abmart, MG935574;
https://www.ab-mart.com.cn/page.aspx?node=%2077%20&id=%20346307
4) anti-DLAT, Abmart, T58125;
https://www.ab-mart.com.cn/page.aspx?node=%2077%20&id=%203551
5) anti-FDX1, Abmart, T510671;

https://www.ab-mart.com.cn/page.aspx?node=%2077%20&id=%20111277
6) anti-TERT, Abmart, TD7129;
https://www.ab-mart.com.cn/page.aspx?node=%2077%20&id=%2031427
7) anti-adenovirus type 5 E1A, Abcam, ab204123;
https://www.abcam.cn/products/primary-antibodies/adenovirus-type-5-e1a-antibody-ab204123
8) anti-AFG3L2, Abcam, ab68023;
https://www.abcam.cn/products/primary-antibodies/afg3l2-antibody-ab68023
9) anti-ATF3, Abcam, ab207434;
https://www.abcam.cn/products/primary-antibodies/atf3-antibody-epr19488-chip-grade-ab207434
10) anti-HMGB1, Abcam, ab18256;
https://www.abcam.cn/products/primary-antibodies/hmgb1-antibody-ab18256
11) anti-HSP70, Abcam, ab2787;
https://www.abcam.cn/products/primary-antibodies/hsp70-antibody-5a5-ab2787
12) anti-lipoic acid, Abcam, ab58724;
https://www.abcam.cn/products/primary-antibodies/lipoic-acid-antibody-ab58724
13) anti-p62, CST, #5114;
https://www.cellsignal.cn/products/primary-antibodies/sqstm1-p62-antibody/5114
14) anti-β-catenin, CST, #8480;
https://www.cellsignal.cn/products/primary-antibodies/beta-catenin-d10a8-rabbit-monoclonal-antibody/8480
15) anti-β-actin, CST, #4970;
https://www.cellsignal.cn/products/primary-antibodies/beta-actin-13e5-rabbit-monoclonal-antibody/4970
16) HRP-conjugated Goat Anti-Mouse IgG(H+L), Proteintech, Cat SA00001-1;
https://www.ptgcn.com/products/HRP-conjugated-Goat-Anti-Mouse-IgG-H-L-SA00001-1.htm
17) HRP-conjugated Goat Anti-Rabbit IgG(H+L), Proteintech, Cat SA00001-2;
https://www.ptgcn.com/products/HRP-conjugated-Goat-Anti-Rabbit-IgG-H-L-SA00001-2.htm
18) anti-CD8α, CST, #98941;
https://www.cellsignal.cn/products/primary-antibodies/cd8-alpha-d4w2z-rabbit-monoclonal-antibody/98941
19) anti-granzyme B, CST, #44153;
https://www.cellsignal.cn/products/primary-antibodies/granzyme-b-e5v2l-rabbit-monoclonal-antibody/44153
20) anti-Ki67, CST, #12202;
https://www.cellsignal.cn/products/primary-antibodies/ki-67-d3b5-rabbit-monoclonal-antibody-ihc-formulated/12202
21) anti-CRT, CST, #12238;
https://www.cellsignal.cn/products/primary-antibodies/calreticulin-d3e6-rabbit-monoclonal-antibody/12238
22) HRP-conjugated secondary antibody (anti-rabbit), undiluted, Cell Signaling Technology, Cat 8114;
https://www.cellsignal.cn/products/secondary-antibodies/boost-ihc-detection-reagent-hrp-rabbit/8114
23) HRP-conjugated secondary antibody (anti-mouse), undiluted, Cell Signaling Technology, Cat 8125;
https://www.cellsignal.cn/products/secondary-antibodies/boost-ihc-detection-reagent-hrp-mouse/8125
24) DyLight 488–conjugated anti-rabbit secondary antibody, Abbkine, #23220;
https://www.abbkine.com/product/dylight-488-goat-anti-rabbit-igg-a23220/
25) Fixable Viability Dye, eBioscience, eFluor 506, Cat 65-0866-14;
https://www.thermofisher.cn/order/catalog/product/65-0866-14?SID=srch-srp-65-0866-14
26) anti-mouse CD45, eBioscience, APC-Cy7, Clone I3/2.3, Cat A15395;
https://www.thermofisher.cn/cn/zh/antibody/product/CD45-Antibody-clone-I3-2-3-Monoclonal/A15395
27) anti-mouse CD4, eBioscience, eFluor 450, Clone RM4-5, Cat 48-0042-82;
https://www.thermofisher.cn/cn/zh/antibody/product/CD4-Antibody-clone-RM4-5-Monoclonal/48-0042-82
28) anti-mouse CD8α, eBioscience, PerCP, Clone 5H10, Cat MCD0831;
https://www.thermofisher.cn/cn/zh/antibody/product/CD8-alpha-Antibody-clone-5H10-Monoclonal/MCD0831
29) anti-mouse CD44, eBioscience, PE, Clone IM7, Cat 12-0441-82;
https://www.thermofisher.cn/cn/zh/antibody/product/CD44-Antibody-clone-IM7-Monoclonal/12-0441-82
30) anti-mouse CD62L, eBioscience, APC, Clone MEL-14, Cat 17-0621-82;
https://www.thermofisher.cn/cn/zh/antibody/product/CD62L-L-Selectin-Antibody-clone-MEL-14-Monoclonal/17-0621-82
31) anti-mouse CD25, eBioscience, APC, Clone PC61.5, Cat 17-0251-82;
https://www.thermofisher.cn/cn/zh/antibody/product/CD25-Antibody-clone-PC61-5-Monoclonal/17-0251-82
32) anti-mouse Foxp3, eBioscience, PE, Clone FJK-16s, Cat 12-5773-82;
https://www.thermofisher.cn/cn/zh/antibody/product/FOXP3-Antibody-clone-FJK-16s-Monoclonal/12-5773-82
33) anti-mouse CD80, eBioscience, PE, Clone 16-10A1, Cat 12-0801-82;
https://www.thermofisher.cn/cn/zh/antibody/product/CD80-B7-1-Antibody-clone-16-10A1-Monoclonal/12-0801-82
34) anti-mouse CD86, eBioscience, APC, Clone GL1, Cat 17-0862-82;
https://www.thermofisher.cn/cn/zh/antibody/product/CD86-B7-2-Antibody-clone-GL1-Monoclonal/17-0862-82
35) anti-mouse F4/80, eBioscience, PE-Cy7, Clone BM8, Cat 25-4801-82;
https://www.thermofisher.cn/cn/zh/antibody/product/F4-80-Antibody-clone-BM8-Monoclonal/25-4801-82
36) anti-mouse CD3ε, 1:500, BioLegend, PE-Cy7, Clone 145-2C11, Cat 100320;
https://www.biolegend.com/en-us/products/pe-cyanine7-anti-mouse-cd3epsilon-antibody-1899
37) anti-mouse CD11b, 1:500, BioLegend, PerCP, Clone M1/70, Cat 101230;
https://www.biolegend.com/en-us/products/percp-anti-mouse-human-cd11b-antibody-4315
38) anti-mouse CD11c, 1:500, BioLegend, PerCP, Clone N418, Cat 117326;
https://www.biolegend.com/en-us/products/percp-anti-mouse-cd11c-antibody-4259
39) anti-mouse CD86, BioLegend, PE, Clone A17199A, Cat 159204;
https://www.biolegend.com/en-us/products/pe-anti-mouse-cd86-antibody-18945

40) anti-mouse CD206, BioLegend, APC, Clone C068C2, Cat 141707;
https://www.biolegend.com/en-us/products/apc-anti-mouse-cd206-mmr-antibody-7425
41) anti-CD8α, Bio X Cell, BE0061;
https://bioxcell.com/invivomab-anti-mouse-cd8-alpha-be0061?queryID=eba49e96143aafdd651c2d1b3565b15e&objectID=30539&indexName=bioxcell_live_default_products
42) anti-CD4, Bio X Cell, BP0003-1;
https://bioxcell.com/invivomab-anti-mouse-cd4-be0003-1?queryID=ee50cee53ecfb3c8f7f4e2fe869bdf54&objectID=30483&indexName=bioxcell_live_default_products
43) IgG2b isotype control antibody, Bio X Cell, BP0090.
https://bioxcell.com/invivoplus-rat-igg2b-isotype-control-anti-keyhole-limpet-hemocyanin-bp0090

Eukaryotic cell lines

Policy information about cell lines and Sex and Gender in Research

Cell line source(s)	The murine breast cancer cell line 4T1 was purchased from the American Type Culture Collection (ATCC; CRL-2539). The murine oral carcinoma cell line MOC1 and colon adenocarcinoma cell line MC38 were obtained from Kerafast (catalog numbers EWL001-FP and ENH204-FP, respectively). The murine esophageal squamous cell carcinoma cell line AKR was sourced from Otwo Biotech (HTX2545). The murine macrophage cell line RAW 264.7 was acquired from ATCC (TIB-71). The human embryonic kidney 293T cells was from ATCC (CRL-3216).
Authentication	Each cell line was morphologically confirmed according to the information provided by the cell-source center, and the cell lines were authenticated with short tandem repeat (STR) analysis.
Mycoplasma contamination	All the cell lines presented in this study were tested for mycoplasma contamination and they were free of mycoplasma contamination.
Commonly misidentified lines (See ICLAC register)	No commonly misidentified cell lines were used.

Animals and other research organisms

Policy information about studies involving animals; ARRIVE guidelines recommended for reporting animal research, and Sex and Gender in Research

Laboratory animals	Female BALB/c and C57BL/6 mice (6–8 weeks old) were obtained from GemPharmatech (Nanjing, China). Mice were maintained under specific pathogen-free conditions at approximately 22 °C and 50% relative humidity under a 12-h light/12-h dark cycle, with food and water provided ad libitum. The numbers of animals used in individual experiments are reported in the corresponding figure legends and Source Data.
Wild animals	The study did not involve wild animals.
Reporting on sex	Only female mice were used in the animal experiments. The study was designed to evaluate therapeutic efficacy rather than sex-dependent responses; therefore, sex was not included as an experimental variable and no sex-based comparisons were performed.
Field-collected samples	The study did not involve samples collected from the field.
Ethics oversight	All animal procedures were approved by the Institutional Animal Care and Use Committee of Zhengzhou University (approval number: ZZU-LAC20251212[28]) and complied with the national guidelines for laboratory animal care and use issued by the State Council of the People's Republic of China.

Note that full information on the approval of the study protocol must also be provided in the manuscript.

Plants

Seed stocks	This study does not involve.
Novel plant genotypes	This study does not involve.
Authentication	This study does not involve.

Flow Cytometry

Plots

Confirm that:

- [x] The axis labels state the marker and fluorochrome used (e.g. CD4-FITC).
- [x] The axis scales are clearly visible. Include numbers along axes only for bottom left plot of group (a 'group' is an analysis of identical markers).
- [x] All plots are contour plots with outliers or pseudocolor plots.
- [x] A numerical value for number of cells or percentage (with statistics) is provided.

Methodology

Sample preparation	For lymphocyte analysis, tumors and tumor-draining lymph nodes were collected from mice and made into cell suspensions by gentleMACS™ dissociator and digestive enzyme (Miltenyi Biotec) according to the manufacturer's instructions. Then, the samples were passed through 200-mesh nylon mesh filters to obtain single-cell suspensions. Live or dead cells were separated by Fixable Viability Dye (eBioscience, eFluor 506). For all samples, cells were first stained with antibodies against surface antigens. In some experiments, cells were subsequently fixed, permeabilized and stained for intracellular antigens.
Instrument	CytoFLEX flow cytometer (Beckman).
Software	CytExpert v. 2.3 software (Beckman), FlowJo v10.0.6 (TreeStar).
Cell population abundance	Cell-population frequencies were quantified by analytical flow cytometry; no cell sorting was performed.
Gating strategy	The gating strategy was shown in Supplementary Fig. 16-19. Generally, single cell gates based on SSC-H and SSC-A, and FSC-H and FSC-A were used to exclude non-singlets. A live/dead cell gate based on fixable viability dye was used to exclude dead cells.

- [x] Tick this box to confirm that a figure exemplifying the gating strategy is provided in the Supplementary Information.
