# Supplementary material for: A cuproptosis-inducing covalent organic framework synergizes with oncolytic virus for tumor radiotherapy
Source: Nat Commun. 2026 Aug 21;17:10011. doi: 10.1038/s41467-026-76947-w (PMC13590516; doi:10.1038/s41467-026-76947-w)
Supplement: Supplementary file 3 [file 41467_2026_76947_MOESM3_ESM.pdf]

nature portfolio

Peer Review File

A cuproptosis-inducing covalent organic framework synergizes with oncolytic virus for tumor radiotherapy

Corresponding Author: Professor Wei He

This file contains all reviewer reports in order by version, followed by all author rebuttals in order by version.

Version 0:

Reviewer comments:

Reviewer #1

(Remarks to the Author)

This manuscript reports a radiation responsive copper incorporated COF (COF Tpy Se Cu) combined with an oncolytic adenovirus (OVAD) to amplify cuproptosis and improve radiotherapy, with additional immune microenvironment remodeling claims. However, several critical controls and quantitative validations are needed to firmly support the central conclusion that the in vivo efficacy/immune activation is irradiation-triggered and copper-driven.

Major comments:
1. In vitro data show IR-triggered structural disruption and Cu elevation, but it remains unclear whether COF Tpy Se Cu is stable in vivo without irradiation, or whether Cu leaks due to tumor microenvironment factors (pH/redox/proteins/enzymes). The authors should check Cu release profiles in physiologically relevant conditions ±IR (e.g., serum, pH 7.4/6.5/5.0, ±GSH/H_2O_2), and ideally in vivo tumor Cu kinetics (ICP MS biodistribution/time course) with and without IR to show that Cu elevation is predominantly IR-dependent.

2. Current in vivo groupings mainly show "material + IR works," but do not isolate copper's role. The authors should check IR + COF Tpy Se (Cu free scaffold control) in the same tumor models and immune readouts, and add IR + COF Tpy Se Cu + copper chelator group (in vivo chelation rescue; the authors can choose an appropriate chelator) to test whether efficacy and immune activation diminish when copper is sequestered.

Without these key controls, the manuscript cannot conclusively attribute effects to Cu/cuproptosis rather than other mechanisms.

3. The manuscript infers cuproptosis via downstream markers and FDX1 dependence, but does not directly demonstrate that IR-triggered copper reaches cuproptosis-effective levels in tumors. The authors should include the quantification of intratumoral copper (and ideally mitochondrial copper) after treatment across groups, plus correlation with more direct cuproptosis hallmarks from tumor lysates (e.g., lipoylated protein aggregation/Fe S protein loss/proteotoxic stress markers).

4. The "lung metastasis" section is largely qualitative. The authors should add nodule counts and/or metastatic area fraction on lung sections, and adjust magnification to show both nodule size and surrounding microenvironment.

5. Supplementary lung H&E panels (e.g., Supp. Fig. 12 and 21) appear to show possible structural alterations in some treated groups. The authors need to verify there is no misused images, and add blinded pathology scoring (at least for lung).

Minor comments:
The protocol describes tail vein injection of tumor cells to establish lung lesions; this is more consistent with an experimental lung colonization model, not "spontaneous metastasis." Please correct terminology in text/figures.

The Methods state the OVAD vector map is in "Supplementary Fig. 17a," but the vector map appears in Supp. Fig. 18a. Please correct cross-references.

Reviewer #2

(Remarks to the Author)
This study begins with the scientific observation that radioresistant cancer cells exhibit increased susceptibility to cuproptosis, and combines a radiation-responsive, copper-incorporated covalent organic framework (COF-Tpy-Se-Cu) with an oncolytic adenovirus (OVAD) to synergistically induce cuproptosis. Although the manuscript presents a substantial amount of data, its exploration of the mechanisms underlying tumor immunotherapy and molecular signaling is insufficient. Moreover, several issues exist in the experiments related to immune data and molecular pathway analysis. This work is more characteristic of a study focused on the synthesis and application of novel materials. Therefore, it is not recommended for publication in Nature Communications; resubmission to a materials science-oriented journal would be more appropriate.

Major Comments
1. 8i: The in vivo CD8 depletion antibody experiment indicates that after $CD8^+$ cell depletion, there remains a substantial gap in tumor growth between the COF Tpy Se Cu-OVAD group and both the CD8 depletion-only group and the control group (although no statistical difference is shown). Furthermore, previous COF Tpy Se Cu-OVAD treatment experiments (7e-7i) showed changes in $CD25^+FOXP3^+$ cells. Does this suggest that $CD4^+$ cells also play a role? It is necessary to evaluate tumor rescue in the COF Tpy Se Cu-OVAD group by using $CD8^+$ and $CD4^+$ depletion antibodies, both alone and in combination.

2. 8h: Quantify the number of metastatic foci per group.

3. S13: In the flow cytometry gating strategy for immune-cell profiling, label the proportion of each population. Also, show the initial population (prior to doublet exclusion). It is perplexing that the proportion of $CD3^+$ T cells appears to account for over 50% of $CD45^+$ cells, while $CD11b^+$ myeloid cells also represent far more than 50% of $CD45^+$ cells. Please provide a reasonable explanation.

4. S23: Clearly indicate in the flow cytometry plots which parent cell population each gated population was derived from.

5. S24: Please specify which cell group the gate was applied to. Are the displayed cells macrophages positive for CD86 and CD206? If so, why are there also a small proportion of $F4/80^-$ cells?

6. 446-449: It is difficult to determine from Figure 6d that protein lipoylation is enhanced in the OVAD group, as FDX levels are also markedly elevated in the OVAD group. Further detailed investigation using IP or other experiments is recommended.

7. Evidence is required to demonstrate that, following intratumoral injection, OVAD effectively infects tumor cells, mediates tumor cell lysis, and undergoes amplification within the tumor tissue.

8. A critical function of oncolytic viruses in anti-tumor therapy is the abscopal effect. Whether the combination therapy employing framework materials and OVs can simultaneously affect distal tumor foci requires investigation and validation—for example, via bilateral tumor-bearing models to monitor tumor growth and tumor microenvironment changes.

9. What is the specific information about your oncolytic virus? It should be assessed whether radiotherapy resistant tumor cells affect the expression of receptors for the oncolytic virus, as increased receptor expression can enhance viral infectivity and therapeutic efficacy. This requires validation at both cellular and animal levels. The cellular-level impact of radioresistance on tumor cell sensitivity to the oncolytic virus should be examined. In vivo experiments, a radiotherapy alone followed by oncolytic virus treatment group should be included to observe tumor growth differences compared with other groups.

Minor Comments
1. 4h: Add a quantitative statistical difference map.

2. 5f: Label the CD4 proportion on the figure and include a statistical plot showing the proportion of CD8 among $CD45^+$ cells.

3. 5j: Quantification of immunohistochemistry data is lacking. Additionally, the IHC images show that the morphology of CD8 and GZMB staining is not quite accurate. It would be better to use multiplex immunofluorescence to co-stain CD8, GZMB, KI67, and lipoic acid, as this would more intuitively demonstrate co-expression characteristics.

Reviewer #3

(Remarks to the Author)
This study utilizes the radioresistant cancer cells have enhanced cuproptosis susceptibility to design a radiation-responsive copper-incorporated COF (COF-Tpy-Se-Cu), which releases Cu^{2+} under X-ray irradiation to trigger cuproptosis. Combined with oncolytic adenovirus (OVAD) — which depletes GSH and stabilizes FDX1 by inhibiting AFG3L2-mediated degradation — this regimen synergistically amplifies cuproptosis, remodels the tumor immune microenvironment, converts cold tumors to hot ones, elicits robust T-cell-dependent antitumor immunity, suppresses metastasis and establishes durable immune

memory. In general, the data provided in this study can’t support its conclusions. The novelty also needs further explanation.

1. The combination of cuproptosis and re-radiation sensitization have been reported before. In addition, radioresistant cancer cells exhibiting enhanced cuproptosis susceptibility was also not the first time to be identified. The specific novelty of the current work remains to be clearly articulated and emphasized.
2. How were MOC1, 4T1 and MC38 tumor types chosen? Additionally, the specific design principles of the current nanomaterial (COF-Tpy-Se-Cu) tailored to the biological characteristics of these tumor types, and how it achieves targeted therapeutic effects against them, need to be elaborated in detail.
3. Considering the specific tumor type, orthotopic tumor models should be established to better illustrate therapeutic effects.
4. In vivo biodistribution and excretion profiles of as-prepared medicine should be studied.
5. The framework contains too many components, underlying mechanism of individual components, pairwise combinations or the full composite systems haven’t been systematically studied.
6. To assess radiotherapeutic effects, colony formation experiment is a golden standard. This core data is missing.

Version 1:

Reviewer comments:

Reviewer #1

(Remarks to the Author)
The authors have largely addressed the comments made by this reviewer. The overall quality and rigor of this study have significantly improved. The revised manuscript has met the requirements for its publication in NC.

The recent published work on the role of cuproptosis in ICD and antitumor immunity (PMID: 42330950) is highly relevant to this study. This recent publication is cited in the revised manuscript (reference 48) but without any discussion. The authors should add a brief discussion to put their findings in the context of this highly relevant study.

Reviewer #2

(Remarks to the Author)
Following rigorous revision and the addition of substantial experimental evidence, the regulatory mechanisms and immunological rationale are now thoroughly substantiated. Key additions include in vivo$CD4^+$/$CD8^+$ T cell depletion assays, co-immunoprecipitation validation of DLAT lipoylation, systematic characterization of intratumoral viral replication, bilateral tumor models to evaluate the abscopal effect, and standardized documentation of all flow cytometry gating strategies. Consequently, we believe the manuscript meets the publication criteria for Nature Communications, and we respectfully request the Editor's consideration for acceptance

Reviewer #3

(Remarks to the Author)
Many data can't support its conclusion. For instance, Figure S30e didn't show "the triple combination further promoted DLAT oligomerization" and S30f can't compare with 30e seperately to support the statement of "enhanced molecular phenotype remains copper-dependent".

Open Access This Peer Review File is licensed under a Creative Commons Attribution 4.0 International License, which permits use, sharing, adaptation, distribution and reproduction in any medium or format, as long as you give appropriate credit to the original author(s) and the source, provide a link to the Creative Commons license, and indicate if changes were made.
In cases where reviewers are anonymous, credit should be given to 'Anonymous Referee' and the source.
The images or other third party material in this Peer Review File are included in the article's Creative Commons license, unless indicated otherwise in a credit line to the material. If material is not included in the article's Creative Commons license and your intended use is not permitted by statutory regulation or exceeds the permitted use, you will need to obtain permission directly from the copyright holder.
To view a copy of this license, visit https://creativecommons.org/licenses/by/4.0/

Responses to the comments of Reviewer #1

Comment:

This manuscript reports a radiation responsive copper incorporated COF (COF Tpy Se Cu) combined with an oncolytic adenovirus (OVAD) to amplify cuproptosis and improve radiotherapy, with additional immune microenvironment remodeling claims. However, several critical controls and quantitative validations are needed to firmly support the central conclusion that the in vivo efficacy/immune activation is irradiation-triggered and copper-driven.

Response:

We sincerely appreciate the reviewer's constructive feedback, which has been very helpful in strengthening our work. We also apologize for any lack of clarity or insufficient supporting detail in the original presentation. We have carefully reconsidered each of your concerns and suggestions, and have performed additional experiments and analyses as recommended. Following thorough revisions that address all comments, we hope that the quality and clarity of the manuscript have been substantially improved. All revisions are highlighted in yellow in the revised main text and supplementary files.

Comment 1:

1. In vitro data show IR-triggered structural disruption and Cu elevation, but it remains unclear whether COF Tpy Se Cu is stable in vivo without irradiation, or whether Cu leaks due to tumor microenvironment factors (pH/redox/proteins/enzymes). The authors should check Cu release profiles in physiologically relevant conditions ±IR (e.g., serum, pH 7.4/6.5/5.0, ±GSH/H_2O_2), and ideally in vivo tumor Cu kinetics (ICP MS biodistribution/time course) with and without IR to show that Cu elevation is predominantly IR-dependent.

Response 1:

We sincerely appreciate the reviewer's valuable suggestion. In response, and to more clearly demonstrate that copper release from COF-Tpy-Se-Cu is predominantly

triggered by X-ray irradiation rather than passive leakage driven by tumor microenvironmental factors, we have systematically characterized copper release profiles under pathophysiologically relevant conditions (Supplementary Fig. 7) and performed time-resolved in vivo quantification of tumor copper levels (Supplementary Fig. 15a). All corresponding data have been added to the revised manuscript and Supplementary Information, as detailed below.

(1) In vitro copper release kinetics under pathophysiologically relevant conditions

Specifically in the manuscript:

"To further verify the structural stability and radiation-specific copper release profile, we monitored copper release kinetics over 72 h under a panel of pathophysiologically relevant conditions, with or without 4 Gy X-ray irradiation (Supplementary Fig. 7). In the absence of irradiation, negligible spontaneous copper release was observed across all tested conditions, including neutral physiological buffer, 10% serum, 10 mM glutathione (GSH), 100 μM hydrogen peroxide (H_2O_2), and acidic buffers (pH 6.5 and pH 5.0) mimicking the tumor microenvironment. These results confirm that COF-Tpy-Se-Cu maintains high structural integrity and exhibits minimal non-specific copper leakage when exposed to the redox, pH, and protein gradients characteristic of tumor tissue. By contrast, robust and time-dependent copper release was consistently detected across all conditions upon X-ray irradiation, verifying that copper liberation is primarily triggered by radiation (Supplementary Fig. 7)."

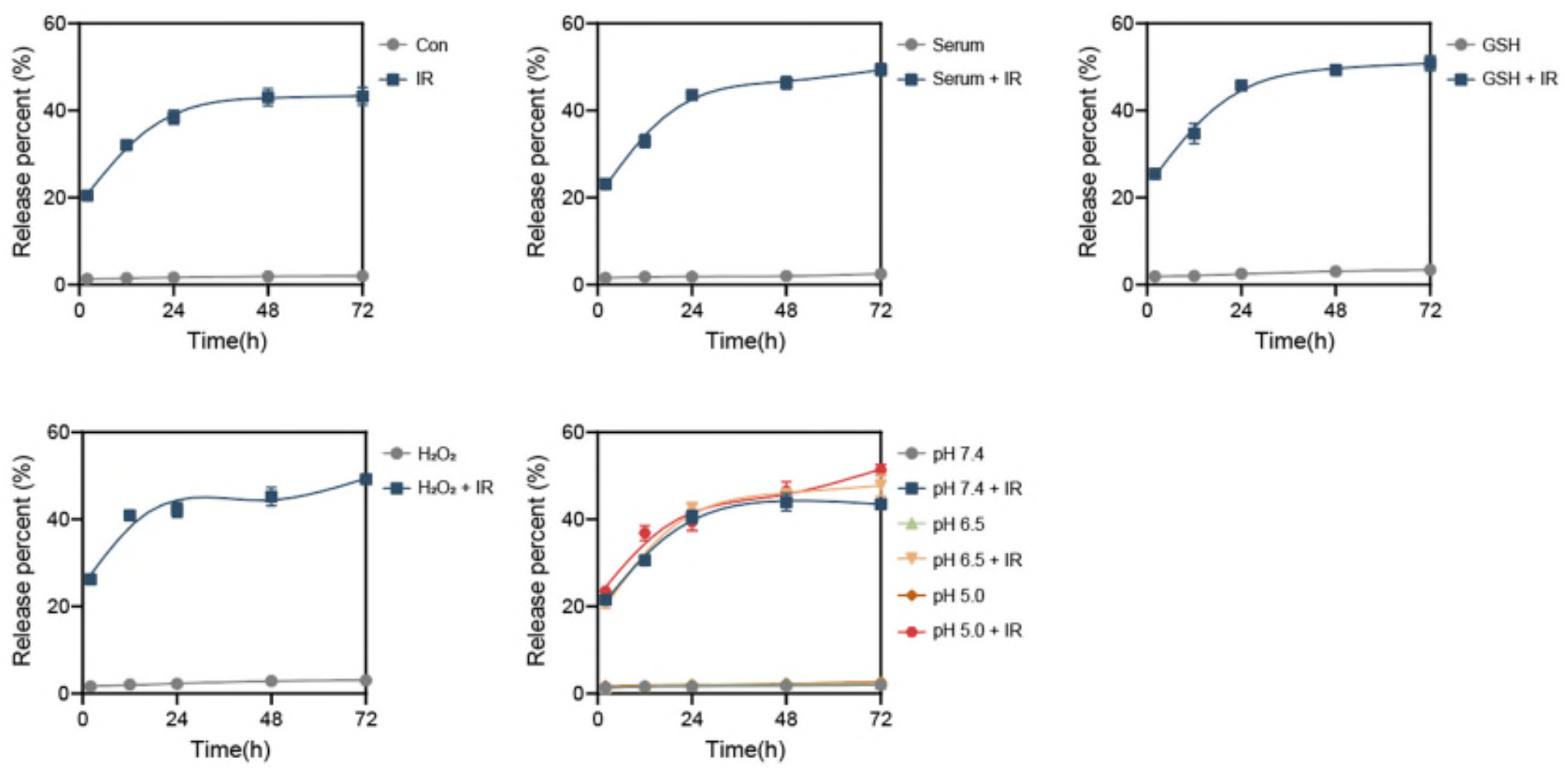

Supplementary Fig. 7| Copper release kinetics of COF-Tpy-Se-Cu under pathophysiologically relevant conditions. Time-dependent copper release profiles were determined over 72 h with or without 4 Gy X-ray irradiation, across a panel of conditions including neutral physiological buffer, 10% serum, 10 mM glutathione (GSH), 100 μM hydrogen peroxide (H_2O_2), and acidic buffers (pH 6.5 and pH 5.0) mimicking the tumor microenvironment. Data are presented as mean ± s.e.m. from at least three independent experiments.

(2) Time-course in vivo intratumoral copper quantification by ICP-MS

Specifically in the manuscript:

"To validate that intratumoral copper elevation is driven by irradiation rather than spontaneous framework leakage, we performed time-course ICP-MS analysis of copper levels in tumor (Supplementary Fig. 15a). In the absence of irradiation, intratumoral copper concentrations in the COF-Tpy-Se-Cu monotherapy group were comparable to those in the control and IR-only groups, confirming negligible spontaneous copper release from the framework in vivo. By contrast, the COF-Tpy-Se-Cu + IR group exhibited a pronounced, time-dependent increase in intratumoral copper content, which peaked at 24 h post-irradiation (Supplementary Fig. 15a)."

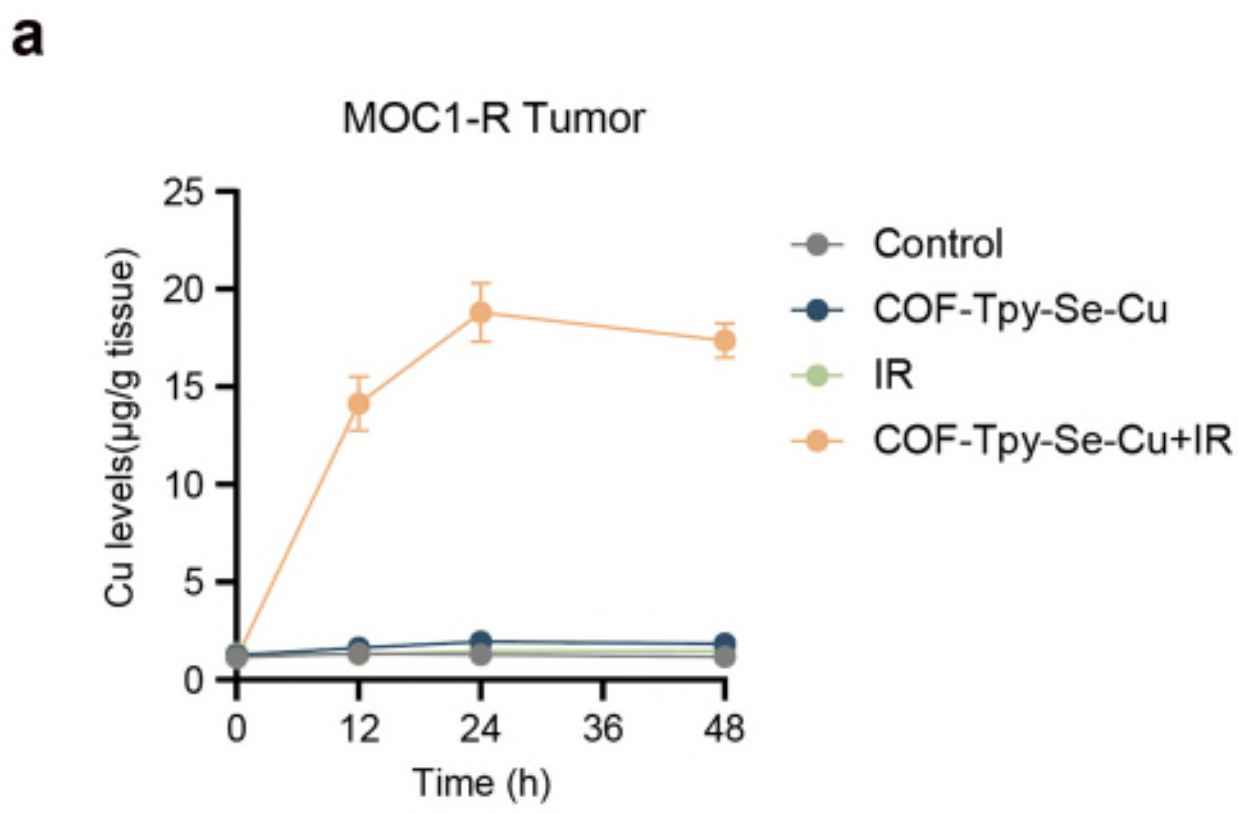

Supplementary Fig. 15| a Time-course ICP-MS quantification of copper concentrations in tumor, liver, and spleen tissues from the MOC1-R model across different treatment groups (COF-Tpy-Se-Cu, 2 mg kg^{-1}; IR, 4 Gy). Copper levels are normalized to tumor tissue weight. Data are presented as mean ± s.e.m. from at least three independent experiments.

Collectively, the in vitro release profiles under multiple pathophysiological

conditions and the in vivo time-course ICP-MS data consistently support that COF-Tpy-Se-Cu remains structurally stable in the tumor microenvironment without irradiation, and that intratumoral copper elevation is predominantly triggered by X-ray irradiation. We hope that these newly added data adequately address the reviewer's concern and further support the radiation-controlled mechanism of action of our material.

Comment 2:

2. Current in vivo groupings mainly show "material + IR works," but do not isolate copper's role. The authors should check IR + COF Tpy Se (Cu free scaffold control) in the same tumor models and immune readouts, and add IR + COF Tpy Se Cu + copper chelator group (in vivo chelation rescue; the authors can choose an appropriate chelator) to test whether efficacy and immune activation diminish when copper is sequestered.

Without these key controls, the manuscript cannot conclusively attribute effects to Cu/cuproptosis rather than other mechanisms.

Response 2:

We sincerely thank the reviewer for this important suggestion. We fully acknowledge that the original in vivo grouping did not sufficiently isolate the contribution of copper, and this comment has enabled us to provide further evidence that the in vivo antitumor efficacy and immune remodeling effects are specifically driven by copper release and subsequent cuproptosis, rather than by the COF scaffold itself or off-target effects. To isolate the contribution of copper, we have completed two complementary sets of in vivo control experiments in both MOC1-R orthotopic and 4T1-R subcutaneous tumor models, together with genetic validation of the core cuproptosis effector FDX1. All data have been incorporated into the revised manuscript and Supplementary Information, as detailed below.

(1) Copper-free scaffold control (IR + COF-Tpy-Se) (Supplementary Fig. 22)

The copper-free COF backbone (COF-Tpy-Se), administered at the same dosage and following the same treatment schedule as the copper-loaded formulation, was

evaluated both alone and in combination with irradiation. Neither COF-Tpy-Se alone nor COF-Tpy-Se combined with IR exerted significant tumor growth inhibition or immune remodeling effects in either tumor model, thereby helping to rule out a contribution from the COF framework itself.

(2) In vivo copper chelation rescue (IR + COF-Tpy-Se-Cu + TTM) (Supplementary Fig. 23)

Systemic administration of the copper-specific chelator tetrathiomolybdate (TTM) largely abrogated the tumor-suppressive effect of COF-Tpy-Se-Cu + IR, and reversed the associated upregulation of inflammatory chemokines and $CD8^+$ T cell effector genes, supporting the conclusion that bioavailable copper is required for both therapeutic efficacy and immune activation.

(3) Genetic validation via FDX1 ablation (Supplementary Fig. 24)

Genetic ablation of the core cuproptosis effector FDX1 further attenuated the antitumor activity and immune remodeling effects of the combination regimen in vivo, supporting its dependence on the canonical cuproptosis pathway.

Specifically in the manuscript:

"To confirm that the in vivo therapeutic effects are mediated by copper-dependent cuproptosis, we included material-based and pharmacological control groups. The copper-free scaffold COF-Tpy-Se, either alone or in combination with irradiation, exerted no antitumor activity in either model, ruling out a contribution from the framework itself (Supplementary Fig. 22a-f). Systemic administration of the copper chelator TTM largely abrogated the radiosensitizing efficacy of COF-Tpy-Se-Cu, verifying that bioavailable copper is required for tumor suppression (Supplementary Fig. 23a–f). Genetic ablation of FDX1 further attenuated the tumor-suppressive effect of the combination regimen in vivo (Supplementary Fig. 24a–d). Gene expression profiling further showed that upregulation of inflammatory chemokines (e.g., *Cxcl9* and *Cxcl10*) and T cell effector genes (e.g., *Gzmb* and *Ifng*) was restricted to the IR + COF-Tpy-Se-Cu group, and was abolished by copper chelation or FDX1 knockout (Supplementary Fig. 25a, b). Collectively, these data demonstrate that COF-Tpy-Se-Cu

mediates in vivo radiosensitization via FDX1-dependent cuproptosis, which drives immunogenic cell death and remodels the tumor microenvironment toward a T-cell-inflamed phenotype.”

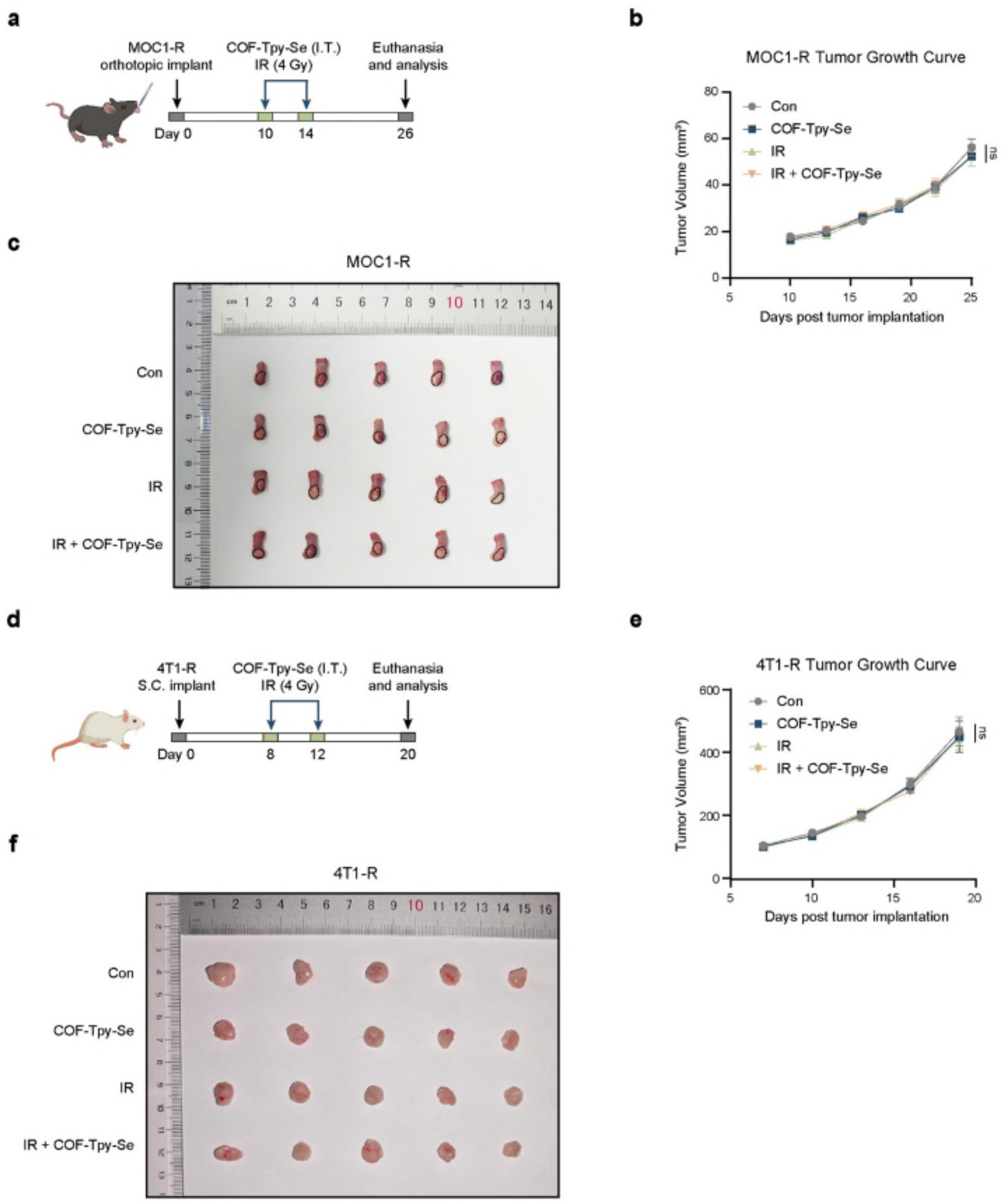

Supplementary Fig. 22| The copper-free COF-Tpy-Se scaffold exerts no antitumor efficacy in vivo. a Experimental scheme for tumor implantation and treatment in the MOC1-R models. Mice received intratumoral (i.t.) COF-Tpy-Se (2 mg kg^{-1}), followed by localized irradiation (IR, 4 Gy). **b** Tumor growth curves for MOC1-R tumors across the indicated treatment groups (n = 5 mice per group). **c** Representative images of excised tongues from mice bearing orthotopic MOC1-R tumors at the study endpoint. Tumor regions are delineated by black dashed lines. **d** Experimental scheme for tumor implantation and treatment in the 4T1-R models. Mice received intratumoral (i.t.) COF-Tpy-Se (5 mg kg^{-1}), followed by localized irradiation (IR, 4 Gy). **e** Tumor growth curves for 4T1-R tumors across the indicated treatment groups (n = 5 mice per group). **f** Representative images of resected subcutaneous 4T1-R tumors from each treatment group at the endpoint. Statistical

significance was assessed using two-way ANOVA (**b, e**). Data are presented as mean ± s.e.m.

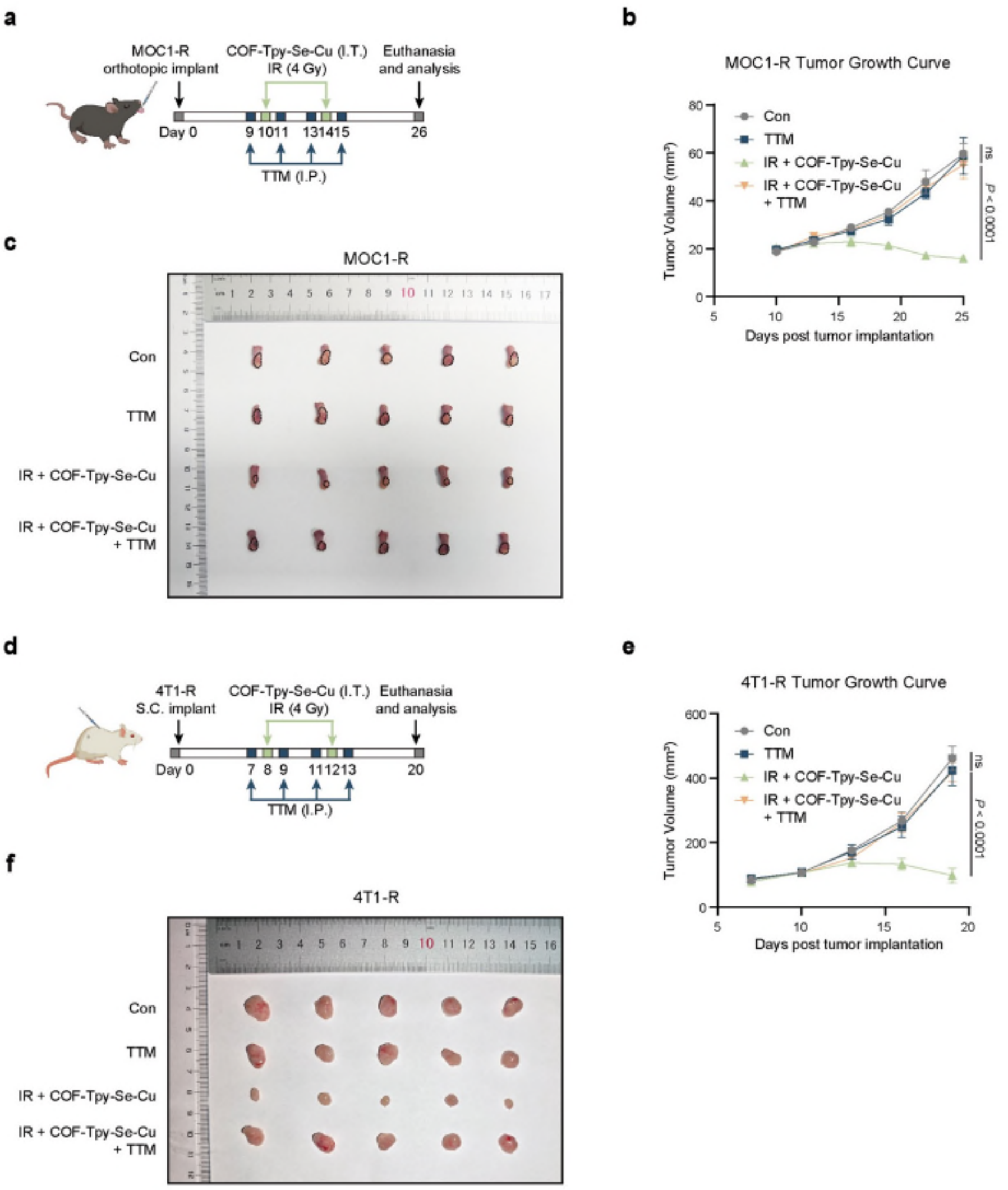

Supplementary Fig. 23| Copper chelation by TTM abrogates the in vivo radiosensitizing efficacy of COF-Tpy-Se-Cu. a Experimental schedule for the TTM rescue assay in the MOC1-R orthotopic tumor model. Mice received intratumoral (i.t.) injection of COF-Tpy-Se-Cu (2 mg kg^{-1}) and intraperitoneal (i.p.) administration of the copper chelator TTM (10 mg kg^{-1}), combined with localized irradiation (IR, 4 Gy) as indicated. **b** Tumor growth curves of MOC1-R orthotopic xenografts across the indicated treatment groups (n = 5 mice per group). **c** Representative images of excised MOC1-R orthotopic tumors from each treatment group at the study endpoint. **d** Experimental schedule for the TTM rescue assay in the 4T1-R subcutaneous tumor model. Mice received intratumoral (i.t.) injection of COF-Tpy-Se-Cu (5 mg kg^{-1}) and intraperitoneal (i.p.) TTM (10 mg kg^{-1}), combined with localized irradiation (IR, 4 Gy) as indicated. **e** Tumor growth curves of 4T1-R subcutaneous xenografts across the indicated treatment groups (n = 5 mice per group). **f** Representative images of excised 4T1-R subcutaneous tumors from each treatment group at the study endpoint. Statistical significance was assessed using two-way ANOVA (**b, e**). Data are

presented as mean ± s.e.m.

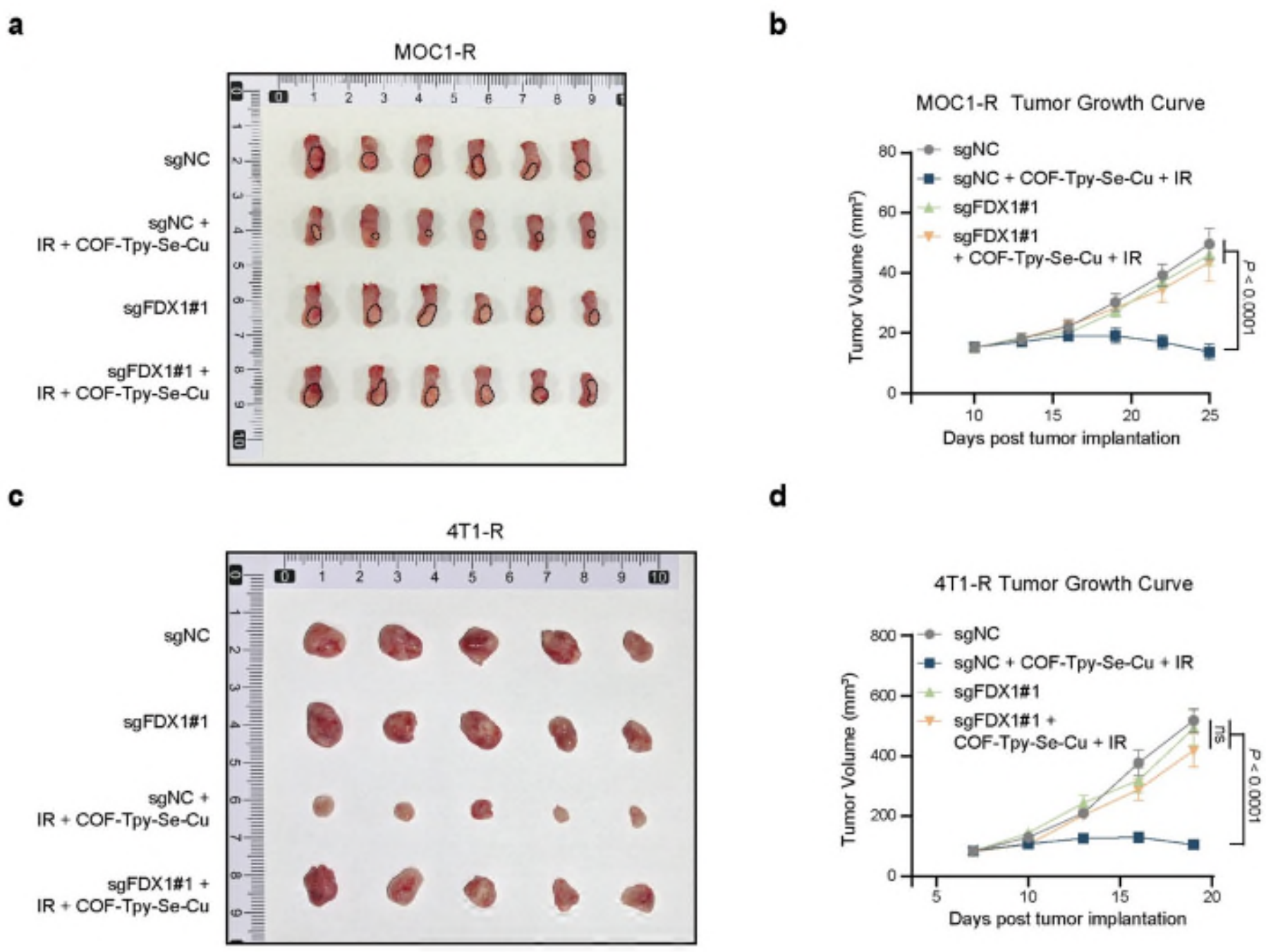

Supplementary Fig. 24| Genetic ablation of FDX1 attenuates the in vivo antitumor activity of COF-Tpy-Se-Cu plus irradiation. a Representative images of excised tongues from mice bearing orthotopic MOC1-R tumors at the study endpoint (n = 6 mice per group). Tumor regions are outlined with black dashed lines. **b** Tumor growth curves for MOC1-R tumor-bearing mice under the indicated treatment regimens. **c** Representative images of resected subcutaneous 4T1-R tumors from each treatment group at the endpoint (n = 5 mice per group). **d** Tumor growth curves for 4T1-R tumor-bearing mice under the indicated treatment regimens. Statistical significance was assessed using two-way ANOVA (**b, d**). Data are presented as mean ± s.e.m. from at least three independent experiments.

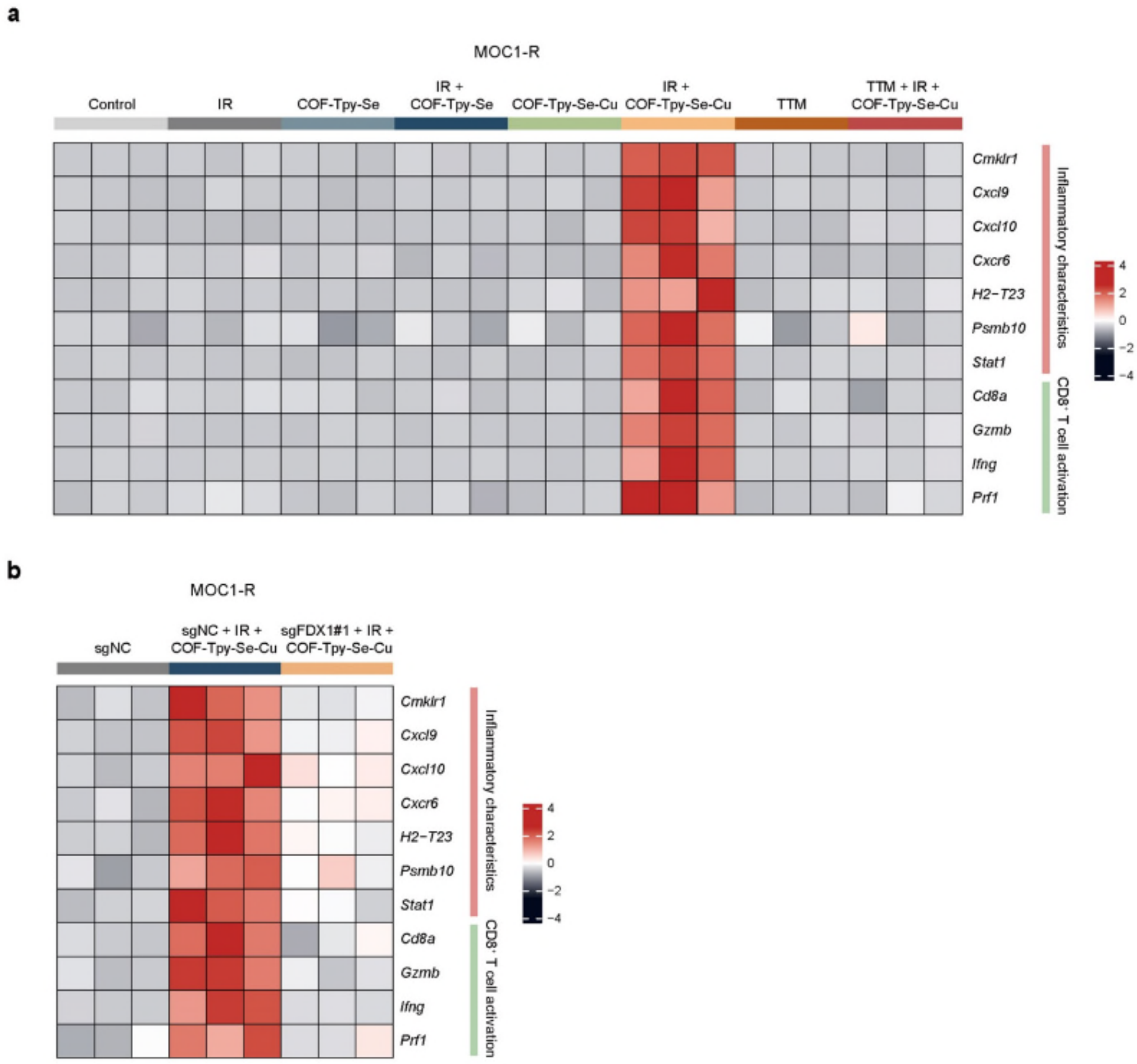

Supplementary Fig. 25| Heatmap summarizing expression of selected genes associated with inflammatory signatures and CD8^{+} T cell activation quantified by quantitative real-time PCR (qPCR) across treatment groups.

Collectively, three independent lines of evidence—copper-free scaffold control experiments, pharmacological copper chelation rescue assays, and genetic ablation of the core cuproptosis effector FDX1—consistently support that the in vivo antitumor efficacy and immune microenvironment remodeling are specifically driven by radiation-triggered copper release and subsequent FDX1-dependent cuproptosis, rather than by non-specific effects of the material scaffold itself or alternative off-target mechanisms. We are grateful to the reviewer for prompting us to add these key controls, which have substantially strengthened this conclusion.

<u>Comment 3:</u>

3. The manuscript infers cuproptosis via downstream markers and FDX1 dependence, but does not directly demonstrate that IR-triggered copper reaches cuproptosis-effective levels in tumors. The authors should include the quantification of intratumoral copper (and ideally mitochondrial copper) after treatment across groups, plus correlation with more direct cuproptosis hallmarks from tumor lysates (e.g., lipoylated protein aggregation/Fe S protein loss/proteotoxic stress markers).

Response 3:

We sincerely thank the reviewer for this valuable and rigorous suggestion. We fully agree that the previous presentation did not directly establish the functional link between radiation-triggered copper release and cuproptosis execution in vivo. To address this important point, we have now integrated quantitative analyses of subcellular copper distribution (Supplementary Fig. 15b, c) and canonical cuproptosis biochemical hallmarks (Supplementary Fig. 15d) across all treatment groups, with all data added to the revised manuscript and Supplementary Information, as detailed below. Specifically in the manuscript:

"We next assessed whether radiation-triggered copper release achieves functionally effective concentrations to drive cuproptosis in vivo by examining subcellular copper distribution and canonical cuproptosis biochemical hallmarks in tumor lysates. ICP-MS revealed no significant alterations in total cellular or mitochondrial copper levels following COF-Tpy-Se-Cu or irradiation alone (Supplementary Fig. 15b, c). In contrast, the combination treatment markedly elevated both total tumor copper and mitochondrial copper levels (Supplementary Fig. 15b, c), verifying selective copper enrichment within the mitochondrial compartment — the primary subcellular site of cuproptosis execution. At the protein level, the combination upregulated the proteotoxic stress marker HSP70 and induced DLAT oligomerization, a defining biochemical feature of cuproptosis (Supplementary Fig. 15d). Together, these data demonstrate that radiation-controlled copper delivery drives sufficient mitochondrial copper overload and elicits cuproptotic proteotoxic stress in vivo."

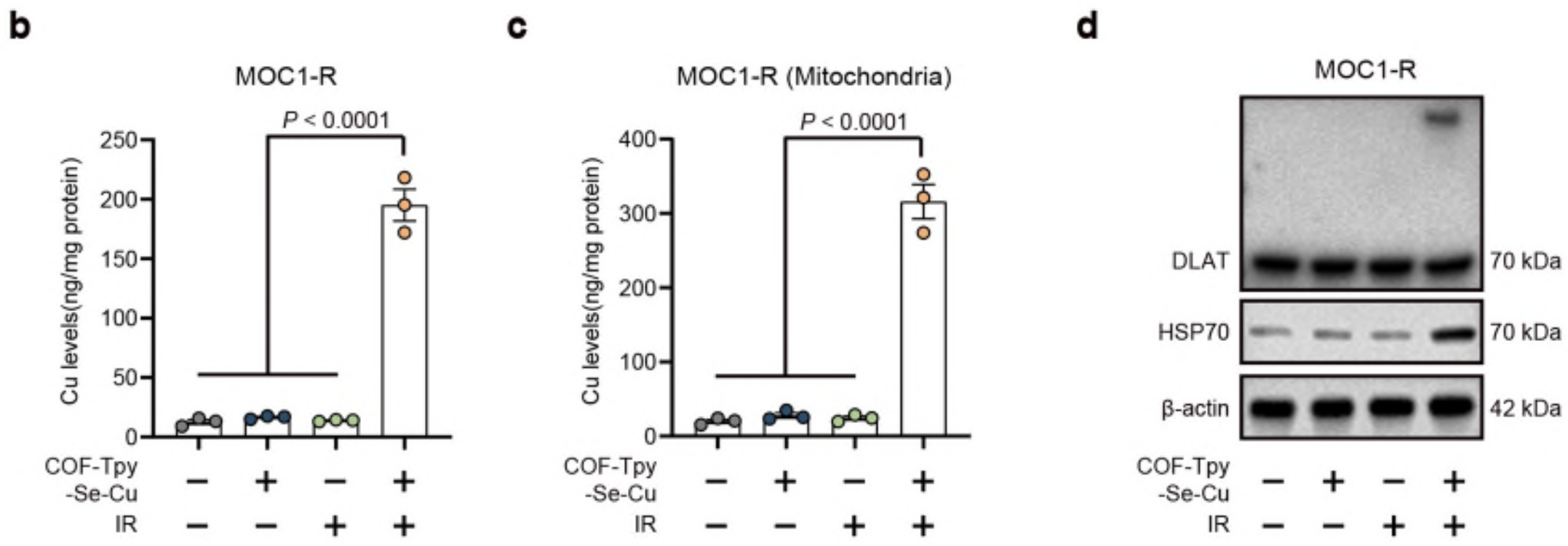

Supplementary Fig. 15| b ICP-MS quantification of total cellular copper levels in MOC1-R tumor tissues 24 h after the indicated treatments. Copper contents are normalized to total cellular protein. **c** ICP-MS quantification of mitochondrial copper levels in MOC1-R tumor tissues 24 h after the indicated treatments, normalized to mitochondrial protein. **d** Immunoblot analysis of DLAT oligomerization and the proteotoxic stress marker HSP70 in MOC1-R tumor lysates. β-actin served as the loading control. Statistical significance was assessed using one-way ANOVA with Tukey's multiple-comparisons test (**b, c**). Data are presented as mean ± s.e.m. from at least three independent experiments.

Collectively, the ICP-MS quantification and biochemical marker analyses support that radiation-triggered copper release from COF-Tpy-Se-Cu achieves functionally effective concentrations within tumor mitochondria and induces canonical cuproptotic proteotoxic stress and lipoylated protein aggregation in vivo. We hope that these newly added biochemical data provide a more direct basis for our central conclusion that the therapeutic effects are driven by copper-dependent cuproptosis.

<u>Comment 4:</u>

4. The "lung metastasis" section is largely qualitative. The authors should add nodule counts and/or metastatic area fraction on lung sections, and adjust magnification to show both nodule size and surrounding microenvironment.

<u>Response 4:</u>

We sincerely thank the reviewer for this constructive suggestion, which has enabled us to strengthen the quantitative rigor of our metastasis analysis. To address this point, we have now complemented the histological presentation with quantitative

enumeration of pulmonary metastatic nodules across treatment groups, and adjusted the image magnification to include both low-magnification panoramic views (to show overall metastatic burden) and high-magnification insets (to detail nodule morphology and the surrounding pulmonary parenchymal microenvironment).

Specifically in the manuscript:

"Furthermore, in an experimental lung colonization model, the triple combination regimen significantly attenuated pulmonary metastatic burden compared with the untreated control, and exhibited the most potent antimetastatic activity across all tested treatment arms (Fig. 8f–h and Supplementary Fig. 37k)."

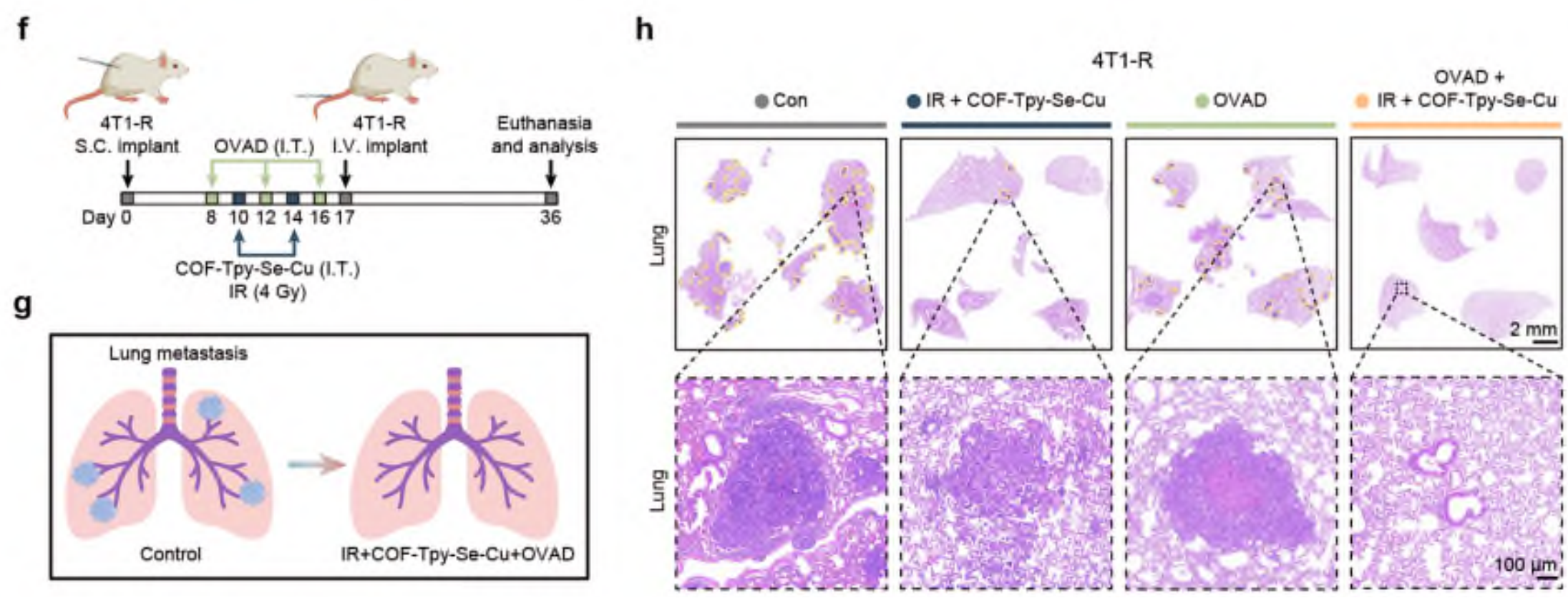

Fig. 8| f–h Experimental lung colonization model. (f, g) Schematic of the experimental design for assessing pulmonary metastatic burden. (h) Representative H&E-stained lung sections showing metastatic nodules across treatment groups (scale bar, 100 μm).

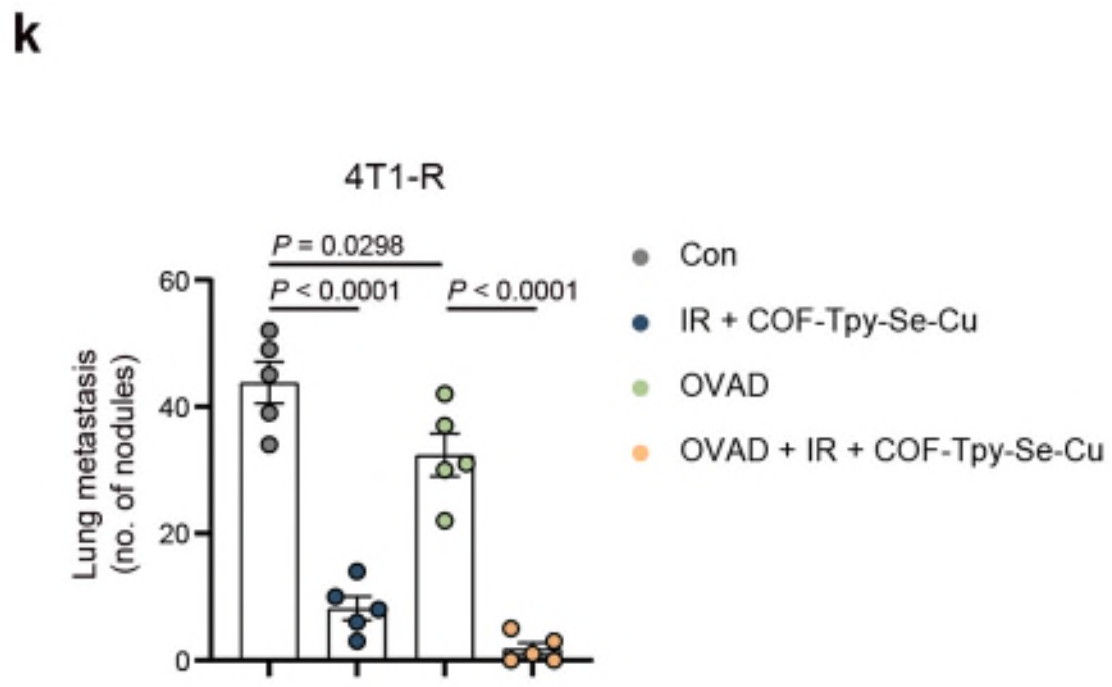

Supplementary Fig. 37| k Quantification of pulmonary metastatic nodules across treatment groups. Statistical significance was assessed using one-way ANOVA with Tukey's multiple-comparisons test (**k**). Data are presented as mean ± s.e.m.

<u>Comment 5:</u>

5. Supplementary lung H&E panels (e.g., Supp. Fig. 12 and 21) appear to show

possible structural alterations in some treated groups. The authors need to verify there is no misused images, and add blinded pathology scoring (at least for lung).

Response 5:

We sincerely thank the reviewer for raising this important point regarding image integrity and rigorous pathological assessment. We apologize for any ambiguity caused by the previous presentation of the lung H&E panels.

First, we carefully rechecked all lung H&E images (Supplementary Figs. 14, 33; formerly Supplementary Fig. 12, 21) in the supplementary figures by cross-referencing each panel with our original experimental records and raw image archives. We replaced several panels with more representative fields to avoid ambiguity.

Second, to provide an unbiased quantitative assessment of potential pulmonary toxicity, we added blinded histopathological scoring performed independently by two board-certified pathologists who were unaware of group assignments. A 0–4 semi-quantitative scoring system with 0.5-point increments was applied across four pathological domains: alveolar structural integrity, inflammatory cell infiltration, parenchymal tissue injury, and metastatic lesion load. For each sample, an average score across the four domains was calculated for group-level statistical analysis.

The scoring results confirm that no overt treatment-related histopathological abnormalities were detected in the normal lung parenchyma of any treatment group relative to controls, consistent with the favorable systemic safety profile supported by serum biochemistry and complete blood count data.

We have updated the corresponding figure accordingly:

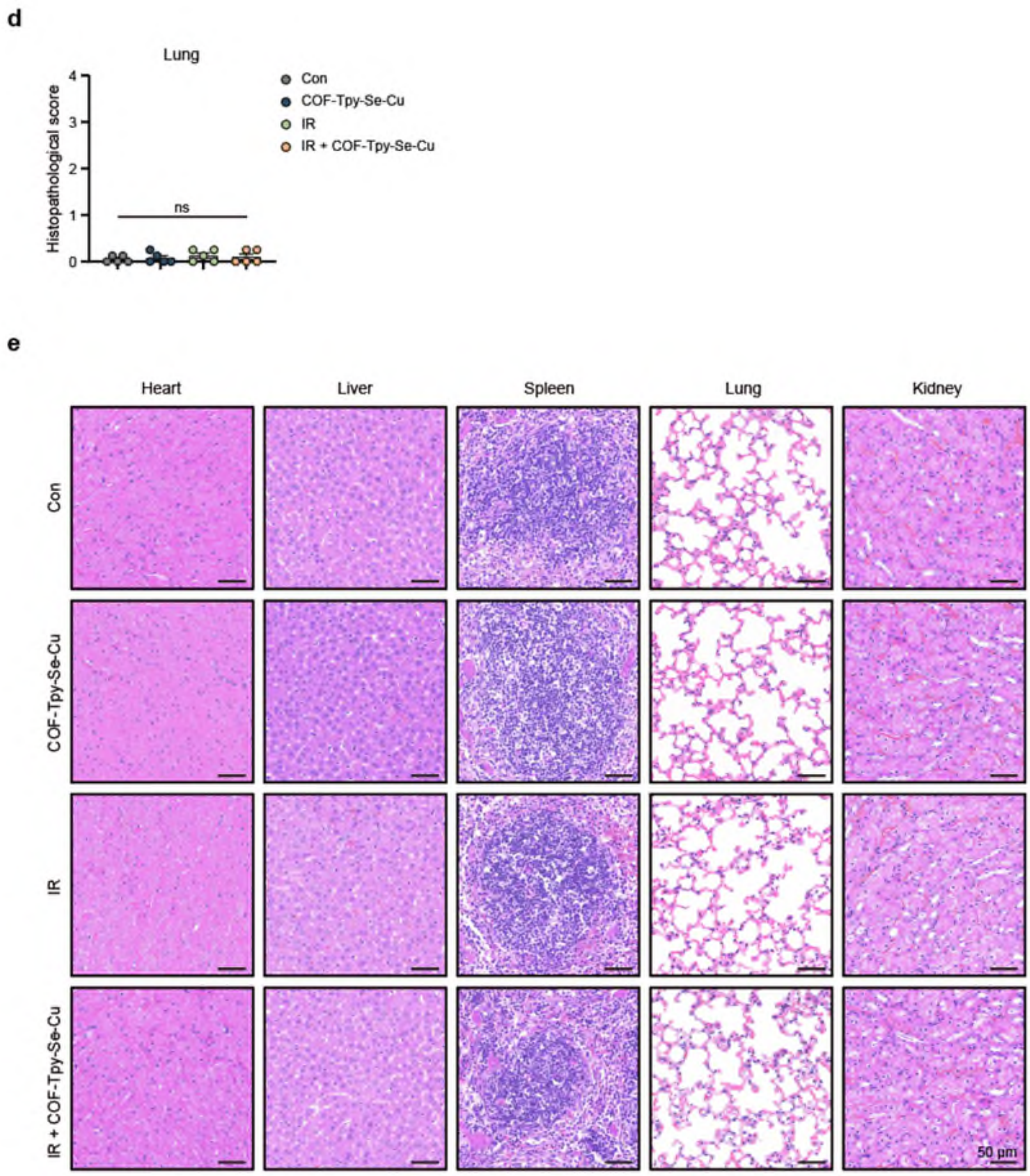

Supplementary Fig. 14| d Blinded semi-quantitative histopathological scoring of H&E-stained lung tissue sections across the indicated treatment groups. **e** Representative hematoxylin and eosin (H&E)–stained sections of the heart, liver, spleen, lung, and kidney from treated and control BALB/c mice are shown (scale bar, 50 μm). No overt histopathological abnormalities were observed in any treatment group relative to controls. Images are representative of five mice per group, with consistent findings across all groups. Statistical significance was assessed using one-way ANOVA with Tukey's multiple-comparisons test (**d**). Data are presented as mean ± s.e.m..

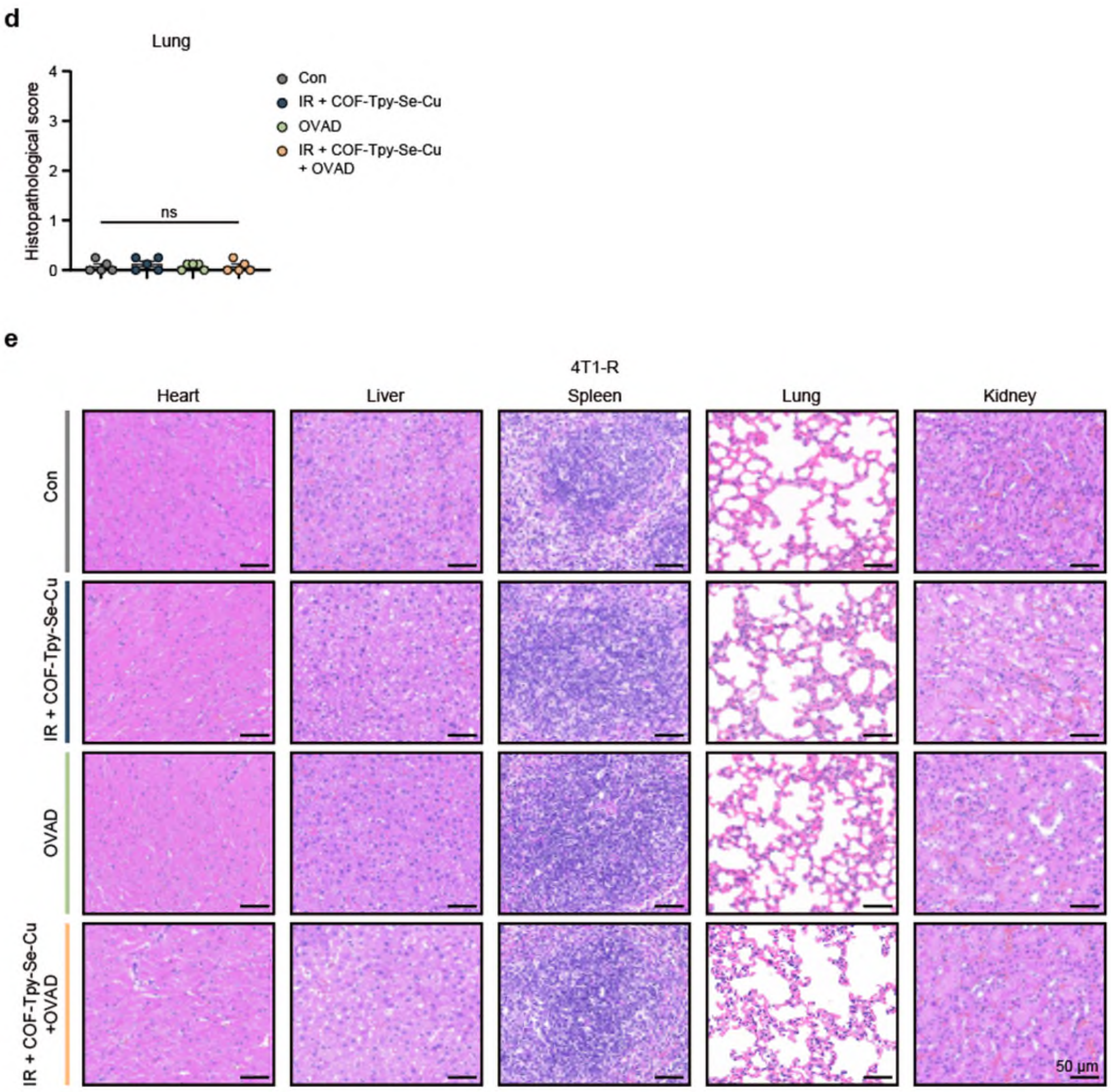

Supplementary Fig. 33| d Blinded semi-quantitative histopathological scoring of H&E-stained lung tissue sections across the indicated treatment groups. **e** Representative H&E-stained sections of major organs (heart, liver, spleen, lung, and kidney) from treated and control BALB/c mice (scale bar, 50 μm). No overt histopathological abnormalities were observed in any treatment group relative to controls. Images are representative of five mice per group, with consistent findings across all groups. Statistical significance was assessed using one-way ANOVA with Tukey's multiple-comparisons test (**d**). Data are presented as mean ± s.e.m..

Comment 6:

6. The protocol describes tail vein injection of tumor cells to establish lung lesions; this is more consistent with an experimental lung colonization model, not "spontaneous metastasis." Please correct terminology in text/figures.

Response 6:

We sincerely thank the reviewer for this precise and valuable correction. We apologize for the inaccurate terminology in the original version. Accordingly, we have

revised all relevant descriptions throughout the main text, figure legends, and Supplementary Information, replacing the inaccurate "spontaneous metastasis" statement with the standardized term "experimental lung colonization model".

Specifically in the "Results" section:

"Furthermore, in an experimental lung colonization model, the triple combination regimen significantly attenuated pulmonary metastatic burden compared with the untreated control, and exhibited the most potent antimetastatic activity across all tested treatment groups (Fig. 8f–h and Supplementary Fig. 37k)"

Specifically in the "Methods" section:

"To evaluate the lung colonization capacity of tumor cells, an experimental lung colonization model was established. BALB/c mice bearing established subcutaneous 4T1-R tumors were treated with COF-Tpy-Se-Cu and OVAD, followed by intravenous injection of 5×10^5 4T1-R cells via the tail vein to generate pulmonary metastatic foci. Lungs were harvested on day 36 for histopathological assessment and quantitative analysis of metastatic nodules."

Specifically in the figure legend:

"**Fig. 8| f–h** Experimental lung colonization model. (f, g) Schematic of the experimental design for assessing pulmonary metastatic burden. (h) Representative H&E-stained lung sections showing metastatic nodules across treatment groups (scale bar, 50 μm)."

We have verified the consistent use of this terminology across the entire manuscript to align with standard nomenclature in the field.

<u>Comment 7:</u>

7. The Methods state the OVAD vector map is in "Supplementary Fig. 17a," but the vector map appears in Supp. Fig. 18a. Please correct cross-references.

<u>Response 7:</u>

We sincerely thank the reviewer for carefully identifying this cross-reference discrepancy, and we apologize for this oversight. Following the reorganization and renumbering of supplementary figures during manuscript revision, the OVAD vector map is now located in Supplementary Fig. 26. We have corrected the mislabeled citation in the Oncolytic adenovirus subsection of the Methods section to match its actual position. To prevent similar numbering inconsistencies, we have also performed a full cross-check of all figure and supplementary figure citations throughout the manuscript to ensure complete alignment between in-text references and figure numbering.

Specifically in the "Methods" section:

"**Oncolytic adenovirus**

The oncolytic adenovirus OVAD-hTERT-E1A-IRES-E1B-SV40-EGFP-RGD (catalog no. OVAD184-2282; FuBio Biotechnology, Suzhou, China) was used in this study, with its vector map provided in Supplementary Fig. 26. This serotype 5 adenovirus features E1A/E1B expression driven by the human telomerase reverse transcriptase (hTERT) promoter for tumor-selective replication, a complete E3 region deletion, and an RGD-modified fiber knob mediating viral entry. An EGFP reporter was incorporated for infection tracking."

Specifically in the "Supplementary figure" section:

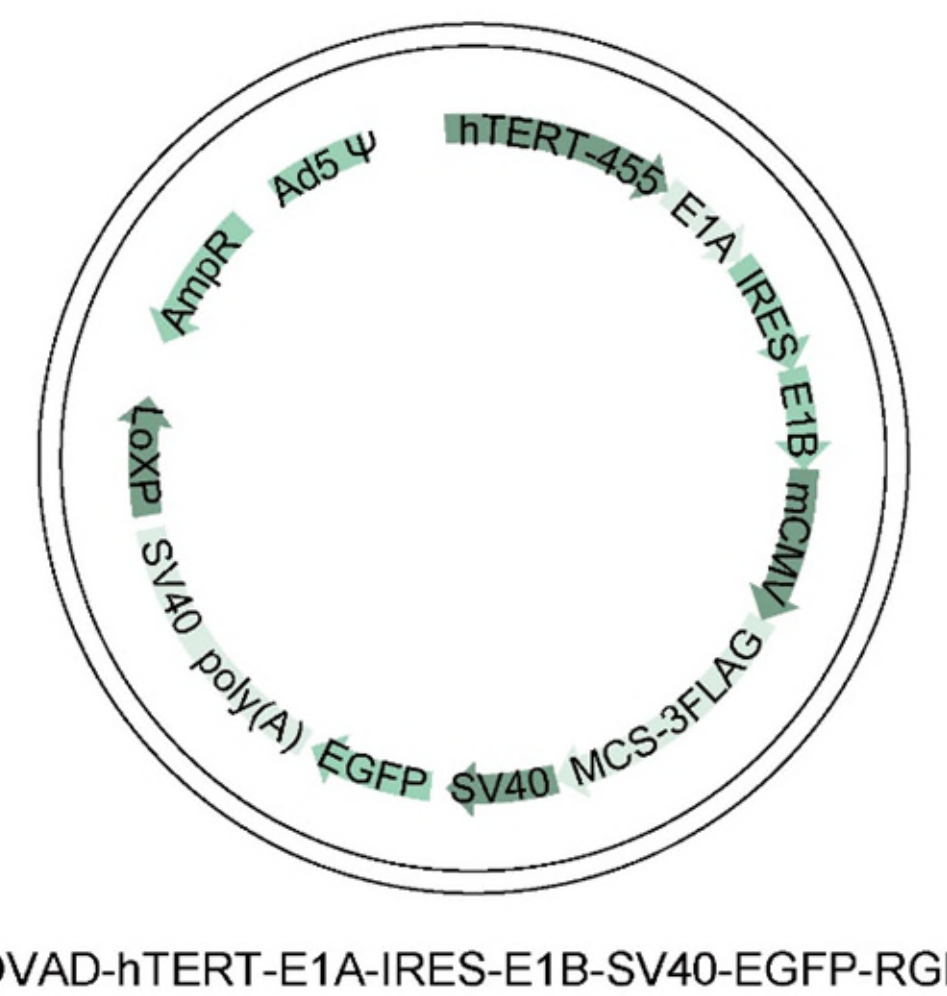

Supplementary Fig. 26| Schematic of the engineered oncolytic adenovirus vector OVAD-hTERT-E1A-IRES-E1B-SV40-EGFP-RGD (OVAD184-2282).

Responses to the comments of Reviewer #2

Comment:

This study begins with the scientific observation that radioresistant cancer cells exhibit increased susceptibility to cuproptosis, and combines a radiation-responsive, copper-incorporated covalent organic framework (COF-Tpy-Se-Cu) with an oncolytic adenovirus (OVAD) to synergistically induce cuproptosis. Although the manuscript presents a substantial amount of data, its exploration of the mechanisms underlying tumor immunotherapy and molecular signaling is insufficient. Moreover, several issues exist in the experiments related to immune data and molecular pathway analysis. This work is more characteristic of a study focused on the synthesis and application of novel materials. Therefore, it is not recommended for publication in Nature Communications; resubmission to a materials science-oriented journal would be more appropriate.

Response:

We sincerely appreciate your critical and constructive assessment of our work, which has helped us recognize key gaps in mechanistic depth and immune characterization and has guided us to substantially strengthen the study. We respectfully acknowledge that the original manuscript did not fully provide the level of mechanistic and immunological characterization expected for this work, and we apologize for the insufficiency of the previous presentation. We have carefully reconsidered every concern raised and performed a comprehensive set of additional experiments to address the issues regarding immunological mechanisms and molecular signaling pathways. These include in vivo $CD4^+/CD8^+$ T cell depletion assays, co-immunoprecipitation validation of DLAT lipoylation, systematic characterization of intratumoral viral replication, bilateral tumor models for abscopal effect evaluation, and standardized documentation of all flow cytometry gating strategies.

Following these thorough revisions addressing all points raised, we believe the manuscript has been substantially strengthened in scientific depth, mechanistic rigor, and biological relevance. With these added mechanistic and immunological data, we hope the revised manuscript may now meet the multidisciplinary publication standards

of Nature Communications. We respectfully submit the revised work for your consideration. All revisions made in response to your comments are highlighted in yellow in both the main manuscript and supplementary files. Our point-by-point responses are detailed below.

Comment 1:

1. 8i: The in vivo CD8 depletion antibody experiment indicates that after $CD8^+$ cell depletion, there remains a substantial gap in tumor growth between the COF Tpy Se Cu-OVAD group and both the CD8 depletion-only group and the control group (although no statistical difference is shown). Furthermore, previous COF Tpy Se Cu-OVAD treatment experiments (7e-7i) showed changes in $CD25^+FOXP3^+$ cells. Does this suggest that $CD4^+$ cells also play a role? It is necessary to evaluate tumor rescue in the COF Tpy Se Cu-OVAD group by using $CD8^+$ and $CD4^+$ depletion antibodies, both alone and in combination.

Response 1:

We sincerely thank the reviewer for this insightful and constructive suggestion, which has allowed us to systematically delineate the respective contributions of $CD8^+$ and $CD4^+$ T cell subsets to the antitumor efficacy of the combination regimen. In response to this comment, and to address the reviewer's concern as directly as possible, we have performed additional in vivo antibody-mediated depletion experiments, including $CD4^+$ T cell depletion alone, combined $CD4^+/CD8^+$ T cell depletion, and a mouse IgG2b isotype control as the negative control group, in both the 4T1-R subcutaneous and MOC1-R orthotopic tumor models. Depletion efficiency was validated by flow cytometry. All newly generated data have been incorporated into the revised manuscript and presented in Supplementary Fig. 38.

Our key findings are summarized below:

(1) $CD8^+$ T cell depletion significantly attenuated the antitumor effect of the triple combination regimen, confirming that $CD8^+$ effector T cells serve as the central mediators of the immune-driven antitumor activity induced by the combined treatment.

(2) Notably, a partial tumor growth inhibitory effect was retained after $CD8^+$ T cell depletion. We attribute this residual effect to the direct cytotoxicity of COF-Tpy-Se-Cu plus irradiation, which triggers FDX1-dependent cuproptosis in tumor cells in a T cell–independent manner. This direct tumoricidal effect is independent of adaptive immunity and therefore cannot be abrogated by T cell depletion.

(3) In contrast, $CD4^+$ T cell depletion alone did not significantly alter the overall tumor growth inhibition induced by the combination regimen. Furthermore, combined depletion of $CD4^+$ and $CD8^+$ T cells did not yield a therapeutic outcome significantly different from that observed with $CD8^+$ T cell depletion alone.

We attribute the lack of a significant net effect upon pan-$CD4^+$ T cell depletion to the functional heterogeneity of $CD4^+$ T cell populations within the tumor microenvironment. As the reviewer correctly noted, our previous data demonstrated that the combination regimen significantly reduces the proportion of immunosuppressive $CD4^+CD25^+FOXP3^+$ regulatory T cells (Tregs) in tumors (Fig. 7l). Bulk $CD4^+$ T cell depletion simultaneously eliminates two $CD4^+$ subsets with opposing functions: conventional $CD4^+$ type 1 helper T (Th1) cells support $CD8^+$ T cell priming, clonal expansion, and memory formation, such that their depletion would be expected to attenuate therapeutic efficacy. By contrast, $CD4^+$ Tregs exert immunosuppressive effects and constrain antitumor immunity, such that their depletion would be expected to enhance therapeutic efficacy.

These two countervailing effects offset each other upon bulk $CD4^+$ T cell depletion, leading to no net significant change in short-term tumor growth. In addition, our combination regimen induces robust release of damage-associated molecular patterns (DAMPs) and strongly promotes dendritic cell (DC) maturation, thereby providing sufficient co-stimulatory signals to drive $CD8^+$ T cell activation.

It should be noted that $CD4^+$ T cells are well recognized to play a critical role in the establishment and long-term maintenance of $CD8^+$ T cell memory. Although $CD4^+$ T cell depletion did not significantly impair acute tumor growth control under our experimental conditions, their established contribution to durable immunological

memory is consistent with our observation of expanded memory T cell populations in the combination regimen.

All corresponding data have been incorporated into the revised manuscript and supplementary files. We have also included a detailed discussion of this point in the revised Discussion section, and we hope this additional clarification adequately addresses the reviewer's concern.

The corresponding text in the manuscript has been revised as follows:

"To delineate the respective contributions of $CD8^+$ and $CD4^+$ T cells to the observed therapeutic efficacy, we performed in vivo antibody-mediated cell depletion during treatment (Fig. 8i and Supplementary Fig. 38a-f). $CD8^+$ T cell depletion significantly attenuated the antitumor effect of the triple regimen. By contrast, $CD4^+$ T cell depletion alone did not significantly alter treatment efficacy (Fig. 8i and Supplementary Fig. 38a-f)."

Specifically in the "Methods" section:

"To assess the role of adaptive immunity in antitumor responses induced by the COF-Tpy-Se-Cu + IR + OVAD combination regimen, a separate cohort of 4T1-R tumor–bearing mice received intraperitoneal injections of anti-CD8α antibody (Bio X Cell, BE0061, NH, USA, 10 mg kg^{-1}), anti-CD4 antibody (Bio X Cell, BP0003-1, 10 mg kg^{-1}), or IgG2b isotype control antibody (Bio X Cell, BP0090, 10 mg kg^{-1})."

Specifically in the "Discussion" section:

"Depletion of $CD8^+$ T cells significantly attenuated the therapeutic efficacy, confirming $CD8^+$ effector T cells as the central mediators of the immune-driven antitumor response. Notable residual tumor growth inhibition was retained after $CD8^+$ T cell depletion, which we attribute to the direct, T cell–independent cytotoxicity of radiation-triggered copper release from COF-Tpy-Se-Cu, which induces FDX1-mediated cuproptosis in tumor cells irrespective of adaptive immunity. In contrast, pan-$CD4^+$ T cell depletion alone did not significantly alter the overall antitumor effect,

and combined depletion of $CD4^+$ and $CD8^+$ T cells yielded outcomes comparable to $CD8^+$ T cell depletion alone. This lack of net effect upon bulk CD4 depletion reflects the functional heterogeneity of $CD4^+$ T cell subsets in the tumor microenvironment. As also evidenced by our observation of reduced intratumoral Treg frequency, pan-CD4 depletion simultaneously eliminates two functionally opposing populations: conventional type 1 helper T (Th1) cells that support $CD8^+$ T cell priming and clonal expansion, and immunosuppressive $CD4^+CD25^+FOXP3^+$ regulatory T cells that constrain antitumor immunity. These two countervailing effects offset each other, resulting in no significant net change in short-term tumor growth control. Additionally, the robust release of DAMPs and strong DC maturation induced by the combination regimen provide sufficient co-stimulatory signals to drive $CD8^+$ T cell activation. It is worth noting that although $CD4^+$ T cell depletion did not significantly impair acute tumor growth control in our model, $CD4^+$ T cells are well recognized to support the long-term maintenance of $CD8^+$ T cell memory. This established function is consistent with our observation of expanded memory T cell populations and prolonged antitumor protection in the combination regimen."

i

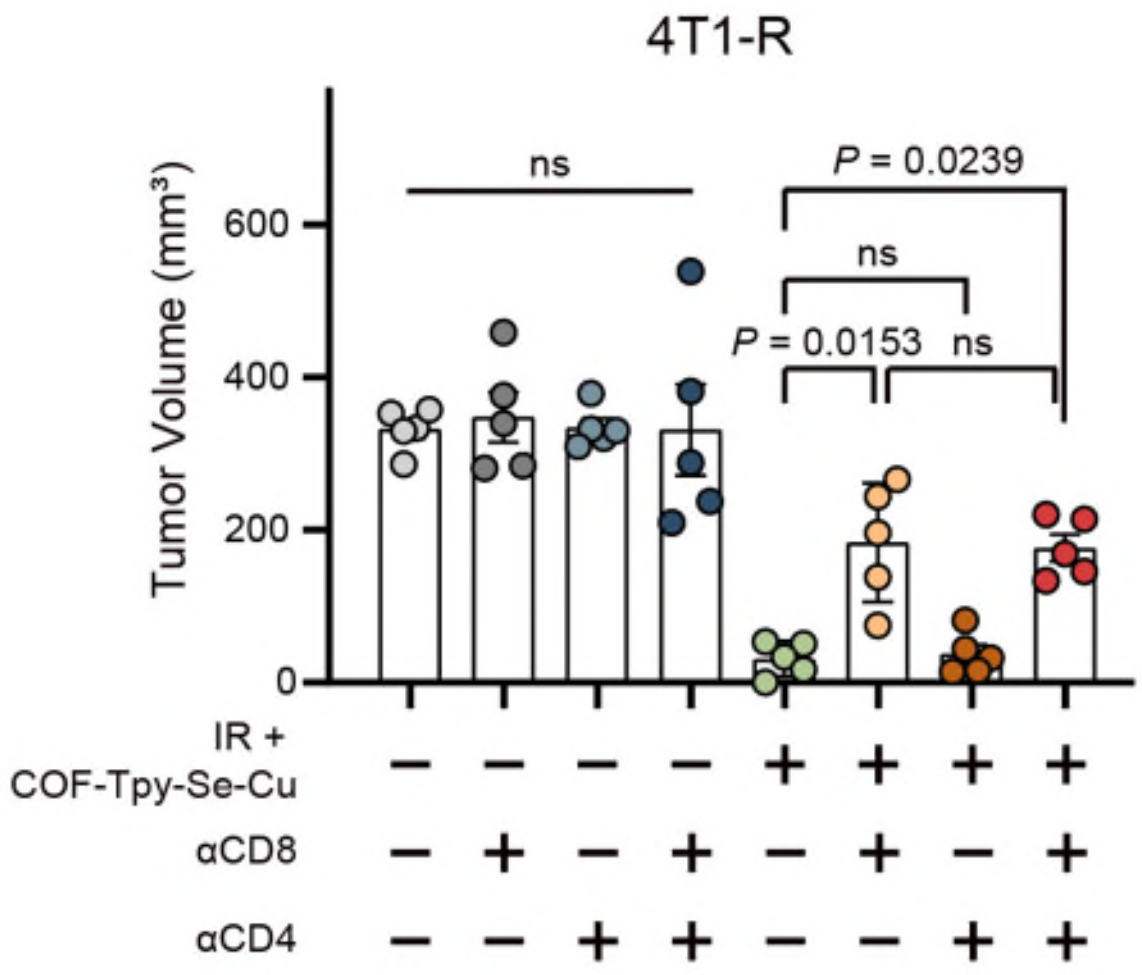

Fig. 8| i Tumor volume for the indicated treatment groups (n = 5 mice per group). Statistical significance was assessed using one-way ANOVA with Tukey's multiple-comparisons test (**i**). Data are presented as mean ± s.e.m.

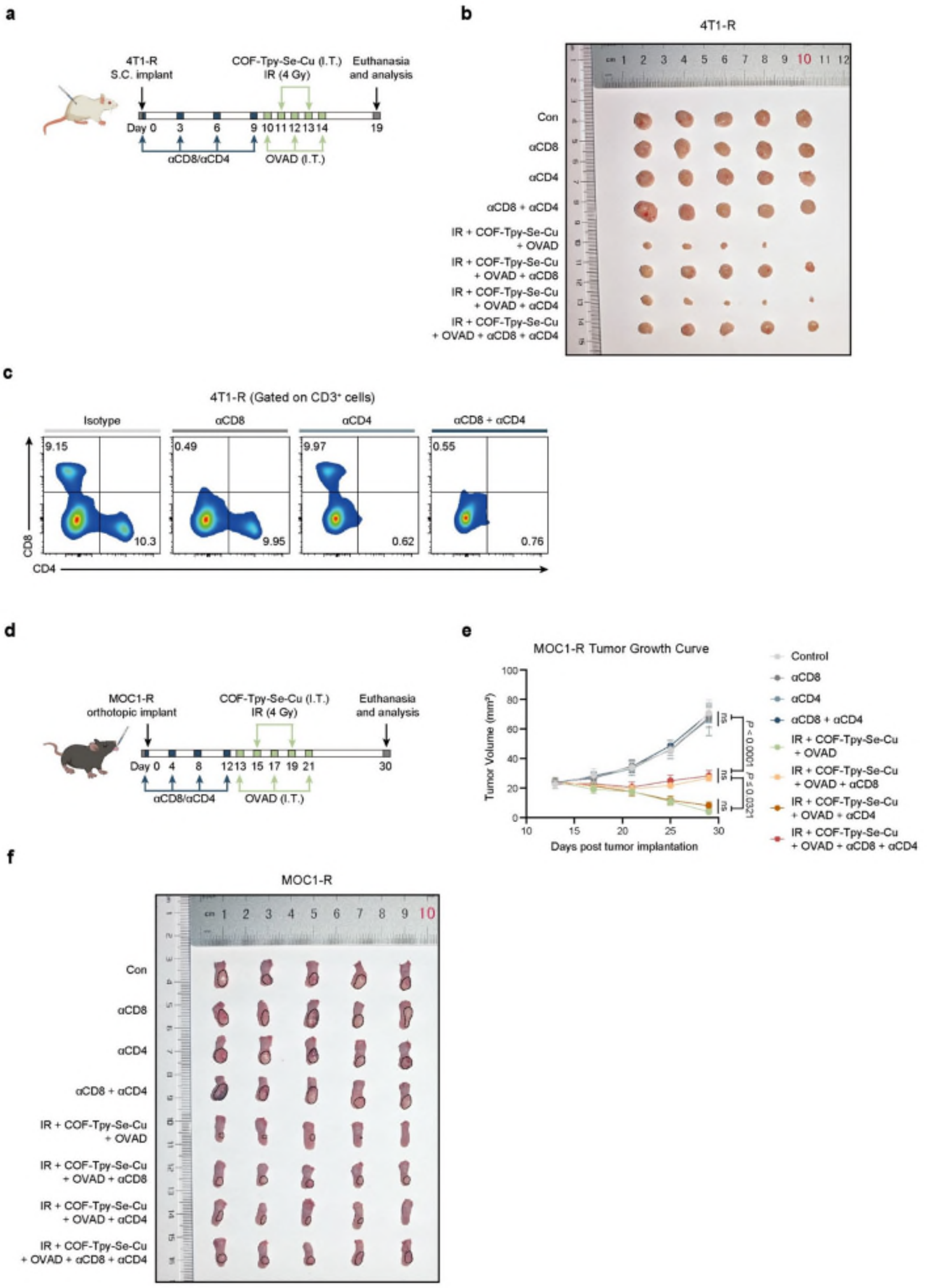

Supplementary Fig. 38| Antitumor efficacy of the combination regimen is dependent on CD8⁺ T cells in radioresistant tumor models. a Schematic of the T cell depletion experiment in the 4T1-R subcutaneous tumor model. Mice received intratumoral combination therapy with or without intraperitoneal injection of anti-CD8α antibody (10 mg kg^{-1}), anti-CD4 depleting antibody (10 mg kg^{-1}), the combination of both, or IgG isotype control antibody (10 mg kg^{-1}) at the indicated time points. **b** Representative images of excised subcutaneous 4T1-R tumors from each treatment group at the endpoint (n = 5 mice per group). **c** Flow cytometric validation of $CD8^+$ and $CD4^+$ T cell depletion efficiency in 4T1-R tumor tissues (gated on $CD3^+$ T cells), with isotype antibody serving as the negative control. **d** Schematic of the T cell depletion experiment in the MOC1-R orthotopic tumor model. Mice received the combination treatment along with anti-CD8α (10 mg kg^{-1}, i.p.),

anti-CD4 depleting antibody (10 mg kg^{-1}, i.p.), the combination of both, or IgG isotype control antibody (10 mg kg^{-1}, i.p.) at the indicated time points. **e** Tumor growth curves of MOC1-R orthotopic xenografts across the indicated treatment and T cell depletion groups (n = 5 mice per group). **f** Representative images of excised tongues from mice bearing orthotopic MOC1-R tumors at the endpoint. Tumor boundaries are delineated by black dashed lines. Statistical significance of tumor growth curves was assessed by two-way ANOVA (**e**). Data are presented as mean ± s.e.m.

Comment 2:

2. 8h: Quantify the number of metastatic foci per group.

Response 2:

We sincerely appreciate the reviewer's valuable suggestion, which has enabled us to strengthen the quantitative rigor of our metastasis analysis. To address this point, we have now complemented the histological presentation with quantitative enumeration of pulmonary metastatic nodules across treatment groups, and adjusted the image magnification to include both low-magnification panoramic views (to show overall metastatic burden) and high-magnification insets (to detail nodule morphology and the surrounding pulmonary parenchymal microenvironment).

Specifically in the manuscript:

"Furthermore, in an experimental lung colonization model, the triple combination regimen significantly attenuated pulmonary metastatic burden compared with the untreated control, and exhibited the most potent antimetastatic activity across all tested treatment groups (Fig. 8f–h and Supplementary Fig. 37k)."

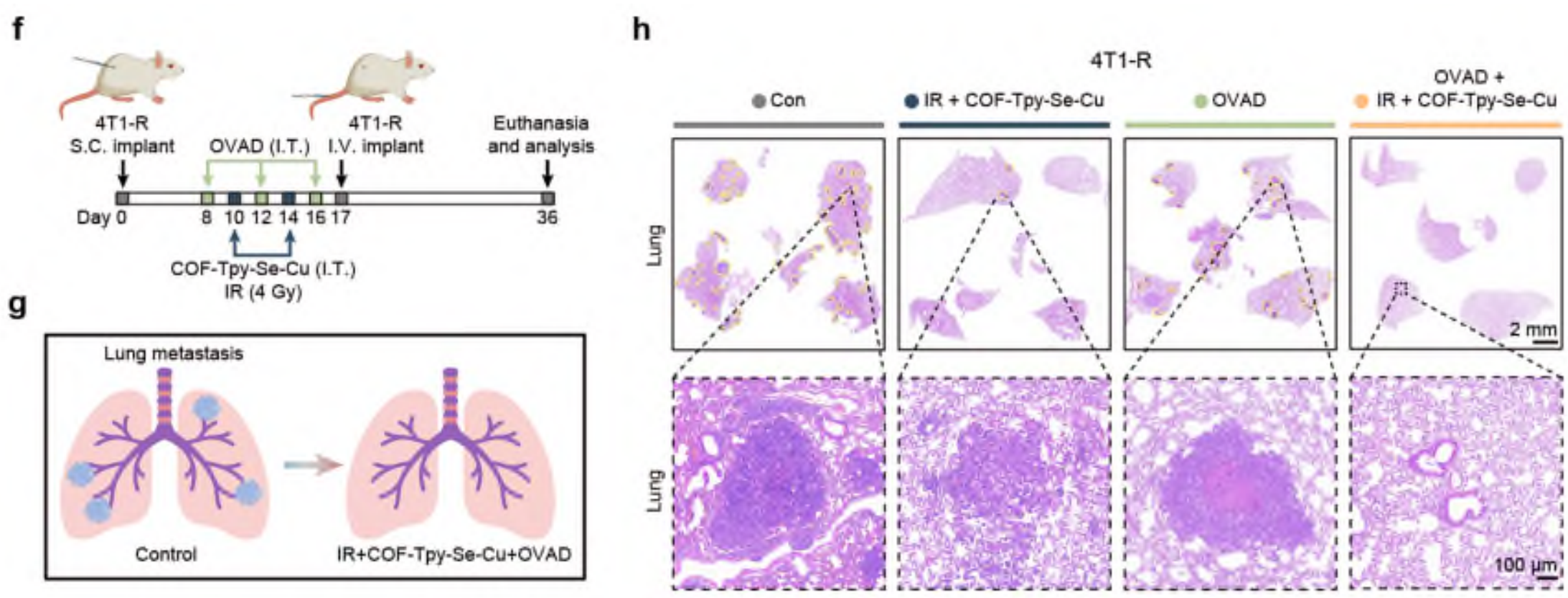

Fig. 8| f–h Experimental lung colonization model. (f, g) Schematic of the

experimental design for assessing pulmonary metastatic burden. (h) Representative H&E-stained lung sections showing metastatic nodules across treatment groups (scale bar, 100 μm).

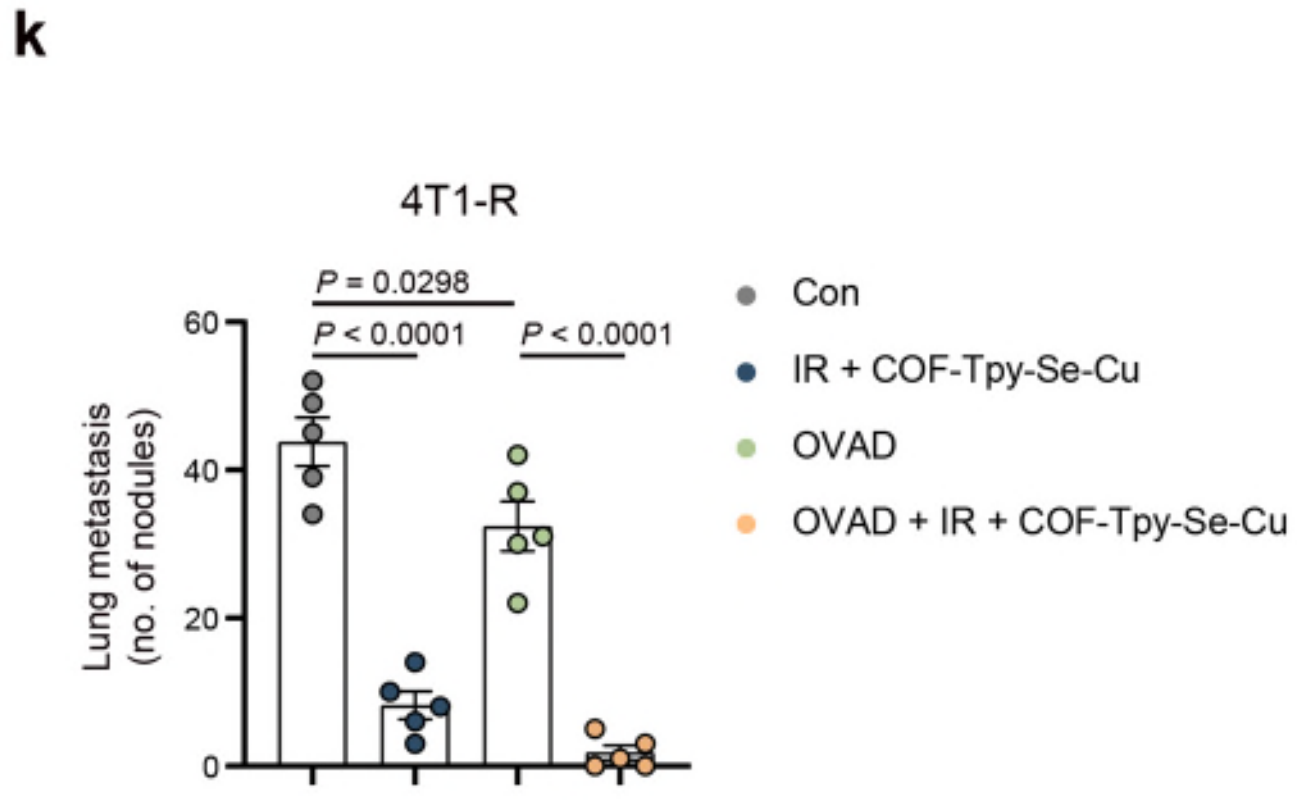

Supplementary Fig. 37| k Quantification of pulmonary metastatic nodules across treatment groups. Statistical significance was assessed using one-way ANOVA with Tukey's multiple-comparisons test (**k**). Data are presented as mean ± s.e.m.

Comment 3:

3. S13: In the flow cytometry gating strategy for immune-cell profiling, label the proportion of each population. Also, show the initial population (prior to doublet exclusion). It is perplexing that the proportion of $CD3^+$ T cells appears to account for over 50% of $CD45^+$ cells, while $CD11b^+$ myeloid cells also represent far more than 50% of $CD45^+$ cells. Please provide a reasonable explanation.

Response 3:

We sincerely thank the reviewer for raising this important point regarding the flow cytometry gating strategies. We apologize for the ambiguity caused by our previous presentation, in which gating plots derived from different tissue sources were consolidated into a single figure for compactness, resulting in confusion regarding cell population proportions.

The apparent discrepancy in $CD45^+$ subset proportions stems from the distinct tissue origins of the samples in the original composite figure. $CD3^+$ T cell profiling was performed on cells isolated from tumor-draining lymph nodes (TDLNs), where T cells constitute the dominant leukocyte population and routinely account for more than 50% of $CD45^+$ cells. In contrast, $CD11b^+$ myeloid cell profiling was performed on cells

isolated from tumor tissues, where myeloid cells represent the predominant leukocyte compartment and accordingly also exceed 50% of CD45$^+$ cells. These distribution patterns are consistent with the well-established immunological characteristics of secondary lymphoid organs and the tumor microenvironment.

In the revised manuscript, we have separated the gating strategies into four independent supplementary figures (Supplementary Figs. 16–19; formerly Supplementary Fig. 13), each dedicated to one immune cell population from its corresponding tissue source:

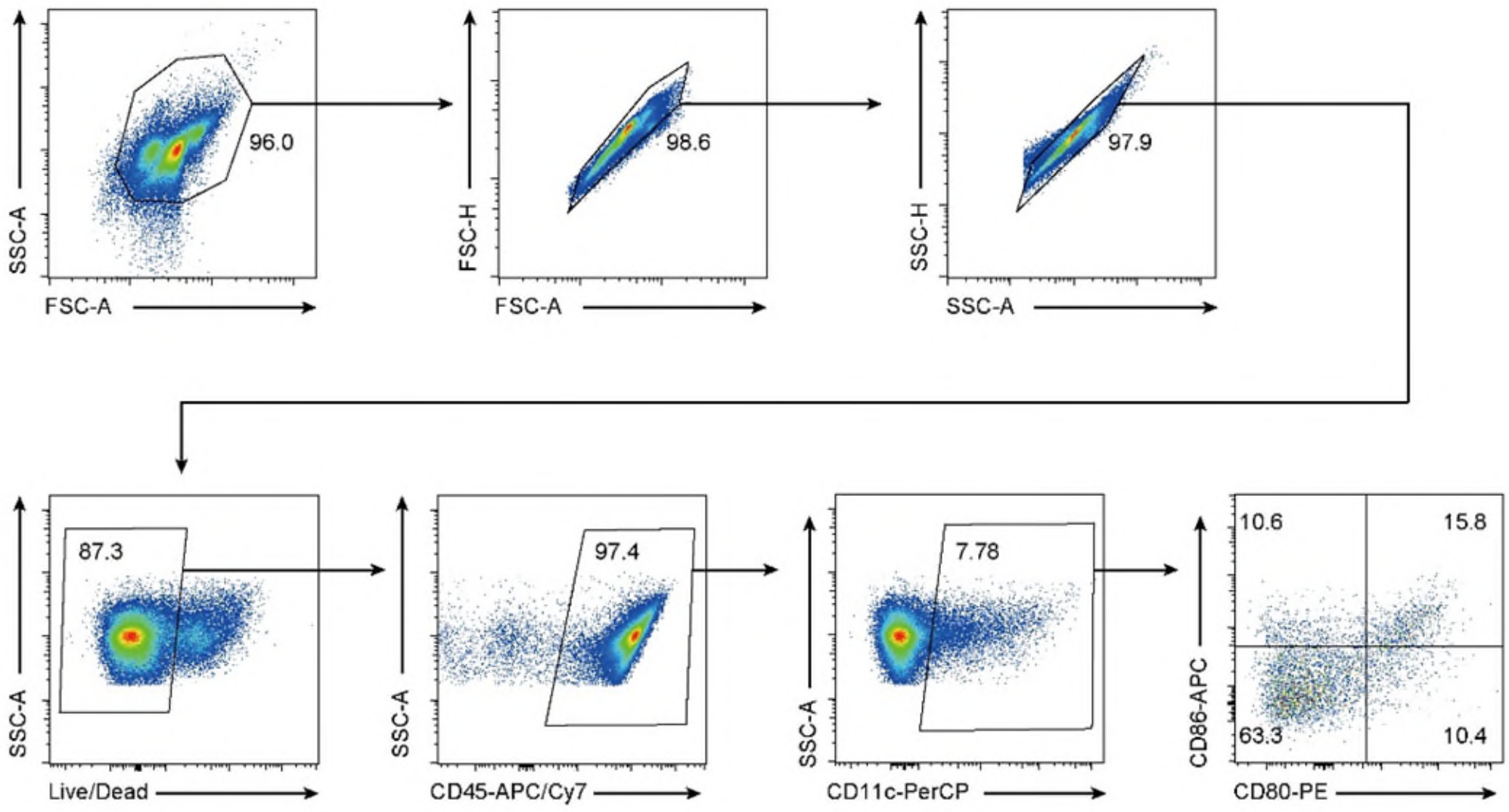

Supplementary Fig. 16| Flow-cytometry gating strategy of dendritic cells (DCs) in tumor-draining lymph nodes (TDLNs).

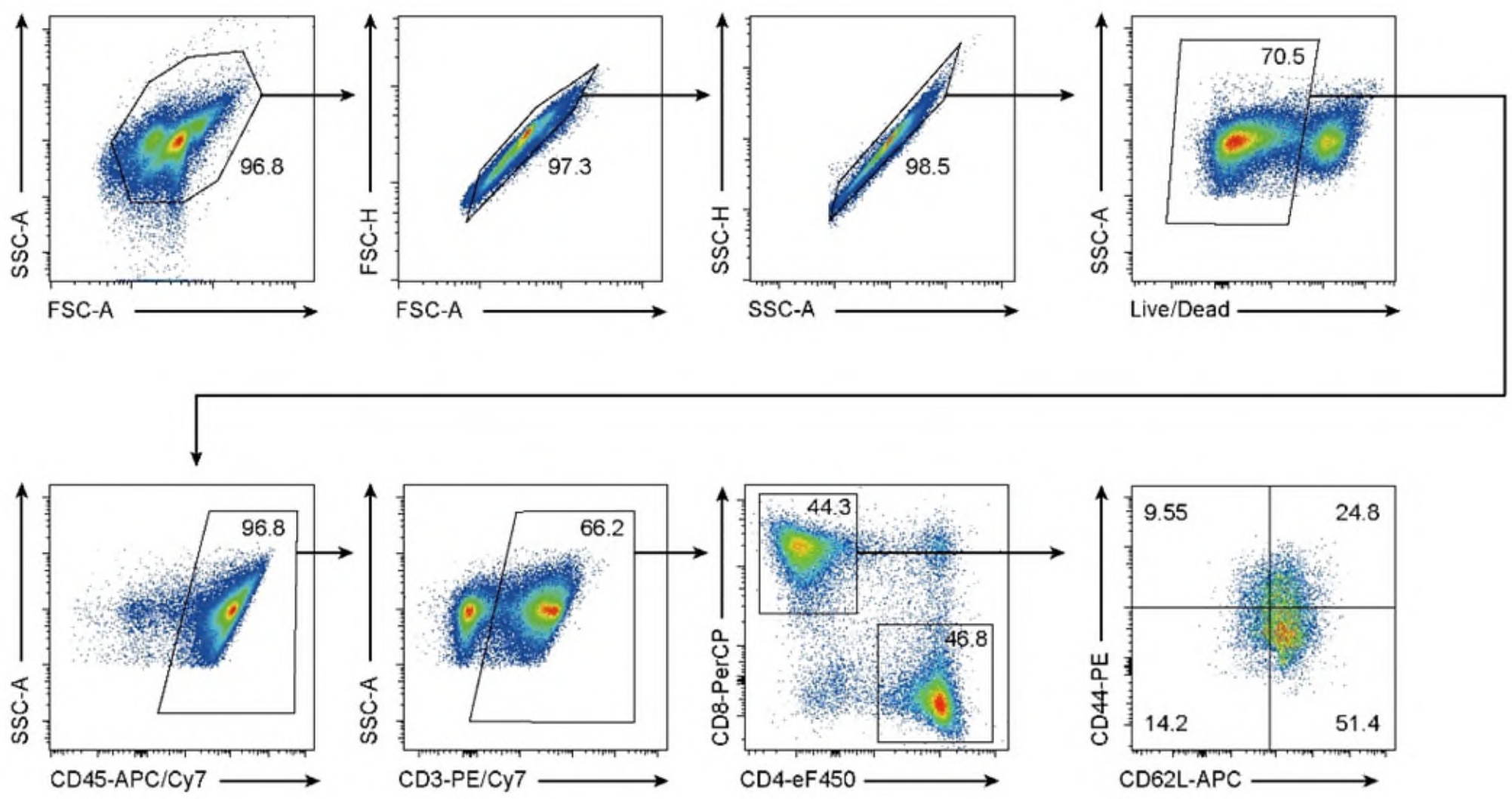

Supplementary Fig. 17| Flow-cytometry gating strategy of memory T cells in

tumor-draining lymph nodes (TDLNs).

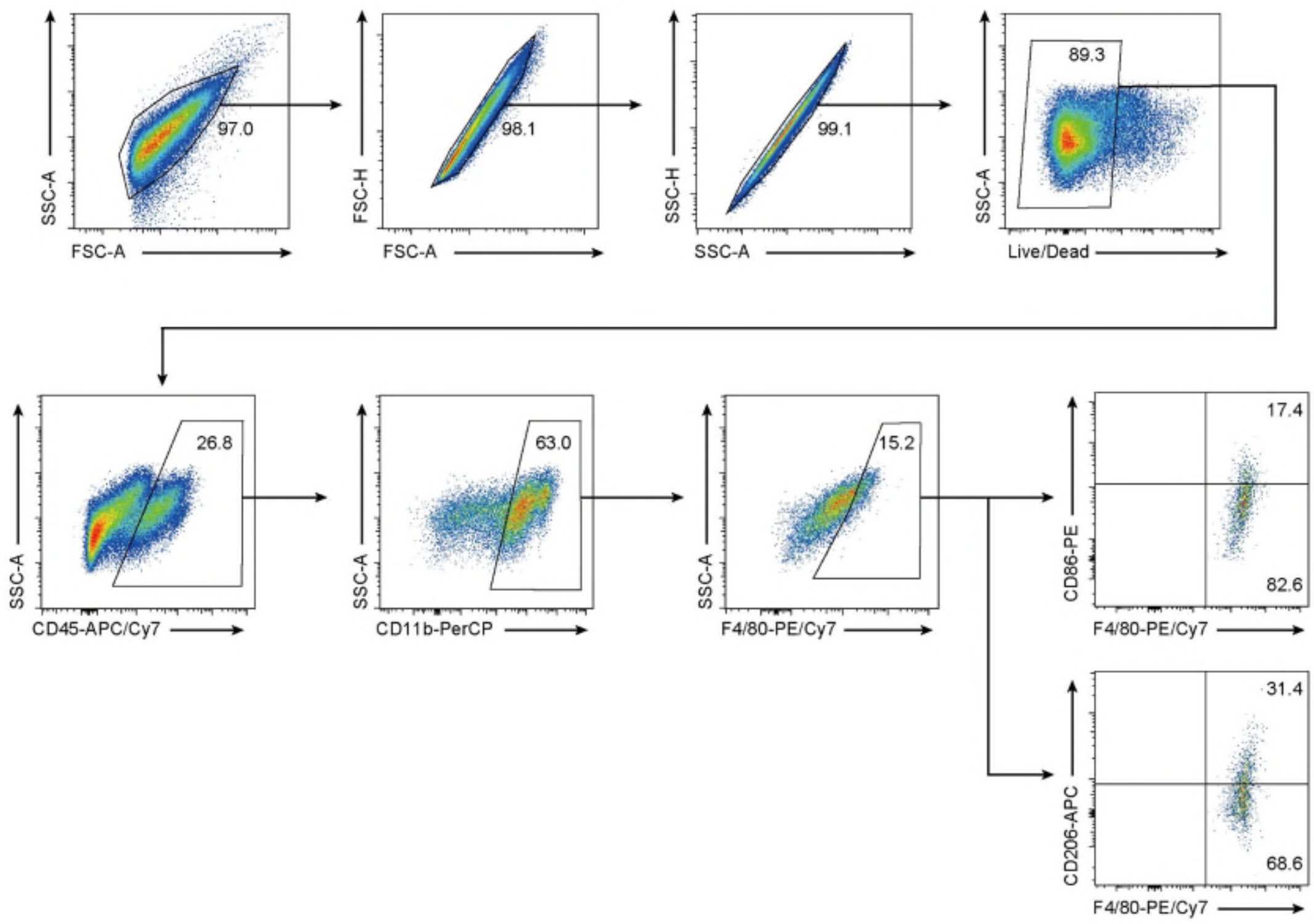

Supplementary Fig. 18| Flow-cytometry gating strategy of tumor-associated macrophages (TAMs) in tumor tissues.

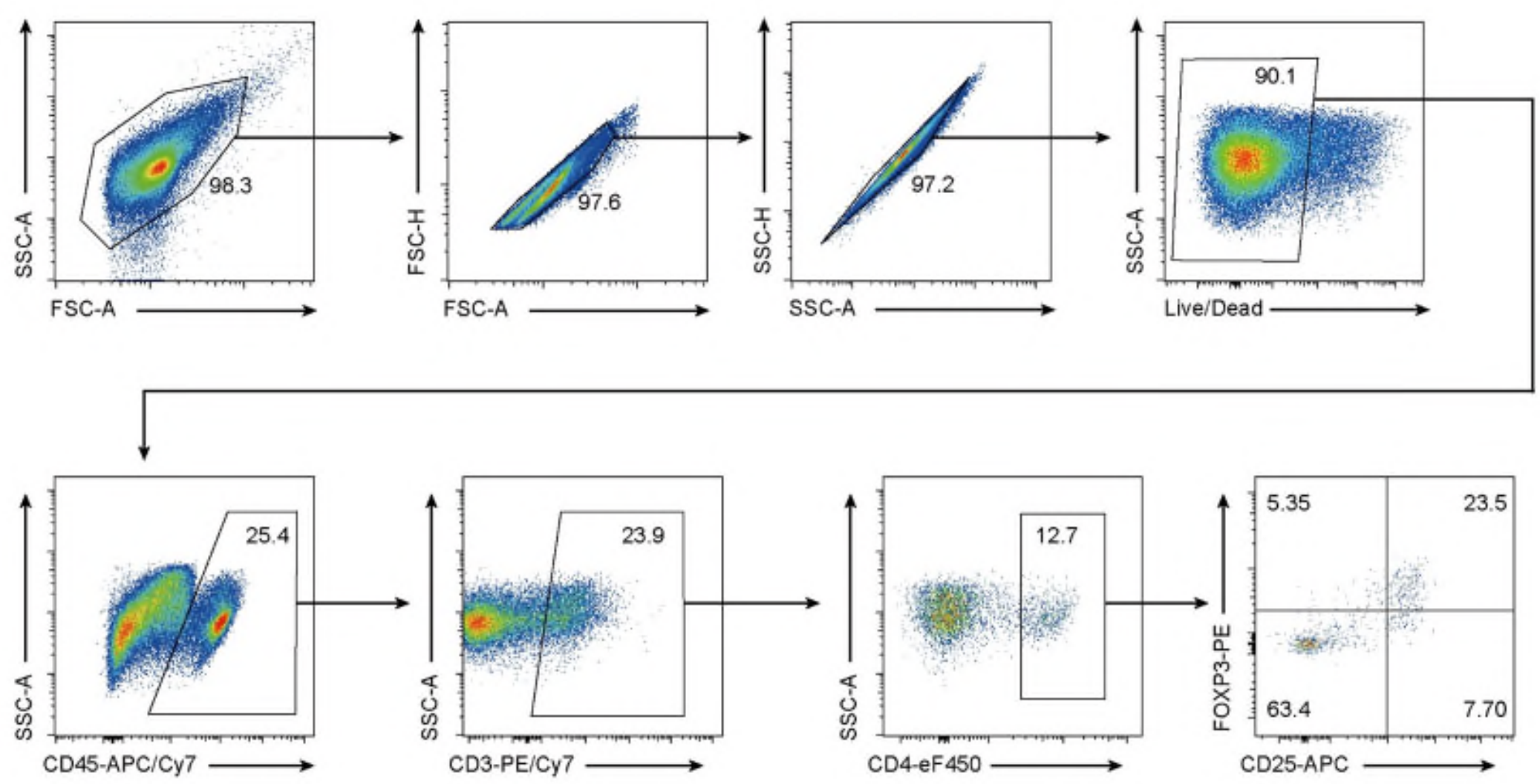

Supplementary Fig. 19| Flow-cytometry gating strategy of regulatory T cells (Tregs) in tumor tissues.

For each gating panel, we have labeled the percentage of the gated population relative to its parent population, and we have included the initial FSC-A/SSC-A population prior to doublet exclusion to illustrate the full gating hierarchy. We respectfully hope that these revisions adequately address the reviewer's concern and substantially improve the clarity and rigor of our flow cytometry data presentation.

Comment 4:

4. S23: Clearly indicate in the flow cytometry plots which parent cell population each gated population was derived from.

Response 4:

We sincerely thank the reviewer for this valuable suggestion to improve the clarity of our flow cytometry data presentation, and we apologize for the insufficient annotation in the previous version.

We have revised Supplementary Fig. 35 (previously Supplementary Fig. 23) and updated its figure legend to explicitly state the parent cell population for each gated subset. The detailed gating hierarchies are specified below:

Panel a shows $EGFP^+$ virus-infected cells, gated from the $CD45^-$ tumor cell population.

Panel b shows $CD80^+CD86^+$ mature dendritic cells, gated from $CD11c^+$ dendritic cells isolated from tumor-draining lymph nodes (TDLNs).

Panel c shows $CD8^+$ and $CD4^+$ T cell subpopulations, gated from $CD3^+$ T cells within tumor tissue.

Panel f shows $CD44^+CD62L^+$ memory T cells, gated from $CD8^+$ T cells in TDLNs.

The revised full figure legend is provided below:

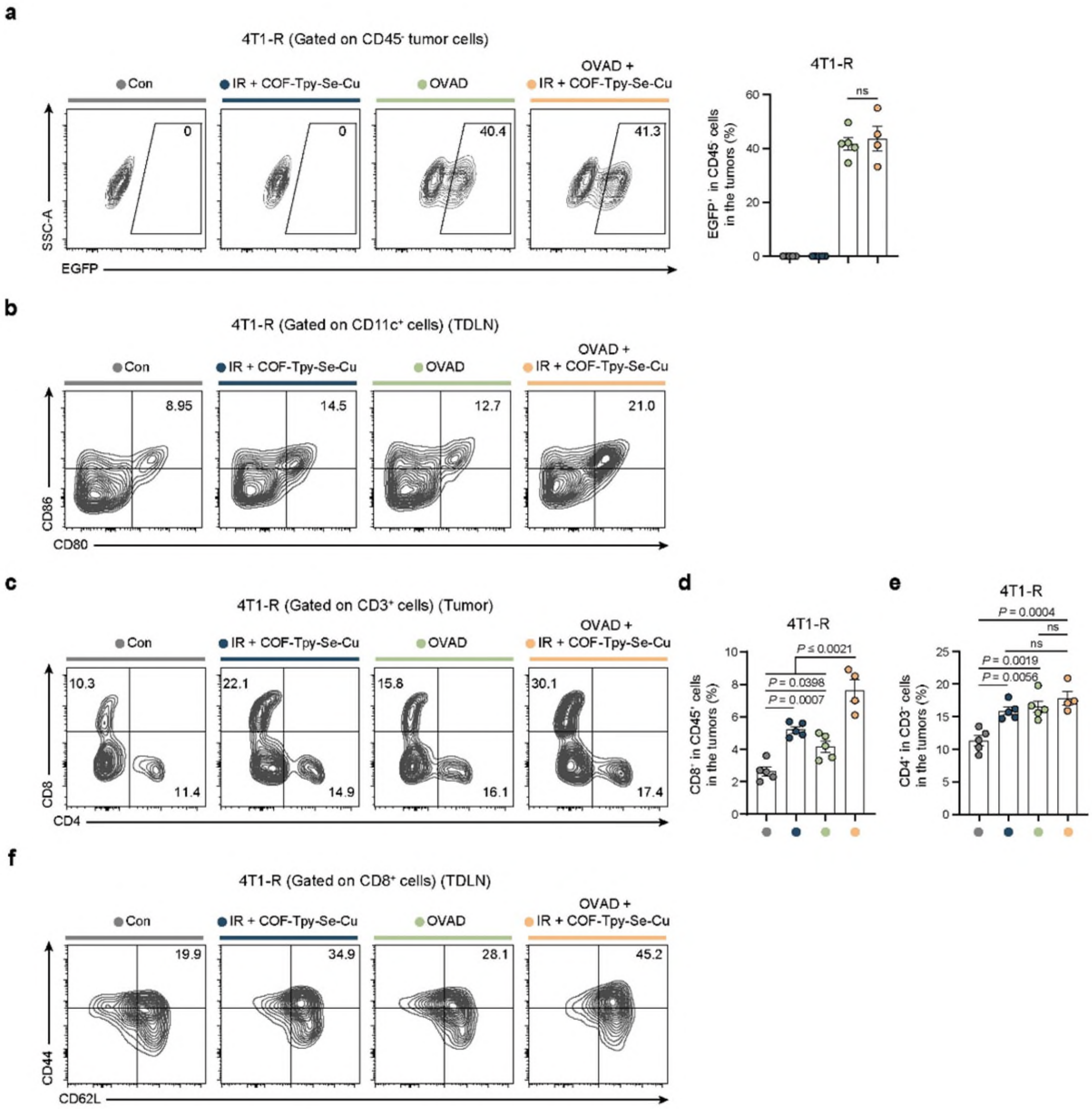

Supplementary Fig. 35| Combination therapy remodels the tumor immune microenvironment. a Flow cytometric assessment of intratumoral OVAD infection efficiency, quantified as the percentage of EGFP-positive cells among CD45⁻ tumor cells in 4T1-R xenografts across the indicated treatment groups. **b** Representative plots showing the frequency of CD80⁺CD86⁺ cells among CD11c⁺ DCs in tumor-draining lymph nodes (TDLNs) from 4T1-R models. **c** Flow cytometric analysis of tumor-infiltrating CD8⁺ T and CD4⁺ T cells among CD3⁺ T cells from 4T1-R models. **d** Quantification of CD8⁺ T cell frequency among CD45⁺ leukocytes. **e** Quantification of CD4⁺ T cell frequency among CD3⁺ T cells. **f** Frequency of CD44⁺CD62L⁺ memory T cells among CD8⁺ T cells in TDLNs from 4T1-R models. Statistical significance was assessed using one-way ANOVA with Tukey's multiple-comparisons test (**a, d, e**). Data are presented as mean ± s.e.m. from at least three independent experiments.

Comment 5:

5. S24: Please specify which cell group the gate was applied to. Are the displayed cells macrophages positive for CD86 and CD206? If so, why are there also a small proportion of F4/80^{-} cells?

<u>Response 5:</u>

We sincerely thank the reviewer for pointing out the ambiguous gating presentation in our flow cytometry plots and for raising this valuable question. We apologize for the confusion caused by the insufficient annotation of parent populations in the original figure.

First, we clarify that all quantitative analyses of CD86^{+} M1-like and CD206^{+} M2-like tumor-associated macrophages (TAMs) were performed exclusively on the F4/80^{+} parent population. The F4/80-positive gate was rigorously defined using the corresponding fluorescence-minus-one (FMO) control to objectively distinguish specific staining from background autofluorescence. No F4/80^{-} events were included in any statistical calculations, and all quantitative conclusions remain robust and unaltered.

To minimize potential interpretive ambiguity, we have revised Supplementary Fig. 36 (previously Supplementary Fig. 24) and updated its legend to clearly define the parent cell population for every gated subset. In addition, a full stepwise flow cytometry gating workflow for tumor-associated macrophage identification is provided independently in Supplementary Fig. 18 to document our analytical pipeline with full transparency.

The revised figure is presented below:

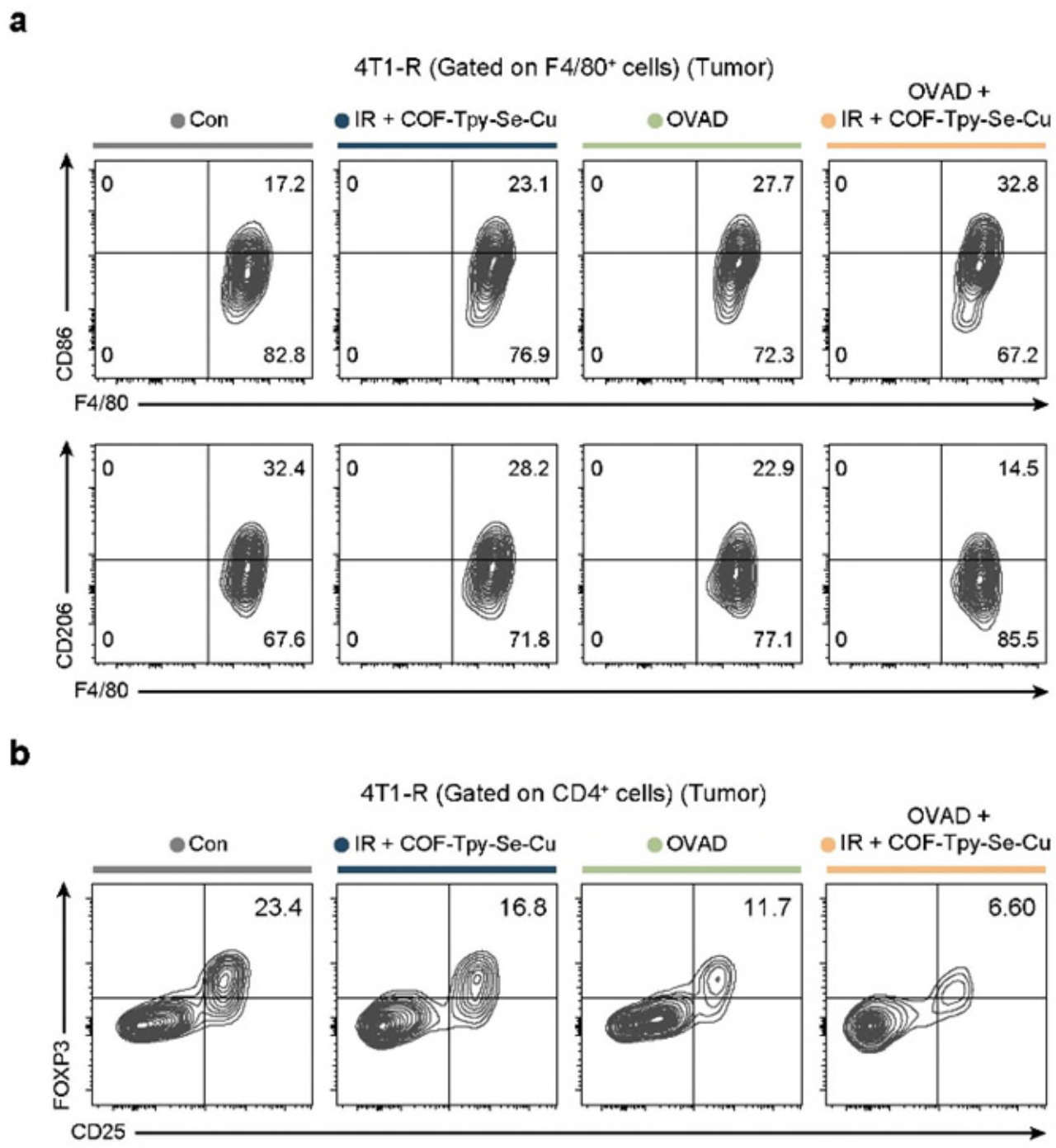

Supplementary Fig. 36| Combination therapy remodels the tumor immune microenvironment. a Representative flow cytometric plots of M1-like ($CD86^+$) and M2-like ($CD206^+$) tumor-associated macrophages (TAMs), gated on F4/80^+ cells, in 4T1-R tumor tissues across the indicated treatment groups. **b** Representative flow cytometric plots of $CD25^+FOXP3^+$ regulatory T cells (Tregs) among $CD4^+$ cells in 4T1-R tumor tissues.

<u>Comment 6:</u>

6. 446-449: It is difficult to determine from Figure 6d that protein lipoylation is enhanced in the OVAD group, as FDX levels are also markedly elevated in the OVAD group. Further detailed investigation using IP or other experiments is recommended.

<u>Response 6:</u>

We sincerely thank the reviewer for raising this important point and for the helpful experimental suggestion. We fully agree that immunoblotting of whole-cell lysates cannot distinguish whether an increased lipoylation signal stems from elevated total protein abundance or from enhanced post-translational lipoylation of individual protein molecules. To address this concern more rigorously, we selected DLAT, the most well-characterized lipoylated target and a core effector of cuproptosis, as the representative target for co-immunoprecipitation (co-IP) validation.

We immunoprecipitated the core lipoylated TCA cycle protein DLAT from cell lysates using an anti-DLAT antibody, and then immunoblotted the precipitates with an anti-lipoic acid antibody to quantify the fraction of lipoylated DLAT. Equivalent IP input across samples was verified by parallel immunoblotting of the same immunoprecipitated fractions with an anti-DLAT antibody.

Our results show that OVAD treatment markedly increases the abundance of lipoylated DLAT in the immunoprecipitates, whereas total DLAT protein levels remain unchanged (Supplementary Fig. 29b). This demonstrates that OVAD specifically increases DLAT lipoylation rather than merely increasing total DLAT expression. These data support a model in which OVAD-mediated FDX1 stabilization functionally increases its enzymatic activity, leading to enhanced lipoylation of downstream target proteins.

We have added these data and the corresponding interpretation to the revised manuscript:

"We then examined the effects of OVAD on core regulators of the cuproptosis pathway. OVAD infection increased FDX1 protein abundance and enhanced DLAT lipoylation in radioresistant cells (Fig. 6d). To determine whether the elevated lipoylation signal arose from increased total protein levels or enhanced FDX1-mediated post-translational modification, we immunoprecipitated DLAT and immunoblotted with an anti-lipoic acid antibody. OVAD treatment markedly increased the abundance of lipoylated DLAT in the immunoprecipitates, whereas total DLAT protein levels remained unchanged (Supplementary Fig. 29b). This result indicates that OVAD increases FDX1 protein abundance, which in turn enhances DLAT lipoylation and may thereby modulate cellular sensitivity to cuproptosis."

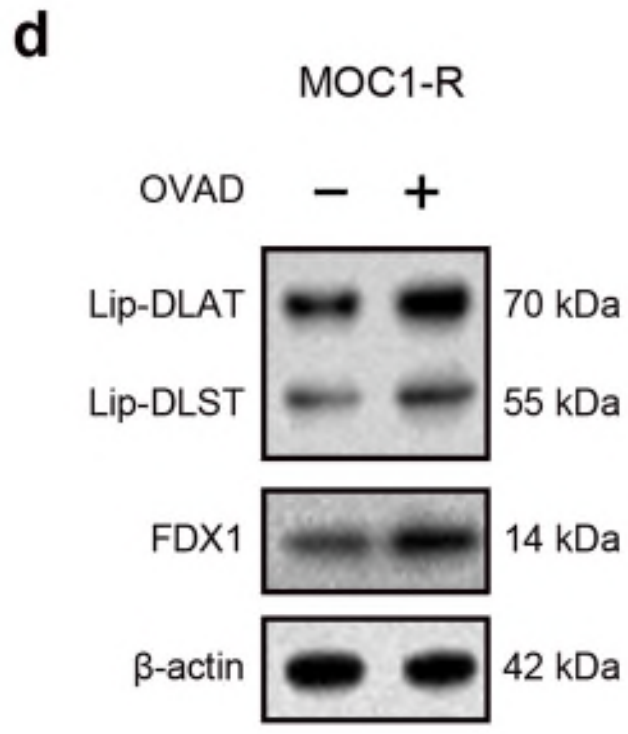

Fig. 6| d Immunoblot analysis of lipoylated DLAT, lipoylated DLST, and FDX1 in MOC1-R cells after 12 h OVAD treatment (MOI = 1). β-actin served as a loading control.

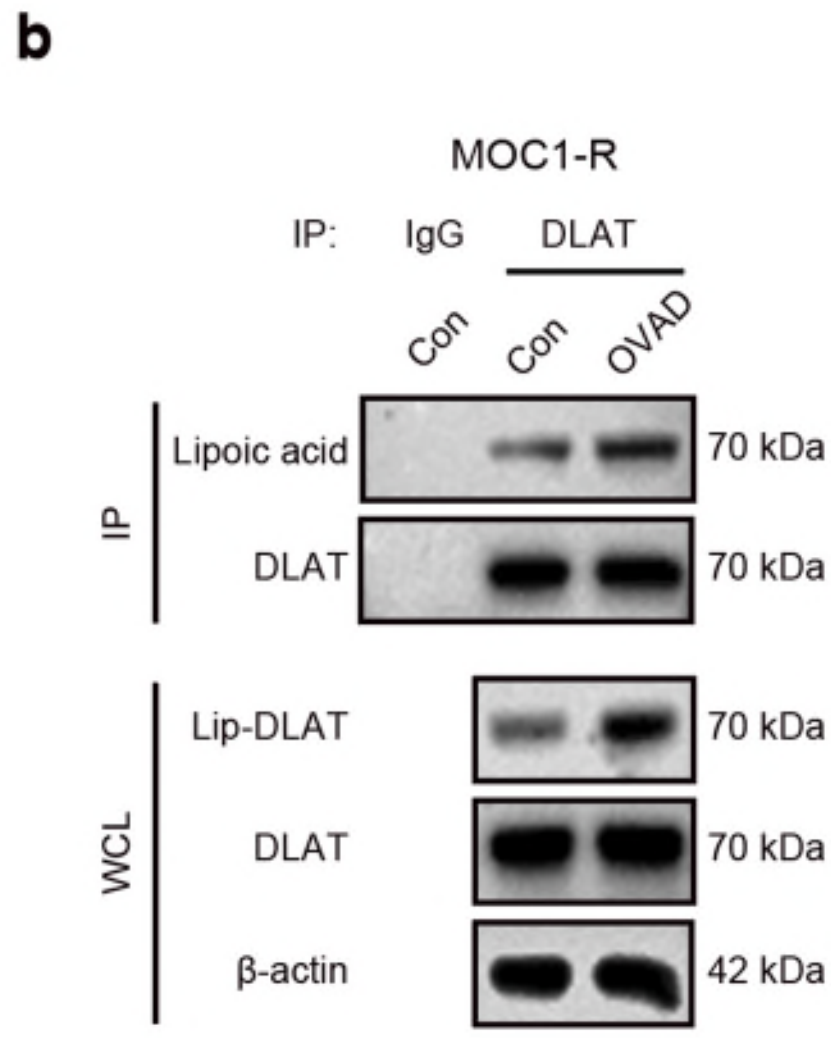

Supplementary Fig. 29| b Co-immunoprecipitation (IP) analysis of DLAT lipoylation in MOC1-R cells with or without OVAD infection. DLAT was immunoprecipitated and immunoblotted with anti-lipoic acid antibody to detect lipoylated DLAT (Lip-DLAT), and with anti-DLAT antibody to confirm equal IP input. Whole-cell lysates (WCL) were analyzed in parallel. β-actin served as the loading control.

Comment 7:

7. Evidence is required to demonstrate that, following intratumoral injection, OVAD effectively infects tumor cells, mediates tumor cell lysis, and undergoes amplification within the tumor tissue.

Response 7:

We sincerely thank the reviewer for this important comment. We fully agree that more direct evidence was required to support intratumoral OVAD infection, replication,

and oncolytic activity, and this comment prompted us to further validate these processes following intratumoral administration.

To address this point, we performed time-course analyses of OVAD infection and oncolytic activity in both the MOC1-R orthotopic and 4T1-R subcutaneous tumor models after local intratumoral injection. Multiple complementary lines of evidence support effective intratumoral viral amplification and tumor cell lysis:

(1) Tumor cell infection efficiency

Leveraging the EGFP reporter encoded in the OVAD genome, we tracked viral infection by gating on $CD45^{-}$ tumor cells over time. In both models, we observed a progressive, time-dependent increase in the percentage of EGFP-positive tumor cells, indicating sustained viral spread within tumor tissue (Supplementary Fig. 31a).

(2) Active viral replication

Immunoblot analysis of tumor tissue lysates revealed gradual accumulation of the adenoviral E1A protein, an essential early regulator of adenoviral genome replication, supporting active viral genome replication and de novo viral protein synthesis in vivo (Supplementary Fig. 31b).

(3) Virus-mediated tumor cell lysis

In parallel, we detected elevated levels of cleaved caspase-3 and increased release of HMGB1 in tumor tissues, consistent with virus-induced tumor cell death and the release of damage-associated molecular patterns (Supplementary Fig. 31b).

All corresponding data have been added to the revised manuscript and are presented in Supplementary Fig. 31. The relevant section in the main text has been updated as follows:

"We next examined the intratumoral infectivity and replicative capacity of OVAD in vivo. By flow cytometric analysis gating on the $CD45^{-}$ tumor cell population, we observed a time-dependent increase in the frequency of EGFP-positive cells in both MOC1-R and 4T1-R models, indicating that the virus progressively infects more tumor cells and spreads within the tumor parenchyma after intratumoral injection (Supplementary Fig. 31a). In line with this, immunoblot analysis revealed a gradual

increase in the viral E1A protein, a marker of active adenoviral replication, supporting ongoing viral replication within tumors (Supplementary Fig. 31b). Meanwhile, we detected elevated cleaved caspase-3 and decreased intracellular HMGB1, indicative of augmented tumor cell death and HMGB1 release, consistent with oncolytic virus-mediated cytolysis (Supplementary Fig. 31b).”

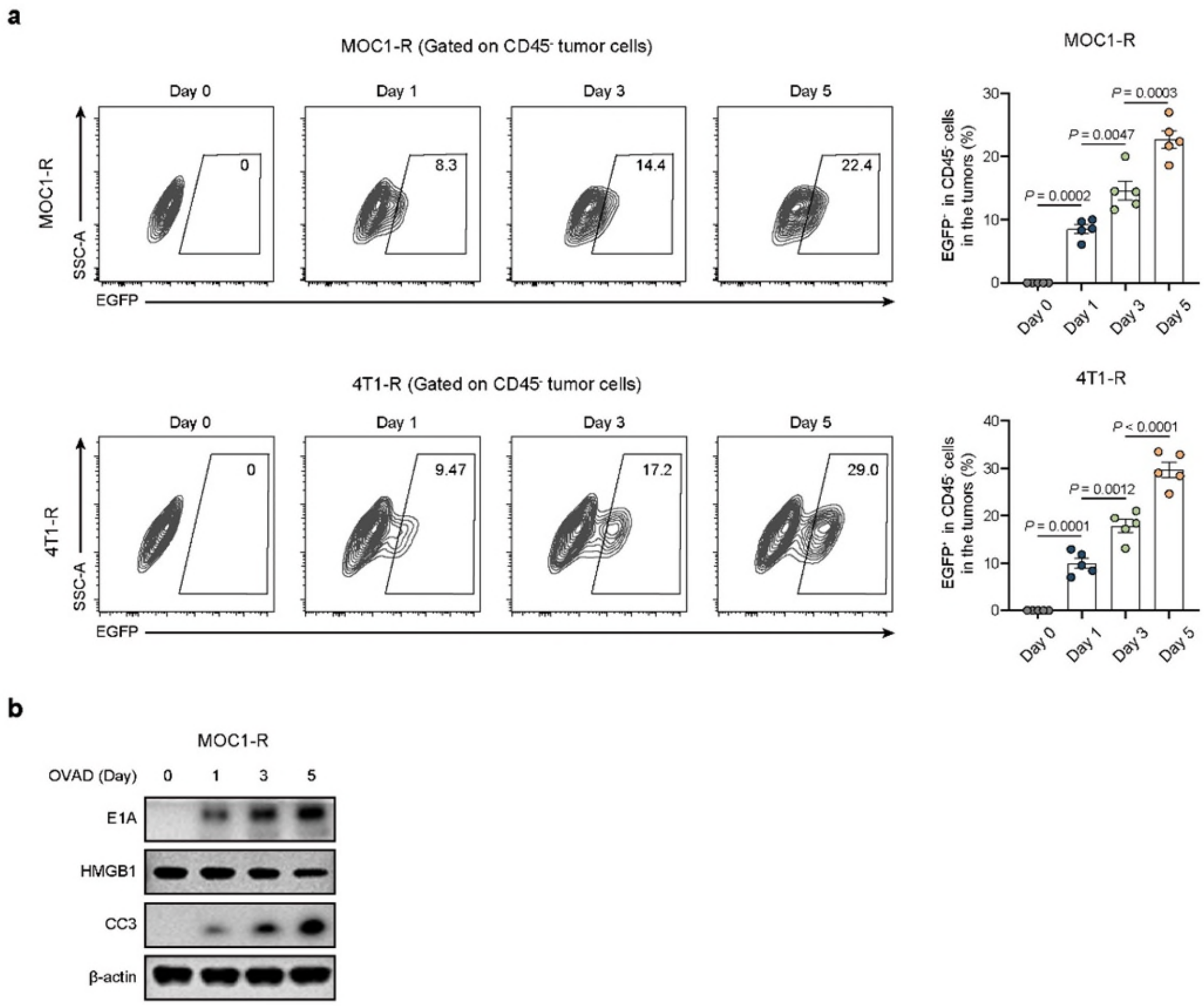

Supplementary Fig. 31| Intratumoral replication and cytotoxicity of oncolytic adenovirus in radioresistant tumor xenografts. a Flow cytometric analysis of EGFP-positive cell frequency in MOC1-R (upper) and 4T1-R (lower) tumor tissues at the indicated days after intratumoral OVAD injection (0.4×10^6 PFU for MOC1-R; 2×10^6 PFU for 4T1-R). **b** Immunoblot analysis of viral E1A protein, HMGB1, and cleaved caspase-3 (CC3) in MOC1-R tumor lysates across sequential time points post OVAD administration. β-actin served as the loading control. Statistical significance was assessed using one-way ANOVA with Tukey’s multiple-comparisons test (**a**). Data are presented as mean ± s.e.m. from at least three independent experiments.

Collectively, these data support that intratumorally administered OVAD efficiently infects tumor cells, undergoes productive replication, and elicits oncolytic cell death within the tumor microenvironment. We appreciate the reviewer’s suggestion, which

has helped us substantiate this point more rigorously.

Comment 8:

8. A critical function of oncolytic viruses in anti-tumor therapy is the abscopal effect. Whether the combination therapy employing framework materials and OVs can simultaneously affect distal tumor foci requires investigation and validation—for example, via bilateral tumor-bearing models to monitor tumor growth and tumor microenvironment changes.

Response 8:

We sincerely thank the reviewer for this insightful comment. We respectfully agree that the abscopal effect represents a clinically relevant endpoint for oncolytic virus-based cancer immunotherapy, and we have performed dedicated experiments to evaluate whether our combination regimen elicits systemic antitumor immunity against untreated distal lesions.

To address this question, we established a bilateral subcutaneous 4T1-R tumor model, in which local treatment was administered exclusively to the ipsilateral primary tumor, while the contralateral distal tumor received no direct intervention (Fig. 8c). Our key findings are summarized below:

(1) The triple combination of IR, COF-Tpy-Se-Cu and OVAD significantly suppressed the growth of both locally treated primary tumors and untreated contralateral distal tumors, and this effect was more pronounced than that of OVAD monotherapy or the IR + COF-Tpy-Se-Cu dual regimen (Fig. 8d, e and Supplementary Fig. 37a, b).

(2) Immunophenotypic analyses further revealed that combination treatment enhanced dendritic cell maturation and increased the frequency of memory $CD8^+$ T cells in the contralateral tumor-draining lymph nodes, and was associated with elevated $CD8^+$ T cell infiltration within distal tumor tissue (Supplementary Fig. 37c-j).

Collectively, these findings indicate that local administration of the triple combination regimen induces systemic adaptive antitumor immunity that extends to

untreated distal tumor sites. All corresponding results have been incorporated into the revised manuscript. Tumor growth curves are presented in Fig. 8c–e, and detailed immunological characterization is provided in Supplementary Fig. 37c-j.

The relevant section of the main text has been updated as follows:

"We subsequently evaluated the abscopal antitumor activity of the regimen using a bilateral 4T1-R tumor model, with treatment restricted exclusively to the primary lesion (Fig. 8c). The triple combination of IR, COF-Tpy-Se-Cu, and OVAD significantly suppressed the growth of both the treated primary tumors and the untreated contralateral distant tumors, outperforming both OVAD monotherapy and the IR + COF-Tpy-Se-Cu dual regimen (Fig. 8d, e and Supplementary Fig. 37a, b). Immunophenotyping of contralateral tumor-draining lymph nodes showed enhanced DC maturation and expanded memory $CD8^+$ T cell populations, paired with increased $CD8^+$ T cell infiltration in distal tumors (Supplementary Fig. 37c-j). Collectively, these data demonstrate that the combination therapy elicits robust systemic antitumor immunity capable of controlling distant tumor foci."

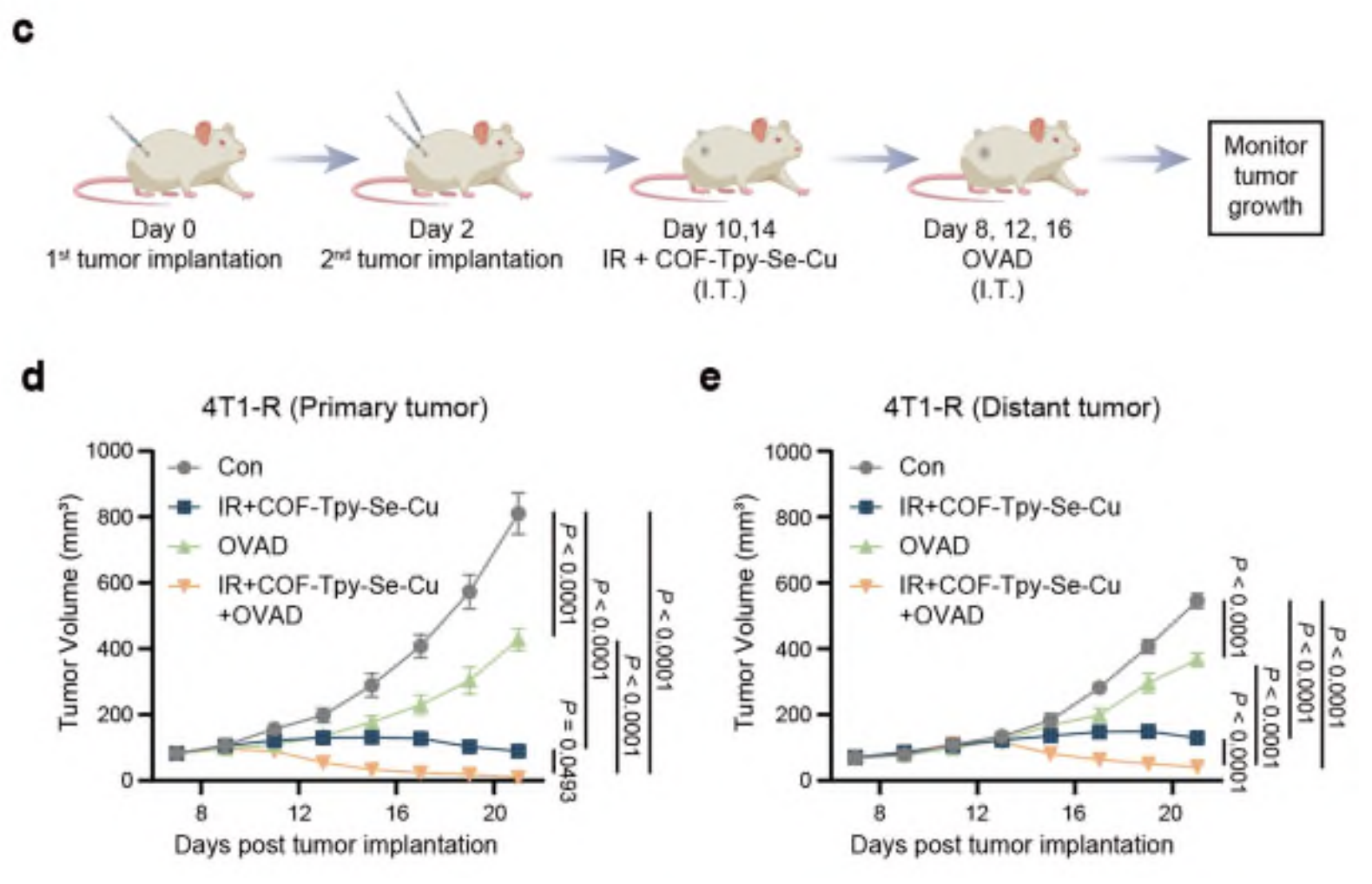

Fig. 8| c–e Evaluation of abscopal activity using a bilateral tumor model in which a secondary contralateral tumor was established. (c) Treatment schedule for the combination regimen. Growth curves of the treated primary tumor (d) and the untreated distant tumor (e).

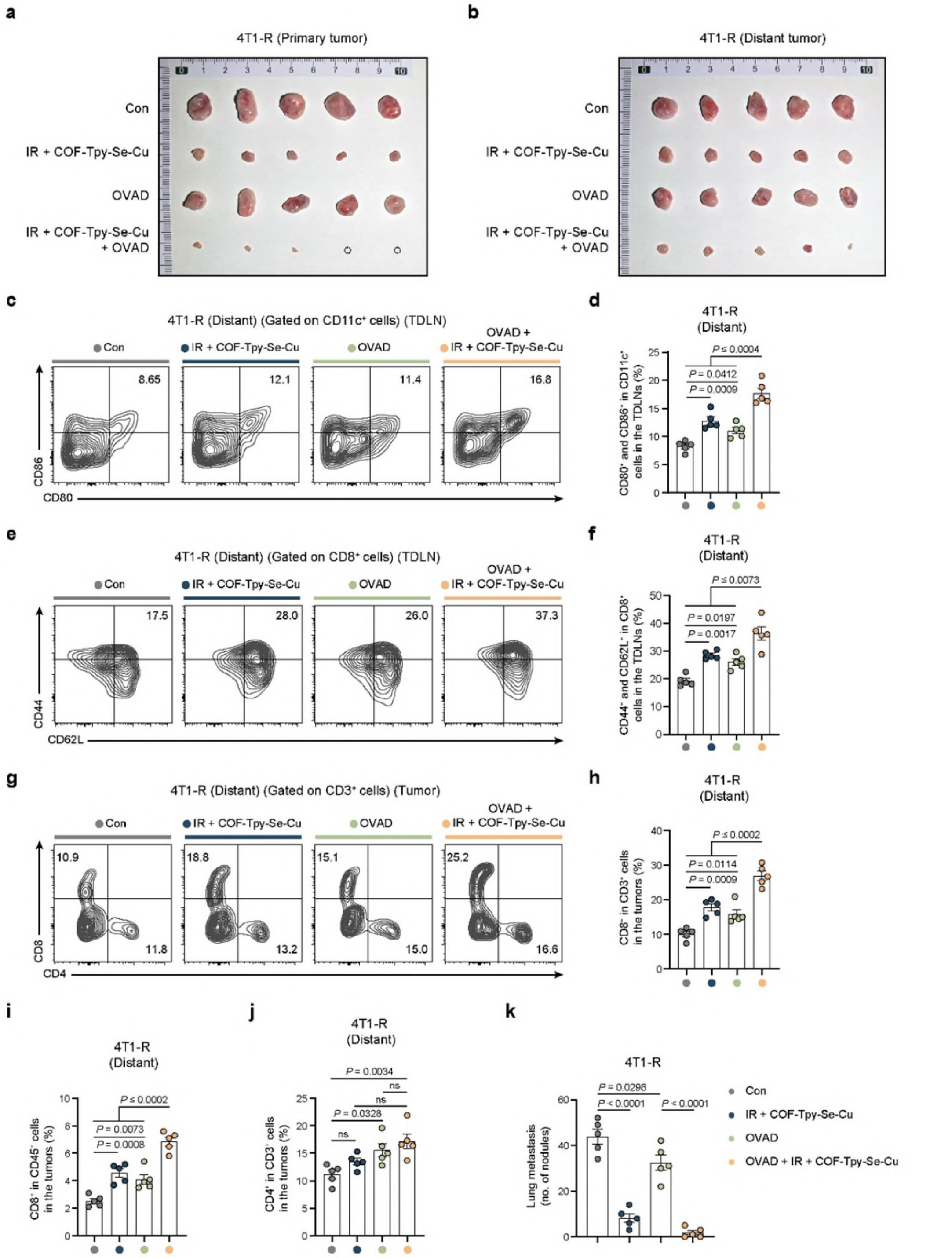

Supplementary Fig. 37| Combination therapy elicits a robust abscopal effect and systemic antitumor immunity. a Representative images of excised primary (ipsilateral, treated) tumors from 4T1-R–bearing mice under the indicated treatments. **b** Representative images of the corresponding distant (contralateral, untreated) tumors, demonstrating tumor rejection following combination therapy. **c, d** Flow cytometric analysis of $CD80^+CD86^+$ cells among $CD11c^+$ DCs in distant-tumor-draining lymph nodes (TDLNs). **e, f** Flow cytometric analysis of $CD44^+CD62L^+$ memory T cells among $CD8^+$ T cells in distant-tumor-draining lymph

nodes. **g–j** Flow cytometric profiling of tumor-infiltrating T cells in distant tumors. **k** Quantification of pulmonary metastatic nodules across treatment groups. Statistical significance was assessed using one-way ANOVA with Tukey's multiple-comparisons test (**d, f, h, i, j, k**). Data are presented as mean ± s.e.m. from at least three independent experiments.

Comment 9:

9. What is the specific information about your oncolytic virus? It should be assessed whether radiotherapy resistant tumor cells affect the expression of receptors for the oncolytic virus, as increased receptor expression can enhance viral infectivity and therapeutic efficacy. This requires validation at both cellular and animal levels. The cellular-level impact of radioresistance on tumor cell sensitivity to the oncolytic virus should be examined. In vivo experiments, a radiotherapy alone followed by oncolytic virus treatment group should be included to observe tumor growth differences compared with other groups.

Response 9:

We sincerely thank the reviewer for this comprehensive and constructive comment, which has prompted us to clarify the detailed characteristics of the oncolytic virus, carefully assess the potential confounding effect of radioresistance on viral infectivity, and evaluate the efficacy of sequential radiotherapy followed by oncolytic virus as a dedicated control group. We appreciate the opportunity to address these important points, and our point-by-point responses are provided below.

(1) Detailed information on the oncolytic adenovirus

We have supplemented the Methods section with full details of the oncolytic virus used in this study, and a schematic vector map is provided in Supplementary Fig. 26. The corresponding manuscript text reads as follows:

"**Oncolytic adenovirus**

The oncolytic adenovirus OVAD-hTERT-E1A-IRES-E1B-SV40-EGFP-RGD (catalog no. OVAD184-2282; FuBio Biotechnology, Suzhou, China) was used in this study [1,2], with its vector map provided in Supplementary Fig. 26. This serotype 5 adenovirus features E1A/E1B expression driven by the human telomerase reverse

transcriptase (hTERT) promoter for tumor-selective replication, a complete E3 region deletion, and an RGD-modified fiber knob mediating viral entry. An EGFP reporter was incorporated for infection tracking.

Virus was amplified in HEK293 cells. Cells were incubated with viral inoculum and cultured until widespread cytopathic effect appeared. Harvested cells were subjected to three freeze-thaw cycles, and the resulting lysate was concentrated by PEG 8000 precipitation and purified via double CsCl density gradient ultracentrifugation. Viral stocks were titrated by plaque-forming assay and stored at −80 °C until use. For replication kinetics, cells were seeded 24 h before infection at an MOI of 1. After 2 h of incubation, the inoculum was replaced with fresh medium, and viral titers were measured at serial time points using the $TCID_{50}$ assay."

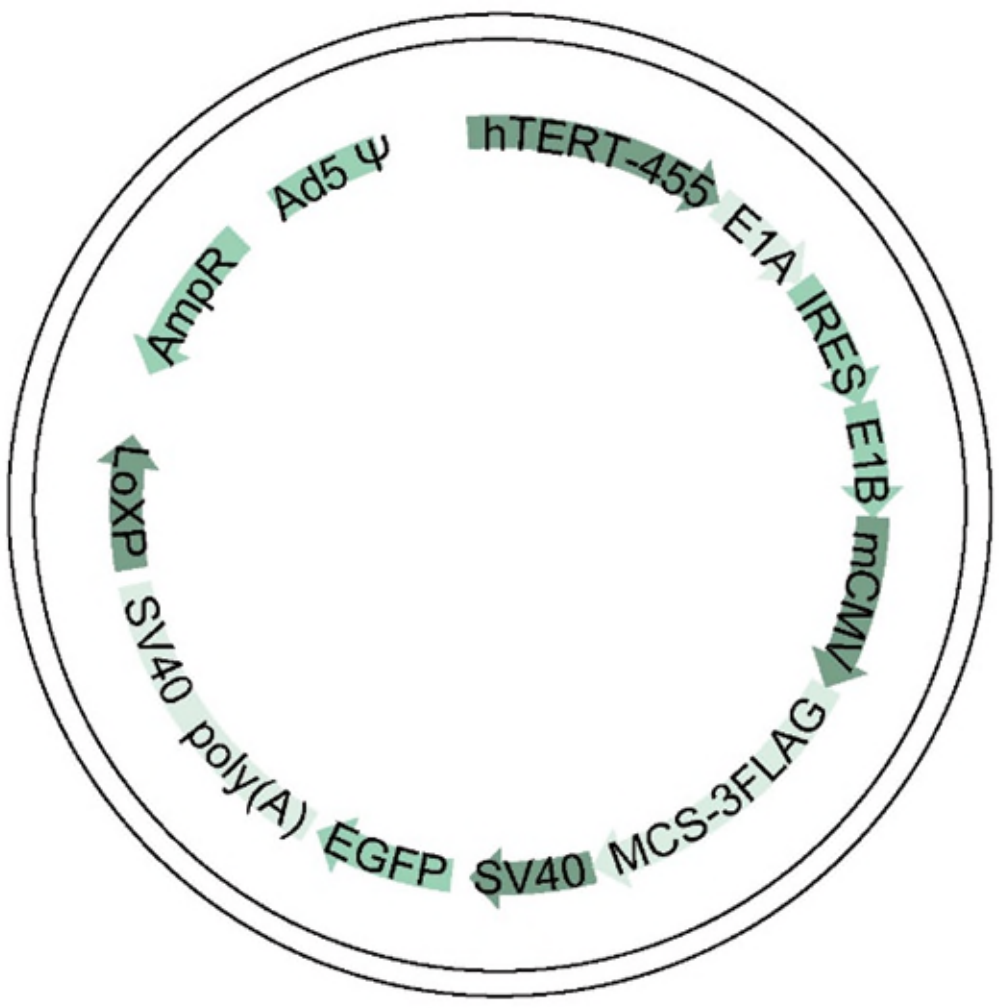

Supplementary Fig. 26| Schematic of the engineered oncolytic adenovirus vector OVAD-hTERT-E1A-IRES-E1B-SV40-EGFP-RGD (OVAD184-2282).

(2) Impact of acquired radioresistance on OVAD infectivity, replication, and cytolytic activity

To address whether radioresistance alters tumor cell sensitivity to OVAD, we systematically compared parental and radioresistant cell lines at both the molecular and functional levels, with further validation in tumor tissues.

At the molecular level, flow cytometry and immunoblotting showed no significant difference in the protein levels of CAR (the primary adenovirus entry receptor) or TERT (the driver of viral selective replication) between parental and radioresistant cells (Supplementary Fig. 27a, b). Immunohistochemical staining of tumor tissues further confirmed comparable expression of both markers in vivo (Supplementary Fig. 27c).

Functionally, $TCID_{50}$ assays performed at an MOI of 1 demonstrated nearly identical viral replication dynamics in MOC1 and MOC1-R cells across the time course (Supplementary Fig. 27d). In addition, OVAD induced equivalent levels of cell death in both parental and radioresistant cell lines across a range of MOIs (Supplementary Fig. 28a, b).

Collectively, these data support that acquired radioresistance does not enhance OVAD infectivity, replicative capacity, or cytolytic efficacy. Therefore, the synergistic therapeutic effect observed in our combination regimen is unlikely to be attributable to increased viral sensitivity of radioresistant tumor cells. All related data are presented in Supplementary Figs. 27 and 28.

The corresponding manuscript text has been updated as follows:

"Before investigating the synergistic interaction, we first rigorously evaluated whether acquired radioresistance alters OVAD infectivity, replication, and cytolytic activity to exclude potential confounding effects of differential viral sensitivity. Expression of the coxsackievirus and adenovirus receptor (CAR), which mediates viral entry, and telomerase reverse transcriptase (TERT), which drives tumor-selective viral replication, did not differ significantly between parental and radioresistant cells at both the cellular protein and tissue levels (Supplementary Fig. 27a–c). $TCID_{50}$ assays performed at a multiplicity of infection (MOI) of 1 confirmed comparable OVAD replication kinetics in MOC1 and MOC1-R cells (Supplementary Fig. 27d). Consistent with these findings, OVAD elicited equivalent levels of cell death in both cell lines across a range of MOIs (Supplementary Fig. 28a, b). Collectively, these data indicate that acquired radioresistance does not impair OVAD infectivity, replicative capacity, or cytolytic activity."

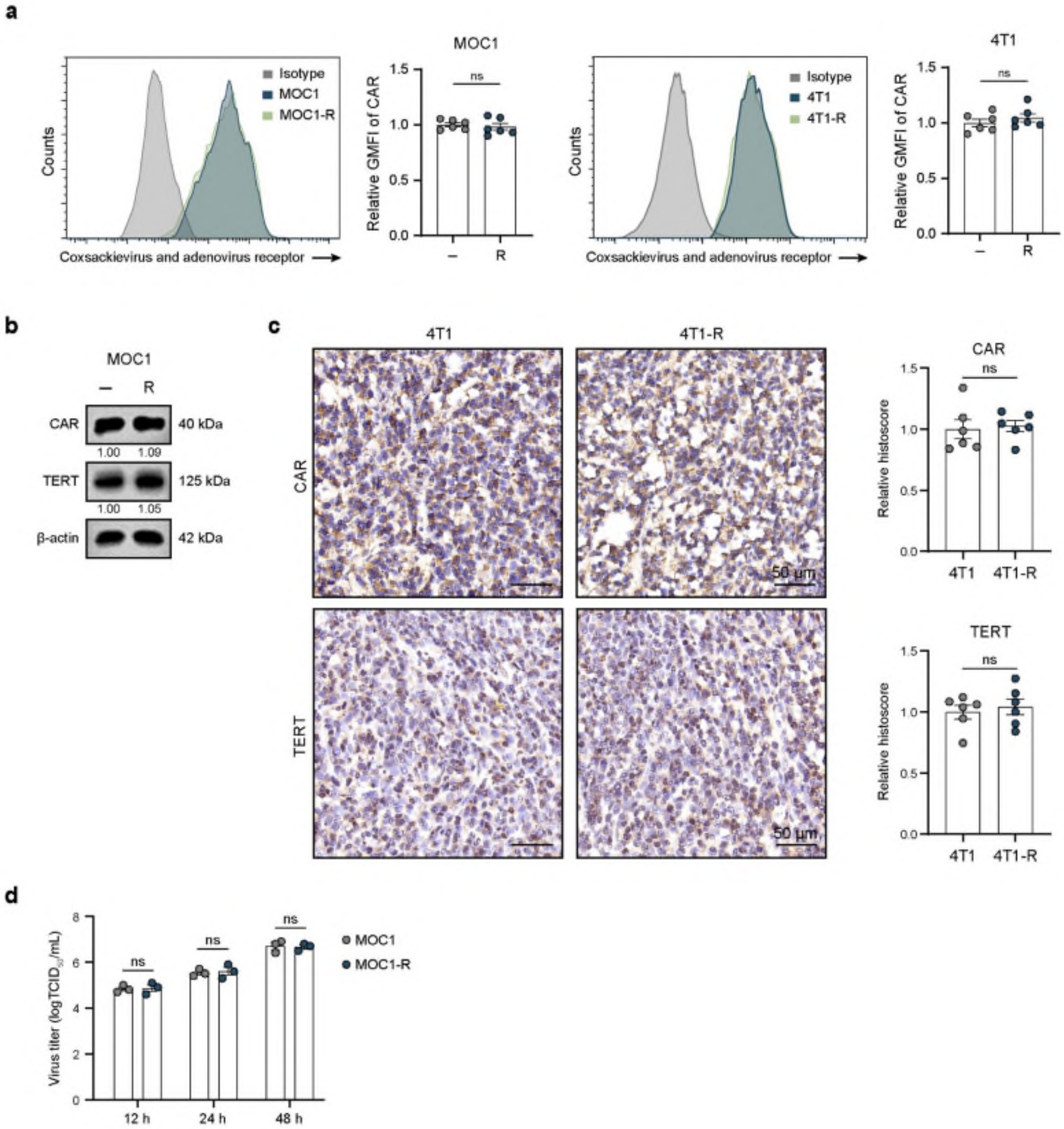

Supplementary Fig. 27| Radioresistance does not alter oncolytic adenovirus receptor expression or viral replication kinetics. a Flow cytometric analysis of coxsackievirus and adenovirus receptor (CAR) expression in parental and radioresistant cells. Isotype antibody was used as the negative control. **b** Immunoblot analysis of CAR and telomerase reverse transcriptase (TERT) protein levels in MOC1 and MOC1-R cells. β-actin served as the loading control, with relative gray values labeled below each band. **c** Immunohistochemical staining of CAR and TERT in 4T1 and 4T1-R tumor tissues, with quantitative analysis of relative histoscore on the right (scale bar, 50 μm). **d** Replication kinetics of OVAD in MOC1 and MOC1-R cells infected at an MOI of 1. Viral titers were determined by $TCID_{50}$ assay at the indicated time points post-infection. Statistical significance was assessed using two-tailed Student's t test (**a, c, d**). Data are presented as mean ± s.e.m. from at least three independent experiments.

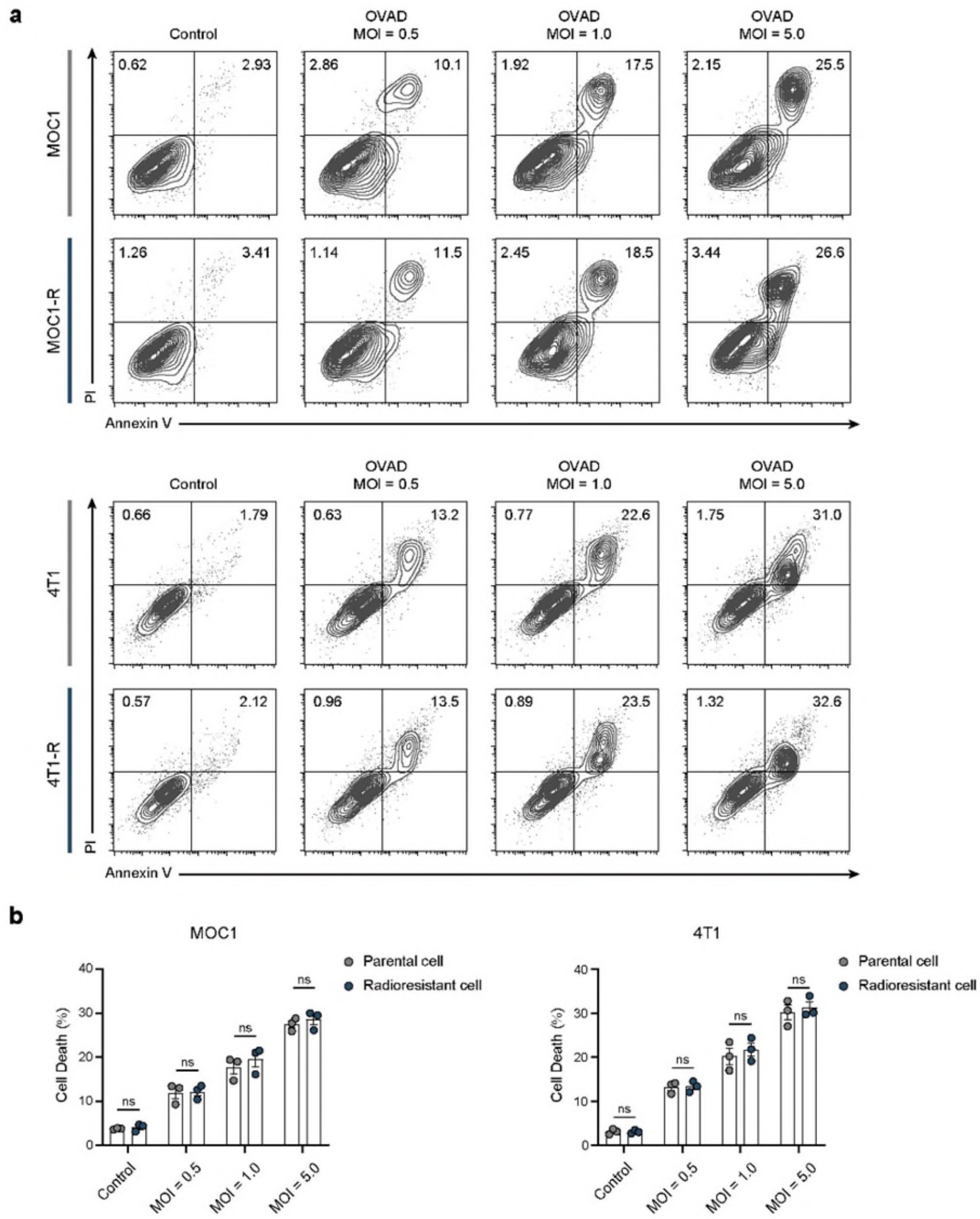

Supplementary Fig. 28| Oncolytic adenovirus exerts equivalent cytotoxicity in parental and radioresistant tumor cells. a Flow cytometric analysis of Annexin V/PI staining in MOC1 and 4T1 parental and radioresistant cells at 24 h after OVAD infection at the indicated multiplicities of infection (MOIs). **b** Quantification of cell death, expressed as the percentage of PI-positive cells. Statistical significance was assessed using two-tailed Student's *t* test (**b**). Data are presented as mean ± s.e.m. from at least three independent experiments.

(3) In vitro and in vivo evaluation of the IR + OVAD control group

As recommended, we have included a sequential treatment group of irradiation followed by OVAD administration in both cellular and animal experiments to enable direct comparison with the other treatment arms.

In vitro findings (Supplementary Fig. 30a, b): In both MOC1-R and 4T1-R

radioresistant cell lines, sequential irradiation followed by OVAD treatment resulted in a non-significant increase in cell death compared with OVAD monotherapy. This result is consistent with the inherent radioresistant phenotype of these cells, in which ionizing radiation alone exerts limited cytotoxicity and cannot substantially amplify oncolytic virus-mediated cell killing. In contrast, the triple combination of IR, COF-Tpy-Se-Cu, and OVAD induced markedly enhanced cell death (Supplementary Fig. 30a, b), consistent with the synergistic amplification of cuproptosis (Supplementary Fig. 30e).

In vivo findings (Supplementary Fig. 32): Concordant results were observed in both the MOC1-R orthotopic and 4T1-R subcutaneous tumor models. The sequential IR-followed-by-OVAD group showed no significant difference in tumor growth inhibition compared with OVAD monotherapy, and its efficacy was substantially lower than that of the IR + COF-Tpy-Se-Cu regimen. The triple combination achieved the strongest tumor growth suppression among all treatment groups.

These results support our mechanistic model: radiotherapy alone is insufficient to overcome radioresistance, whereas radiation-triggered copper release from COF-Tpy-Se-Cu, in concert with OVAD-mediated FDX1 stabilization and GSH depletion, drives robust cuproptosis and potent radiosensitization. The corresponding data are shown in Supplementary Fig. 30 (in vitro) and Supplementary Fig. 32 (in vivo).

The corresponding manuscript text has been updated as follows:

"Relative to irradiation alone, COF-Tpy-Se-Cu significantly increased the proportion of propidium iodide (PI)-positive cells (Fig. 7a, b). Notably, sequential irradiation followed by OVAD administration resulted in a non-significant increase in cell death relative to OVAD monotherapy (Supplementary Fig. 30a, b). This observation is consistent with the intrinsic radioresistant phenotype of these cells, whereby ionizing radiation alone exerts limited cytotoxicity and cannot substantially amplify oncolytic virus-mediated cell killing. In contrast, the triple combination of irradiation, COF-Tpy-Se-Cu, and OVAD induced markedly enhanced cell death, consistent with the synergistic amplification of cuproptosis (Fig. 7a, b and Supplementary Fig. 30a, b)."

"We next evaluated the in vivo antitumor performance of the combined therapeutic regimen. Relative to the control group, OVAD monotherapy and COF-Tpy-Se-Cu plus irradiation both achieved significant tumor growth suppression (Fig. 7e–g and Supplementary Fig. 32). In line with the radioresistant phenotype of these models, the addition of single-modality irradiation to OVAD failed to further improve tumor growth control, resulting in comparable efficacy to OVAD alone (Supplementary Fig. 32). In contrast, the triple combination of COF-Tpy-Se-Cu, OVAD, and irradiation elicited the strongest antitumor effect, with final tumor volumes significantly lower than those of all monotherapy and dual-combination groups (Fig. 7e–g and Supplementary Fig. 32, 33a, b)."

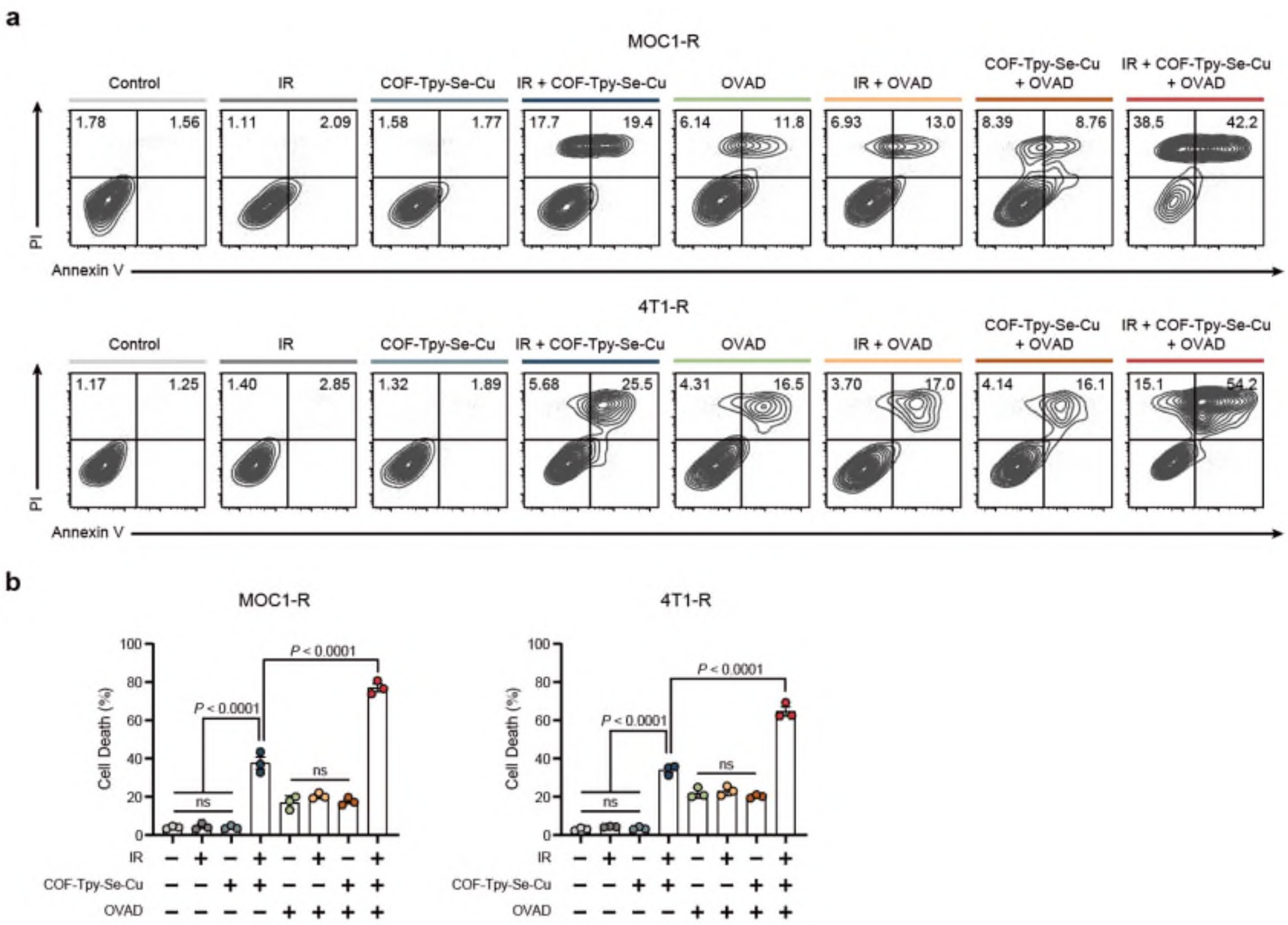

Supplementary Fig. 30| Synergistic radiosensitization efficacy of COF-Tpy-Se-Cu combined with an oncolytic adenovirus in vitro. a Flow cytometric analysis of Annexin V/PI staining in radioresistant MOC1-R (upper) and 4T1-R (lower) cells at 24 h after the indicated treatments (COF-Tpy-Se-Cu, 30 μg mL^{-1}; IR, 4 Gy; OVAD, MOI = 1). **b** Quantification of cell death, expressed as the percentage of PI-positive cells, in MOC1-R (left) and 4T1-R (right) cells.

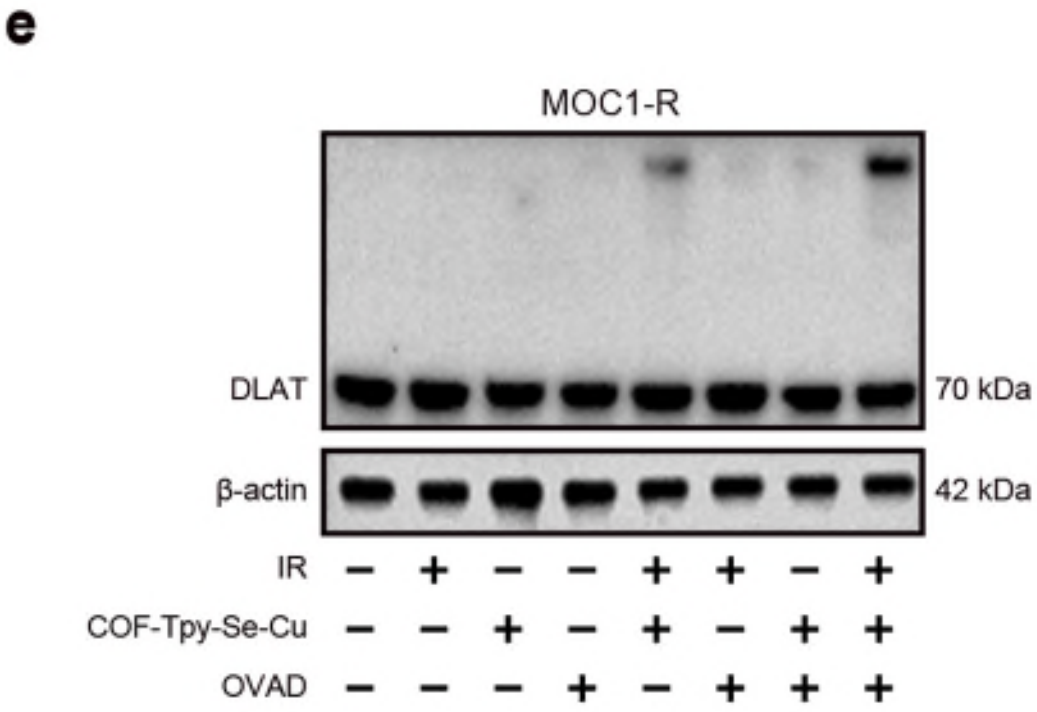

Supplementary Fig. 30| e Immunoblot analysis of DLAT oligomerization in MOC1-R cells under the indicated treatments (COF-Tpy-Se-Cu, 30 μg mL^{-1}; IR, 4 Gy; OVAD, MOI = 1). β-actin served as the loading control.

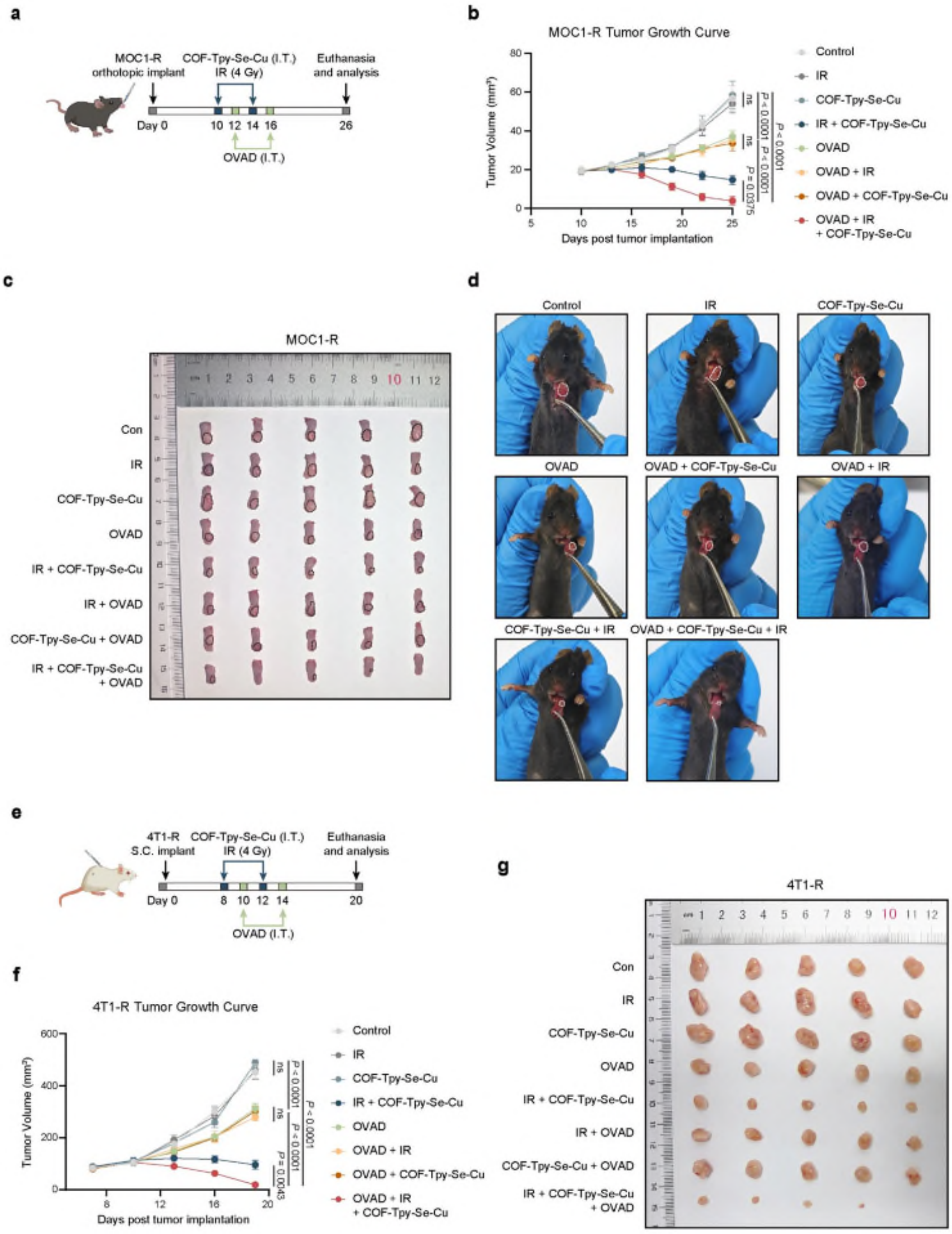

Supplementary Fig. 32| Synergistic radiosensitization efficacy of COF-Tpy-Se-Cu combined with oncolytic adenovirus in vivo. a In vivo treatment schedule. MOC1-R tumor-bearing mice received intratumoral injections of COF-Tpy-Se-Cu (2 mg kg^{-1}) and/or OVAD (0.4×10^6 PFU), combined with localized irradiation (IR, 4 Gy) as indicated. **b** Tumor growth curves for MOC1-R xenografts under the indicated treatments (n = 5 mice per group). **c** Representative images of excised tongues from mice bearing orthotopic MOC1-R tumors at the endpoint (n = 5 mice per group). Tumor boundaries are delineated by black dashed lines. **d** Representative images of mice bearing orthotopic MOC1-R tongue tumor across eight treatment groups. Tumor boundaries are delineated with white dashed lines. **e** In vivo treatment schedule. 4T1-R tumor-bearing mice received intratumoral injections of COF-Tpy-Se-Cu (5 mg kg^{-1}) and/or OVAD (2×10^6 PFU), combined with localized irradiation (IR, 4 Gy) as indicated. **f** Tumor growth curves for 4T1-R xenografts under the indicated treatments (n = 5 mice per group). **g** Representative images of excised subcutaneous 4T1-R tumors from each treatment group at the endpoint (n = 5 mice per group). Statistical significance was assessed using two-way ANOVA (**b, f**). Data are presented as mean ± s.e.m. from at least three independent experiments.

Comment 10:

10. 4h: Add a quantitative statistical difference map.

Response 10:

We sincerely thank the reviewer for this suggestion, which has strengthened the quantitative presentation of our data. In the revised manuscript, we have retained representative flow cytometry histograms of HSP70 expression in Fig. 4h, and added a dedicated quantitative bar graph with formal statistical analysis in Supplementary Fig. 11d.

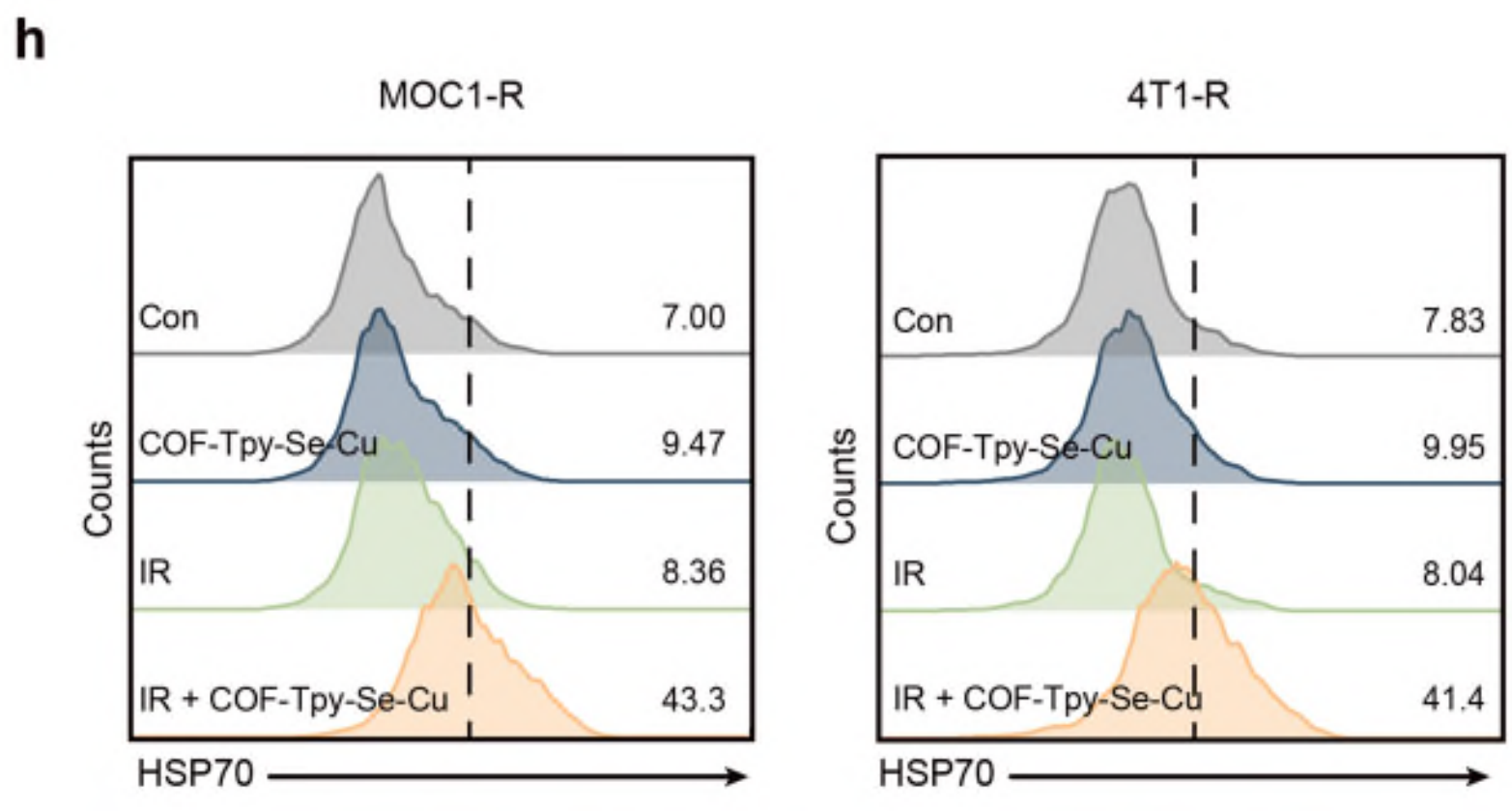

Fig. 4| h Flow cytometric quantification of the relative HSP70-positive cell subset ratio in MOC1-R and 4T1-R cells following the indicated treatments.

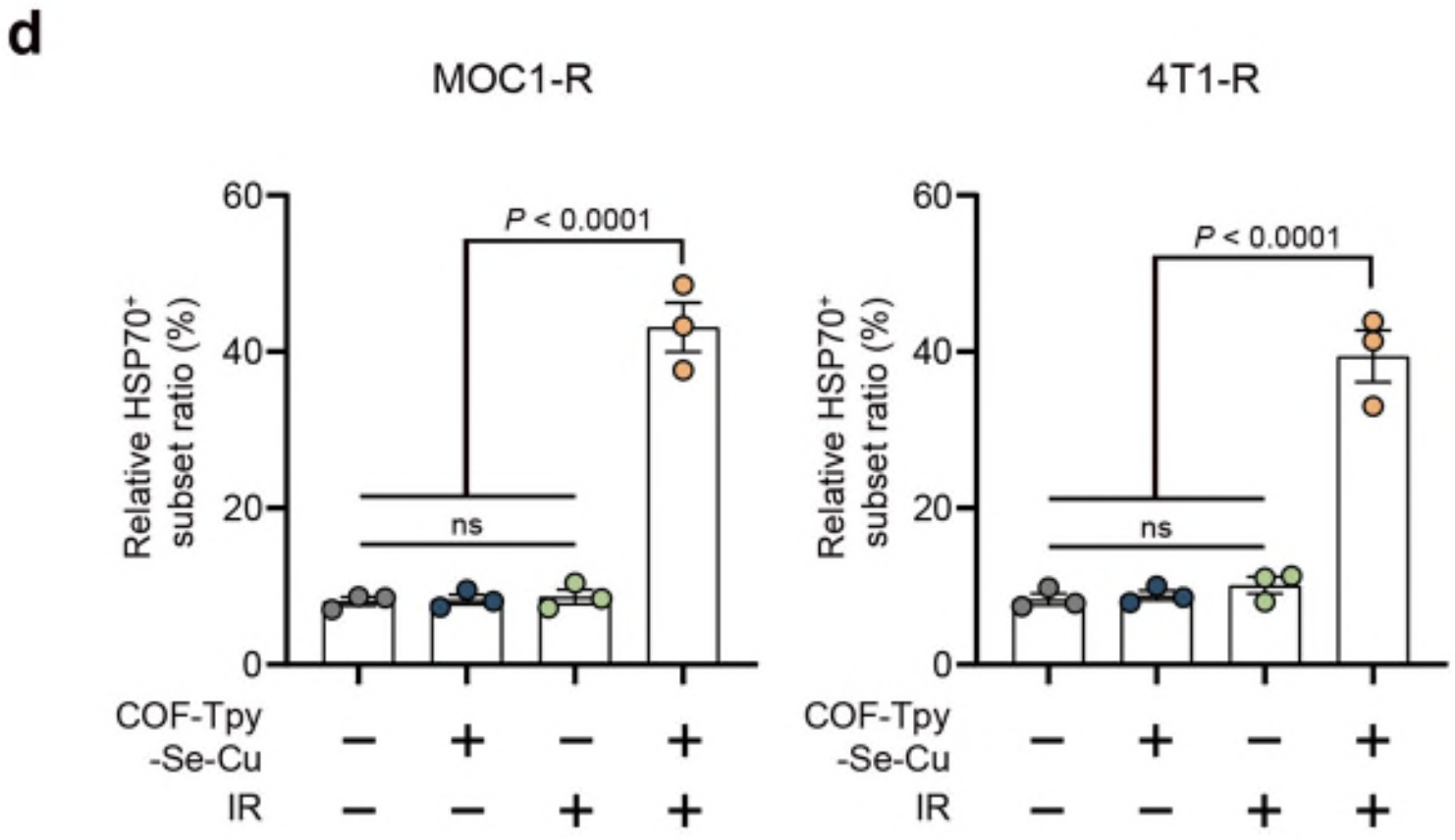

Supplementary Fig. 11| d Flow cytometric quantification of the relative HSP70-positive cell subset ratio in MOC1-R and 4T1-R cells following the indicated treatments. Statistical significance was assessed using one-way ANOVA with Tukey's multiple-comparisons test (**d**). Data are presented as mean ± s.e.m. from at least three independent experiments.

Comment 11:

11. 5f: Label the CD4 proportion on the figure and include a statistical plot showing the proportion of CD8 among $CD45^+$ cells.

Response 11:

We sincerely thank the reviewer for this valuable suggestion to improve the clarity and comprehensiveness of our tumor-infiltrating T cell analysis. We have labeled the percentage values of the $CD4^+$ T cell population directly on the flow cytometry plots in Fig. 5f and Supplementary Fig. 20b. We have added full quantitative statistical analyses covering three key T cell metrics: the frequency of $CD8^+$ T cells among $CD3^+$ T cells, the frequency of $CD8^+$ T cells among $CD45^+$ leukocytes, and the frequency of $CD4^+$ T cells among $CD3^+$ T cells, for both the MOC1-R and 4T1-R tumor models. We retained the core metric ($CD8^+$ frequency among $CD3^+$ T cells) in the main figure to highlight the T cell subset distribution, and placed the $CD45^+$-normalized quantification in the supplementary material (Supplementary Fig. 20c) as a complementary dimension of immune infiltration.

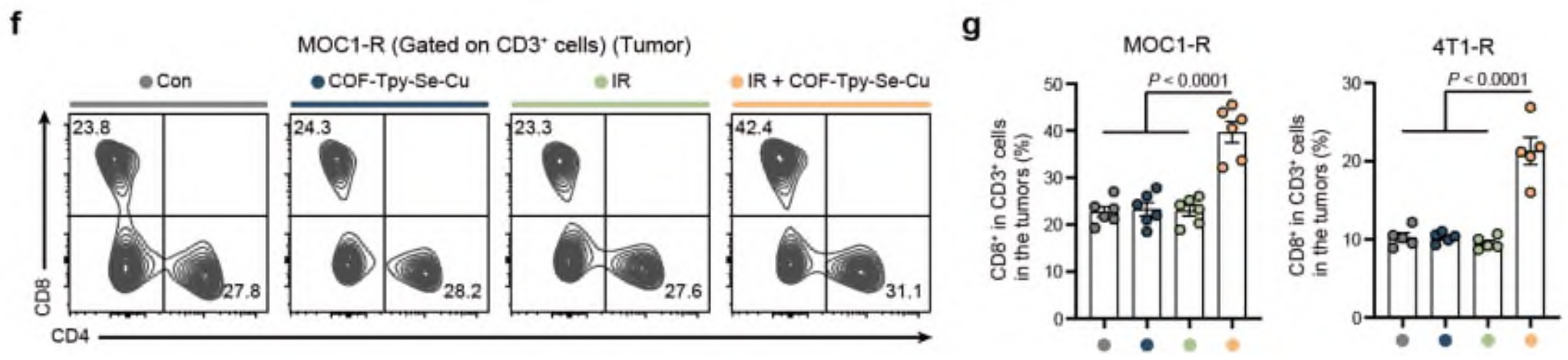

Fig. 5| f, g Flow cytometric analysis of tumor-infiltrating CD8$^+$ T and CD4$^+$ T cells among CD3$^+$ T cells. (f) Representative plots from MOC1-R tumors. (g) Quantification of CD8$^+$ T cell frequency among CD3$^+$ T cells in MOC1-R (left) and 4T1-R (right) tumors.

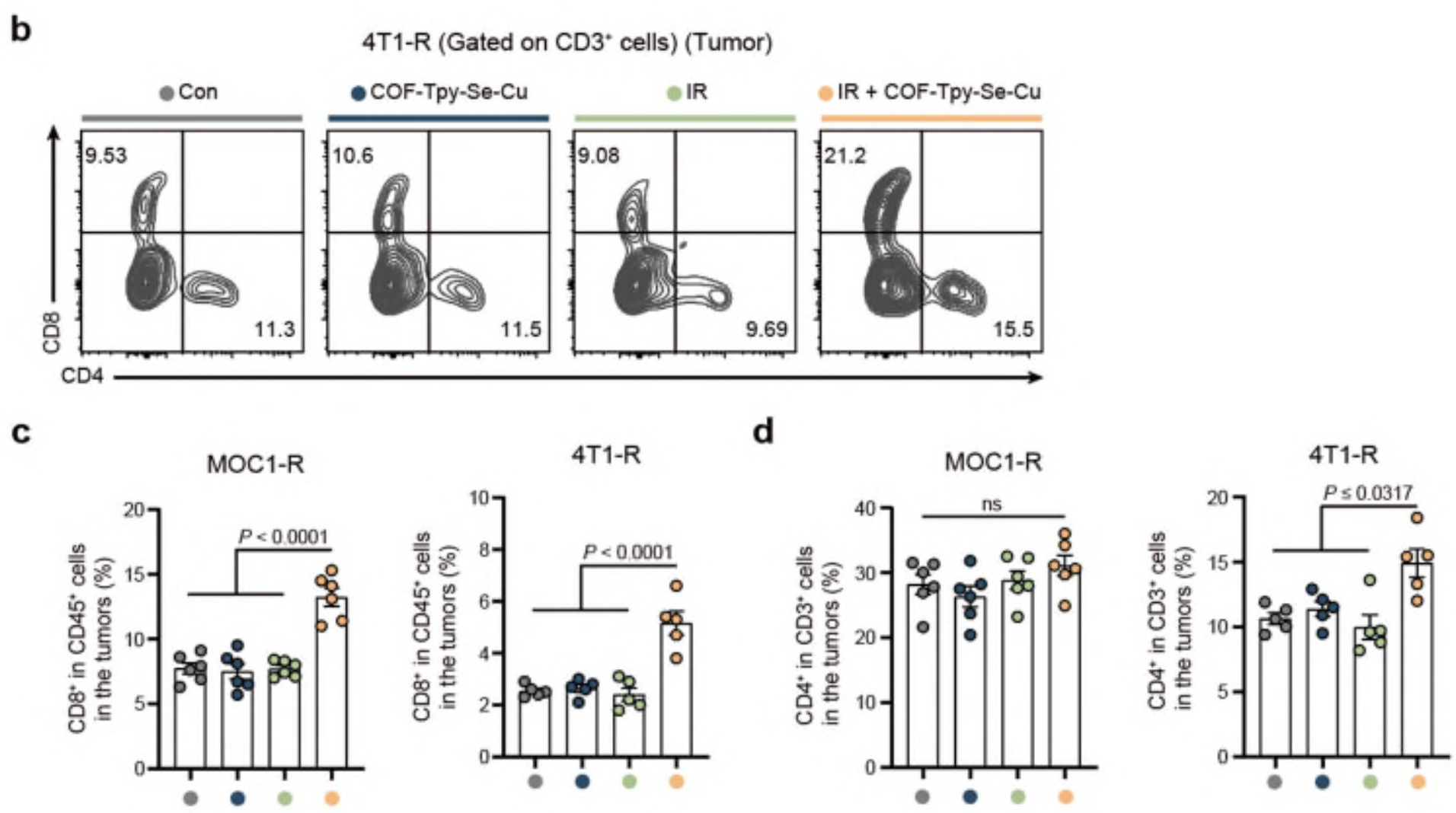

Supplementary Fig. 20| b Flow cytometric analysis of tumor-infiltrating CD8$^+$ T and CD4$^+$ T cells among CD3$^+$ T cells from 4T1-R models. **c** Quantification of CD8$^+$ T cell frequency among CD45$^+$ leukocytes in MOC1-R (left) and 4T1-R (right) tumors. **d** Quantification of CD4$^+$ T cell frequency among CD3$^+$ T cells in MOC1-R (left) and 4T1-R (right) tumors. Statistical significance was assessed using one-way ANOVA with Tukey's multiple-comparisons test (**c, d**). Data are presented as mean ± s.e.m. from at least three independent experiments.

Comment 12:

12. 5j: Quantification of immunohistochemistry data is lacking. Additionally, the IHC images show that the morphology of CD8 and GZMB staining is not quite accurate. It would be better to use multiplex immunofluorescence to co-stain CD8, GZMB, KI67, and lipoic acid, as this would more intuitively demonstrate co-expression characteristics.

Response 12:

We sincerely thank the reviewer for this valuable suggestion, which has improved

the rigor and interpretability of our in situ tissue analysis. We apologize for the limitations of the previous single-plex immunohistochemistry presentation and have revised this part accordingly.

Following the reviewer's recommendation, we performed multiplex immunofluorescence staining to simultaneously visualize CD8, granzyme B, and lipoic acid on the same tumor sections. This approach enables direct in situ assessment of cytotoxic T cell infiltration alongside a canonical biochemical hallmark of cuproptosis, and addresses the prior limitations of single-plex immunohistochemistry regarding staining morphology and quantitative analysis. Representative images are now presented in Fig. 5j, replacing the previous single-plex immunohistochemistry, and quantitative analysis of relative fluorescence intensity for all three markers across treatment groups is provided in Supplementary Fig. 21a.

Given that Ki67 localizes to cell nuclei, its subcellular distribution differs markedly from membrane-bound CD8 and mitochondrially concentrated lipoic acid. Including Ki67 within the same multiplex panel would introduce additional spectral overlap and complicate signal interpretation. Since our core mechanistic focus lies on the interplay between cytotoxic antitumor immunity and cuproptosis, we opted to assess Ki67 separately via conventional immunohistochemistry paired with semi-quantitative histoscoring. This strategy yields robust quantification of tumor proliferative activity while preserving the clarity of our multiplex panel focused on immune effectors and cuproptosis markers. Representative Ki67 staining images and corresponding quantitative histoscoring data from both tumor models are provided in Supplementary Fig. 21b.

The corresponding text in the manuscript has been updated as follows:

"Multiplex immunofluorescence analysis confirmed in situ that combined IR and COF-Tpy-Se-Cu treatment increased $CD8^+$ T cell infiltration and granzyme B expression, and reduced lipoic acid levels, consistent with enhanced cytotoxic immunity and cuproptosis induction (Fig. 5j and Supplementary Fig. 21a). Immunohistochemical staining for Ki67, quantified via histoscoring, verified

significantly suppressed tumor proliferation in both models (Supplementary Fig. 21b).”

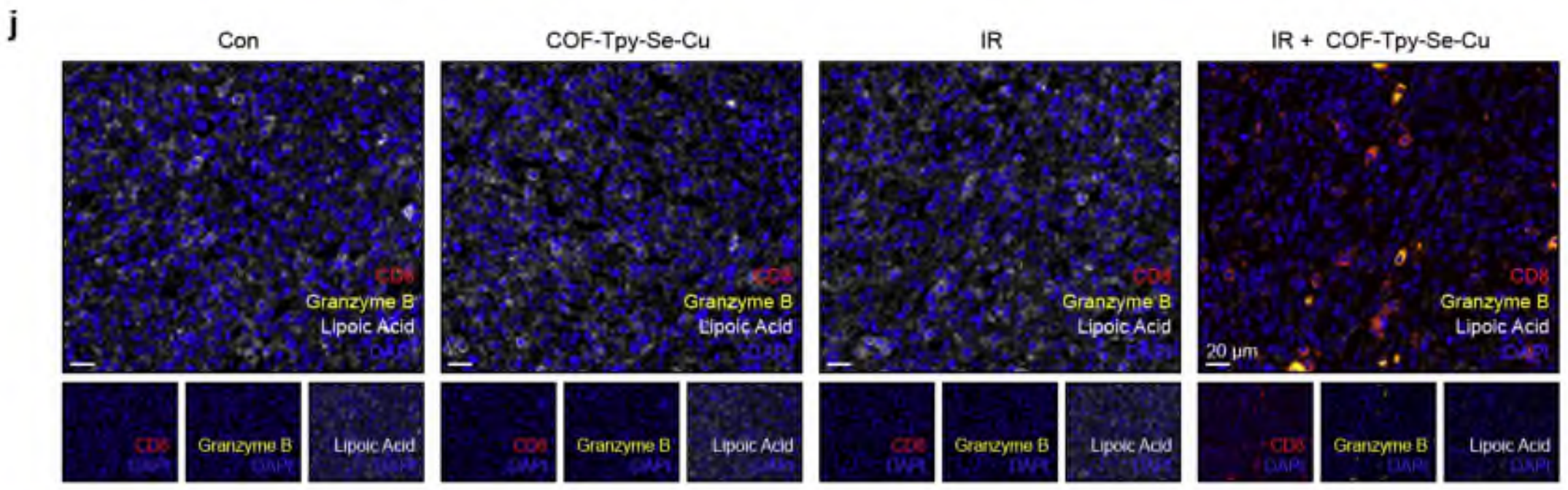

Fig. 5| j Multiplex immunohistochemistry (mIHC) staining showing the expression of CD8 (red), granzyme B (yellow), and lipoic acid (white) in 4T1-R tumor sections from each treatment group.

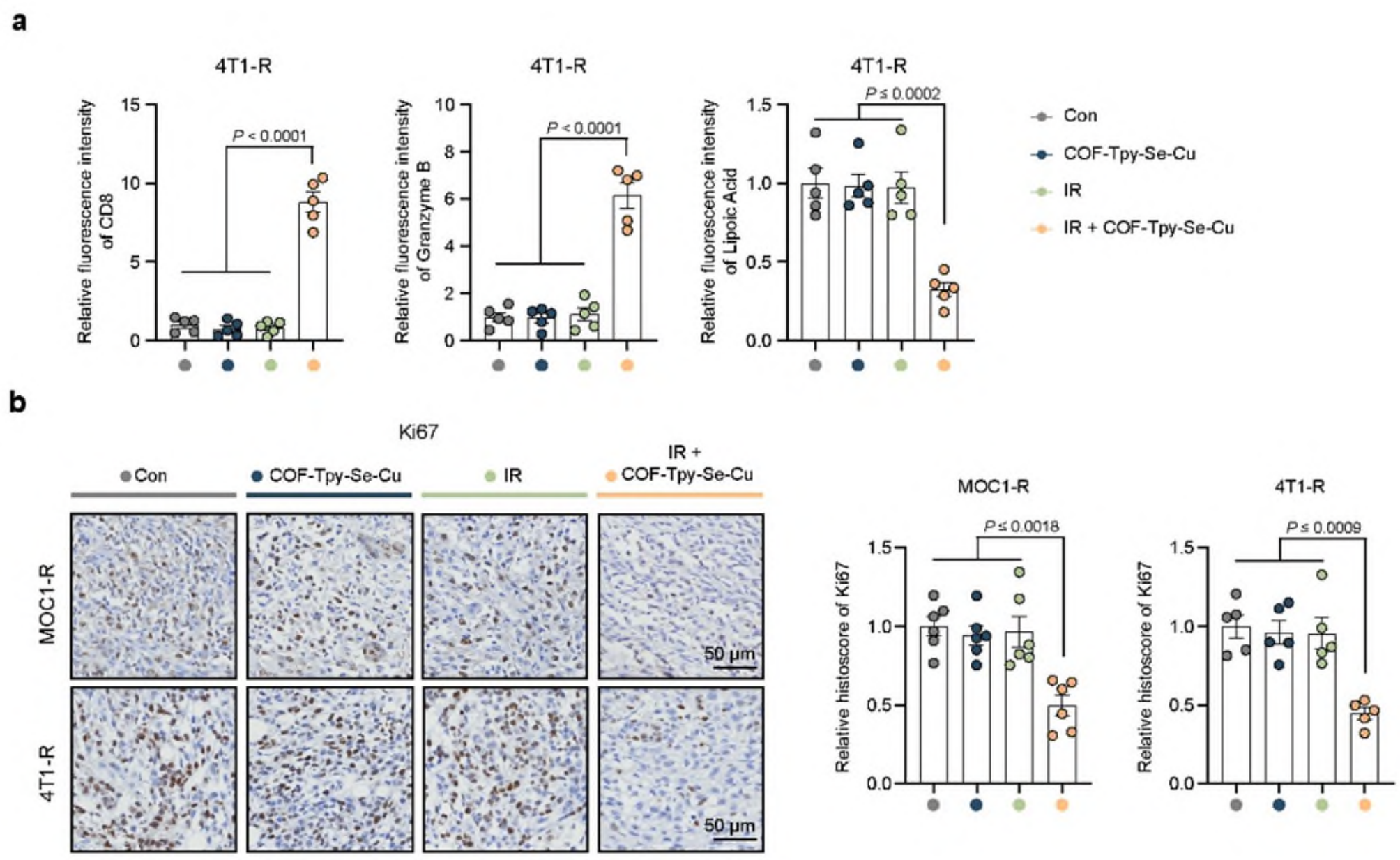

Supplementary Fig. 21| Multiplex immunofluorescence and immunohistochemical analysis of immune activation, cuproptosis hallmarks, and tumor proliferation in vivo. a Quantification of relative fluorescence intensity of CD8, granzyme B, and lipoic acid in 4T1-R tumor sections across the indicated treatment groups, analyzed by multiplex immunohistochemistry (mIHC). **b** Representative immunohistochemistry staining images and quantitative histoscore analysis of the proliferation marker Ki67 in MOC1-R and 4T1-R tumor sections from each treatment group (scale bar, 50 μm). Each point represents an individual tumor. Statistical significance was assessed using one-way ANOVA with Tukey’s multiple-comparisons test (**a, b**). Data are presented as mean ± s.e.m. from at least three independent experiments.

Responses to the comments of Reviewer #3

Comment:

This study utilizes the radioresistant cancer cells have enhanced cuproptosis susceptibility to design a radiation-responsive copper-incorporated COF (COF-Tpy-Se-Cu), which releases Cu^{2+} under X-ray irradiation to trigger cuproptosis. Combined with oncolytic adenovirus (OVAD) — which depletes GSH and stabilizes FDX1 by inhibiting AFG3L2-mediated degradation — this regimen synergistically amplifies cuproptosis, remodels the tumor immune microenvironment, converts cold tumors to hot ones, elicits robust T-cell-dependent antitumor immunity, suppresses metastasis and establishes durable immune memory. In general, the data provided in this study can’t support its conclusions. The novelty also needs further explanation.

Response:

We sincerely appreciate your critical and constructive assessment of our work. We fully acknowledge the concerns regarding insufficient supporting evidence and inadequate articulation of novelty, and we apologize that the original version did not adequately present the evidentiary basis and specific novelty of the study. To address these concerns as carefully as possible, we have added clonogenic survival assays (the gold standard for radiosensitization evaluation), systematic component-deconstruction experiments covering all single agents and pairwise combinations, comprehensive in vivo biodistribution and excretion profiling, quantitative multiplex immunofluorescence validation, and orthogonal copper-dependence validations including scaffold controls, chelator rescue, and FDX1 ablation. We have also supplemented bilateral tumor models for abscopal effect analysis, in vivo T cell depletion assays, and detailed characterization of intratumoral viral replication. We hope that these revisions have substantially reinforced the mechanistic depth and evidentiary rigor of the study. All changes are highlighted in the revised manuscript, with point-by-point responses provided below.

Comment 1:

1. The combination of cuproptosis and re-radiation sensitization have been reported before. In addition, radioresistant cancer cells exhibiting enhanced cuproptosis susceptibility was also not the first time to be identified. The specific novelty of the current work remains to be clearly articulated and emphasized.

Response 1:

We sincerely thank the reviewer for raising this critical and constructive comment. We fully acknowledge that the general concept of cuproptosis-mediated radiosensitization and the enhanced cuproptosis susceptibility of radioresistant cancer cells have been documented in prior studies [3,4]. We apologize that the specific novelty of our work was not sufficiently articulated in the original manuscript. Our work builds on these foundational phenotypic observations and advances the field through three distinct contributions at the molecular, material, and therapeutic strategy levels, as detailed below.

(1) Identification of ATF3 as a previously unrecognized upstream transcriptional regulator of cuproptosis vulnerability in radioresistant cancer cells

While prior studies have reported the phenotypic association between acquired radioresistance and increased cuproptosis sensitivity [3,4], the upstream molecular circuitry driving this phenotype has remained undefined. In this study, we identify the stress-inducible transcription factor ATF3 as a key mediator of this vulnerability. Using RNA sequencing, ChIP assays, and dual-luciferase reporter assays, we show that ATF3 is upregulated in radioresistant cells and directly transactivates the core cuproptosis effector genes *Fdx1* and *Cox17*. These findings provide a mechanistic basis for the increased susceptibility of radioresistant cells to copper-induced proteotoxic stress, moving beyond correlative phenotypic observations (Fig. 2).

(2) A rationally designed COF-based nanoplatform with high physiological stability and predominantly radiation-triggered copper release for targeted radiosensitization

The rationally engineered COF-Tpy-Se-Cu scaffold exhibits high structural stability under diverse pathophysiologically relevant conditions, including

physiological and tumor-mimicking acidic pH, redox gradients, and serum, with negligible spontaneous copper leakage (Fig. 3, Supplementary Fig. 7). Copper release is triggered predominantly by X-ray irradiation, thereby tightly coupling bioavailable copper to the spatiotemporal window of local radiotherapy (Supplementary Fig. 15). Combined with preferential cytotoxicity toward radioresistant tumor cells with intrinsic cuproptosis vulnerability, this design improves the selectivity of local cuproptotic activity while minimizing systemic copper exposure, addressing a notable barrier to clinical translation of copper-based therapeutic strategies (Fig. 4, 5).

(3) A dual-amplification strategy converging on FDX1, pairing nanomaterial-mediated copper delivery with oncolytic virus-mediated sensitization of the endogenous cuproptosis pathway

A well-recognized limitation of cuproptosis-based monotherapies is that their efficacy depends on endogenous FDX1 levels, which decline during cuproptosis execution and may limit sustained cell killing [5-8]. To address this constraint, we developed a combinatorial strategy in which COF-Tpy-Se-Cu delivers exogenous copper as the trigger, while oncolytic adenovirus (OVAD) sensitizes the endogenous cuproptosis machinery.

Critically, we describe a previously unrecognized mechanism: OVAD stabilizes FDX1 protein by inhibiting AFG3L2-mediated mitochondrial proteolytic degradation, and additionally depletes intracellular glutathione (Fig. 6). This virus-mediated modulation lowers the cellular threshold for copper-induced cytotoxicity, such that the two modalities converge on the FDX1 node to exert greater-than-additive synergistic effects (Fig. 7, 8). To our knowledge, this is the first report of an oncolytic virus being leveraged to amplify cuproptosis through direct modulation of a core cuproptosis effector protein.

To ensure that these points of novelty are conveyed more clearly and appropriately, we have comprehensively revised the Abstract and Discussion sections of the manuscript to explicitly contextualize our findings relative to the published literature and delineate our specific contributions.

Specifically in the “Abstract” section:

“**Abstract**

Acquired radioresistance represents a significant obstacle to effective radiotherapy. Cuproptosis, a copper-dependent form of regulated cell death mediated by mitochondrial copper accumulation and lipoylated protein aggregation, has been associated with radiation response. While enhanced cuproptosis susceptibility in radioresistant cancer cells has been documented in prior studies, the upstream regulatory circuitry governing this phenotype remains incompletely characterized, and translational therapeutic strategies targeting this vulnerability remain limited. Here, we confirm heightened cuproptosis sensitivity in radioresistant cancer cells and identify the stress-responsive transcription factor ATF3 as a key upstream regulator that directly transactivates the core cuproptosis effector genes *Fdx1* and *Cox17* to confer this phenotype. To exploit this vulnerability, we developed a radiosensitization strategy that combines a radiation-responsive, copper-incorporated covalent organic framework (COF-Tpy-Se-Cu) with oncolytic adenovirus (OVAD), whose components synergistically converge on the core cuproptosis effector FDX1 to robustly induce cuproptosis. Upon X-ray irradiation, COF-Tpy-Se-Cu releases copper ions to trigger FDX1-mediated cuproptosis. Concurrently, OVAD stabilizes FDX1 by preventing its degradation by the mitochondrial protease, and depletes intracellular glutathione, thereby addressing a central limitation of cuproptosis approaches reliant on endogenous FDX1 expression. Together, COF-Tpy-Se-Cu and OVAD amplify cuproptosis and remodel the tumor immune microenvironment, effectively converting immunologically “cold” tumors into T-cell-inflamed “hot” lesions. This regimen also elicits a robust, T cell–dependent antitumor response that suppresses metastasis and establishes durable immunological memory. Collectively, our study delineates a molecular mechanism linking acquired radioresistance to cuproptosis vulnerability, and presents a combinatorial therapeutic approach.”

Specifically in the “Discussion” section:

"Radiotherapy is a standard treatment for cancer, but its effectiveness is often limited by acquired resistance [9,10]. Building on prior observations linking cuproptosis to cellular radiation response, in this study, we confirm heightened cuproptosis sensitivity in radioresistant cancer cells and identify the stress-responsive transcription factor ATF3 as a key upstream regulator that directly transactivates the core cuproptosis effector genes *Fdx1* and *Cox17* to confer this phenotype. To exploit this vulnerability, we developed a radiosensitization strategy that combines a radiation-responsive, copper-incorporated covalent organic framework (COF-Tpy-Se-Cu) with OVAD, whose components converge on the core cuproptosis effector FDX1 to synergistically induce cuproptosis. Our approach combines controlled copper delivery, viral immunotherapy, and radiotherapy to overcome acquired radioresistance, enhance tumor immunogenicity, and elicit robust systemic antitumor immunity."

Comment 2:

2. How were MOC1, 4T1 and MC38 tumor types chosen? Additionally, the specific design principles of the current nanomaterial (COF-Tpy-Se-Cu) tailored to the biological characteristics of these tumor types, and how it achieves targeted therapeutic effects against them, need to be elaborated in detail.

Response 2:

We sincerely thank the reviewer for this insightful and constructive comment, which has prompted us to more comprehensively clarify the rationale for tumor model selection, the tumor biology-tailored design principles underlying the COF-Tpy-Se-Cu nanosystem, and its mechanism of targeted therapeutic action.

(1) Rationale for tumor model selection

The three tumor models were selected to encompass distinct clinical radiotherapy indications, tumor immune contexts, and therapeutic endpoints, thereby enabling rigorous evaluation of both therapeutic efficacy and the broad generalizability of our cuproptosis-based radiosensitization strategy. All three cell lines were subjected to long-term fractionated irradiation to generate isogenic radioresistant sublines, enabling

direct comparison with parental cells and specifically modeling the clinical scenario of acquired radioresistance.

MOC1 (murine head and neck squamous cell carcinoma): Radiotherapy is a cornerstone of definitive and postoperative management for head and neck squamous cell carcinoma, and acquired radioresistance is a major cause of treatment failure [11,12]. The MOC1-R orthotopic tongue tumor model recapitulates the native tissue architecture, stromal microenvironment, and lymphatic drainage patterns of human oral cancer, providing a physiologically relevant preclinical system for evaluating local radiotherapy response [13-15].

4T1 (murine triple-negative breast cancer): Triple-negative breast cancer is routinely treated with radiotherapy, and the 4T1 model recapitulates key clinical features including high local recurrence rates, spontaneous metastatic dissemination, and an immunologically “cold” tumor microenvironment [16-18]. As a well-validated platform for investigating metastasis and systemic antitumor immunity, this model was included to assess antimetastatic activity and abscopal effects in addition to local radiosensitization.

MC38 (mouse colon adenocarcinoma): Preoperative chemoradiotherapy is the standard of care for locally advanced colorectal cancer. MC38 is a widely adopted immuno-oncology model with moderate immunogenicity, representing a tumor type with a distinct tissue origin and a different baseline immune status from the other two models [19]. It was included to test whether our cuproptosis-based radiosensitization strategy is broadly applicable beyond head and neck and breast cancer. Consistent radiosensitization and cuproptosis induction were confirmed in the MC38-R model, supporting the generalizability of our findings.

(2) Material design principles aligned with tumor biology and clinical radiotherapy

The design of COF-Tpy-Se-Cu is closely aligned with the shared biological features of radioresistant tumors and the clinical practice of local fractionated radiotherapy. Tumor selectivity is achieved at three complementary levels, as detailed

below.

First, tissue-level enrichment via local administration. The material is delivered via intratumoral injection, which concentrates the carrier directly within the tumor parenchyma and minimizes systemic exposure. The covalently crosslinked framework confers high structural stability against pH gradients, redox species, and enzymatic factors in the tumor microenvironment (Supplementary Fig. 7). This stability supports prolonged intratumoral retention after injection, which is particularly important for dense orthotopic tumor tissue such as MOC1-R tongue tumors, and enables responsiveness to repeated fractionated irradiation across all models.

Second, spatiotemporal control via radiation-gated copper release. The radiation-cleavable diselenide bonds in the COF backbone confer predominantly X-ray-dependent copper release (Supplementary Fig. 15). This design tightly couples bioavailable copper delivery to each session of localized radiotherapy, restricting cytotoxic copper activity to the irradiated volume. This feature is universally applicable across all three tumor models and matches the spatiotemporal pattern of clinical fractionated radiotherapy.

Third, cellular-level selectivity via exploitation of intrinsic metabolic vulnerability. Radioresistant cancer cells across all three models show upregulated expression of FDX1 and COX17 and heightened susceptibility to cuproptosis (Fig. 2). By delivering copper in a radiation-controlled manner, the material confers preferential cytotoxicity toward radioresistant tumor subpopulations, while sparing cells with lower cuproptosis sensitivity. This metabolic selectivity operates independently of tissue origin and thus supports efficacy across diverse tumor types.

To ensure that these points are clearly communicated, we have elaborated the corresponding sections in the Results and Discussion of the revised manuscript.

Specifically in the manuscript:

"Acquired radioresistance is a major barrier to effective radiotherapy [20]. To investigate this phenotype and evaluate candidate therapeutic strategies, we selected a panel of three representative tumor cell lines encompassing distinct histological origins

and radiobiological contexts: MOC1 (murine head and neck squamous cell carcinoma), 4T1 (murine triple-negative breast cancer), and MC38 (murine colon adenocarcinoma). This panel was chosen to encompass diverse tissue origins, established clinical radiotherapy indications, and distinct baseline immune microenvironments, enabling evaluation of both antitumor efficacy and the broader generalizability of our approach [15-19]. An additional murine esophageal squamous cell carcinoma line, AKR, was included for parallel mechanistic validation. To uniformly recapitulate the clinical scenario of acquired radioresistance, we derived isogenic radioresistant sublines from each parental line through long-term fractionated irradiation (2 Gy per fraction, 30 fractions total) (Fig. 2a)."

Specifically in the "Discussion" section:

"To therapeutically target this defined vulnerability, we rationally designed COF-Tpy-Se-Cu with material principles tailored to the shared biological features of radioresistant tumors and the clinical practice of local fractionated radiotherapy across multiple cancer types [9,11,21-23]. Its covalently crosslinked scaffold incorporates radiation-cleavable diselenide (–Se–Se–) bonds [24-26], which undergo selective cleavage under X-ray irradiation to trigger localized copper release within tumor tissue. This radiation-responsive release behavior is engineered to align with clinical fractionated radiotherapy regimens, with the goal of achieving on-demand copper delivery coupled to each radiation session. Additionally, the robust covalent framework confers high structural stability under pathophysiologically relevant tumor microenvironmental conditions, which is expected to support prolonged intratumoral retention and responsiveness to repeated irradiation. Collectively, this design minimizes systemic copper exposure and selectively exploits the intrinsic cuproptosis vulnerability of radioresistant cancer cells with upregulated FDX1 and COX17 expression, enabling preferential elimination of treatment-resistant tumor subpopulations. COF-Tpy-Se-Cu acts as a radiosensitizer by triggering FDX1-dependent cuproptosis and enhancing tumor immunogenicity, as evidenced by elevated release of DAMPs such as HMGB1,

ATP, and CRT."

Comment 3:

3. Considering the specific tumor type, orthotopic tumor models should be established to better illustrate therapeutic effects.

Response 3:

We sincerely thank the reviewer for emphasizing the importance of orthotopic tumor models, a consideration that was central to our experimental design. We would like to clarify that the MOC1-R model used throughout this study is a well-established orthotopic tongue tumor model, commonly used as a preclinical platform for head and neck cancer radiotherapy research [13,14,27]. Tumor cells are inoculated into the tongue parenchyma of immunocompetent mice, which recapitulates the native tissue architecture, stromal microenvironment, and lymphatic drainage patterns of human oral squamous cell carcinoma.

During the revision process, we have further strengthened our validation in the MOC1-R orthotopic model. All core findings—including in vivo material biodistribution and excretion profiles, biochemical hallmarks of cuproptosis, tumor growth suppression, and immune microenvironment remodeling—have been verified in this orthotopic setting, with parallel corroboration in the 4T1-R subcutaneous model. The consistent therapeutic efficacy observed across both orthotopic and subcutaneous tumor models further supports the robustness and clinical translational potential of our strategy.

The 4T1 subcutaneous model was retained primarily for bilateral tumor abscopal studies and experimental lung colonization assays [16-18,28]. Notably, the subcutaneous 4T1 model is a widely adopted and well-validated preclinical system for these specific research endpoints [16-18,28], as it enables precise, highly reproducible tumor volume monitoring and consistent pulmonary metastatic seeding—endpoints that are technically challenging to assess and prone to high variability in orthotopic mammary fat pad models. This design aligns directly with our study goals of evaluating systemic

antitumor immunity and antimetastatic efficacy.

The full panel of therapeutic efficacy data for all eight treatment groups in the MOC1-R orthotopic model is presented in Supplementary Fig. 32, which includes the in vivo treatment schedule, tumor growth curves, and representative images of excised orthotopic tongue tumors at the study endpoint.

We consider that this multi-model approach—an orthotopic head and neck cancer model for assessing local radiotherapy efficacy, paired with a subcutaneous breast cancer model for evaluating systemic immunity and metastatic outcomes—provides both clinically relevant local efficacy data and comprehensive mechanistic validation of systemic antitumor effects. To eliminate any ambiguity, we have further clarified the model specifications in the corresponding figure legends.

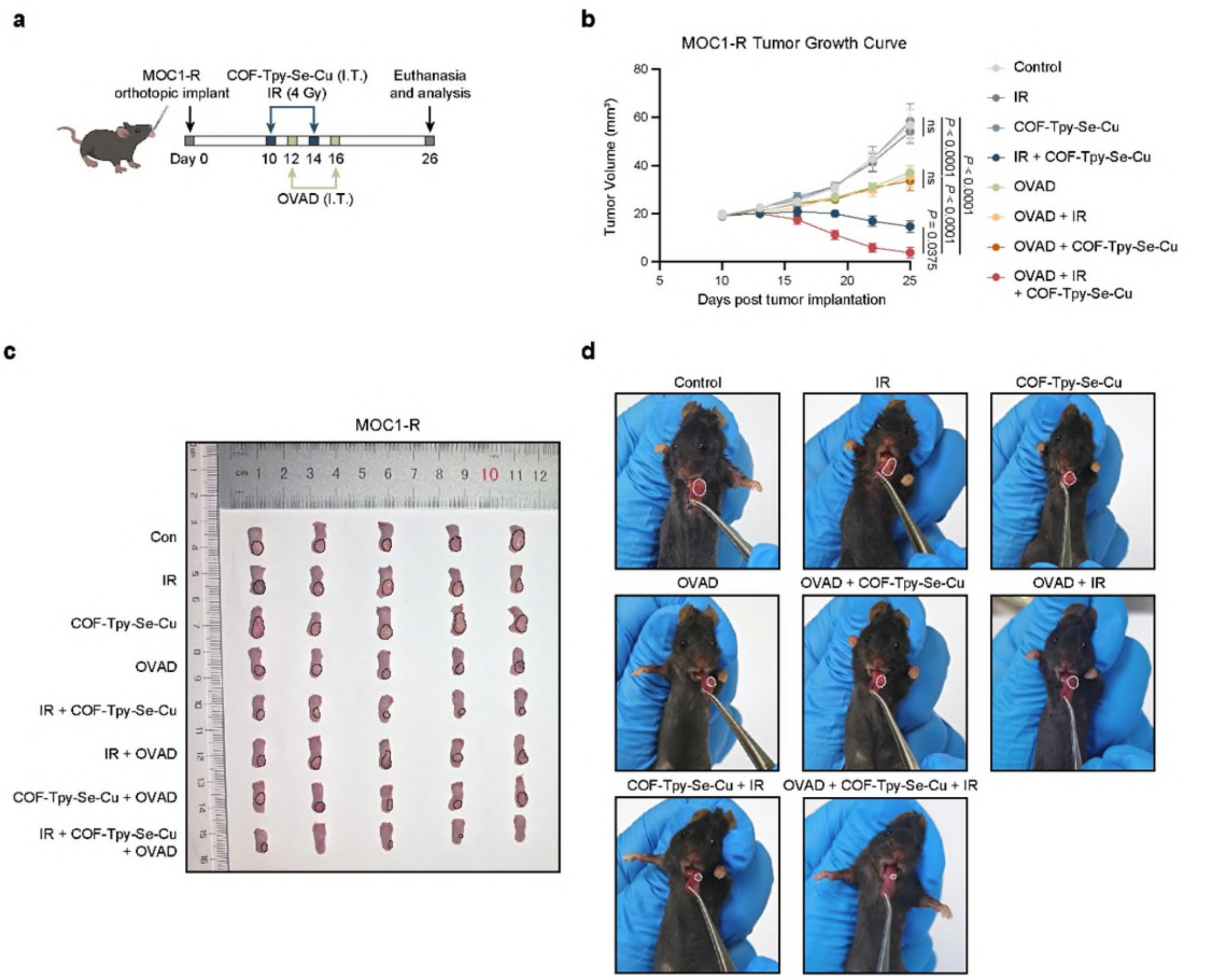

Supplementary Fig. 32| Synergistic radiosensitization efficacy of COF-Tpy-Se-Cu combined with oncolytic adenovirus in vivo. a In vivo treatment schedule. MOC1-R tumor-bearing mice received intratumoral injections of COF-Tpy-Se-Cu (2 mg kg^{-1}) and/or OVAD (0.4×10^6 PFU), combined with localized irradiation (IR, 4 Gy) as indicated. **b** Tumor growth curves for MOC1-R orthotopic tumors under the indicated treatments (n = 5 mice per group). **c** Representative images

of excised tongues from mice bearing orthotopic MOC1-R tumors at the endpoint (n = 5 mice per group). Tumor boundaries are delineated by black dashed lines. **d** Representative images of mice bearing orthotopic MOC1-R tongue tumor across eight treatment groups. Tumor boundaries are delineated with white dashed lines. Statistical significance was assessed using two-way ANOVA (**b**). Data are presented as mean ± s.e.m. from at least three independent experiments.

Comment 4:

4. In vivo biodistribution and excretion profiles of as-prepared medicine should be studied.

Response 4:

We sincerely thank the reviewer for this valuable comment. We agree that biodistribution and excretion profiling is important for evaluating the in vivo behavior and safety of the as-prepared material. To systematically characterize the in vivo biodistribution and clearance of COF-Tpy-Se-Cu following intratumoral administration, we performed complementary analyses using ex vivo fluorescence imaging and inductively coupled plasma mass spectrometry (ICP-MS). Our findings are summarized below.

(1) Ex vivo fluorescence imaging (Supplementary Fig. 12a, b)

Cy5.5 was loaded into the COF pores as a fluorescent tracer. At 7 days post-injection, we harvested tumor tissues and major organs from both MOC1-R orthotopic and 4T1-R subcutaneous tumor models for ex vivo imaging. Fluorescent signals were most intense in tumor tissues, with detectable accumulation also observed in the liver and spleen. Signals in the heart, lungs, and kidneys were minimal, confirming favorable local retention of the intratumorally delivered material.

(2) Time-resolved quantitative biodistribution by ICP-MS (Supplementary Fig. 12c)

We then selected the more clinically relevant MOC1-R orthotopic model for detailed time-resolved quantitative analysis. We used an independent cohort of MOC1-R orthotopic tumor-bearing mice that received unlabeled native COF-Tpy-Se-Cu (without Cy5.5 loading). Tissues were harvested at serial time points, weighed, and subjected to microwave-assisted acid digestion. Selenium content in tissues was

measured via ICP-MS over a 30-day monitoring period, using selenium as an intrinsic elemental tracer covalently integrated into the COF backbone to reflect the in vivo fate of the intact nanomaterial. Endogenous selenium background from untreated controls was subtracted for all quantitative calculations. Consistent with the fluorescence imaging results, among the normal organs examined, the material preferentially accumulated in the major mononuclear phagocyte system (MPS) organs (i.e., the liver and spleen), with tissue concentrations peaking at days 7–14 followed by gradual clearance. Levels in the heart, lungs, and kidneys remained low throughout the observation window.

(3) Excretion profiling (Supplementary Fig. 12d)

Daily fecal and urinary excretion rates of COF-Tpy-Se-Cu were monitored for 30 days using metabolic cages, applying the same selenium-based tracer strategy and endogenous background correction as described above. Fecal excretion exhibited an initial rise followed by a gradual decline, peaking at approximately days 9–12. In contrast, urinary excretion remained at very low levels at all time points. These data indicate that fecal excretion via the hepatobiliary pathway is the primary clearance route for COF-Tpy-Se-Cu.

This distribution and clearance pattern is consistent with the favorable systemic safety profile supported by serum biochemistry and histopathological analyses.

All corresponding data have been added to the revised manuscript and are presented in Supplementary Fig. 12, with a full description included in the main Results section. Detailed experimental protocols have also been added to the Methods section.

The updated manuscript text reads as follows:

"We next evaluated the in vivo biodistribution and excretion profiles of COF-Tpy-Se-Cu following intratumoral administration. For semi-quantitative ex vivo fluorescence assessment, Cy5.5 was loaded into the COF pores to serve as a fluorescent tracer. Major organs and tumor tissues were harvested at 7 days post-injection for ex vivo fluorescence imaging (Supplementary Fig. 12a). In both MOC1-R orthotopic and 4T1-R subcutaneous tumor models, fluorescent signals were predominantly retained in

tumor tissues, with apparent accumulation also detected in the liver and spleen, and negligible signals observed in the heart, lungs, and kidneys (Supplementary Fig. 12b). Quantitative analysis of average radiant efficiency confirmed that the signal intensity in tumors was significantly higher than that in all examined normal organs (Supplementary Fig. 12b), demonstrating the favorable local retention effect of intratumoral delivery. For accurate, time-resolved quantification of biodistribution, we used an independent cohort of tumor-bearing mice that received unlabeled native COF-Tpy-Se-Cu (without Cy5.5 loading). Tissues were harvested at serial time points, weighed, and subjected to acid digestion. Selenium, an intrinsic elemental constituent of the COF backbone, was used as an elemental tracer, and its content in each tissue was measured via ICP-MS. Among the normal organs examined, the material preferentially accumulated in the major mononuclear phagocyte system (MPS) organs (i.e., the liver and spleen), with tissue concentrations peaking between days 7 and 14 followed by gradual clearance (Supplementary Fig. 12c). Only minimal amounts of the material were detected in the heart, lungs, and kidneys throughout the 30-day monitoring period (Supplementary Fig. 12c).

To define the excretion profile, we monitored daily fecal and urinary excretion rates over 30 days using metabolic cages, with selenium content in fecal and urinary samples quantified by ICP-MS using the same elemental tracer strategy described above. The daily fecal excretion rate exhibited an initial rise followed by a gradual decline, peaking at approximately days 9–12. By contrast, urinary excretion remained consistently low at all time points, indicating that fecal elimination via the hepatobiliary pathway is the dominant clearance route for COF-Tpy-Se-Cu (Supplementary Fig. 12d).”

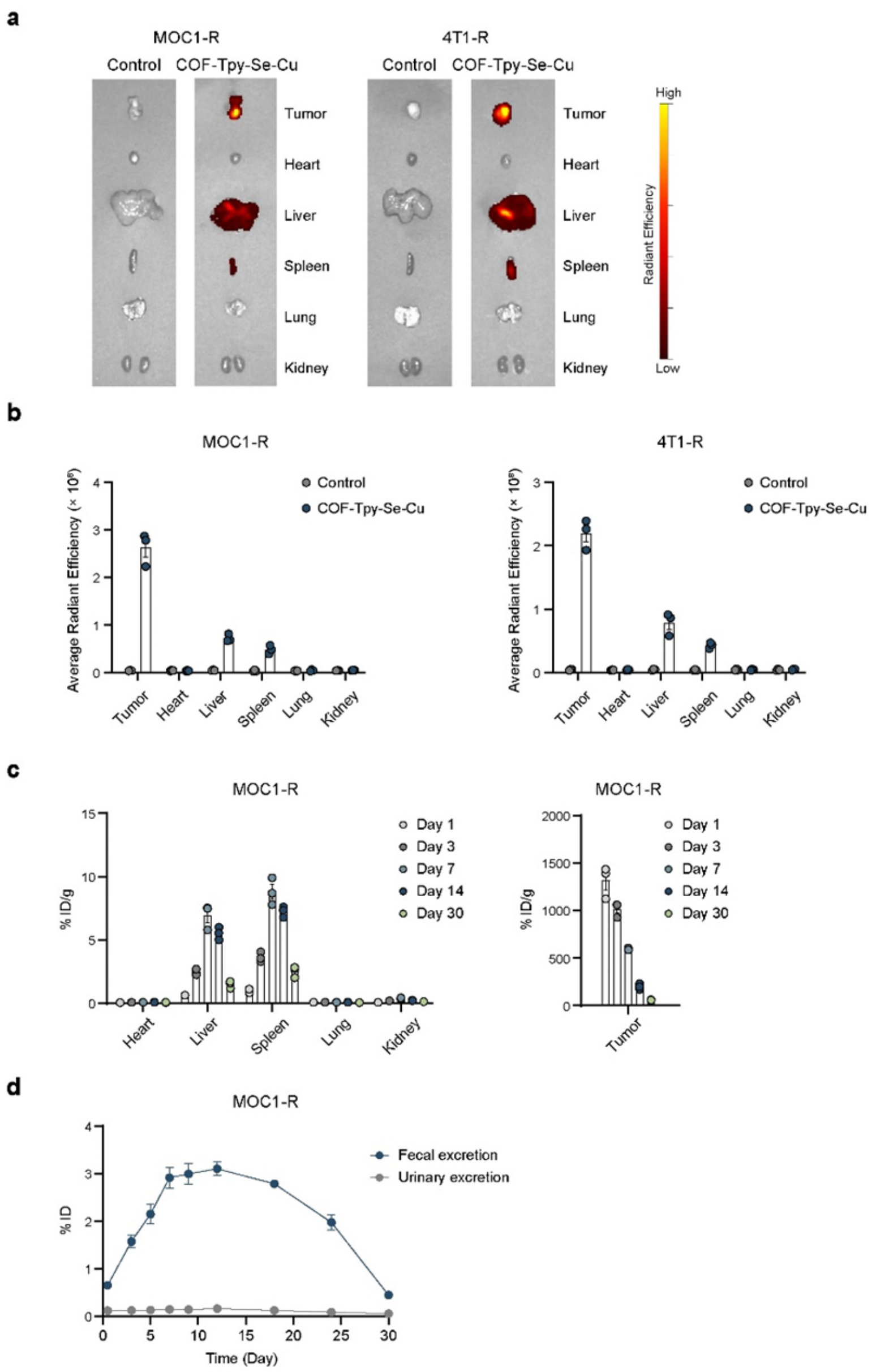

Supplementary Fig. 12| In vivo biodistribution and excretion profile of COF-Tpy-Se-Cu in tumor-bearing mice. a Ex vivo fluorescence imaging of tumor tissues and major organs (heart, liver, spleen, lung, kidney) from mice bearing MOC1-R orthotopic or 4T1-R subcutaneous tumors at 7 days after intratumoral injection of Cy5.5-loaded COF-Tpy-Se-Cu. The color scale indicates relative radiant efficiency. **b** Quantitative analysis of average radiant efficiency in tumor and each organ. **c** Time-course biodistribution of COF-Tpy-Se-Cu in major organs (left) and tumor tissues (right) of MOC1-R tumor-bearing mice following intratumoral injection, quantified by inductively coupled plasma mass spectrometry (ICP-MS) using selenium as the elemental tracer. COF-Tpy-Se-Cu content is expressed as the percentage of injected dose per gram of tissue (% ID/g). **d** Daily fecal and urinary excretion rates of COF-Tpy-Se-Cu in MOC1-R tumor-bearing mice over 30 days post-injection. Data are presented as the percentage of injected dose per day (% ID/day). Data are presented as

mean ± s.e.m. from at least three independent experiments.

Specifically in the "Methods" section:

"Biodistribution and excretion study of COF-Tpy-Se-Cu

Female C57BL/6 mice bearing MOC1-R orthotopic tongue tumors were employed for in vivo biodistribution and excretion assays. For ex vivo fluorescence imaging, Cy5.5-loaded COF-Tpy-Se-Cu (2 mg kg^{-1} per mouse) was administered via intratumoral injection. At 7 days post-injection, mice were euthanized, and major organs (heart, liver, spleen, lung, kidney) along with tumor tissues were excised and imaged using an in vivo optical imaging system. The average radiant efficiency of each tissue was quantified for semi-quantitative evaluation of material distribution. For quantitative time-course biodistribution analysis, unlabeled native COF-Tpy-Se-Cu (2 mg kg^{-1} per mouse) was intratumorally injected. Mice were sacrificed at predetermined time points (1, 3, 7, 14, and 30 days post-administration). Tumors and major organs were collected, accurately weighed, and digested via microwave-assisted acid digestion. Selenium, covalently integrated into the COF framework as an intrinsic elemental tracer, was quantified by ICP-MS. All quantitative results were corrected by subtracting the average endogenous selenium background from untreated controls. Material accumulation in each tissue was normalized to tissue weight and expressed as percentage of injected dose per gram of tissue (% ID/g).

For excretion profiling, tumor-bearing mice were individually housed in metabolic cages immediately after injection. Urine and fecal samples were collected at serial intervals over a 30-day monitoring period. Feces were lyophilized, ground into fine powder, and digested following the same acid digestion protocol. Urine samples were diluted with dilute nitric acid prior to ICP-MS measurement. The baseline selenium levels in feces and urine were also determined using samples from the same untreated control cohort, and all excretion data were corrected by subtracting the corresponding endogenous selenium background. Daily fecal and urinary excretion rates were calculated and expressed as the percentage of injected dose per day."

Comment 5:

5. The framework contains too many components, underlying mechanism of individual components, pairwise combinations or the full composite systems haven't been systematically studied.

Response 5:

We sincerely thank the reviewer for this insightful and constructive comment regarding the systematic dissection of our multi-component therapeutic system. We fully acknowledge that the original presentation did not sufficiently delineate the contribution of each individual component or clarify the mechanistic basis of the combinatorial effects. To address this concern, we established a complete panel of experimental groups covering single agents, all three pairwise combinations, and the triple-combination regimen, which were validated both in vitro (Supplementary Fig. 30) and in vivo (Supplementary Fig. 32) in two independent models, to systematically deconvolve the function of each element and their interactions. Our detailed findings are summarized below.

(1) Activity of individual components

We first defined the baseline efficacy of each single component to characterize its independent contribution.

Ionizing radiation (IR): At the dose employed in this study (4 Gy), IR induced only minimal cell death in both MOC1-R and 4T1-R radioresistant cell lines (Supplementary Fig. 30a, b), consistent with their acquired radioresistant phenotype. In vivo, IR monotherapy exerted no marked tumor growth inhibition in either the MOC1-R orthotopic tongue model or the 4T1-R subcutaneous model (Supplementary Fig. 32).

COF-Tpy-Se-Cu: In the absence of irradiation, COF-Tpy-Se-Cu exhibited negligible cytotoxicity in vitro (Supplementary Fig. 30a, b), in line with its high structural stability and minimal spontaneous copper leakage under physiological conditions. Correspondingly, COF-Tpy-Se-Cu monotherapy produced no significant tumor growth delay in vivo (Supplementary Fig. 32).

Oncolytic adenovirus (OVAD): OVAD monotherapy induced moderate cytolytic

activity in vitro (Supplementary Fig. 30a, b) and partial tumor growth inhibition in vivo (Supplementary Fig. 32), reflecting its intrinsic oncolytic effect. As a single agent, however, this level of activity was insufficient to overcome the radioresistant phenotype.

(2) Analysis of pairwise combinations

We next evaluated all three pairwise combinations to dissect potential cooperative interactions:

IR + COF-Tpy-Se-Cu: This dual regimen induced significantly increased cell death compared with either monotherapy in both cell lines (Supplementary Fig. 30a, b), and substantially suppressed tumor growth in vivo (Supplementary Fig. 32). This combination forms the core radiation-triggered cuproptosis module of our system. The copper- and FDX1-dependence of this radiosensitizing effect has been fully validated through orthogonal approaches, including a copper-free scaffold control (Supplementary Fig. 22), in vitro copper chelation rescue assays (Supplementary Fig. 23), and genetic ablation of FDX1 (Supplementary Fig. 24). At the molecular level, this dual effect is further supported by copper release profiling (Supplementary Fig. 7), subcellular copper quantification (Supplementary Fig. 15), and canonical hallmarks of cuproptosis such as DLAT oligomerization (Supplementary Fig. 15).

IR + OVAD: No statistically significant enhancement of cell death (Supplementary Fig. 30a, b) or tumor growth inhibition (Supplementary Fig. 32) was observed for the IR + OVAD dual regimen relative to OVAD alone. This result indicates that direct synergy between IR and viral oncolysis is not the primary driver of the triple-combination efficacy, and that the major cooperative effect is mediated through modulation of the cuproptosis pathway.

COF-Tpy-Se-Cu + OVAD (without irradiation): Compared with OVAD monotherapy, the combination of COF-Tpy-Se-Cu and OVAD in the absence of irradiation did not significantly increase cell death in radioresistant cells (Supplementary Fig. 30a, b), nor did it produce notable tumor growth suppression in vivo (Supplementary Fig. 32). This finding aligns with the low level of spontaneous copper release from the intact COF scaffold in the absence of radiation triggering.

(3) Efficacy of the triple-combination regimen

The triple combination of IR, COF-Tpy-Se-Cu, and OVAD resulted in the greatest extent of cell death in vitro (Supplementary Fig. 30a, b) and the most robust suppression of tumor growth in both the MOC1-R orthotopic and 4T1-R subcutaneous models (Supplementary Fig. 32), outperforming all individual components and all pairwise combinations. At the mechanistic level, the triple combination further promoted DLAT oligomerization, a canonical biochemical hallmark of cuproptosis (Supplementary Fig. 30e). The elevated DLAT oligomerization induced by the triple combination was completely abrogated by the copper chelator TTM, confirming that this enhanced molecular phenotype remains copper-dependent (Supplementary Fig. 30f). In our proposed model, radiation-triggered copper release from the COF scaffold provides the exogenous cytotoxic trigger, whereas OVAD-mediated FDX1 stabilization and concurrent GSH depletion potentiate the endogenous cuproptotic response (Fig. 8j).

Taken together, this full matrix of experimental groups enables systematic deconvolution of each component's contribution and the mechanistic basis of the combinatorial effect, thereby addressing the reviewer's concern. We appreciate the reviewer's suggestion, which has helped us present the contribution of each component more rigorously. All corresponding data have been incorporated into the revised manuscript and supplementary files.

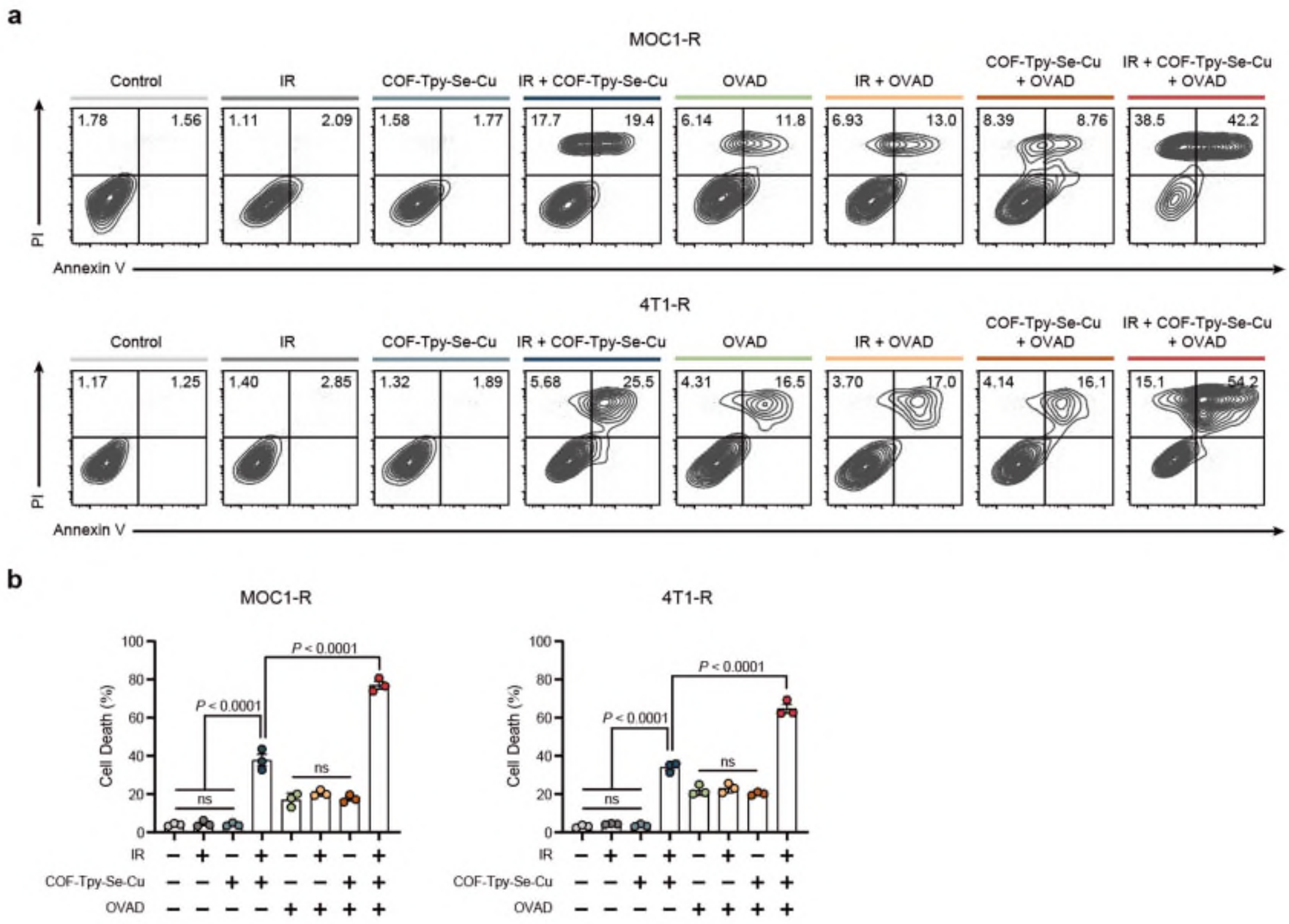

Supplementary Fig. 30| Synergistic radiosensitization efficacy of COF-Tpy-Se-Cu combined with an oncolytic adenovirus in vitro. a Flow cytometric analysis of Annexin V/PI staining in radioresistant MOC1-R (upper) and 4T1-R (lower) cells at 24 h after the indicated treatments (COF-Tpy-Se-Cu, 30 μg mL^{-1}; IR, 4 Gy; OVAD, MOI = 1). **b** Quantification of cell death, expressed as the percentage of PI-positive cells, in MOC1-R (left) and 4T1-R (right) cells. Statistical significance was assessed using one-way ANOVA with Tukey's multiple-comparisons test (**b**). Data are presented as mean ± s.e.m. from at least three independent experiments.

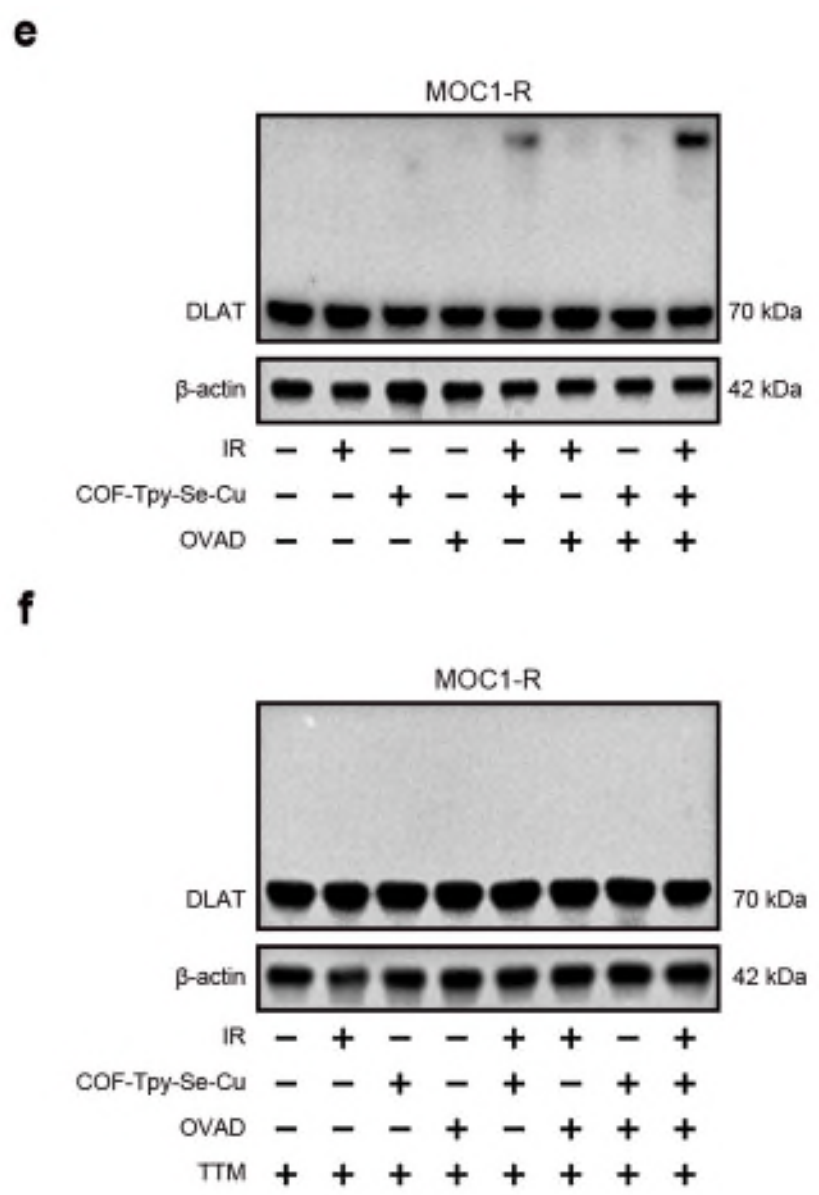

Supplementary Fig. 30| e, f Immunoblot analysis of DLAT oligomerization in MOC1-R cells under the indicated treatments (COF-Tpy-Se-Cu, 30 μg mL^{-1}; IR, 4 Gy; OVAD, MOI = 1; TTM, 40 μM).

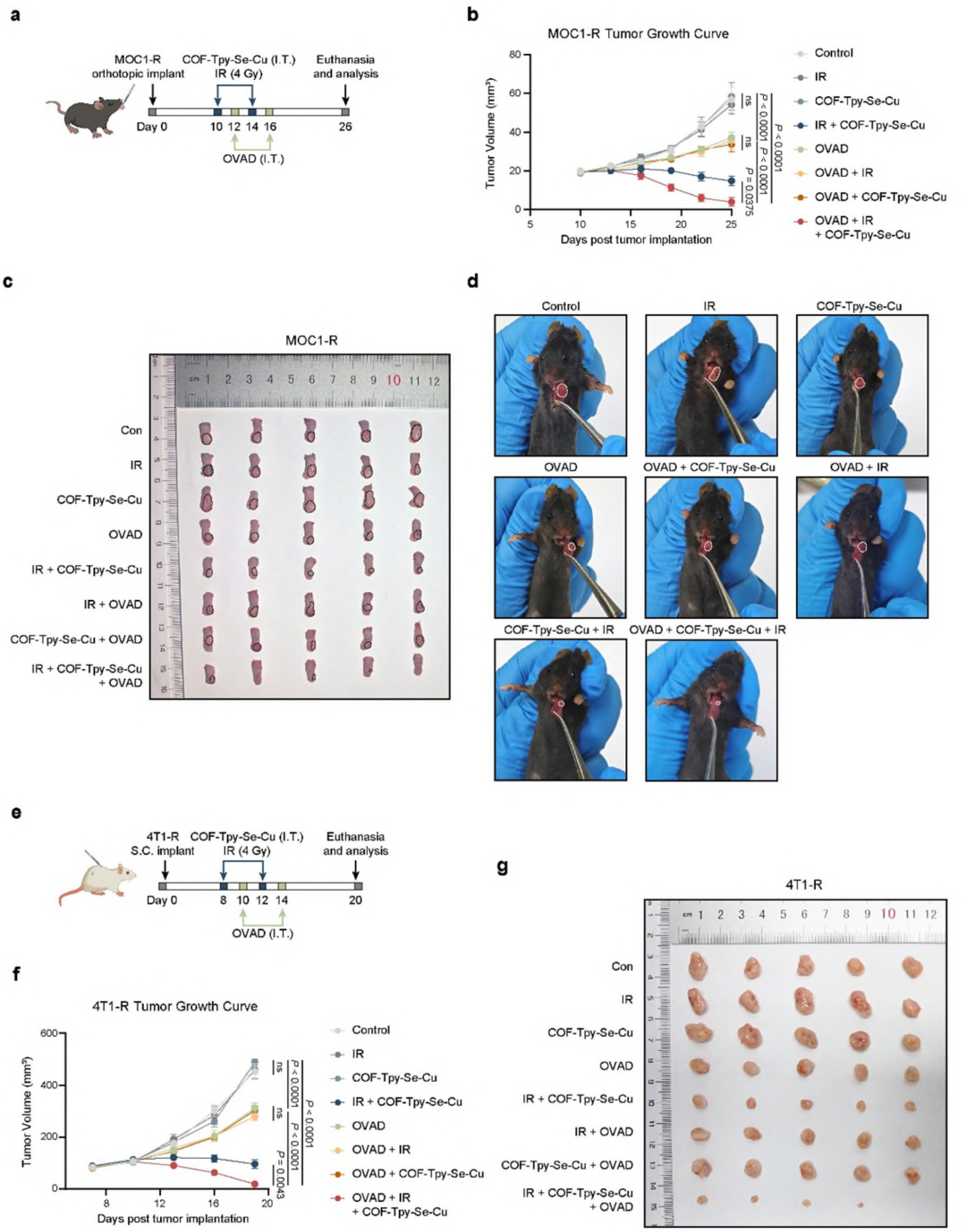

Supplementary Fig. 32| Synergistic radiosensitization efficacy of COF-Tpy-Se-Cu combined with oncolytic adenovirus in vivo. a In vivo treatment schedule. MOC1-R tumor-bearing mice received intratumoral injections of COF-Tpy-Se-Cu (2 mg kg^{-1}) and/or OVAD (0.4 × 10^{6} PFU), combined with localized irradiation (IR, 4 Gy) as indicated. **b** Tumor growth curves for MOC1-R orthotopic

tumors under the indicated treatments (n = 5 mice per group). **c** Representative images of excised tongues from mice bearing orthotopic MOC1-R tumors at the endpoint (n = 5 mice per group). Tumor boundaries are delineated by black dashed lines. **d** Representative images of mice bearing orthotopic MOC1-R tongue tumor across eight treatment groups. Tumor boundaries are delineated with white dashed lines. **e** In vivo treatment schedule. 4T1-R tumor-bearing mice received intratumoral injections of COF-Tpy-Se-Cu (5 mg kg^{-1}) and/or OVAD (2×10^6 PFU), combined with localized irradiation (IR, 4 Gy) as indicated. **f** Tumor growth curves for 4T1-R xenografts under the indicated treatments (n = 5 mice per group). **g** Representative images of excised subcutaneous 4T1-R tumors from each treatment group at the endpoint (n = 5 mice per group). Statistical significance was assessed using two-way ANOVA (**b, f**). Data are presented as mean ± s.e.m. from at least three independent experiments.

j

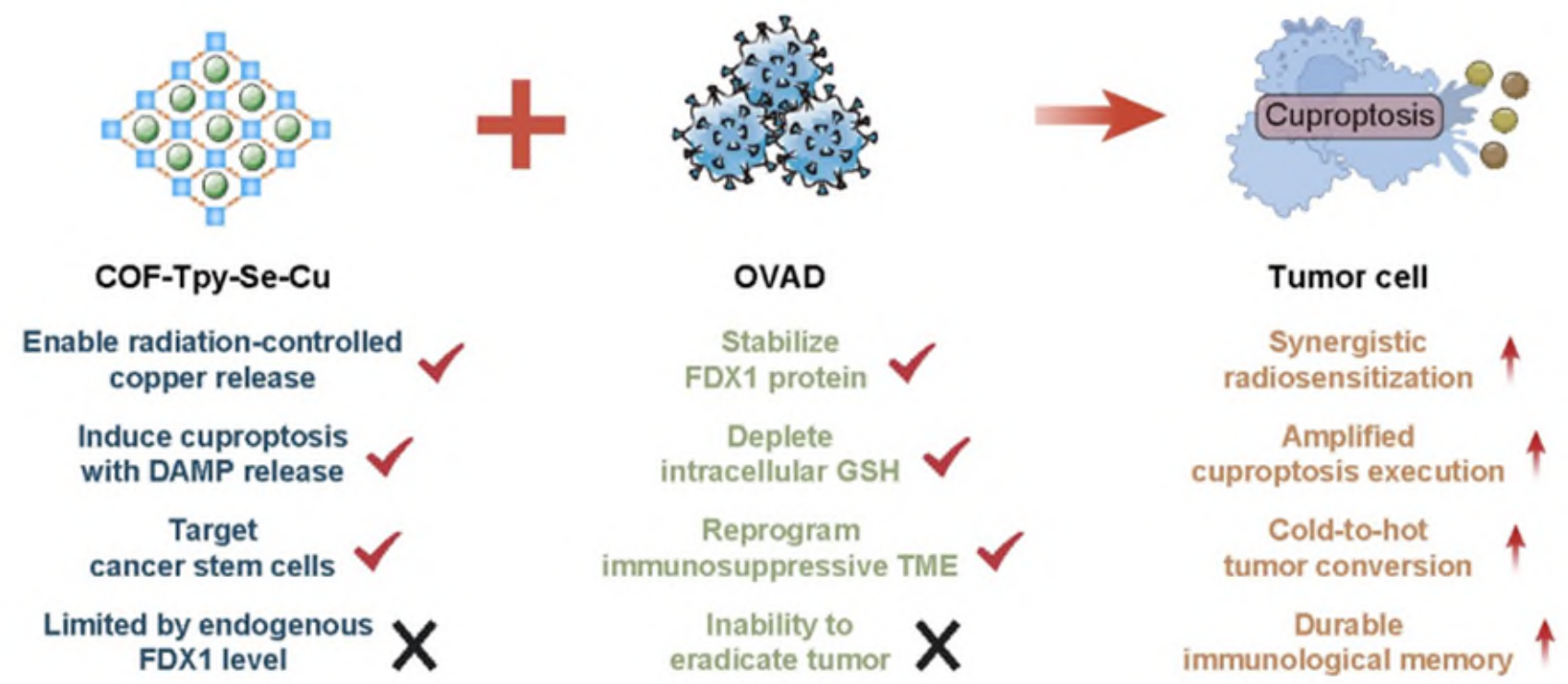

Fig. 8| j Schematic overview of the proposed treatment strategy, which synergistically induces cuproptosis, overcomes acquired treatment resistance, reprograms the tumor immune microenvironment, and suppresses metastasis.

Comment 6:

6. To assess radiotherapeutic effects, colony formation experiment is a golden standard. This core data is missing.

Response 6:

We sincerely thank the reviewer for this insightful and constructive comment. We fully agree that the clonogenic survival assay is the gold standard for assessing long-term proliferative capacity and bona fide radiosensitizing efficacy, and we apologize for the omission of this core dataset in the original submission. In response, we have performed comprehensive clonogenic assays assessing both the radiosensitization effect of the dual-modality regimen (COF-Tpy-Se-Cu plus irradiation) and the enhanced efficacy of the triple-modality combination

(COF-Tpy-Se-Cu combined with OVAD and irradiation), as detailed below.

(1) Radiosensitization by COF-Tpy-Se-Cu in combination with irradiation

Clonogenic assays were performed in both MOC1-R and 4T1-R radioresistant cell lines. Surviving fractions were calculated by normalizing the plating efficiency of each treatment group to that of the untreated control group. As shown in Supplementary Fig. 9a, b, irradiation alone resulted in minimal reduction in colony formation relative to untreated controls, consistent with the acquired radioresistant phenotype of these cell lines. In contrast, COF-Tpy-Se-Cu plus irradiation significantly suppressed clonogenic survival compared with irradiation monotherapy in both cell lines, confirming the robust radiosensitizing activity of COF-Tpy-Se-Cu.

(2) Clonogenic survival assays further corroborating the enhanced radiosensitizing efficacy of this triple regimen

These assays were performed in MOC1-R cells, a head and neck squamous cell carcinoma line with direct clinical relevance to radiotherapy. This cell line also serves as the cellular basis for our in vivo orthotopic tumor model. The triple combination yielded the lowest surviving fraction across all treatment groups, demonstrating the most potent suppression of clonogenic capacity in radioresistant cells (Supplementary Fig. 30c, d).

Corresponding descriptions have been incorporated into the Results section of the revised manuscript, as excerpted below:

"We further validated the radiosensitizing effect using the clonogenic survival assay, the gold standard for assessing long-term proliferative capacity and radiotherapeutic efficacy. In both MOC1-R and 4T1-R radioresistant cell lines, the combination of COF-Tpy-Se-Cu and irradiation effectively suppressed colony formation compared with either monotherapy (Supplementary Fig. 9a, b)."

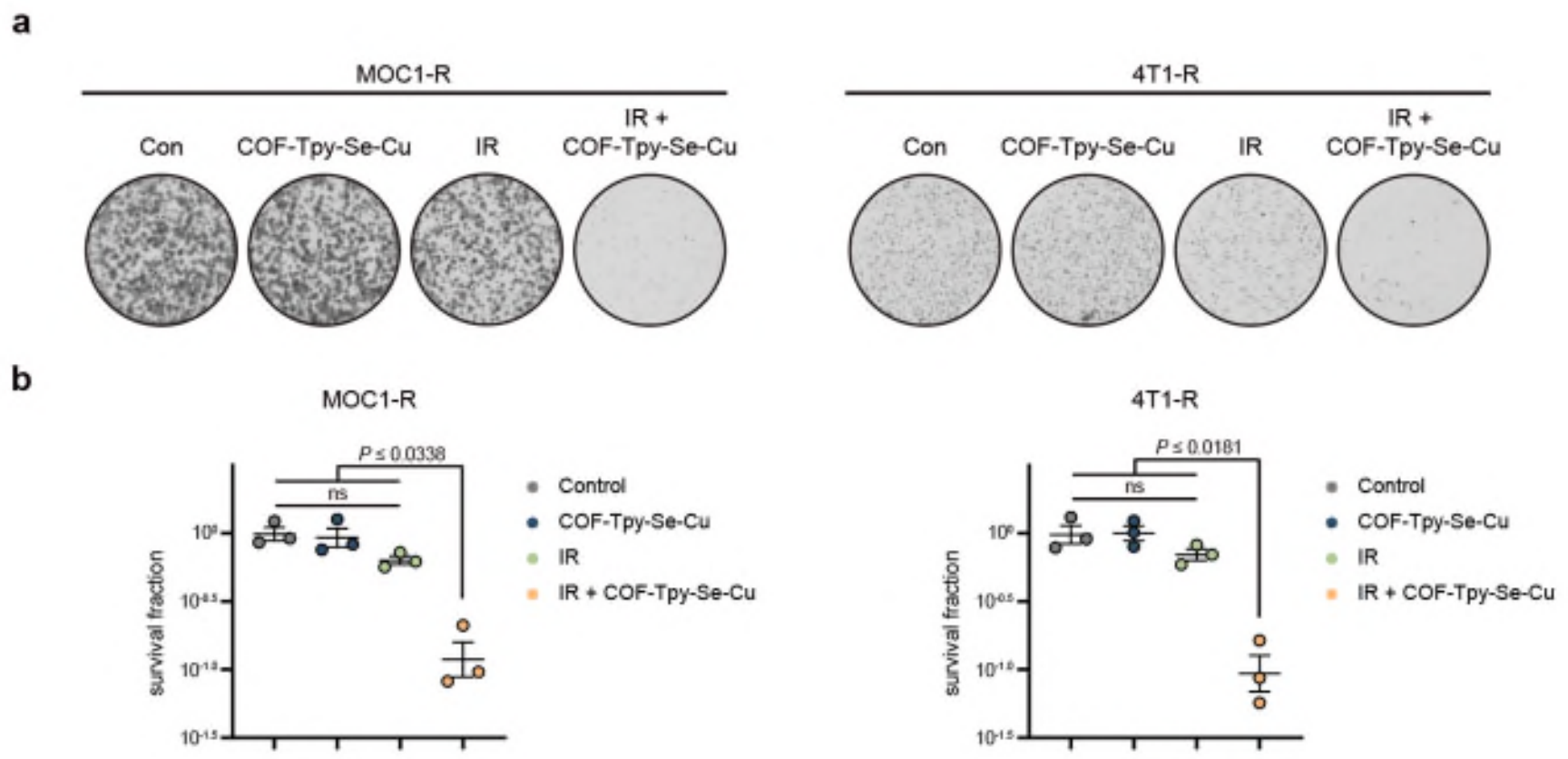

Supplementary Fig. 9| a Representative images of crystal violet-stained colonies of MOC1-R (left) and 4T1-R cells (right) following the indicated treatments (COF-Tpy-Se-Cu, 30 μg mL^{-1}; IR, 4 Gy) after 7 days. **b** Quantification of survival fraction from clonogenic survival assays. Statistical significance was assessed using one-way ANOVA with Tukey's multiple-comparisons test (**b**). Data are presented as mean ± s.e.m. from at least three independent experiments.

"Clonogenic survival assays further corroborated the synergistic radiosensitizing effect of this regimen. The triple combination of OVAD, irradiation, and COF-Tpy-Se-Cu yielded the lowest surviving fraction, confirming durable suppression of the clonogenic capacity of radioresistant cells (Supplementary Fig. 30c, d)."

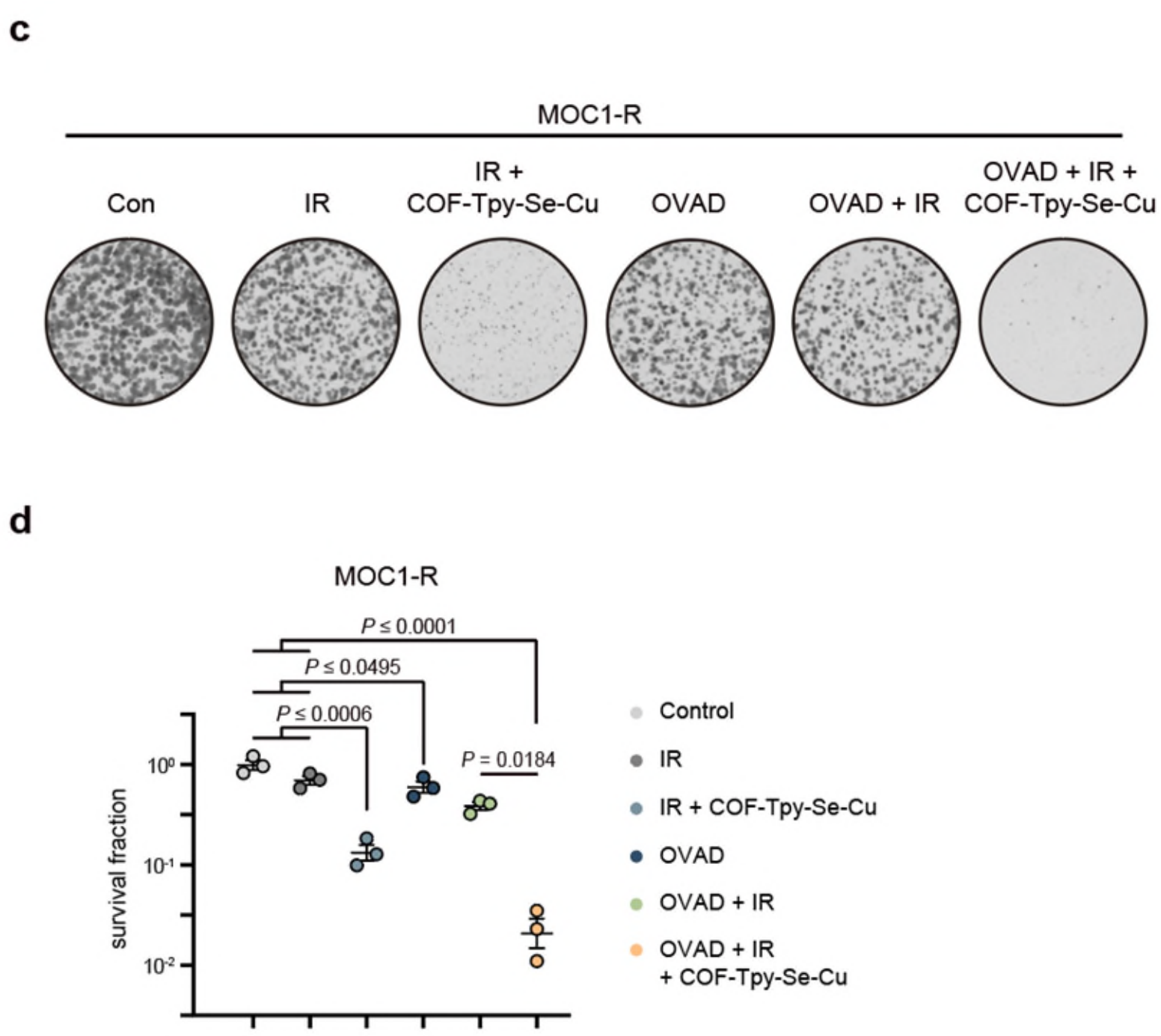

Supplementary Fig. 30| c Representative images of crystal violet-stained colonies of MOC1-R cells following the indicated treatments (COF-Tpy-Se-Cu, 30 μg mL^{-1}; IR, 4 Gy; OVAD, MOI = 1) after 7 days. **d** Quantification of survival fraction from

clonogenic survival assays. Statistical significance was assessed using one-way ANOVA with Tukey's multiple-comparisons test (**d**). Data are presented as mean ± s.e.m. from at least three independent experiments.

We respectfully hope that our responses and the newly added results satisfactorily address the reviewers' concerns.

We would again like to take this opportunity to sincerely thank the reviewers for their careful evaluation and constructive suggestions, which have been invaluable for improving the rigor, clarity, and overall quality of our work.

References

1. Wang Q., et al. Oncolytic adenovirus with MUC16-BiTE shows enhanced antitumor immune response by reversing the tumor microenvironment in PDX model of ovarian cancer. *Oncoimmunology*. **11**, 2096362 (2022).
2. Ding L., et al. An Inter-Supplementary Biohybrid System Based on Natural Killer Cells for the Combinational Immunotherapy and Virotherapy of Cancer. *Adv Sci*. **9**, e2103470 (2022).
3. Liao Y., et al. A cuproptosis nanocapsule for cancer radiotherapy. *Nat Nanotechnol*. **19**, 1892-902 (2024).
4. Lei G., et al. Radiotherapy promotes cuproptosis and synergizes with cuproptosis inducers to overcome tumor radioresistance. *Cancer Cell*. **43**, 1076-92.e5 (2025).
5. Tang D., Kroemer G., and Kang R. Targeting cuproplasia and cuproptosis in cancer. *Nat Rev Clin Oncol*. **21**, 370-88 (2024).
6. Wang S., et al. Near-Infrared Activatable Copper Nanoplatforms Synergize with the 5-Azacytidine Prodrug to Potentiate Cuproptosis. *Angew Chem Int Ed Engl*. **63**, e202411609 (2024).
7. Tsvetkov P., et al. Copper induces cell death by targeting lipoylated TCA cycle proteins. *Science*. **375**, 1254-61 (2022).
8. Lei G., et al. Cuproptosis-immunity crosstalk informs strategy to overcome immunotherapy resistance. *Cell*. https://doi.org/10.1016/j.cell.2026.05.036 (2026).
9. An L., Li M., and Jia Q. Mechanisms of radiotherapy resistance and radiosensitization strategies for esophageal squamous cell carcinoma. *Mol Cancer*. **22**, 140 (2023).
10. Siegel R. L., Kratzer T. B., Giaquinto A. N., Sung H., and Jemal A. Cancer statistics, 2025. *CA Cancer J Clin*. **75**, 10-45 (2025).
11. Petroni G., Cantley L. C., Santambrogio L., Formenti S. C., and Galluzzi L. Radiotherapy as a tool to elicit clinically actionable signalling pathways in cancer. *Nat Rev Clin Oncol*. **19**, 114-31 (2022).
12. Walter K. Head and Neck Cancer. *JAMA*. https://doi.org/10.1001/jama.2026.4962 (2026).
13. Dehghankelishadi P., et al. Formulation of simvastatin within high density lipoprotein enables potent tumour radiosensitisation. *J Control Release*. **346**, 98-109 (2022).
14. Rutihinda C., et al. Inhibition of the CCR6-CCL20 axis prevents regulatory T cell recruitment and sensitizes head and neck squamous cell carcinoma to radiation therapy. *Cancer Immunol Immunother*.

72, 1089-102 (2023).
15. Feng C., et al. METTL3/CD98-mediated glutamate efflux in CAFs drives CD8(+) T cell exhaustion and impedes neoadjuvant immunochemotherapy. *Cell Rep*. **45**, 116933 (2026).
16. Zhang L., et al. Integration of AIEgens into covalent organic frameworks for pyroptosis and ferroptosis primed cancer immunotherapy. *Nat Commun*. **14**, 5355 (2023).
17. Li H., et al. Theranostic near-infrared-IIb emitting nanoprobes for promoting immunogenic radiotherapy and abscopal effects against cancer metastasis. *Nat Commun*. **12**, 7149 (2021).
18. Zhang L., et al. Activation of Pyroptosis Using AIEgen-Based sp(2) Carbon-Linked Covalent Organic Frameworks. *J Am Chem Soc*. **145**, 17689-99 (2023).
19. Zhang L., et al. Trigger inducible tertiary lymphoid structure formation using covalent organic frameworks for cancer immunotherapy. *Nat Commun*. **16**, 44 (2025).
20. Zhao Y., Tan F., Zhao J., Zhou S., Luo Y., and Gong C. Targeting the Enhanced Sensitivity of Radiotherapy in Cancer: Mechanisms, Applications, and Challenges. *MedComm*. **6**, e70202 (2025).
21. Bodei L., Herrmann K., Schöder H., Scott A. M., and Lewis J. S. Radiotheranostics in oncology: current challenges and emerging opportunities. *Nat Rev Clin Oncol*. **19**, 534-50 (2022).
22. De Ruysscher D., Faivre-Finn C., Le Pechoux C., Peeters S., and Belderbos J. High-dose re-irradiation following radical radiotherapy for non-small-cell lung cancer. *Lancet Oncol*. **15**, e620-e24 (2014).
23. Darragh L. B., and Karam S. D. Radiation as an immune modulator: mechanisms and implications for combination with immunotherapy. *Nat Rev Cancer*. **26**, 270-84 (2026).
24. Zhang L., et al. Radioactive Diselenide Bonded Covalent Organic Framework. *Adv Mater*. **37**, e2413002 (2025).
25. Gao S., Li T., Guo Y., Sun C., Xianyu B., and Xu H. Selenium-Containing Nanoparticles Combine the NK Cells Mediated Immunotherapy with Radiotherapy and Chemotherapy. *Adv Mater*. **32**, e1907568 (2020).
26. Zhang L., et al. Discovery of highly fluorescent covalent organic frameworks through AI-assisted iterative experiment-learning cycles. *Nat Chem*. **17**, 1645-54 (2025).
27. Silver N. L., et al. Intratumoral bacteria are immunosuppressive and promote immunotherapy resistance in head and neck squamous cell carcinoma. *Nat Cancer*. **7**, 80-97 (2026).
28. Zeng S., et al. A Photon-Driven Unimolecular Immunostimulant for Self-Amplified Pyroptosis and cGAS-STING Pathway by Destroying the Pyroptosis Checkpoint. *Angew Chem Int Ed Engl*. **64**, e202513815 (2025).

<u>Responses to the comments of Reviewer #1</u>

<u>Comment:</u>

The authors have largely addressed the comments made by this reviewer. The overall quality and rigor of this study have significantly improved. The revised manuscript has met the requirements for its publication in NC.

The recent published work on the role of cuproptosis in ICD and antitumor immunity (PMID: 42330950) is highly relevant to this study. This recent publication is cited in the revised manuscript (reference 48) but without any discussion. The authors should add a brief discussion to put their findings in the context of this highly relevant study.

<u>Response:</u>

We sincerely thank the reviewer for the positive assessment of our revised manuscript and for recognizing the improvements in its quality and rigor. We also appreciate the reviewer's helpful suggestion regarding this highly relevant recent study. We agree that this work provides a valuable mechanistic framework for understanding the relationship between cuproptosis and antitumor immunity, and that our previous revision did not adequately discuss its relevance to our observations. Accordingly, we have added a brief discussion to place our findings in the context of this study and to clarify how our observations are consistent with its conclusions.

Specifically, the Discussion section has been revised as follows:

"Notably, a recent study provided important mechanistic insights into the reciprocal interplay between cuproptosis and antitumor immunity, demonstrating the therapeutic potential of exploiting this crosstalk to overcome immunotherapy resistance [48]. Consistent with these findings, our results show that cuproptosis induction is associated with features of immunogenic cell death and enhanced antitumor immune responses, further supporting the close connection between cuproptosis and tumor immunity."

Responses to the comments of Reviewer #2

Comment:

Following rigorous revision and the addition of substantial experimental evidence, the regulatory mechanisms and immunological rationale are now thoroughly substantiated. Key additions include in vivoCD4$^+$/CD8$^+$ T cell depletion assays, co-immunoprecipitation validation of DLAT lipoylation, systematic characterization of intratumoral viral replication, bilateral tumor models to evaluate the abscopal effect, and standardized documentation of all flow cytometry gating strategies. Consequently, we believe the manuscript meets the publication criteria for Nature Communications, and we respectfully request the Editor's consideration for acceptance

Response:

We sincerely thank the reviewer for the positive assessment of our revised manuscript and for recognizing the additional mechanistic and immunological evidence incorporated during revision. We greatly appreciate the reviewer's constructive comments throughout the review process, which have substantially improved the rigor and clarity of our study.

Responses to the comments of Reviewer #3

Comment:

Many data can't support its conclusion. For instance, Figure S30e didn't show "the triple combination further promoted DLAT oligomerization" and S30f can't compare with 30e seperately to support the statement of "enhanced molecular phenotype remains copper-dependent".

Response:

We thank the reviewer for identifying this overinterpretation. We agree that Supplementary Fig. 30e supports the presence of DLAT oligomerization under the triple-combination condition but does not demonstrate that the triple combination further increased DLAT oligomerization relative to the other treatment groups. We also

agree that Supplementary Fig. 30e and 30f were obtained from separate immunoblot experiments and therefore cannot be directly compared to conclude that TTM completely abrogated this molecular phenotype. Accordingly, we have removed the comparative and definitive wording, including “further promoted,” “completely abrogated,” and “confirming that this enhanced molecular phenotype remains copper-dependent.” The revised text now describes the observations from the two experiments independently without making a direct quantitative comparison between Supplementary Fig. 30e and 30f.

Specifically, the manuscript has been revised as follows:

“Mechanistically, the triple combination was accompanied by detectable DLAT oligomerization, a canonical biochemical hallmark of cuproptosis (Supplementary Fig. 30e). In a separate experiment performed in the presence of the copper chelator TTM, no apparent DLAT oligomerization was detected under the triple-combination condition (Supplementary Fig. 30f).”

In response to the reviewer’s concern, we also carefully re-examined the relevant conclusions throughout the manuscript to ensure that the wording remains limited to what is directly supported by the presented data.

We would again like to take this opportunity to sincerely thank the reviewers for their careful evaluation and constructive suggestions, which have been invaluable for improving the rigor, clarity, and overall quality of our work.
